# Serum Proteomics of Experimental Malaria-Associated ARDS Reveals a Regulation of Acute-Phase Response Proteins

**DOI:** 10.1155/jimr/5642957

**Published:** 2025-03-23

**Authors:** Lívia Rosa-Fernandes, Verônica Feijoli Santiago, Yasmin da Silva-Santos, Tissiane Tarosso Lopes, Erika Paula Machado Peixoto, Stefani Aparecida Minchio Rodrigues, Claudio Romero Farias Marinho, Giuseppe Palmisano, Sabrina Epiphanio

**Affiliations:** ^1^Department of Parasitology, Institute of Biomedical Sciences, University of São Paulo, São Paulo, Brazil; ^2^Department of Clinical and Toxicological Analysis, Faculty of Pharmaceutical Sciences, University of São Paulo, São Paulo, Brazil; ^3^School of Natural Sciences, Macquarie University, Sydney, New South Wales, Australia

## Abstract

Malaria is a parasitic infectious disease considered a public health problem. Acute respiratory distress syndrome (ARDS) is a complication in malaria-infected individuals with a high mortality rate (80% to 100%) and can occur before, during, or after antimalarial drug treatment. Although inflammation and epithelial/endothelial injury pathways have been determined through these studies, specific circulating malaria-associated ARDS markers have not yet been established. We applied a quantitative mass spectrometry (MS)-based proteomic approach to identify altered molecular pathways in a mouse model of malaria-associated ARDS. Acute-phase response (APR) proteins were regulated in the ARDS group, suggesting their potential involvement in the development of the syndrome. They may serve as biomarkers when analyzed alongside other proteins that require further investigation. Additionally, the regulation of APR proteins in the ARDS group provides valuable insights into the pathophysiology of ARDS, contributing to a better understanding of the syndrome.

## 1. Introduction

Malaria is still a serious public health problem worldwide, with increased cases during the COVID-19 pandemic, which affected 247 million people and caused 619,000 deaths in 2021, with a rise of 13.4 million cases between 2019 and 2021 [1]. Severe malaria, caused by *P. falciparum*, can be recognized as cerebral malaria, multiple organ failure, severe anemia, and respiratory complications such as acute respiratory distress syndrome (ARDS). The disease is a consequence of *P. falciparum*-infected erythrocyte sequestration in the deep vasculature of the lung [2], which bind to lung endothelial cells through PfEMP1-ICAM-1 and EPCR interactions [3]. Loss of function and increased permeability of the alveolar–capillary barrier of the lungs are also observed, resulting in pulmonary edema of noncardiogenic origin, decreased gas exchange capacity, increased leukocyte activity and inflammatory mediators, and consequent respiratory failure in critically ill patients [4–6]. Malaria-associated ARDS is a significant cause of death in adults, with a high mortality rate (80% to 100%), and can occur before, during, or after antimalarial drug treatment [7–9]. Despite our understanding of the pathogenesis mechanisms involved, no reliable diagnostics can predict the onset of ARDS. The underlying mechanisms responsible for the transition from uncomplicated malaria to its severe form are still unknown.

Mass spectrometry (MS)-based proteomic strategies map the proteome, post-translational modifications (PTMs), protein–protein interaction, and protein conformational changes in complex biological systems [10]. These analyses can be done without prior knowledge, allowing the discovery of protein signatures and altered pathways that can inform the pathophysiological state of an organism [11]. Proteomic approaches have been applied to identify biomarkers for ARDS, establish prognosis, and understand the pathogenesis of the disease [12, 13]. Although inflammation and epithelial/endothelial injury pathways have been determined through these studies, specific malaria-associated ARDS markers have not yet been established [14, 15]. Moreover, plasma and serum biomarkers are extremely valuable for understanding the pathological changes in ARDS and the development and recovery of the disease, mainly due to the difficulty in collecting lung tissue. Indeed, circulating blood proteins may come from different tissues, such as alveolar epithelial cells and vascular endothelium [16, 17]. Understanding early events leading to the development of ARDS would allow specific progression biomarkers and the design of effective and innovative strategies for prevention and intervention before disease aggravation. In this study, we applied a MS-based proteomic approach to characterize altered molecular pathways in the previously described murine model of malaria-associated ARDS [18–20]. Our initial results show a specific serum protein panel that could discriminate ARDS complications due to *P. berghei* infection in mice. Furthermore, acute-phase response (APR) was enriched within the datasets and further investigated within the regulated processes.

## 2. Materials and Methods

### 2.1. Animals, Ethical Statement, and Euthanasia

DBA/2 male mice (purchased from the Biomodel Research Center from the School of Pharmaceutical Sciences, University of São Paulo, Brazil), 6–8 weeks old, were maintained in a 12/12-h light/dark cycle with food and water ad libitum. All experiments were approved by the Animal Health and Committee of the School of Pharmaceutical Sciences of the University of São Paulo (protocol number 590/2019) and executed according to the ethical guidelines established by the National Council for Control of Animal Experimentation (CONCEA) and the Brazilian Federal Law no. 11.794. Euthanasia, using ketamine (150 mg/kg)/xylazine (15 mg/kg), was performed at chosen time points or when mice demonstrated suffering or signaled imminent death (humane endpoint). All efforts were accomplished to minimize animal sorrow.

### 2.2. Infection and Parasitemia

DBA/2 mice were infected with 1 × 10^6^*Plasmodium berghei* ANKA (clone1.49L) (PbA)-infected red blood cells (iRBCs) by intraperitoneal route. Parasitemia and weight were monitored daily from the fifth day postinfection (DPI) onward. Parasitemia was determined by Giemsa staining (blood smear) and DRAQ5 nucleic acid marker (DRAQ5 Abcam) using flow cytometry (BD FACSCalibur) and expressed as the percentage of iRBCs.

### 2.3. Serum Proteomics Analysis

#### 2.3.1. Animal Model and Endpoints

Noninfected control (CTRL) and infected DBA/2 mice were euthanized on the 7 and 9 DPIs. During necropsy, mice were assessed for the presence/absence of pleural effusion and pale or red and congested lungs. Mice with pleural effusion and red and congested lungs were diagnosed with ARDS, and posteriorly, pulmonary histological analyses confirmed ARDS, showing hemorrhage and alveolar and interstitial edema, inflammatory cells (neutrophils and mononuclear cells), and, sometimes, hyaline membranes, according to previously studies [19]. Whole blood was collected by cardiac puncture, transferred to a dry tube, and incubated for 30 min before centrifugation at 2000× *g* for 10 min. Serum was collected and stored at −80°C.

#### 2.3.2. Direct Serum Protein Extraction

Mouse serum samples were mixed with five volumes of denaturation buffer consisting of 8 M urea, complete protease inhibitor (Roche), PhosSTOP phosphatase inhibitor (Roche), 50 mM TEAB, and 10 mM dithiothreitol (DTT). Protein reduction, alkylation and digestion are described below.

#### 2.3.3. DTT/Acetonitrile (ACN) Serum Protein Extraction

Albumin concentration was reduced by sequential application of DTT/ACN, according to de Jesus et al. [21], with modifications. Briefly, mouse serum samples were mixed with 10% v/v of 500 mM DTT in 20 mM TEAB and vortexed. The sample was incubated at RT for 60 min and centrifuged at 13,000× *g* for 40 min. Supernatants were collected and diluted 3× with MilliQ water before adding 1 volume of ACN. Samples were bath-sonicated for 10 min, vortexed and further sonicated for 10 min, and centrifuged at 13,000× *g* for 10 min at room temperature. Finally, the supernatants were vacuum-dried and reconstituted in urea denaturation buffer. Protein reduction, alkylation, and digestion are described here.

#### 2.3.4. Trichloroacetic Acid (TCA)/Acetone Serum Protein Extraction

Albumin concentration was reduced using TCA/acetone precipitation, according to Chen et al. [22], with modifications. Briefly, mouse serum samples were precipitated by adding four volumes of 10% (w/v) TCA in ice-cold acetone and mixed by vortexing. The mixture was incubated at −20°C overnight and centrifuged at 15,000× *g*, 4°C, for 15 min. Then, the supernatant was removed, and the precipitate was left to dry at room temperature before resuspension in urea denaturation buffer. Protein reduction, alkylation, and digestion are described here.

#### 2.3.5. Protein Reduction, Alkylation, and Digestion

Sample preparation for MS-based proteomics was carried out as previously described [23]. Samples in denaturation buffer were incubated for 45 min at 30°C and alkylated in 40 mM iodoacetamide (IAA) for 30 min at room temperature in the dark. Following incubation, the samples were diluted to 1 M urea final concentration, and proteins were quantified with Qubit fluorescence detection system (Thermo Fisher Scientific) before trypsin digestion (Promega) at 1:50 (m/m) at 30°C overnight. Trifluoroacetic was added to 1% (v/v) final concentration, and peptide solutions were desalted using C18 reverse phase microcolumns (3M Empore) [24] and vacuum dried.

#### 2.3.6. Nano-LC-MS/MS Analysis

Peptide samples were resuspended in 0.1% FA before analysis using an EASY-nLC system (Thermo Fisher Scientific) coupled to LTQ-Orbitrap Velos mass spectrometer (Thermo Fisher Scientific). The peptides were loaded on Acclaim PepMap C18 (Thermo, Germany) trap column (2 cm × 100 µm inner diameter; 5 µm) and separated onto a Reprosil-Pur C18-AQ (15 cm × 75 µm inner diameter; 3 µm) column and separated with a gradient from 100% mobile phase A (0.1% FA) to 35% phase B (0.1% FA, 95% ACN) during 75 min, 35%–55% in 25 min, 55%–55% in 2 min, and 18 min at 95% at a constant flow rate of 300 nL/min. The LTQ-Orbitrap Velos was operated in positive ion mode with data-dependent acquisition. The full scan was acquired in Orbitrap with an automatic gain control (AGC) target value of 10e^6^. Each precursor ion scan was obtained by a resolution of 60,000 FWHM in the 350–1800 m/z mass range. Peptide ions were fragmented by CID with a normalized collision energy of 35. The 20 most abundant peptides were selected for MS/MS and dynamically excluded for 15 s.

#### 2.3.7. Database Search

Raw data from large-scale DDA proteomic experiments were processed using Thermo Proteome Discoverer v3.0.0.757 (Thermo Fisher Scientific) and searched against the UniProt *Mus musculus* reference database (August 2022) using the embedded Sequest HT server with a MS accuracy of 10 ppm and 0.6 Da for MS/MS. Cysteine carbamidomethylation (57.021 Da) was set as fixed modification, and two missed cleavages for trypsin. Methionine oxidation (15.994 Da) and protein N-terminal acetylation (42.010 Da) were set as variable modifications. PSMs, proteins, and peptides were accepted at less than 1% FDR. The datasets generated for this study have been deposited to the ProteomeXchange Consortium via the PRIDE (http://www.ebi.ac.uk/pride) partner repository under the project accession PXD050496.

#### 2.3.8. Bioinformatics Analyses

Principal component analysis (PCA) was performed using all identified proteins with two or more unique peptides. Quantitative analysis was conducted on the log2 values or log2 ratio of the normalized abundances using Perseus v1.6.10.50. Protein regulation among groups was accessed by ANOVA multiple-sample test with Benjamini–Hochberg correction (*q*-value < 0.05). Post hoc Tukey's HSD was performed on the ANOVA, and significant differences were found with FDR 0.05. The term “upregulated” describes proteins and peptides more abundant in a given comparison, while “downregulated” refers to those less abundant ones.

Protein–protein interaction networks were constructed using STRING database [25] with high confidence parameter (0.700) uploaded in Cytoscape v3.8.1 [26]. ClueGo plug-in v2.5.7 [27] was used for enrichment analysis with 5% FDR. Overrepresented pathways, upstream regulators, and biological and cellular functions were derived from Ingenuity Pathway Analysis (Qiagen). Gene ontology, pathway, and disease enrichment analysis were also conducted on the web-based ToppGene Suite [28] with an FDR correction of 1%.

### 2.4. Orthogonal Validation

#### 2.4.1. Animal Model and Endpoints

Infected DBA/2 mice were divided into two groups. Respiratory patterns were monitored on 7 DPI. The noninfected CTRL and experimental groups were euthanized on the 7 DPI, while the survival group was monitored for 17 days. In this model, around 50% of the survival group develop ARDS (characteristics described in the topic “Serum Proteomics Analyses”) and die between 7 and 12 DPI. On the other hand, mice that develop hyperparasitemia (HyP) show pale lungs with black spots (malaria pigment), chronic interstitial pneumonia, and severe anemia, dying between 13 and 20 DPI [19].

The group euthanized on the 7 DPI was retrospectively diagnosed as ARDS or HyP-developing mice using respiratory patterns and the degree of parasitemia as predictive criteria, comparing their respiratory patterns and parasitemia measured on the 7 DPI with the cutoff values from the survival group (in which the cause of death is known), as previously described [19, 20, 29]. Respiratory patterns (respiratory frequency [RF] and enhanced pause [Penh]) were monitored by unrestrained whole-body plethysmography (WBP) chamber (Buxco Electronics, USA) for 10 min (basal level) according to previously described methods [19].

#### 2.4.2. Acute-Phase Protein Quantification and Analysis

SimpleStep ELISA technology (Abcam) measured serum amyloid A (Saa) proteins, following the manufacturer's protocols. The concentrations of Saa were measured in duplicate, interpolated from the Saa standard curve, and corrected for sample dilution.

Serum levels of C reactive protein (Crp), haptoglobin (Hp), alpha-1 acid glycoprotein (AGP, also known as orosomucoid Orm gene), and serum amyloid P protein (SAP, also known as pentraxin 2; Apcs gene) were measured by multiplex analysis, using the MILLIPLEX MAP Acute Phase Magnetic Bead Panel (Merck) according to the manufacturer's recommendation. Luminex xPONENT for LX100/LX200 software v3.1.871.0 (Luminex Corporation) was used for data acquisition. A five–parameter curve fit (logarithmic scale) on Belysa Experiment Report software v1.1.0 (SW instrument) was used for data analysis.

#### 2.4.3. Statistics

Sample outliers were detected and removed using the ROUT test (*Q* = 0.1%). The statistical differences between group means were tested by one-way ANOVA followed by Tukey's post-test for multiple comparisons. A value of *p*  < 0.05 was considered statistically significant in all analyses. Results are presented as mean ± SEM. All analyses were performed using GraphPad Prism v9.4.1.

## 3. Results

**Figure 1:**
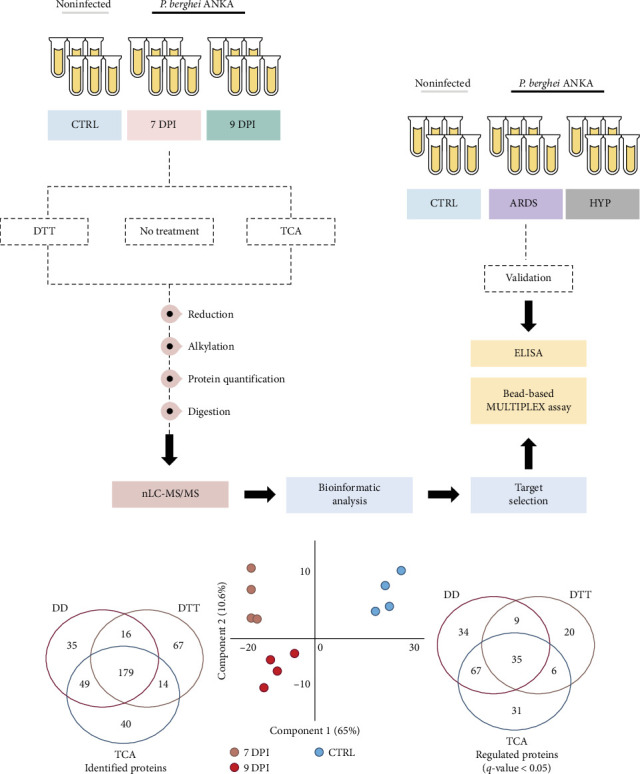
Serum proteome profiling of an experimental model of malaria-associated ARDS. (A) Workflow applied to characterize malaria-associated ARDS serum protein regulation. (B) Number of proteins identified using three methodological approaches. (C) Principal component analysis (PCA) based on all quantified proteins showed a modulation of the serum proteome profile upon pulmonary malaria. (D) Regulated proteins across methodological approaches (*q*-value < 0.05). DPI, days postinfection; DTT, dithiothreitol; TCA, trichloroacetic acid. Nontreatment: ARDS, acute respiratory distress syndrome; DD, direct digestion; HyP, hyperparasitemia; nLC-MS/MS, nanoliquid chromatography coupled mass spectrometry.

Blood protein levels mirror pathophysiological conditions and indicate possible circulating biomarkers in the bloodstream. To further characterize altered molecular pathways in the initial stages of malaria-associated ARDS, we analyzed serum from noninfected CTRL and PbA-infected DBA/2 (ARDS) mice diagnosed with ARDS on the 7 and 9 DPI (Figure 1A). Three sample preparation strategies were used to map quantitatively the serum proteome, including using (1) untreated, (2) TCA, and (3) a combination of DTT and ACN (Table S1–S3). Subsequently, tryptic peptides were analyzed using a bottom-up shotgun MS approach to obtain a broader coverage of the circulating proteome. Indeed, 400 proteins were identified, 179 common to the three methodologies (Figure 1B, Table S4). All quantified proteins were first analyzed by PCA, which displayed clear separation between ARDS and CTRL samples according to their total abundance variation (Figure 1C). However, ARDS from 7 and 9 DPI did not show the same separation. Differentially expressed proteins among noninfected CTRL and 7 and 9 DPI were selected with 5% false discovery rate (Figure 1D, Table S5) for each methodology before bioinformatic analysis.

**Figure 2:**
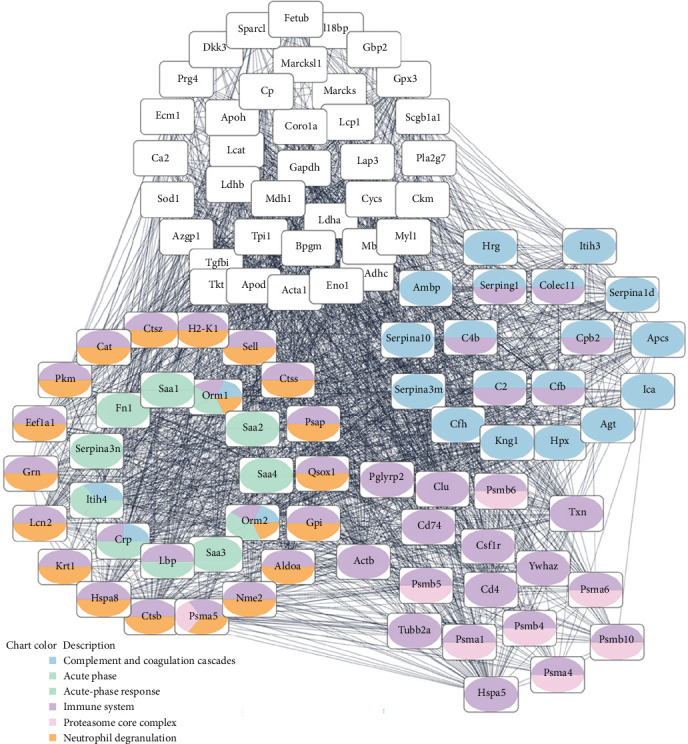
Network of upregulated serum protein malaria-associated acute respiratory distress syndrome (ARDS), indicating the interaction of proteins from acute-phase response, complement activation, neutrophil degranulation, and proteasomal core complex.

Functional analysis of proteins upregulated at 7 and 9 DPI revealed enrichment of APR, complement activation, neutrophil degranulation, and proteasomal core complex (Figure 2). We used the tissue proteome atlas [30] to better understand the origin of the identified proteins. Indeed, we could map the majority of up- and downregulated proteins produced by the liver (Figure S1).

**Figure 3:**
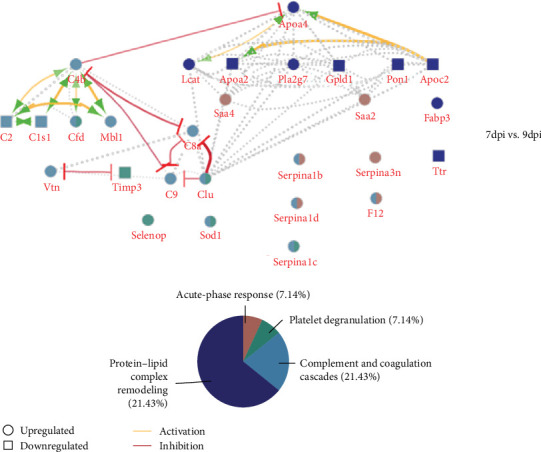
Serum protein modulation during malaria-associated acute respiratory distress syndrome (ARDS) progression. Network of regulated proteins between 7 and 9 days postinfection (DPI). Proteins represented in circles and squares are more and less abundant at 7 DPI, respectively. The yellow and red lines indicate process activation and inhibition, respectively. Biological process analysis indicated proteins belonging to four ontologies; acute-phase response (APR) proteins are represented in pink, platelet degranulation in green, complement and coagulation cascades in light blue, and protein–lipid complex in dark blue.

We have also addressed the time-resolved proteome modulation of serum from mice that developed malaria-associated ARDS. To directly evaluate the difference between the selected time points, we observed the regulation of 51 proteins between ARDS 7 and 9 DPI (*q*-value < 0.05) (Figure 3, Table S1–S4).

**Figure 4:**
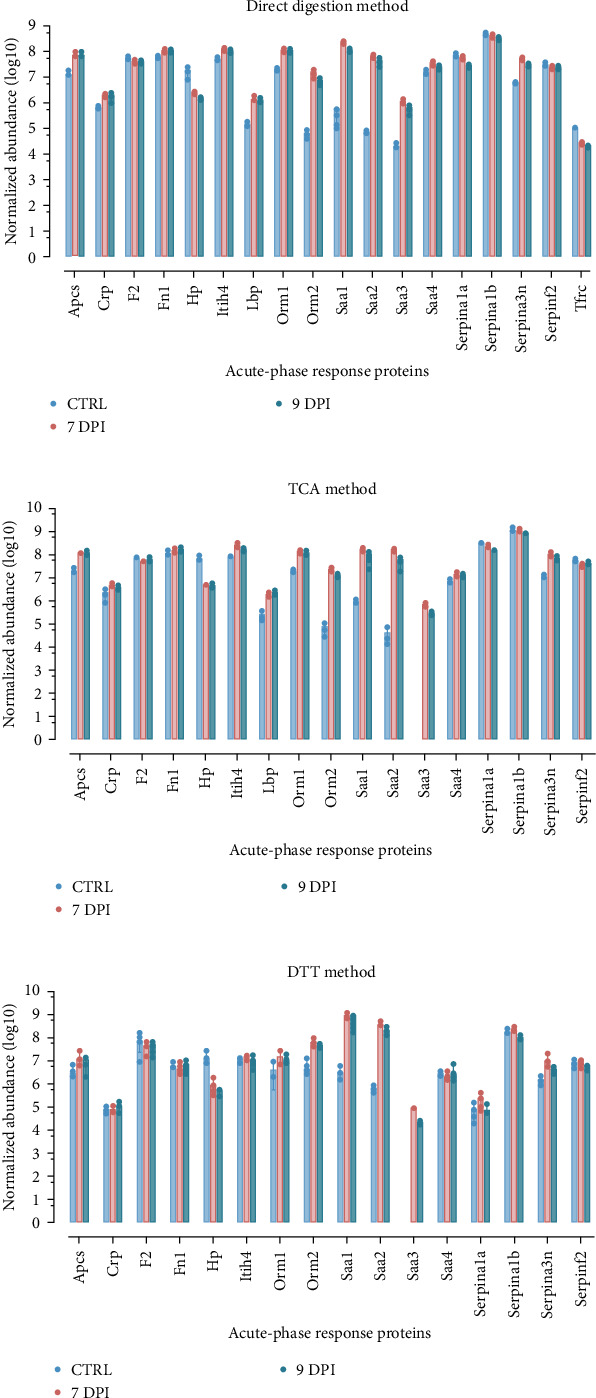
Normalized abundancy distribution (log10) of detected acute-phase response proteins in all serum samples. Bars are expressed as mean ± SEM: (A) direct digestion method; (B) trichloroacetic acid (TCA) method; and (C) dithiothreitol (DTT) method.

Interestingly, we could observe several APR proteins that were more abundant at the early time point. This prompted us to examine their normalized abundance distribution in all serum samples to observe if APR proteins could provide a discriminative signature (Figure 3). We identified Apcs; Crp; Orm 1 and Orm 2; Saa1, Saa2, and Saa3; Hp; Lbp; Itih4; and Serpina1a and Serpina3n proteins by at least two detection methods (DD, DDT, and TCA), and these proteins could provide a discriminative profile (Figure 4, Table S5–S7).

**Figure 5:**
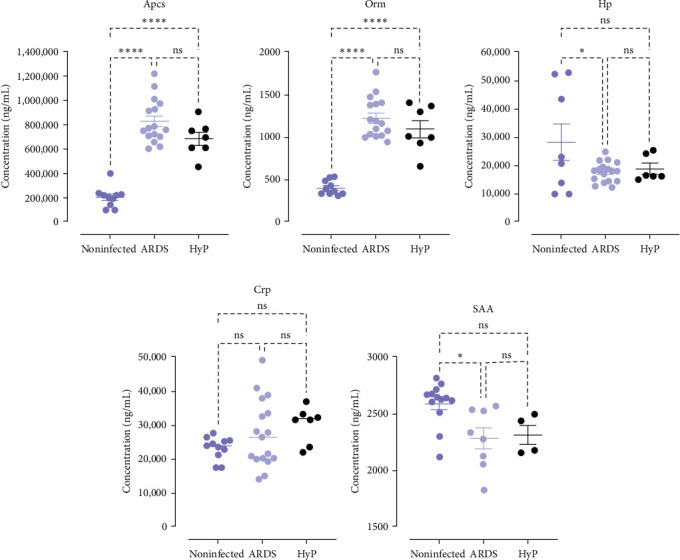
Expression of selected acute-phase response proteins in the serum, in an independent experimental cohort of noninfected controls (*n* = 11), ARDS-developing (*n* = 18), and HyP-developing mice (*n* = 9). (A–D) Serum levels of Apcs, Orm, Hp, and Crp were measured by multiplex analysis using the MILLIPLEX MAP Acute Phase Magnetic Bead Panel. (E) Saa was measured by SimpleStep ELISA (noninfected [*n* = 14]; ARDS-developing [*n* = 14]; and HyP-developing mice [*n* = 4]). Bars are expressed as mean ± SEM (*⁣*^*∗*^*p*-value < 0.05, *⁣*^*∗∗∗∗*^*p*-value < 0.0001, ns: not significant). Apcs, serum protein P, ARDS, acute respiratory distress syndrome; Crp, C-reactive protein; Hp, haptoglobin; HyP, hyperparasitemia; Orm, orosomucoid or alpha-1 acid glycoprotein; Saa, serum amyloid A.

To validate these findings, we analyzed the expression of Apcs, Orm, Crp, Hp, and Saa in the serum of an independent cohort of noninfected CTRL and PbA-infected mice. Based on the predictive model previously described [19, 29], we classified these infected mice as ARDS-developing mice or HyP-developing mice on the 7 DPI. We found that Apcs and Orm proteins were less abundant in the CTRL, being able to distinguish between infected and noninfected serum but showed no statistical difference between samples from ARDS-developing and HyP-developing mice (Figures 5A,B). Comparably, Hp was more abundant in the CTRL but showed a statistical difference only compared to ARDS-developing mice (Figure 5C). Crp, a common APR protein, and inflammation marker, did not show statistical difference between the three conditions (Figure 5D). We found more abundant Saa in the noninfected mice (Figure 5E), different from the results observed in the first assays for Saa1, Saa2, and Saa3 (Figure 4).

## 4. Discussion

Serum and plasma are widely used samples for diagnostic tests, being a circulating signature of proteins in both patho- and physiological states [31]. Nevertheless, the same content diversity observed as an advantage also poses as an analytical challenge for large-scale MS analysis [32]. The predominance of 22 highly abundant proteins, which make up to 99% of the total protein mass, poses an extra challenge for searching biomarkers [33]. The dynamic range of protein concentration spans at least 10 orders of magnitude, from albumin to less abundant cytokines. Simplifying the serum protein profile is key to mining the serum proteome [32].

The discovery phase of this study included three analytical strategies, direct digestion, TCA precipitation, and DTT/ACN treatment, to comprehensively cover the serum proteome. Combined DTT-/ACN-based depletion is a simple alternative in the depletion of thiol-rich proteins, such as albumin and transferrin by DTT, as well as of high molecular weight proteins by ACN [21]. Furthermore, combining TCA and acetone as precipitating agents has also been shown to be an effective albumin depletion strategy [22]. Indeed, combining these strategies allowed us to increase our protein identification by 30%, compared to direct digestion alone.

The pursuit of circulating biomarkers is an essential application of serum proteomics in malaria and has been employed in severe and uncomplicated malaria studies. For example, Huang et al. used a glycoprotein-focused enrichment strategy combined with MS-based proteomics to analyze the saliva collected from 17 children with uncomplicated malaria compared with age- and sex-matched malaria-negative cases. Host proteins involved in inflammation were upregulated in the infected group. Moreover, three *P. falciparum* proteins, PFL0480w, PF08_0054, and PFI0875w, were identified [34]. Recently, Venkatesh et al. [35] used a large-scale quantitative proteomics approach to analyze the proteome of *P. vivax*-infected individuals and parasite isolates [35]. Within the 38 proteins identified in human plasma from malaria cases, five proteins were also identified in *P. vivax* isolates suggesting potential diagnostic markers. In the infection model used here, we were not able to identify circulating parasite proteins.

The quantitative comparison of the proteome between severe and uncomplicated cases is a powerful tool for detecting changes associated with complications. Differential protein expression in the frontal lobe of patients with cerebral malaria was determined using isobaric labeling quantitative proteomics [36]. Proteins associated with the innate immune response and coagulation were upregulated, while myelination and oxidative stress regulation processes were downregulated. Bachmann J. et al. analyzed the plasma proteome of a cohort of 719 children with different malaria conditions and CTRL. A total of 1015 proteins were analyzed using an antibody array, and 13 proteins involved in oxidative stress and muscular and endothelial damage were differentially expressed between discriminated uncomplicated malaria from severe malaria syndromes [37]. In addition, serum proteomic analysis from patients with nonsevere and severe malaria infected by *P. falciparum* revealed 169 proteins (55 upregulated and 114 downregulated) and 179 proteins (60 upregulated and 119 downregulated), respectively, among APRs [38].

Here, we studied a murine model of malaria-associated ARDS developed in our group [18–20] to identify a circulating protein panel for the detection of ARDS-associated malaria. We identified APR among the enriched processes, even in an earlier stage of the disease.

APR comprises a systematic pattern of events in reply to tissue damage, inflammation, or infection, aiming at restoring homeostatic status. Upon local inflammatory response, the production of soluble mediators directs the metabolic response of the whole organism [39], which characteristically involves fever, leukocytosis, activation of complement and clotting cascades, and circulating neutrophils and their precursors. In parasitic infections, activation of monocytes or macrophages leads to the secretion of hepatocyte-stimulating factors, which in turn promotes the increase of expression and secretion of blood-circulating proteins by the liver [40]. Drastic changes in the plasma levels of acute phase proteins are detected in these conditions being up- and downregulated depending on the disease stage [41, 42]. These changes are rapid and guarantee clearance of the infection without eliciting persistent inflammation. The synthesis of these proteins is mainly regulated by proinflammatory cytokines such as IL-6, IL-1, and TNF-α [43, 44]. Elevated plasma levels of IL-1β and IL-6 have been suggested as predictors of poor outcome in ARDS patients [45]. On the other hand, it was shown that increased IL-10, an anti-inflammatory cytokine, in the lungs promotes microbiota dysbiosis and induces ARDS in a murine model [46].

APRs as serum protein P (Apcs), fibronectin (Fn1), inter-alpha-trypsin inhibitor, heavy chain 4 (Itih4), lipopolysaccharide-binding protein (Lbp), orosomucoid or alpha-1 acid glycoprotein (Orm1 and Orm2), Saa (Saa1, Saa2, Saa3, and Saa4), Crp, prothrombin (F2), Hp, serine protease inhibitor (Serpina1a, Serpina1b, and Serpina 3 n), alpha-2-antiplasmin (Serpinf2), and transferrin receptor protein (Tfrc) were differentially expressed in the serum proteome of mice with ARDS complications due to malaria infection.

Multiplex and ELISA assays confirmed the regulation of Orm, Apcs, Hp, and Saa proteins in ARDS-developing mice compared to CTRL on the 7 DPI. Notwithstanding, it was not possible to differentiate these proteins between mice with the development of ARDS and HyP. We believe that earlier or later time points (instead of 7 DPI) could show more significant differences. However, the predictive model to classify mice in ARDS or HyP groups previously developed by Ortolan et al. [19] is unable to predict causes of death before or after the 7 DPI.

Serum amyloid protein A is involved in APR during infection [47–50]. Indeed, its plasma levels can increase up to 1000-fold. Four isoforms have been described Saa1, Saa2, Saa3, and Saa4. Together with Crp, Saa1 and Saa2 levels are used as markers of infections and can indicate a chronic pathological status if persistently high. Indeed, Crp and Saa show the highest increase during APR in humans [51]. Meta-analysis study regarding Crp levels revealed Crp as a biomarker for the early identification and supervising of malaria gravity [52]. However, our data from multiplex analyses of Crp did not show any difference among the groups. On the other hand, exponential reduction in the biological half-life of Crp and Saa occurred in both patients and murine models. In a study of community-acquired pneumonia in patients, Crp and Saa levels decreased by around 50% and more than 25% in 3–5 days and 7–14 days, respectively, after the first examination (admission) [53]. Using Crp iodine, Vigushin and colleagues show that the half-life of Crp was 4 h in mice and 7 h in rabbits. On the other hand, in patients with inflammatory and bacterial diseases (acute or chronic) or healthy individuals, the half-life of CPR ranged from 14 to 19,9h [54].

Surprisingly, ELISA measurement showed a higher concentration of Saa in the noninfected CTRL. We hypothesize that the difference is due to the combined assessment of Saa 1, 2, 3, and 4 by the assay when we clearly observed changes in Saa1 and 2 levels, while no difference was observed in Saa4 levels with our LC-MS/MS strategy. Another possibility is that clearance has already occurred, as previously mentioned, due to the time point.

Patients with liver diseases are at high risk of developing ARDS suggesting the existence of the liver–lung axis [55]. Saa1 partially protects mice against lung injury caused by LPS treatment, cecal ligation, and puncture [56]. A complex formed by Saa1 binding to LPS was shown to contribute to this effect. Interestingly, it has been shown that in ARDS caused by pneumonia, pulmonary immune cells secrete cytokines that induce the expression of APRs in the liver [57, 58]. The APRs migrate to the lung causing local inflammation due to the activation of alveolar macrophages [59]. Besides this function, hepatic APRs exert host defense against pathogens and protect the liver attenuating systemic inflammation [60, 61].

Even though we only observed a statistical difference in Hp concentration between noninfected and ARDS-developing mice, low levels of this protein were used as a marker of malaria prevalence in endemic areas confirming the downregulation found in our data [62]. Hp exerts an anti-inflammatory activity, and its biosynthesis in the liver is stimulated by IL-6 [63]. Previously, we also demonstrated that IL-6 levels are increased in *Plasmodium berghei* ANKA-infected DBA/2 mice compared with noninfected mice [64]. Moreover, this protein is toxic to *P. falciparum* in vitro [65]. In infected individuals, the plasma levels of Hp are reduced during malaria [66]. Binding of Hp to free hemoglobin with subsequent clearance is reported as the cause of low levels of Hp in the blood [67]. Ray et al. [38] studied nonsevere and severe malaria in *P. falciparum* patients and showed a significant decrease in Hp compared with healthy people [38]. These data corroborated our study since decreased levels of Hp were significantly reduced in *Plasmodium*-infected mice with ARDS complications but not in HyP-developing mice.

Due to that, APRs have been suggested as potential biomarkers in *P. falciparum*-infected human sera [68]. Our data support this hypothesis and add another layer of selectivity, showing their role in detecting severe malaria, including ARDS-associated complications. On the other hand, we recognize the limitation of the study since mice that ARDS-developing mice also did not show differences between HyP-developing mice. Furthermore, we must remember that HyP-developing mice also have severe disease (in the end of infection) once they die from chronic interstitial pneumonia, HyP, and severe anemia.

It would be exciting and essential to evaluate different murine models of severe malaria (cerebral malaria, hepatic, and renal insufficiency) and compare them with self-limiting and asymptomatic infection models. However, we could not carry out these experiments due to the difficulties of establishing and comparing models with some strains of mice with different parasites, dosages, and time points.

## 5. Conclusions

The work developed here intended to characterize the expression of serum proteins in malaria-associated ARDS. Samples of a well-defined and controlled murine model of malaria-associated ARDS were analyzed by quantitative proteomic analysis, and two time points were chosen to evaluate the progression of the disease. An unbiased and large-scale approach initially allowed us to identify several novel candidate proteins to model the pathogenesis of malaria-associated ARDS in mice. Indeed, serum proteomics analysis allowed us to integrate some of the results observed in our previous studies with systemic response to disease severity. Our primary focus was to investigate whether APR proteins could serve as potential biomarkers for malaria-associated ARDS, a condition where diagnostic methods remain a significant challenge. Our findings demonstrated that these proteins were modulated in this disease context. Although this work offers perspectives on previously described markers that could be repositioned and utilized as noninvasive diagnostic tools, further studies are required to develop reliable prognostic tools. Additionally, validation using orthogonal techniques is essential to confirm the dysregulation of these processes

## Data Availability

All data are available from the corresponding authors.

## Supporting Information

Supporting Information Table S1: List of serum proteins identified and quantified using the direct digestion approach in the conditions control (CTRL), 7 DPI, and 9 DPI. Table S2: List of serum proteins identified and quantified using the DTT approach in the conditions control (CTRL), 7 DPI, and 9 DPI. Table S3: List of serum proteins identified using the TCA approach in the conditions control (CTRL), 7 DPI, and 9 DPI. Table S4: Compiled list of serum proteins identified and quantified using the three approaches in the conditions control (CTRL), 7 DPI, and 9 DPI. Table S5: Serum proteins differentially regulated in the conditions control (CTRL), 7 DPI, and 9 DPI. Table S6: Analysis of variance of differentially regulated serum proteins in control (CTRL), 7 DPI, and 9 DPI. Table S7: Analysis of variance per method of differentially regulated serum proteins in control (CTRL), 7 DPI, and 9 DPI conditions. Significance <0.05 by ANOVA and 9 DPI conditions. Figure S1: Protein atlas origin analysis indicated the liver as the main source of ARDS-modulated proteins.
