# Supplementary material for: Serum Proteomics of Experimental Malaria-Associated ARDS Reveals a Regulation of Acute-Phase Response Proteins
Source: J Immunol Res. 2025 Mar 23;2025:5642957. doi: 10.1155/jimr/5642957 (PMC11955258; doi:10.1155/jimr/5642957)
Supplement: Supporting Information — Table S1: List of serum proteins identified and quantified using the direct digestion approach in the conditions control (CTRL), 7 DPI, and 9 DPI. Table S2: List of serum proteins identified and quantified using the DTT approach in the conditions control (CTRL), 7 DPI, and 9 DPI. Table S3: List of serum proteins identified using the TCA approach in the conditions control (CTRL), 7 DPI, and 9 DPI. Table S4: Compiled list of serum proteins identified and quantified using the three approaches in the conditions control (CTRL), 7 DPI, and 9 DPI. Table S5: Serum proteins differentially regulated in the conditions control (CTRL), 7 DPI, and 9 DPI. Table S6: Analysis of variance of differentially regulated serum proteins in control (CTRL), 7 DPI, and 9 DPI. Table S7: Analysis of variance per method of differentially regulated serum proteins in control (CTRL), 7 DPI, and 9 DPI conditions. Significance <0.05 by ANOVA and 9 DPI conditions. Figure S1: Protein atlas origin analysis indicated the liver as the main source of ARDS-modulated proteins. [file 5642957.f1.pdf]

SUPPLEMENTARY INFORMATION

SERUM PROTEOMICS OF EXPERIMENTAL MALARIA-ASSOCIATED ARDS REVEALS A REGULATION OF ACUTE PHASE RESPONSE PROTEINS

Lívia Rosa-Fernandes[1#], Verônica Feijoli Santiago [1], Yasmin da Silva-Santos[2], Tissiane Tarosso Lopes[2], Erika Paula Machado Peixoto[1], Stefani Aparecida Minchio Rodrigues[2], Claudio Romero Faria Marinho[1], Giuseppe Palmisano[1,3#&], Sabrina Epiphanio[2#&]

1 Department of Parasitology, Institute of Biomedical Sciences, University of São Paulo, São Paulo, São Paulo, Brazil.

2 Department of Clinical and Toxicological Analysis, Faculty of Pharmaceutical Sciences, University of São Paulo, São Paulo, Brazil

3 School of Natural Sciences, Macquarie University, Sydney, New South Wales, Australia.

liviarosa.f@gmail.com, palmisano.gp@usp.br and sabrinae@usp.br
&: These authors contributed equally

Supplementary Table 1: list of serum proteins identified and quantified using the direct digestion approach in the conditions control (CTRL), 7DPI and 9DPI.

Supplementary Table 2: list of serum proteins identified and quantified using the DTT approach in the conditions control (CTRL), 7DPI and 9DPI.

Supplementary Table 3: list of serum proteins identified using the TCA approach in the conditions control (CTRL), 7DPI and 9DPI.

Supplementary Table 4: compiled list of serum proteins identified and quantified using the three approaches in the conditions control (CTRL), 7DPI and 9DPI.

Supplementary Table 5: serum proteins differentially regulated in the conditions control (CTRL), 7DPI and 9DPI.

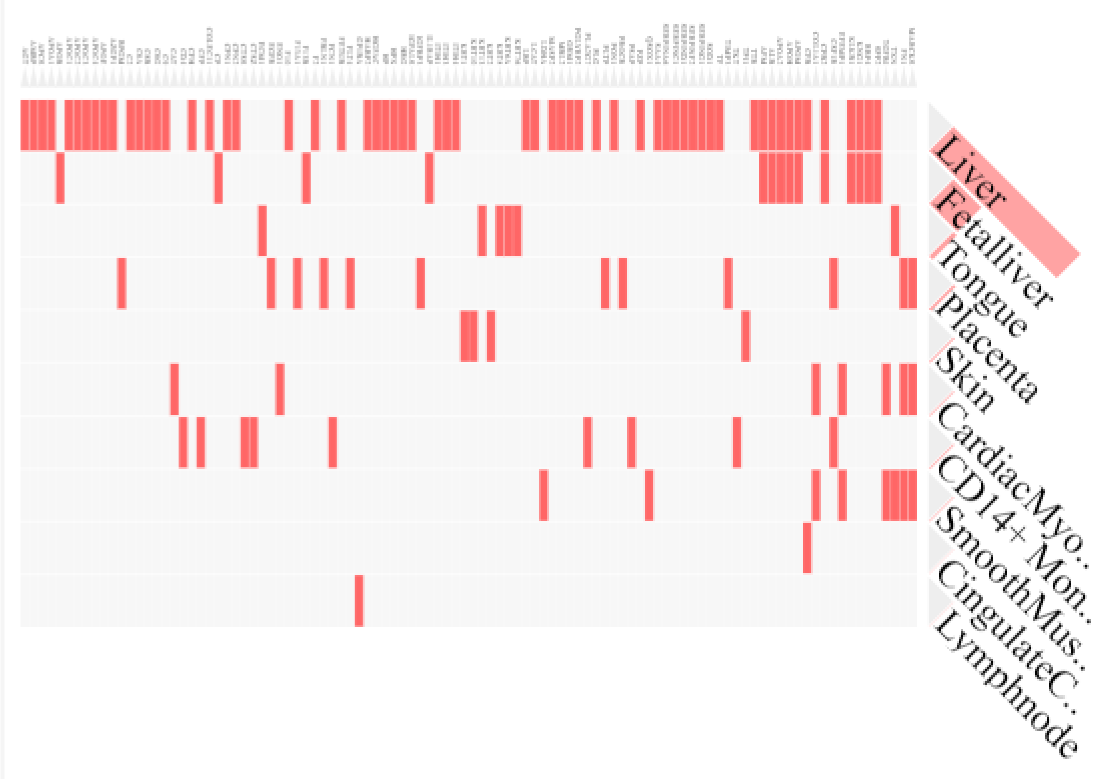

Supplementary Figure 1: Protein atlas origin analysis indicated the liver as the main source of ARDS modulated proteins

Supplementary Table 1: list of serum proteins identified and quantified using the direct digestion approach in the conditions control (CTRL), 7dpi and 9dpi

Accession	Description	Gene Name	# PSMs	# Unique Peptides	Abundance Ratio: (7dpi) / (CTRL)	Abundance Ratio: (9dpi) / (CTRL)	Abundances (Normalized): F1: Sample, CTRL	Abundances (Normalized): F2: Sample, CTRL	Abundances (Normalized): F3: Sample, CTRL	Abundances (Normalized): F4: Sample, CTRL	Abundances (Normalized): F5: Sample, 7dpi	Abundances (Normalized): F6: Sample, 7dpi	Abundances (Normalized): F7: Sample, 7dpi	Abundances (Normalized): F8: Sample, 7dpi	Abundances (Normalized): F9: Sample, 9dpi	Abundances (Normalized): F10: Sample, 9dpi	Abundances (Normalized): F11: Sample, 9dpi	Abundances (Normalized): F12: Sample, 9dpi
P62259	14-3-3 protein epsilo	Ywhae	2	1	100.00	100.00						5122.587265		8880.506601	11914.1612		16384.13616	
P68254	14-3-3 protein theta (	Ywhaq	2	1	100.00	100.00					43324.15077	92553.79094	57758.85227	69843.48329	42983.06957			75198.74855
Q9R0Y5	Adenylate kinase isoe	Ak1	1	1	1.83	1.34	61538.10938				91408.83327	241376.0816	59135.55954	138705.4827	24437.72362		119561.0485	82259.55301
P40124	Adenylyl cyclase-assc	Cap1	6	1	4.40	4.03	24228.74805				104280.2598	112408.7347	108820.8393	91073.63095	90724.81566	105320.2484	76023.55051	157378.6707
P07758	Alpha-1-antitrypsin 1	Serpina1a	3183	1	0.60	0.37	90170495.61	95018884.5	74918264.11	77287694.96	52324701.92	52061293.46	56459039.62	73967246.21	27690220.72	34948674.31	30284870.16	23004710.72
Q00896	Alpha-1-antitrypsin 1	Serpina1c	4643	1	0.18	0.17	1000186.039	616015.4385	814291.2185	772208.0481	112215.1015	110231.4684	163793.7167	210783.166	167600.8029	131954.3616	61043.91791	100026.4513
Q02357	Ankyrin-1 OS=Mus m	Ank1	5	1	100.00	100.00						4527.237262	8014.480306					66623.04716
Q8BFZ3	Beta-actin-like protei	Actbl2	111	1														
O35490	Betaine--homocystei	Bhmt	1	1	100.00	100.00					54836.27665	64377.8489	102476.0736	81278.63622	36294.63812			
P15327	Bisphosphoglycerate	Bpgm	1	1	1.60	2.71	142564.3594		58032.33099		173162.6928	182533.6199	161707.3848	116552.1089	156020.1751	210356.2957	209866.4191	389086.1671
O88200	C-type lectin domain	Clec11a	1	1	0.44	0.47	79889.19531	115373.7297	97486.79158	77993.55445	32657.7338	59318.14968	49074.5697	34375.39317				41511.67306
Q61508	Extracellular matrix p	Ecm1	8	1	3.57	1.71	9087.005859	16939.19574	7953.494023	16362.27828	40990.26752	31785.09958	58045.7188	58624.76692	17691.44963	24622.32725	32789.98989	5733.059115
O09164	Extracellular superox	Sod3	2	1	0.42	0.40		266276.7611		417515.5798	147787.0491	198130.4478	81997.33837	156023.5019	155279.8817	115055.7971	124239.1324	
P06745	Glucose-6-phosphate	Gpi	7	1	100.00	100.00					118300.4926	135653.6294	130699.4312	200329.316	75286.10956	105726.8465	52326.157	35044.70308
P14426	H-2 class I histocomp	H2-D1	26	1	100.00	100.00					387478.3968	316855.8151	271301.7669	307750.044	253253.7883	282804.727	244937.9851	331576.9987
P01902	H-2 class I histocomp	H2-K1	27	1	13.28	11.01	47239.35547	86915.04215	60221.10858	77940.04864	1237478.52	1010713.824	786714.0148	637636.0462	672198.7315	944094.024	592618.3243	768511.3412
P01897	H-2 class I histocomp	H2-L	46	1	15.60	13.47	60967.59766				1138208.353	1060481.478	784091.9789	932431.8321	834070.8919	1076498.677	686240.6431	808482.344
P14430	H-2 class I histocomp	H2-Q8	56	1	14.17	13.25	94766.45313	126242.1898	110025.6094	137110.2363	1643195.011	1594412.753	1654925.119	1747855.597	1567467.961	1926517.992	1099871.332	1577439.457
P07901	Heat shock protein H	Hsp90aa1	2	1	100.00	100.00						500532.5065	63702.03947			281597.3753		
P02104	Hemoglobin subunit	Hbb-y	137	1	2.87	4.80	44923.70313				134171.7501	123535.9996	240793.6576	54777.67659				215795.9582
P26928	Hepatocyte growth fa	Mst1	2	1	100.00	100.00							139493.5934	150643.5468	66223.86997			
Q64523	Histone H2A type 2-C	Hist2h2ac	1	1	2.37	1.50	36732.19141				73880.34878	285001.6698	65850.06927	102395.9892	62520.89971	58986.92038	29342.68964	51610.60542
P84228	Histone H3.2 OS=Mu	Hist1h3b	1	1	100.00	100.00						84665.89311						6858.702559
Q9ERH7	Homeodomain-intera	Hipk3	6	1	0.24	0.46	4951189.338	253601.3996	662246.2225	1009406.675	1077569.313	177361.4275	25656.76229	320447.2048	96922.06355			2704499.417
P01758	Ig heavy chain V regi	Igh-VJ558	4	1	1.45	7.85			49518.8017			71653.30297	102359.9804	41141.90089	371648.3763	381328.0031	905414.6461	396318.9471
P01803	Ig heavy chain V regi	1	7	1	1.06	1.60	302679.125	545522.1605	157003.4041	335353.0273	4141912.951	129305.9095	340901.9028	641128.1493	226129.2549	613455.297	251522.8839	495172.2051
P01759	Ig heavy chain V regi	4	4	1	2.79	13.84		17724.69098		33983.61126	79331.01242	78505.60884	66743.55145	58998.76864	266603.5884	347747.378	461919.2842	249777.8162
P01812	Ig heavy chain V regi	1	34	1														
P01797	Ig heavy chain V-III re	1	14	1	1.11	2.23	106369.9297	608768.0703	111283.7253	219960.6649	421394.8072	119564.2241	297002.5576	122202.7695	288531.8316	448039.0137	459875.1735	263625.7455
P01633	Ig kappa chain V19-1	Igk-V19-17	12	1	1.54	1.67	3649778.98	4029601.754	3707800.341	3190146.223	4299200.535	3563316.258	4550809.291	3527270.582	5059493.683	6937006.498	5636682.973	1602209.025
P03976	Ig kappa chain V-II re	1	5	1	1.23	2.08		233102.6889	101013.4532	131728.1754	285817.0123	221744.2643	103387.2721		310133.1456	261493.3092	332241.3613	219924.3268
P01630	Ig kappa chain V-II re	1	19	1	1.80	2.98	621811	1207384.427	867895.5128	817883.793	1383118.103	1581299.352	2309682.634	1108827.829	2966399.683	3589539.825	2364773.006	1853595.136
P01661	Ig kappa chain V-III re	1	36	1		100.00									107889.3933	107221.1011	127492.5575	54634.98813
P01674	Ig kappa chain V-III re	1	16	1	3.24	15.53	86952.6875	428982.6618	595849.1199	425170.5169	1061990.773	1279952.117	1496803.796	1593443.831	4467566.552	7933967.747	7768159.757	4935602.979
P01660	Ig kappa chain V-III re	1	124	1	15.24	100.00	158153.0352	213665.5879	194961.7143	214380.1675	5115320.196	3385087.258	2612644.691	2593001.17	16865700.59	22553110.61	35654436.11	23295647.63
P01663	Ig kappa chain V-III re	1	65	1	5.29	35.86	112289.2988	88448.93087	193447.3826	138568.5934	641790.732	728651.0061	598172.8049	728313.2686	4076473.299	4907006.883	6479234.624	3405943.678
P01670	Ig kappa chain V-III re	1	100	1	4.73	10.34	656651.1172	1660808.003	702966.028	1126313.709	7673961.924	4573931.778	6168318.785	6115622.882	20965416	21220616.62	23396583.65	16235070.78
P01680	Ig kappa chain V-IV re	4	5	1	1.16	2.48		406931.8475					577739.4869	387529.6569	1009685.5	1135859.331	851885.5546	
P01642	Ig kappa chain V-V re	Gm10881	11	1	2.14	5.08	184020.5625	565654.5147	332205.3137	431133.7068	912149.3703	509153.4509	953106.3823	715777.5796	2264309.217	1889387.028	2118185.044	1478297.596
P01636	Ig kappa chain V-V re	1	2	1	2.13	5.19		55462.18018				110698.9405		126502.0716	239558.5062	305170.4338	344550.4155	271674.1164
P01679	Ig kappa chain V-VI re	1	7	1	0.29	0.63	766983.125	648822.5978	668683.7783	188670.8756	144519.3327	127966.4234	240924.7832	193890.2548	302755.2446	448928.1528	449076.6348	383027.6077
P04940	Ig kappa chain V-VI re	2	2	1	0.60	1.57	172077.2344	221688.5231	323372.1758	229280.5475	164058.2866	135460.2212	92654.796	141952.9991	379275.0888	331576.0965	429770.1739	305960.9578
P04945	Ig kappa chain V-VI re	2	11	1	2.80	7.16	71366.11719	117469.1036	109878.3756	137246.5975	354398.9059	332238.4292	289117.0013	305145.6143	586156.7148	835934.7911	988179.5516	637902.4627
P01845	Ig lambda-3 chain C r	Iglc3	14	1	3.21	3.17	45403.125	184067.3571	188462.6861	224385.0651	668291.1908	651083.6266	254836.0253	352769.8473	546469.1266	688022.9206	472566.8638	619047.7213
P47878	Insulin-like growth fa	Igfbp3	3	1	0.60	0.34	122139.7578	136500.8895	116673.6307	127936.0572				75241.29898	49076.61673		37441.49027	
O35664	Interferon alpha/beta	Ifnar2	3	1	0.76	0.42	73391.70313	17996.55069	41964.74228	49978.84505	31950.72369	37856.32666	13730.22295	86545.10944	43375.80321	17435.0612	17840.22563	9174.687641
Q9Z0M9	Interleukin-18-bindin	Il18bp	2	1														
Q02257	Junction plakoglobin	Jup	3	1	0.01	0.01	3223.902588	36012.14263										
P08730	Keratin, type I cytosk	Krt13	24	1	0.01	1.12	210601.6328	1132565.531	200435.3649	42951.25072					101557.7205		601557.4872	
Q6IFX2	Keratin, type I cytosk	Krt42	21	1	0.05	0.05			612976.3146		28872.63573						44061.41307	23596.25986
Q3UV17	Keratin, type II cytosk	Krt76	6	1	0.26	0.12	663732.3125	17694965.48	3979183.643	1336178.258	1032915	176386.9211	911217.7768	1107416.264	456848.6682	529634.6583	1314172.653	19960.30339
Q922U2	Keratin, type II cytosk	Krt5	63	1	0.21	0.34	342979.8135	9382878.238	1304739.495	791879.1854	737373.3318	174020.3687	651114.2233	241984.1539	363587.5704	352192.038	1429842.749	124095.6413
Q6IME9	Keratin, type II cytosk	Krt72	2	1	0.01	0.78		306789.7108	80129.16925								122815.5081	
Q6NXH9	Keratin, type II cytosk	Krt73	10	1	0.24	0.33	74250.92188	942557.5327	147273.8732	158315.3705	32861.60782	25866.46359	39722.69286	47784.84911	49265.6644	42507.98493	101723.1403	50009.64113
Q8VED5	Keratin, type II cytosk	Krt79	44	1	0.21	0.54	326172.4688	1862036.553	1964129.239	471627.9854	422402.1558	64183.07758	213410.6985	230895.9633	291068.9143		1933509.919	151506.943
Q3V2T4	Keratinocyte differen	Krtdap	4	1	0.12	0.11	165842.3125	126199.325	140164.7364	145511.8461	17112.12066	22475.26351		13698.6369	16100.0966	15220.54061	26243.6706	8743.587986
Q8CIM1	Leucine-rich repeat-c	Lrrc45	23	1	0.95	1.30	63033.80469	52351.1853	49695.11619	44915.04836	58936.08846	60856.30596	35390.50189	46025.27568	67816.94843	59846.1819	85787.53626	58637.43101
Q8VCC2	Liver carboxylesteras	Ces1	84	1	1.25	1.21	1030488.875	1006670.8	1076828.893	1144522.919	1366468.724	1403250.821	1221523.518	1319163.443	1009909.91	1600862.808	1718719.503	1050571.496
P16125	L-lactate dehydrogen	Ldhb	8	1	0.76	0.64	10846322.95	12666409.07	12088508.36	12894510.13	10767591.77	6645714.702	7809932.212	10303658.08	8418114.711	9151279.477	6825454.723	1736848.77
P18337	L-selectin OS=Mus m	Sell	2	1	6.93	1.95	7819.941895			11778.05901	74646.16347	23547.56571	59332.82905	92861.25072	15364.36238	22889.55565		
P26645	Myristoylated alanine	Marcks	1	1	100.00	100.00					82224.27025	87714.47443	66638.83386	68416.36929	38074.3779	60121.5823	48173.33655	53071.72135
P17742	Peptidyl-prolyl cis-tra	Ppia	1	1	1.21	1.06			139649.1083		139510.1735	221027.4227	171287.2555	166770.3611	111274.2018	147123.056	149914.9407	176166.1276
P97298	Pigment epithelium-d	Serpinf1	12	1	0.81	0.41	168499.0313	183563.6292	180697.5758	188896.5524	163123.1194	126920.5846	85712.56664	200999.9047	72562.88608	86700.55546	71924.80415	
O08742	Platelet glycoprotein	Gp5	1	1	0.11	0.35	259152.9688		165596.9277	88310.08892	17629.96599					44382.53514	42291.39391	91537.06407
P62962	Profilin-1 OS=Mus mu	Pfn1	1	1														
Q9QUM9	Proteasome subunit a	Psma6	4	1	4.43	4.49	69415.15625	96894.59151	70898.99935	66764.42079	387746.321	400985.6366	310426.6533	258522.6273	316621.6417	403261.5606	297252.2031	319931.2136
O35955	Proteasome subunit	Psmb10	5	1	6.10	11.15		25606.89813			166035.0256	221803.1578	146795.6149	133664.3137	287515.0614	283429.9459	264747.7764	369865.2791
P28076	Proteasome subunit	Psmb9	10	1	2.53	1.80	7433.475586		8820.278575		6815.728682	8173.111178	51158.5909	77016.87049	71416.15946	16450.67151	10117.12519	12942.00771
Q5I2A0	Serine protease inhib	Serpina3g	399	1	3.51	3.13	5608.762207	4253.960353	4763.338957	6127.932588	4366.18232	18146.09683		33213.54403	17881.24449	15848.72398	11123.03846	18780.38694
Q9ES07	Solute carrier family	Slc15a2	1	1	0.74	0.14		87411.98767	339692.7561	318507.3011	166982.2922	114857.5971	120392.7017	352285.3628	43692.7236			
P08228	Superoxide dismutas	Sod1	4	1	3.11	0.75	87121.10938		44938.6885	37410.4406	167386.6166	77954.85999	153203.5664	247818.8963	33757.66655	40055.87626	33722.00392	
Q80YX1	Tenascin OS=Mus mu	Tnc	1	1														
P43025	Tetranectin OS=Mus	Clec3b	2	1														
P10639	Thioredoxin OS=Mus	Txn	1	1	2.73	1.36	87782.42188		59239.87389		158180.1243	244611.134	232292.177	194100.3302	58979.57509	76907.62024	125509.7906	138426.1368
Q62351	Transferrin receptor	Tfrc	1	1	0.22	0.21		135864.6939		125914.3506		35255.76126		23661.81789	26782.97604			
Q06318	Uteroglobin OS=Mus	Scgb1a1	4	1	3.71	0.70			120964.2753		311384.6901	383379.5796	526031.0405	1028908.408	84380.65838		84247.94234	160652.5297
P35917	Vascular endothelial	Flt4	6	1	0.30	0.01	40536.43066	40294.88454	34608.27744	30552.99141	15211.2152			7107.288674				
P33587	Vitamin K-dependent	Proc	2	1	2.71	0.35		55049.78526		40511.25066	109005.5957		110266.6356	184499.1967		36630.44736	7606.202312	
P68134	Actin, alpha skeletal r	Acta1	191	2	68.80	47.49	28914.98633	122722.0727		176143.8327	5297135.001	10451491.72	7845191.507	9086485.031	5302339.029	6405241.767	3971733.191	6899359.438
P00687	Alpha-amylase 1 OS=	Amy1	5	2	1.52	1.38	223758.1875	234806.6154	249272.1787	216451.8991	269187.2413	164302.9085	186708.9001	647323.7129	909345.2978	367055.9828	232617.3541	264622.2183
P34928	Apolipoprotein C-I OS	Apoc1	37	2	0.77	0.59	12239038.91	7716930.842	6790520.729	6135500.218	4248003.58	5820389.621	4764523.877	3411429.61	2928313.274	4917073.361	3717058.533	3128373.509
P21550	Beta-enolase OS=Mu	Eno3	6	2	100.00	100.00					136806.369	298474.4621	123229.9178	244397.7777	17292.23396		194001.4562	12784.56468
P07743	BPI fold-containing fa	Bpifa2	14	2	100.00	100.00					166812.1535	99613.54819	120210.8532	1026191.932	1417953.747	75218.74158	51931.51535	24376.91539
P16015	Carbonic anhydrase 3	Ca3	7	2	2.26	1.05	39420.9375		173219.766	65914.39821	48934.08735	509659.0824	200168.3958	416961.9612	39192.78714	32416.72211	273661.9714	121193.885
Q9WVJ3	Carboxypeptidase Q (	Cpq	3	2	1.24	0.93	322274.6094	348336.9811	389786.0565	457515.2603	511916.404	493032.0344	421376.8788	409165.5172	366269.8934	403885.388	340709.928	317646.5859
P24270	Catalase OS=Mus mu	Cat	7	2	2.36	2.59	53986.84961				112335.8806	179006.3557	173768.7347	115111.0365	119596.6012	125739.1149		317220.7513
Q9WUU7	Cathepsin Z OS=Mus	Ctsz	7	2	2.68	2.86	89061.46289	101380.5226	119067.2274	116050.6663	342211.6719	339175.7367	358009.1703	355877.1709	387116.7011	337180.0323	256370.8667	275100.2805
Q8R1U2	Cell growth regulator	Cgref1	6	2	100.00							12312.30867	10529.91061	12413.15696				
P14106	Complement C1q sub	C1qb	15	2	0.39	0.47	1781894.25	2406946.551	1941421.333	1326937.541	658026.631	717119.1516	576667.0327	403228.7709	635766.9312	757924.4146	1368074.628	742272.9196
Q02105	Complement C1q sub	C1qc	20	2	0.40	0.58	865870.9531	1769655.727	1134087.218	751455.7641	587611.7581	571023.5786	308858.5732	545146.2907	508976.4847	438644.1941	815821.0532	434415.6825

O89053	Coronin-1A OS=Mus	Coro1a	3	2	5.69	3.20	11187.86426	13320.11075		12347.50763	69620.07419	76510.19048	140291.977	208021.7508	49850.86293	46663.9758	32714.46033	130621.3742
Q8BPB5	EGF-containing fibuli	Efemp1	6	2	2.32	0.01	91418.25781	41716.063	108257.0566	71061.57235	89968.06601	109864.8433	177060.5281	105834.0566				
Q923D2	Flavin reductase (NAI	Blvrb	14	2	1.60	0.90	139829.5469			99620.5116	449534.8497	465290.2961	565964.6737	340272.8051	48677.63501	241328.0262	437735.355	781729.2995
Q8K0D2	Hyaluronan-binding p	Habp2	4	2	0.70	0.61	491959.9063	515428.9897	348827.0394	386679.3716	259051.8583	290370.6696	231488.4126	341914.29	261783.9665	120163.3283	289405.5159	216808.7196
P01750	Ig heavy chain V regi	1	13	2	1.72	5.08	70056.10156	212764.5174	62410.34457	96791.58847	544969.3747	373920.4629	456560.5282	444398.3249	1876033.044	2164958.318	2777039.786	1257194.347
P18531	Ig heavy chain V regi	Ighv3-6	6	2	3.08	6.36	61606.46875	68558.18446	102715.9465	35139.33958	359184.7387	268699.928	239270.4495	253078.5848	515529.2951	864173.0022	801745.1975	176987.9315
P01786	Ig heavy chain V regi	1	42	2	1.28	2.99	1108734.344	1286093.377	741891.0805	1031388.828	1145333.945	1365526.504	1329815.773	1458980.322	3008616.75	4173421.292	3895007.478	2378297.557
P01631	Ig kappa chain V-II re	1	46	2	1.93	2.38	2387050.078	4234506.679	2911411.527	3416888.436	10195139.1	5287359.216	5669334.382	5631545.673	7564231.44	10191326.68	11247204.68	6469274.932
P01655	Ig kappa chain V-III re	1	116	2	2.79	8.79	69065.4375	1111635.946	326225.3578	298017.5269	744557.7384	886285.4458	823708.3983	1161424.213	2622627.001	2835101.523	4855460.269	2610959.693
P01646	Ig kappa chain V-V re	1	38	2	1.99	7.81	117367.8125	419080.0818	335596.1891	284693.9324	702755.2768	635116.8603	470840.9625	723504.9578	2174117.673	1766178.485	4341112.356	2701175.058
P01635	Ig kappa chain V-V re	1	16	2	1.52	6.18	144233.9531	762571.4419	635824.8643	614551.7403	1252830.217	962327.2667	1050772.349	716015.5716	4489066.212	4228345.578	6731725.489	3686416.921
P01639	Ig kappa chain V-V re	Gm5571	10	2	1.27	3.28	159297.1563	324315.4976	270451.9586	511239.0422	582606.7033	630358.248	388700.5615	237931.8067	1151934.977	1360037.252	1196039.243	782352.0353
P01723	Ig lambda-1 chain V r	1	27	2	2.39	4.92	190220.7305	502475.0675	300297.609	349819.4923	1078857.397	664756.2501	566080.793	796126.7105	1413855.597	1720122.201	1648369.565	963714.9775
Q61781	Keratin, type I cytosk	Krt14	58	2	0.31	0.16		943918.2542	2864906.205	118929.6667	130916.0554		364513.8367	37041.31738	178268.832	86408.74517	194155.7328	
P04104	Keratin, type II cytosk	Krt1	25	2	0.28	0.41	1835027.711	40912852.97	9183888.807	3874849.564	2532498.69	831180.0635	2237273.388	2206517.741	1895049.911	2203288.973	9721070.463	1381154.936
Q91WP0	Mannan-binding lecti	Masp2	10	2	0.68	0.35	109191.0391	125365.7638	54097.06115	62131.67273	89304.75569	70304.26318	40114.08434	41893.28481	23236.45542	23542.51484		
P26041	Moesin OS=Mus mus	Msn	4	2	9.47	5.99	7080.12793				183497.4692	197201.31	169649.3616	245841.78	183582.7871	211266.393	77004.16052	129767.6627
P28666	Murinoglobulin-2 OS	Mug2	1894	2	4.11	1.31	25016.37305	93311.92923	32597.98091	80515.03845	2734808.819	131403.5481	336707.4786	72558.89537	82102.1666	44569.00979	109500.1562	71473.55086
P55065	Phospholipid transfe	Pltp	14	2	1.10	1.21	612574.0938	799702.3896	708926.1146	832376.8369	783303.4253	1038905.668	814130.2777	515030.0315	907376.6566	890306.1882	917721.3552	679290.3311
Q9Z126	Platelet factor 4 OS=	Pf4	28	2	0.08	0.34	8001302	162195.4263	4852993.585	3289473.974	427608.7697	135532.02	607058.4155	198644.4565	331761.0662	1981100.135	1298822.497	1505407.434
Q9Z2U0	Proteasome subunit	Psma7	6	2	3.59	5.00			44042.77237		223268.1059	279114.0076	181365.7691	160570.0915	329553.0008	385103.4836	230159.7733	401415.3248
Q9JM99	Proteoglycan 4 OS=M	Prg4	4	2	6.06	4.06	47240.46094	40433.42389		51055.63733	348616.8838	491933.8753	464506.8802	314439.2146	252846.7761	257309.4763	176117.5126	263018.9984
Q8K1I3	Secreted phosphopro	Spp2	14	2	0.72	0.94	1104352.313	1134329.755	1106192.056	986252.1718	815007.684	771605.8461	917366.4942	570448.1113	1094713.32	1060276.648	773854.8901	1051606.374
Q9Z1T2	Thrombospondin-4 C	Thbs4	6	2	0.77	0.49	160462.1016	172820.7057	200822.6342	219563.4368	82662.91518	145892.0162	87682.506	108228.9511		62932.64338		58182.38592
P61939	Thyroxine-binding gl	Serpina7	2	2	1.62	1.00	85316.88281	119878.7461		150728.1545	120125.0231			279098.7246	92825.81716	114663.3292	158077.8922	103768.925
P40142	Transketolase OS=Mu	Tkt	2	2	6.15	2.39	9757.011719		5750.20456		60491.64752	68596.83	80272.81209	73106.72774	24142.4675	22542.92361	13324.33722	16395.41729
P63101	14-3-3 protein zeta/d	Ywhaz	17	3	9.03	6.07	89707.27734	29184.61828	59757.36119	86360.60144	714148.2374	984992.7918	692169.0584	1027587.931	570418.4522	669153.1563	406365.3804	748779.8826
P07361	Alpha-1-acid glycopro	Orm2	1643	3	100.00	100.00	60549.91406	83216.72748	38766.44844		8911610.2	13787649.14	12388114.45	19945088.71	8074871.958	4682306.633	6968504.937	9011965.289
Q6GQT1	Alpha-2-macroglobul	A2m	46	3	0.51	1.06	183436.6152	316661.9107	293525.4181	175913.2081	78644.75153	77648.81573	111195.8508	528767.8361	233338.7489	418586.4339		33253.71635
Q8C7G5	Apolipoprotein A-V C	Apoa5	4	3	100.00	100.00					265332.8149	480675.7577	490512.9687	274453.5147	338531.7242	317760.4517		190101.37
Q05020	Apolipoprotein C-II O	Apoc2	112	3	0.11	0.22	6187958.57	4397269.875	4754837.359	3922689.367	465058.4893	277970.9514	747921.9705	621814.3402	1448807.536	679774.9842	1506761.542	663727.8759
Q9Z1R3	Apolipoprotein M OS	Apom	51	3	0.64	0.53	4536157.344	4391665.915	4077401.976	4064440.898	2967196.676	3413019.406	3978972.348	1671581.112	1919624.717	2523522.869	2616460.016	1317937.366
P04919	Band 3 anion transpo	Slc4a1	7	3	0.84	0.69	55131.00391		10765.57336		41654.39321	56315.80633	69977.55083	34718.62278	4423.119392	6667.899494	7406.085105	508785.955
P01887	Beta-2-microglobulin	B2m	66	3	0.77	1.09	14175102.22	16131725.51	15932574.85	15703038.05	12030609.77	11049458.15	8021765.852	14115880.78	14908827.64	17002057.15	15737187.28	16719908.88
P62897	Cytochrome c, somat	Cycs	17	3	5.00	4.02	93346.58594	61869.00938	33454.00366	179835.8834	668741.6044	1069162.434	607845.7761	948521.4212	573751.7671	745787.4487	371135.5787	471656.4912
P11404	Fatty acid-binding pr	Fabp3	16	3	0.66	1.07	6924962.5	8371436.919	10164375.23	8011373.839	6120327.421	4185691.134	2081752.719	5275843.971	8346725.924	9064737.644	8780334.062	
Q08879	Fibulin-1 OS=Mus mu	Fbln1	4	3	0.45	0.28	278014.1797	501984.772	637710.6319	560657.7575	99764.906	162252.2991	160013.5895	150924.7622	32168.61506	61240.98532	174931.3969	49032.73788
O70165	Ficolin-1 OS=Mus mu	Fcn1	6	3	0.59	0.53	203049.7422	338328.6826	203524.7975	706166.8886	65357.29024	134199.77	49456.76672	82448.0609	553540.0254	110229.0401	49832.10037	50726.55796
P62806	Histone H4 OS=Mus r	Hist1h4a	3	3	100.00	100.00					185520.4141	974458.1931	232614.6818	92970.08265	136282.9009	109307.2996		230130.2488
P06330	Ig heavy chain V regi	1	44	3	4.08	14.59	492435.0469	2274944.573	1855649.585	1162365.346	6630958.605	5115876.925	5498917.772	4848249.981	17533502.66	22357097.54	23981039.22	14677400.84
P01807	Ig heavy chain V regi	1	75	3	0.64	1.29	4660638.031	5476209.394	4835929.873	3667443.524	3425654.914	2648429.868	2839059.988	3118618.253	5409873.687	7153916.431	9133969.212	5547952.853
P01665	Ig kappa chain V-III re	1	122	3	4.91	24.12	87931.72656	108381.1096	75230.28069	123196.4419	808210.8371	727203.7142	974603.8644	1188658.263	4619731.583	5553265.034	7562911.085	4545990.901
P01638	Ig kappa chain V-V re	4	14	3	1.84	4.43	254720.3555	469389.375	231270.4331	367578.688	800469.7193	519744.9677	664190.0396	886358.3028	1255318.027	1864570.245	1964410.138	1051661.342
P01843	Ig lambda-1 chain C r	1	44	3	1.43	2.41	2641764.457	2296366.923	2513728.08	3287819.776	3582979.068	3233118.923	5042137.524	3001068.947	5149411.241	5874188.738	5420925.817	2629445.656
P01592	Immunoglobulin J ch	Jchain	43	3	1.79	1.53	1273887.25	1749567.274	1868918.049	1873129.196	2964244.322	2249762.322	2929969.123	2829782.32	2086171.47	2759276.119	2771770.598	1653974.809
P02535	Keratin, type I cytosk	Krt10	78	3	0.35	0.64	2059063.367	7901874.823	9119239.044	3311897.336	2639472.25	611792.1741	1519338.061	2045416.925	1872931.696	2237792.156	11115742.98	1517655.834
Q9Z2K1	Keratin, type I cytosk	Krt16	53	3	0.23	0.43	643709.1035	8377691.41	14609326.18	1597057.499	770702.0612	157300.6844	628800.0977	394329.0764	735385.6555	746897.3922	8749981.48	263307.7958
P50446	Keratin, type II cytosk	Krt6a	33	3	0.28	0.54	1065305.35	12064817.31	6909685.974	1269602.751	842517.2161	304435.331	628748.5993	789598.8172	815156.0733	773867.9414	1511994.277	595357.0509
B5X0G2	Major urinary protei	Mup17	145	3	0.09	0.02	9528259.625	5958032.335	5108957.445	5662480.859	281544.1002	290773.1999	304680.0173	370902.8873	224169.1998	16091.42673	41917.06194	365927.2345
P14152	Malate dehydrogena	Mdh1	14	3	5.75	2.84	121442.1094	35228.46005	46305.75227	249632.6656	1163816.751	1176971.619	922785.9236	1374457.566	236840.5292	591236.7092	610501.8505	732837.3343
P39039	Mannose-binding pro	Mbl1	12	3	0.51	0.40	1723044.344	2003053.908	1939731.86	2118201.017	1099525.873	1219844.184	1072683.951	741580.0123	743212.3181	651092.7245	837214.484	518241.4282
P10404	MLV-related proviral	1	10	3	4.21	1.25	647727.1172	280701.239	513750.1778	524001.494	1101348.005	1623772.485	1158896.44	1605367.064	620831.1941	611596.3462	465596.2044	753249.4967
P11672	Neutrophil gelatinase	Lcn2	20	3	8.84	2.61	48260.21094	66444.9649			2026640.383	1877508.829	1672083.733	4338117.213	345244.9754	550345.9346	122508.3894	314126.3051
Q01768	Nucleoside diphosph	Nme2	17	3	6.62	5.41	103000.5313		58665.72514		832468.0397	1100629.792	1055636.793	776237.2086	585957.9828	617780.5347	495484.9992	905861.688
Q61233	Plastin-2 OS=Mus mu	Lcp1	16	3	100.00	100.00					414381.0111	474243.1633	407783.3255	443446.6246	279098.7698	324333.2667	241443.4477	293403.7022
P52480	Pyruvate kinase PKM	Pkm	11	3	5.05	2.58	158653.4453	138055.3401	178236.7861	223558.252	724057.1296	993050.3752	1133647.739	948747.6502	498361.8105	481098.1271	479343.3842	608377.1745
P04918	Serum amyloid A-3 p	Saa3	34	3	25.00	9.97		27319.36184		18770.89649	946497.1051	1259470.595	1400708.959	1400606.891	367147.4572	526766.3995	91945.08774	803575.724
P17751	Triosephosphate isor	Tpi1	4	3	2.44	1.02	91518.63281			68768.42437	3410854.272	2818402.745	203982.6556	383340.3709	51887.77819	56053.12934	188949.3131	116770.1433
Q00897	Alpha-1-antitrypsin 1	Serpina1d	3690	4	2.65	1.77	10207073.09	9616661.117	8905330.241	9103212.671	16669162.01	21524945.84	28621688.47	17722853.12	13664256.72	13955375.75	13144566.87	14191346.83
P33622	Apolipoprotein C-III C	Apoc3	461	4	0.34	0.32	69007220.78	62267417.91	63589707.18	62041842.01	22108968.48	28292645.31	21066167.09	17980008.18	20060117.71	22096774.14	18806205.44	22601369.77
Q61268	Apolipoprotein C-IV C	Apoc4	44	4	0.38	0.38	7024802.828	6322107.557	6068588.678	5498890.63	2304164.675	2887101.793	2262026.803	1298202.575	2885628.132	3546477.978	1764718.199	1933430.393
P10605	Cathepsin B OS=Mus	Ctsb	22	4	2.15	1.52	572504.9458	529345.455	526319.654	554953.386	951441.0583	1282159.181	742817.7526	1151718.591	776306.8314	928869.7039	874741.9513	760581.1668
O88783	Coagulation factor V	F5	13	4	0.70	1.07	446670.6406	533376.2734	471618.5554	393080.5421	282625.705	436809.8996	288649.3539	53988.38149	615430.8364	546270.1686	433290.3965	398362.2275
P03953	Complement factor D	Cfd	79	4	0.78	0.47	10094164.28	8782451.416	7989332.33	8854966.394	6766105.847	7610434.475	5774960.479	7738978.19	3756285.586	4473963.065	4427445.102	3099184.196
E9PV24	Fibrinogen alpha cha	Fga	14	4	1.17	0.65	92363.89844	1183886.974	234263.2296	202589.7084	130790.3767	592352.5447	440282.2706	503863.2981	72241.75741	251275.9578	73146.13348	86841.90766
Q8K0E8	Fibrinogen beta chair	Fgb	4	4	0.66	1.20		1799308.096	31155.06087	152260.6825	92555.09133	455581.9853	395879.705	62546.6792	35394.47712	255885.7428		
Q3TTY5	Keratin, type II cytosk	Krt2	21	4	0.30	0.41	415814.5977	8170305.046	2965457.927	1152727.172	550441.3646	106512.6249	389389.1665	608116.9581	320937.1423	684894.7238	5075835.827	285369.1561
P11589	Major urinary protei	Mup2	155	4	0.13	0.03	39091180.98	48831844.96	30492485.2	37978027.38	2902353.031	5127552.916	4754475.322	5175111.003	1630845.343	309927.806	529322.4825	729782.5046
O35930	Platelet glycoprotein	Gp1ba	13	4	0.38	0.41	569814.5938	492621.9501	888133.0036	746423.0573	169844.0566	205529.4592	265369.7593	120236.6968	266809.456	397389.5948	208602.3409	269809.3847
P09813	Apolipoprotein A-II O	Apoa2	303	5	0.41	0.36	136380135.1	138133107.3	137013911.6	123769081.2	61162544.7	45683890.2	53089994.28	50783406.2	42819088.22	47918993.84	33035564.26	11979373.57
P98086	Complement C1q sub	C1qa	18	5	0.35	0.37	2489190.781	2747666.54	1961119.606	1442122.257	445998.1781	710998.8555	459239.8597	544128.9991	684794.9305	783093.9971	1337653.826	622172.8868
P05064	Fructose-bisphospha	Aldoa	40	5	5.28	2.83	449698.75	28566.75041	199182.31	163885.5928	1426449.01	3269672.518	1480461.086	2214550.786	1593725.567	1308863.781	2183324.724	1046684.002
P01878	Ig alpha chain C regio	1	118	5	4.00	2.93	1189772.455	1636926.53	832152.6295	1131237.028	4486316.24	6340250.388	5816274.685	5846426.437	4577916.881	3350469.365	4021410.374	3737794.589
Q61805	Lipopolysaccharide-b	Lbp	6	5	5.83	4.41	115396.2773	189682.5781	217978.7423	186022.6965	1310699.004	1827334.234	1553529.231	1496968.949	1200352.892	1575176.782	932451.7762	834789.4579
P09581	Macrophage colony-s	Csf1r	28	5	2.06	1.47	366170.8535	558479.9574	485440.2103	671948.6899	1111308.001	1077386.899	1355314.645	1045459.768	788691.7358	773344.1777	1015248.5	662058.4137
P41317	Mannose-binding pro	Mbl2	31	5	0.32	0.23	3396889.313	3008603.39	3272666.469	3233603.325	1630503.377	1463889.211	1015903.636	1549633.672	553984.9969	1257868.627	765352.3271	376309.2823
Q61171	Peroxiredoxin-2 OS=	Prdx2	16	5	2.80	2.61	1625839.75	344126.8064	379356.2914	178888.0975	1548600.421	1995810.551	2916172.417	1512117.684	534778.9711	1535421.924	1551624.013	3338460.625
Q61207	Prosaposin OS=Mus r	Psap	12	5	1.52	1.27	309454.7578	295045.9211	283969.9731	292205.8454	465883.8101	590671.7222	252054.3736	426016.8251	398465.8871	524562.1748	420168.5052	358756.0737
Q9CQW3	Vitamin K-dependent	Proz	26	5	0.16	0.12	1187354.063	2071159.334	1640127.863	1416514.192	142729.4518	140818.1886	372843.9432	61282.91897	77435.8235	12180.43041	147744.5768	176171.2909
Q8CG14	Complement C1s-A s	C1sa	25	6	0.74	0.29	39186718.23	53129447.85	39353382.47	64848639.85	81767510.99	74803850.98	56249598.17	92208668.03	40831838.1	49902627.93	44079868.54	11576120.52
P14847	C-reactive protein OS	Crp	23	6	1.89	0.92	872635.3555	688475.5787	764325.47	777855.6819	2157325.383	1915057.02	2243607.836	2317540.847	1081300.527	1650369.795	2513336.053	1170270.862
P46412	Glutathione peroxida	Gpx3	98	6	1.37	1.32	5958154.938	6844888.774	5063439.915	4755854.468	6247880.352	9880821.907	7879237.211	7799525.122	7033941.336	8255365.361	6946010.505	9041018.173
P16858	Glyceraldehyde-3-ph	Gapdh	41	6	3.60	2.20	17043325.1	4373595.497	12609305.36	5299573.431	20728248.65	22003709.05	21032730.82	14173832.26	11723827.23	13569854.4	23703579.55	36656993.15
P01865	Ig gamma-2A chain C	Igh-1a	397	6	8.69	37.93	1404911.42	8647094.6	2938535.363	5683340.102	53744011.7	44239968.88	44452408.84	48289428.62	205967118	254707975	265394970.8	173740165.5
Q61730	Interleukin-1 recepto	Il1rap	47	6	0.51	0.40	4327583.641	5581318.319	5102577.787	6127819.544	1709632.862	2150833.357	1428192.404	1209959.244	1949182.047	1600361.533	2609976.823	1992239.947
P51885	Lumican OS=Mus mu	Lum	39	6	0.59	0.39	2443908.414	2840827.914	2301784.918	2575842.808	1919369.746	1811774.073	1340089.601	1793250.093	1078515.2	869832.7628	915743.4577	1484960.845
P98064	Mannan-binding lecti	Masp1	32	6	1.06	0.85	1079210.921	1434616.981	1302814.156	1454450.199	1201846.089	1492745.346	1572479.25	1091645.807	1237183.923	1186479.064	813990.5874	912435.6829
P51910	Apolipoprotein D OS=	Apod	106	7	2.01	3.17	3152205.648	4338537.395	3559049.8	4532264.026	8483419.248	8018152.955	7043008.204	7050386.874	12646765.73	12877562.95	14629406.74	13125902.24
Q8VCG4	Complement compor	C8g	110	7	0.50	0.40	12056281.56	13162280.89	11792629.08	13252270.87	5796163.969	7656782.477	6648557.813	5507105.799	4821046.524	5106831.791	5141445.889	4988087.424
P02088	Hemoglobin subunit	Hbb-b1	1227	7	2.56	2.31	441999712.3	85806164.04	221895777.7	95269795.71	371196718.6	382707023.9	451615330.2	258327834.6	229035755.2	250430123.7	407986314.9	777976174.3
P02089	Hemoglobin subunit	Hbb-b2	868	7	2.87	2.97	529533016.6	87247385.43	233573541.8	96704902.68	453391089.3	539344494.9	664324198.5	346585125.4	269199201.1	292481136.4	532012279.2	1038519721
P06151	L-lactate dehydrogen	Ldha	34	7	8.60	6.13	290441.3496		85029.3463	162122.4594	2731306.273	4695694.128	2899935.926	4242646.235	3120848.964	3508345.408	2215372.535	3837250.658
Q00724	Retinol-binding prote	Rbp4	95	7	0.39	0.37	13466281.38	8141648.122	9352704.821	8334312.373	4179059.529	2798393.734	3447007.7	4961908.095	3376631.436	3136204.037	3631199.397	3563688.612
P70274	Selenoprotein P OS=	Selenop	118	7	1.14	0.67	2145245.015	1994326.782	2007767.546	2599180.262	1812349.785	2447388.395	2387556.769	2696066.542	1487152.527	1723268.181	1884163.984	1187951.849
P05367	Serum amyloid A-2 p	Saa2	227	7	15.61	3.69	77788.83691	89549.60451	93641.02032		65588429.88	66553402.84	83127140.01	72455499.52	38662629.42	28525856.03	8634833.556	59004799.18

P52430	Serum paraoxonase/	Pon1	147	7	0.44	0.54	12479986.63	14002017.49	12247748.92	14029975.92	5472107.55	6358020.109	5309061.97	4373096.234	5660594.581	7444924.184	10754514.93	5179045.602
P70663	SPARC-like protein 1	Sparcl1	109	7	2.67	2.74	230529.2991	302991.5115	278109.9773	360638.6203	564280.1975	937725.0369	517812.7376	1188899.16	944258.1231	1367545.282	664255.9573	475236.9757
P60710	Actin, cytoplasmic 1 (	Actb	317	8	12.31	8.37	6435612.229	3615196.92	3572337.836	5420398.016	61434086.02	82901806.74	58398338.79	74806000.38	49271819.35	56765371.6	34688825.88	70434293.96
Q60590	Alpha-1-acid glycopro	Orm1	2328	8	6.07	5.19	25783710.93	21632350.95	18406632.08	22188984.55	115813228.5	129577156.4	100827239.6	141474284.6	122464255.1	101224327.1	128407926.3	129733576
Q9JHH6	Carboxypeptidase B2	Cpb2	87	8	1.96	2.12	1325579.313	1694666.397	887024.8101	1625228.48	2339952.662	3140177.682	3081323.231	3224164.344	3603896.65	3038529.664	3059476.574	2366533.191
Q80YC5	Coagulation factor XI	F12	113	8	1.18	1.32	5556845.061	3794732.16	4414320.998	4264332.638	3983618.509	5341234.757	5362496.75	4520672.951	5498608.581	5816182.791	5589318.471	6652844.521
Q07968	Coagulation factor XI	F13b	20	8	0.37	0.37	1926868.625	2223896.881	2221399.583	2209058.813	673008.7911	723187.7051	792648.8042	746875.8369	794318.1529	834883.1371	917103.9381	479783.2221
P07310	Creatine kinase M-ty	Ckm	30	8	7.13	2.28	396092.2578		31377.86912	201564.4448	1574551.158	6356282.587	2053791.894	4216903.775	1574041.107	1636446.285	3972671.749	1830487.373
Q8VCM7	Fibrinogen gamma ch	Fgg	12	8	1.04	0.36	1088224.875	5626138.733	1235054.967	2287993.579	3224100.873	1991108.164	2839298.016	2689241.841	965581.6082	1165475.9	446971.7874	6788.329232
P01942	Hemoglobin subunit	Hba	2436	8	2.20	1.97	550433661.3	102160815.1	286753414.5	118250751.5	440388281.4	442784685.1	499170869	340895482.4	279265435.3	286034927	503586385.5	908107013.4
P11680	Properdin OS=Mus m	Cfp	133	8	0.69	0.63	4518828.791	6349554.121	5235554.462	4992463.894	3586245.773	3562742.161	3333429.034	2901225.33	3246057.812	2997264.682	3179886.335	2722592.857
P05366	Serum amyloid A-1 p	Saa1	259	8	100.00	100.00	663547.7104	389750.339	186373.1897	147627.4905	246640841.2	200300567.5	294272200.3	274873779.1	140047110.1	124347780.1	38601560.72	116137411.4
P11859	Angiotensinogen OS=	Agt	65	9	1.60	1.48	2672901.82	3027763.966	2794248.998	3068194.87	4019909.411	4450530.569	3723267.273	6421505.501	4984247.864	3908395.26	3608629.105	3377382.491
Q9JJN5	Carboxypeptidase N (	Cpn1	97	9	0.54	0.46	4748400.891	5881707.418	5386002.404	5680345.479	1873758.759	2696185.775	2625740.901	2577714.838	2249946.624	1983972.405	2263185.599	2182610.565
P01869	Ig gamma-1 chain C r	Ighg1	673	9	1.08	4.02	9570824.599	29385700.34	14921671.95	18336310.36	18871579.5	17613212.67	15395528.73	24312489.65	64402699.01	71938647.33	87154399.38	57463413.28
P01837	Immunoglobulin kap	Igkc	717	9	2.37	6.76	7896101.607	9246627.836	9490793.426	9789381.232	28472418.87	19028317.14	27042620.95	20331762.86	49592276.81	62175374.23	70314005.79	30159212.26
P04247	Myoglobin OS=Mus n	Mb	136	9	3.14	1.23	2274905.619	713436.3791	1643230.521	2951965.233	8613118.127	7489498.608	5423773.102	9209151.255	2009036.628	2802440.603	5786174.906	2254217.922
Q8VCS0	N-acetylmuramoyl-L-	Pglyrp2	95	9	1.37	1.68	13436899.34	14250033.57	11694547.55	12970711.64	7329995.972	9411463.273	6632952.322	9341724.141	9697402.85	9999013.692	11002389.25	12554082.69
P16301	Phosphatidylcholine-	Lcat	77	9	3.52	2.12	2480363.919	2780598.589	2456780.155	2412213.408	8085477.227	7663657.304	9710880.652	8256508.374	5975843.039	6190730.65	4655362.548	3978644.641
P28798	Progranulin OS=Mus	Grn	81	9	5.31	3.34	309372.9688	463812.6883	411783.2806	391301.5679	2591202.046	2962620.514	2328590.418	2800536.081	2007519.735	1900406.569	751127.6918	1212376.492
Q03734	Serine protease inhib	Serpina3m	4719	9	3.15	2.89	639522.4746	1058469.876	848560.7507	1227634.05	2902216.95	3883928.347	2714236.466	2739785.349	2845300.404	2700639.43	2435367.198	2868669.183
Q8CG16	Complement C1r-A su	C1ra	27	10	1.03	0.52	21617408.93	25642357.62	26125197.24	27798455.87	27327901.64	22684113.86	26191697.21	26533192.76	20608240.39	23321639.9	21923840.48	11051288.72
Q9R098	Hepatocyte growth fa	Hgfac	79	10	0.79	0.73	1800418.435	1951731.848	1800932.465	1568191.034	1754820.202	1945848.439	1640813.316	1460581.179	1206989.672	1495671.638	1492290.869	1343106.358
P70389	Insulin-like growth fa	Igfals	36	10	0.15	0.15	2806830.844	2191311.853	2055282.097	2154392.919	185457.9152	320694.4463	172538.5544	131396.6865	297906.1356	166978.1796	340085.1047	126076.5931
Q07456	Protein AMBP OS=Mu	Ambp	276	10	2.72	3.10	17817273.8	22290144.43	19322955.48	18272193.87	50357102.88	54507355.27	53003790.02	52000423.5	65371221.49	65476516.84	60871795.9	60197585.15
P07309	Transthyretin OS=Mu	Ttr	617	10	0.43	0.35	279406384.6	270236617.9	312376955.2	295258219.3	125096530.1	126522764.5	130860422.8	140210122.7	103359221.1	110501110.4	113104387.6	129399762.2
P22599	Alpha-1-antitrypsin 1	Serpina1b	6244	11	0.80	0.63	649557135.7	572351475.6	543127880	613512321.6	412715354.1	477726932.1	536538399.9	515638825	363827938	409395033.4	348854268.8	367594889.9
P00920	Carbonic anhydrase 2	Ca2	111	11	9.45	8.09	3468086.105	540436.962	1510708.637	805596.6265	9325114.884	12431387.17	11295994.68	11219529.19	10138522.23	9787528.227	12319342.81	17094238.04
P01867	Ig gamma-2B chain C	Igh-3	268	11	5.73	16.00	2330143.494	4518021.448	1132699.468	1938343.567	23033715.42	12131503.57	16722873.99	15689303.77	33524076.06	45858538.22	54809290.36	44972454.61
P31532	Serum amyloid A-4 p	Saa4	255	11	2.15	1.41	20837337.3	22689872.79	16066009.54	13832996.31	35488038.54	42012332.07	43223651.11	30656130.86	25663738.95	31163656.49	24030411.16	20286700.66
Q9QWK4	CD5 antigen-like OS=	Cd5l	166	12	1.27	1.14	5127821.542	8650816.837	9237937.949	8665108.572	12051497.77	9845921.364	11004364.31	9403212.234	8857416.115	11320244.73	10591620.52	4469427.406
O88947	Coagulation factor X	F10	127	12	0.45	0.50	10262094.75	11455458.28	10339116.6	10402275.56	4186229.446	5549224.75	5214396.722	4482168.261	4543870.894	5134222.14	4759448.604	4568681.908
Q06770	Corticosteroid-bindin	Serpina6	178	12	0.57	0.19	13407260.64	14784241.47	12562712.94	15500200.01	6311073.718	5911232.574	6521013.006	5718254.972	1859542.518	5785069.992	5596834.75	2477170.178
P01898	H-2 class I histocomp	H2-Q10	405	12	0.53	0.47	46077334.16	39284714.67	37117998.14	31027424.34	24776368.1	19552762.89	18964149.61	12914868.6	18503159.55	19697309.93	16647335.24	17069073.72
P63017	Heat shock cognate 7	Hspa8	59	12	10.27	7.90	488108.1094	75055.26947	135682.7089	259095.4758	3457406.987	5378944.909	5176668.551	3670721.822	3609188.279	4273459.045	1883222.192	4797746.71
P03987	Ig gamma-3 chain C r	1	987	12	4.12	9.38	8826339.135	28265850.59	6613008.697	7441073.368	59971180.36	33924238.24	33804182.31	40974910.95	84025895.58	95120323.14	114834523.5	63519590.09
Q60963	Platelet-activating fac	Pla2g7	63	12	5.07	5.89	604596.6748	688848.4428	729188.8895	689817.7305	3868012.726	3633210.984	3847739.527	3828195.835	6097225.322	5870459.626	4191810.018	5950987.946
Q8R121	Protein Z-dependent	Serpina10	108	12	1.50	0.85	3727074.449	4725861.96	2986681.128	3939444.051	4458829.587	6206945.738	5753568.602	6381826.918	3242025.06	3874008.905	3731508.509	3070813.406
Q91WP6	Serine protease inhib	Serpina3n	2345	12	8.25	5.16	8071971.922	8225740.339	7510160.657	8420650.606	50276707.33	40910615.12	52455923.01	65758935.3	38716205.98	33164671.16	24294871.27	35050450.25
P12246	Serum amyloid P-com	Apcs	360	12	6.87	5.74	17145999.26	19787925.87	12638036.02	12167062.88	79254297.28	99218116	88000690.83	96792387.11	101560589.9	91687624.74	74215011.56	78073130.2
Q9QXC1	Fetuin-B OS=Mus mu	Fetub	362	13	1.51	1.74	19508931.99	21544181.15	16656550.75	18594990.87	28175170.52	29275183.21	26567248.56	30992308.74	33922722.33	31187465.27	39080443.56	25391576.16
Q61646	Haptoglobin OS=Mus	Hp	106	13	0.15	0.10	24487545.55	21425583.55	9198642.544	18792159.75	1820682.992	3187662.431	2392771.078	2678282.716	1639391.936	1970048.19	1034678.181	1625638.399
P08607	C4b-binding protein	C4bpa	351	14	1.30	1.26	32717129.66	39065727.75	40473199.73	35746747.75	50366877.26	47706602.72	52826117.22	37494915.04	52562095.69	56648050.99	39276433.48	47897636.16
P21180	Complement C2 OS=I	C2	68	14	2.15	1.67	2761420.094	2577647.941	2481794.088	2441081.198	5165039.89	6221772.844	5587675.012	4483335.572	4878740.976	5132734.554	3944845.163	5590794.423
Q8BND5	Sulfhydryl oxidase 1	Qsox1	138	14	2.24	1.84	16298039.72	14631098.07	5628660.909	14083380.08	16121612.7	16062224.52	16459588.2	19487177.63	13352879.04	15424691.24	13862136.49	13036741.33
P29788	Vitronectin OS=Mus i	Vtn	415	14	1.02	0.95	45071349.46	68267324.8	67440710.12	39492166.22	54699199.35	35766038.97	32776527.72	46833274.05	41238763.78	39859031.19	58362012.66	39274358.55
P29699	Alpha-2-HS-glycoprot	Ahsg	1437	15	1.20	1.21	227788522.1	244891248.3	207500744.1	192052210.8	202875622.7	245470994.1	224157255.3	240602531.8	252515374.3	231797459	249931323.3	266798007.8
P23953	Carboxylesterase 1C	Ces1c	654	15	0.34	0.22	219695635.5	240735064.5	226062202.3	241380370.6	92348879.27	87278607.19	78817766.32	79287975.11	51384099.86	62907011.92	69024930.42	34455742.7
Q9DBB9	Carboxypeptidase N s	Cpn2	252	15	0.43	0.43	22247252.16	25097050.48	25098335.97	24353426.1	9344085.959	11094327.45	10381780.93	8836472.089	11346636.8	11749411.25	10733775.14	9707666.042
P49182	Heparin cofactor 2 O	Serpind1	174	15	1.06	0.94	9339895.277	8695961.43	6767588.917	8240998.463	7994409.524	9362091.719	9886126.399	8551637.834	6801019.972	9752125.516	7016754.384	7743740.629
Q64726	Zinc-alpha-2-glycopro	Azgp1	256	15	1.59	1.62	13914496.47	14616224.12	13230906.53	12268913.98	16382077.16	19353001.18	21487474.31	19288121.31	22431922.26	21553568.59	22336854.25	21067564.68
Q61247	Alpha-2-antiplasmin	Serpinf2	364	16	0.88	0.66	36545532.22	41850666.96	29001936.69	25998321.33	28733103.93	28911726.39	21372924.11	28358554.38	24831597.93	27760705.81	25088885.29	23400230.95
Q8K182	Complement compor	C8a	220	16	0.57	0.36	9525158.191	11774481.76	9621522.47	11688051.85	6288708.022	5871643.059	6847053.238	7026888.654	3597045.807	4171822.037	3731103.56	2911544.524
O70362	Phosphatidylinositol-	Gpld1	253	16	0.74	0.96	24473483.63	26254823.58	21830141.44	20762113.92	17391874.66	18623864.06	17918674.05	12947873.13	23804495.96	22716468.38	23935660.47	20500479.78
P97290	Plasma protease C1 i	Serping1	431	16	3.01	2.00	19865883.18	22444875.89	17314745.4	20716566.81	53965218.1	64330329.18	56309269.16	57385764.54	42342438.47	50214989.52	33207412.31	36173967.26
P42703	Leukemia inhibitory f	Lifr	141	17	0.24	0.08	9260455.648	9790133.071	8098544.22	9670017.053	1552409.013	2153979.415	1738071.113	1305889.076	746335.4247	796959.7408	627957.2587	603712.2355
P08226	Apolipoprotein E OS=	Apoe	419	18	1.79	1.40	56197201.97	46262919.97	42596821.98	50493603.43	78648303.06	70490156.36	85398520.08	52542570.16	67138711.5	80822007.24	55341870.24	57711699.2
P35441	Thrombospondin-1 C	Thbs1	118	18	0.21	0.66	11261025.06	1341647.783	15102752.36	11711262.76	2852577.509	2089719.854	3951156.162	2811019.04	2882586.706	9691302.406	5380580.494	10196889.64
P06683	Complement compor	C9	203	19	0.82	0.33	12148350.48	11635557.3	11113921.05	12767983.35	8009013.993	11380231.57	10968996.62	8876489.927	3982004.632	3300018.569	3578720.955	3477758.135
Q9ESB3	Histidine-rich glycopr	Hrg	336	19	2.66	2.47	12402563.18	13268181.03	1806679.714	11842094.38	22471576.86	20293525.53	26546070.71	47512483.68	27137084.59	20481505.89	37409202.16	30302012.67
P26262	Plasma kallikrein OS=	Klkb1	195	19	0.41	0.40	14378000.3	17143809.97	14561251.75	18037312.82	6212104.397	7411315.817	6350374.841	5468219.135	6860336.309	7086491.927	6318182.364	6777062.418
Q61129	Complement factor I	Cfi	343	20	1.02	1.06	23866810.88	25043663.76	22161044.61	23972695.51	24094101.42	25022535.91	22231594.51	24083923.86	25491802.34	26655352.68	25040678.32	22931808.38
Q06890	Clusterin OS=Mus mu	Clu	563	21	2.28	1.56	49146079.23	54044871.34	47688369.3	50698339.79	97396547.52	105520424.5	102222672.8	96619019.67	84255711.29	77055746.62	59957763.6	73801232.72
Q01279	Epidermal growth fac	Egfr	367	21	0.70	0.54	25085551.6	26795136.54	28268271.28	28730131.98	16176871.4	20663655.41	20603572.77	17059343.09	17726264.22	14351618.07	13706390.8	12809275.38
P01872	Immunoglobulin hea	Ighm	654	21	1.34	1.07	98617947.23	99246996.78	115205326	108880995.9	135345926.9	106157966.3	123971521.9	95401482.69	111253620.2	139817317.4	131648331.6	94027335.17
P32261	Antithrombin-III OS=I	Serpinc1	690	23	0.76	0.81	45458073.69	61680917.16	33095475.5	44771119.22	29620286.74	36063450.15	35108936.3	40019194.9	38925193.54	37400734.36	36718398.67	32158340.88
Q61702	Inter-alpha-trypsin in	Itih1	315	23	0.78	0.63	18485997.03	19242911.55	18370428.81	19683446.29	12898465.15	17844044.33	15052122.91	14920705.13	14549499.52	17228771.54	17113447.45	14576640.51
Q61703	Inter-alpha-trypsin in	Itih2	501	23	0.90	1.14	35474536.72	36174293.91	34225280.55	34971863.08	34401581.24	36418794.08	34074038.07	33015314.87	46674657.1	48463138.69	40225872.78	29551090.61
O89020	Afamin OS=Mus mus	Afm	368	24	0.44	0.30	34317617.57	35999746	37708679.7	33989085.09	16941343.72	18375308.14	14685395.49	15279644.63	9373985.881	12965122.38	12485448.88	11229629.65
Q01339	Beta-2-glycoprotein 1	Apoh	690	24	1.37	1.48	93723561.85	98888318.13	99354758.54	105206789	140356046.4	136055640.6	155543042.3	138562599.6	151633282.5	159770874.1	138833804.6	154321218.3
Q8BH35	Complement compor	C8b	242	24	0.55	0.40	20897585.97	25007731.32	21080232.43	25516162.38	9981965.605	13387523.68	10882118.45	9524453.339	8237597.085	9544675.49	9190784.07	7588899.938
P13020	Gelsolin OS=Mus mus	Gsn	461	25	0.65	0.38	48038998.94	45781078.96	49141334.88	50802473.98	27026971.79	32038865.86	25589445.65	30625460.39	19394606.4	22854440.9	23533398.16	23870482.61
O08677	Kininogen-1 OS=Mus	Kng1	934	25	1.38	1.38	161794877.4	187560101	143936879.1	171142105	203622037.5	221761235	204192494	262592239.9	253029600.8	234336363.9	260995940.7	231801463.6
P04186	Complement factor B	Cfb	626	27	2.07	1.44	25177763.82	27853127.73	29164884.55	30319464.15	51562757.08	55698519.3	58785022.26	65985014.76	42222677.19	48443160.13	47354879.38	36146030.55
P07759	Serine protease inhib	Serpina3k	16982	27	0.71	0.66	1404396271	1171534679	1374055316	1474402685	818963664.6	924391992.2	940038185.3	990203637.4	1029805304	876922546.2	911334022.1	1007647220
Q61704	Inter-alpha-trypsin in	Itih3	1199	30	5.62	5.87	16605246.32	20294829.02	11993568.37	13343317.34	77214980.46	85679710.22	90506079.47	88940980.09	105137206.5	99842438.92	86247447.27	79560775.27
P06728	Apolipoprotein A-IV	Apoa4	1214	31	1.08	2.39	188590001.7	177016326.8	178706098.4	153393550.8	212980685.6	160989117.8	232527629.8	166037029.8	490486031.8	393954861.5	251040240.9	444959655.9
P19221	Prothrombin OS=Mus	F2	606	32	0.81	0.72	76309388.92	64457542.9	54649572.17	68843152.46	44297659.14	48499837.41	47893814.45	38623157.33	41225469.28	44233319.34	50325088.81	39002507.72
Q00623	Apolipoprotein A-I O	Apoa1	2857	34	0.73	1.00	1408570363	1481638380	1481863063	1375446593	1250265554	1068899128	1117671638	1002700051	1452390421	1449501055	1333945044	1154303975
A6X935	Inter alpha-trypsin in	Itih4	1283	36	2.54	1.91	61917197.53	68831803.3	54597404.68	55405944.13	119351782.1	135121278	141395140.3	157822972.4	126128353	120794195	98252998.98	102337569.5
Q9DBD0	Inhibitor of carbonic	Ica	632	37	1.32	1.47	36961411.05	34942268.64	36341897.21	36186448.67	41580588.93	52246641.05	44266287.71	48748102.02	51525548.46	53893308.37	53888220.83	61062055.61
P21614	Vitamin D-binding pr	Gc	1705	40	1.13	0.78	186277194.1	208318356.5	216401445.3	203761283.4	185923553.5	215723365.8	207426299.6	254533981.5	159478704	173613143.7	169988564.1	159577653
Q91X72	Hemopexin OS=Mus	Hpx	4966	41	3.79	3.40	489082332.7	492956539.5	375561167.9	455596041.1	1677092294	1808483535	1740545981	1886089006	1492605438	1497244428	1531661004	1447306759
P20918	Plasminogen OS=Mus	Plg	1397	50	0.79	0.71	191321361.7	220006852	189869881	200009826.5	159285817	152698242.7	151267012.3	156694342.3	128185158.4	153275454.2	149191065.3	134908534.4
P28665	Murinoglobulin-1 OS	Mug1	7924	53	0.51	0.60	982944053	961776456.5	882318201.5	823450094.8	426215514.7	433493092.2	467582693.3	372640982	628974843.8	645516835.3	620000836.6	522097057.3
P06909	Complement factor H	Cfh	1335	54	1.80	1.55	70527506.85	88973402.2	81899285.41	94408614.71	151554104.2	160909471.5	171432037.5	160068411.8	143225117	145259254	129923449.8	108102427.1
P01029	Complement C4-B OS	C4b	1292	60	1.55	0.90	58237041.85	62981067.56	52408823.18	62264562.44	77731784.52	81174693.21	86805482.57	95074436.42	61408145.41	57250630.48	51333010.01	56891438.29
Q61147	Ceruloplasmin OS=M	Cp	3381	61	4.32	4.49	128318721.5	134345206	126978603.1	121710722.7	452629109.6	509483809	497945151.8	534351631.6	576434938.9	538608865.3	554014381.8	500011750.4
Q921I1	Serotransferrin OS=N	Tf	10111	65	1.22	1.06	2221774722	2136314615	2100542392	2125230854	2306686306	2689651471	2447462174	2477463309	2259836225	2428950775	2309284243	2287871916
P11276	Fibronectin OS=Mus	Fn1	1162	69	1.74	1.53	83315146.53	62868130.81	67891367.68	65862295.19	125066799.6	105283542.8	136412024	96588917.8	133043044.4	106799688.8	83978907.24	80939558.15
E9Q414	Apolipoprotein B-100	Apob	560	76	1.72	1.60	87890108.56	89307534.56	69149020.01	73918217.77	74203943.22	82818543.11	89944082.29	64685742.88	76101792.14	79783802.54	59184805.06	58158167.17
P07724	Serum albumin OS=N	Alb	29367	82	0.81	0.84	9740703554	10718702761	10847554410	11075203438	9383542396	8620893924	8459157346	9468039650	9140567834	9265994471	9297634337	8455899698
Q61838	Pregnancy zone prot	Pzp	13739	88	0.76	0.79	2034165711	2292462602	2280751586	2337234868	1945773846	1970173079	1950716802	1452069250	2012503461	1858209352	1679499552	1758059048
P01027	Complement C3 OS=I	C3	4048	115	1.17	0.78	531160321.9	572985870.1	453667852.9	465118097.2	529233810.6	588903793.3	550490011.9	610088109.8	438371776.2	415226313.1	383898387.1	307444527.3

Supplementary Table 2: list of serum proteins identified and quantified using the DTT approach in the conditions control (CTRL), 7dpi and 9dpi

Accession	Description	Gene Name	# PSMs	# Unique Peptides	Abundance Ratio: (7dpi) / (CTRL)	Abundance Ratio: (9dpi) / (CTRL)	Abundances (Normalized): F1: Sample, CTRL	Abundances (Normalized): F2: Sample, CTRL	Abundances (Normalized): F3: Sample, CTRL	Abundances (Normalized): F4: Sample, CTRL	Abundances (Normalized): F5: Sample, 7dpi	Abundances (Normalized): F7: Sample, 7dpi	Abundances (Normalized): F8: Sample, 7dpi	Abundances (Normalized): F9: Sample, 7dpi	Abundances (Normalized): F10: Sample, 9dpi	Abundances (Normalized): F11: Sample, 9dpi	Abundances (Normalized): F12: Sample, 9dpi	Abundances (Normalized): F13: Sample, 9dpi
P63101	14-3-3 protei	Ywhaz	3	1	100	100							145789.2442	43303.16782	40758.95122	65032.95548	53153.45949	
P07758	Alpha-1-antit	Serpina1a	1768	1	2.89	0.794	20415.528	75700.34526	39617.57793	147040.2126	64493.93561	96249.09277	388694.5442	243438.0957	46939.15617	13302.07608	119589.6889	58633.10598
Q00896	Alpha-1-antit	Serpina1c	2210	1	1.31	0.469	26557518.95	25500142.75	20872970.64	34331226.61	31505936.22	65422016.98	41470382.79	20862654.97	14012295.23	8231205.94	15679879.12	8968771.367
P00687	Alpha-amylas	Amy1	5	1	1.477	0.563	37564.86895	129151.7214	48505.05022	100999.1059			333236.741	31743.74941	202212.1875	16290.43403		35503.3319
Q8BFZ3	Beta-actin-lik	Actbl2	134	1														
D3Z6Q9	Bridging inte	Bin2	6	1	0.223	0.764	137455.1146		183153.2371	88229.27316	30602.95962		10722.91013	53701.61829	97266.93375	139781.2553	104951.2711	
P0DP27	Calmodulin-2	Calm2	18	1	0.746	0.664	82316.02016		265701.3258	15427.75349	61645.64319	61368.26172	10005.34516	259901.5449	25326.66807	110501.5229	129568.6944	411478.0793
P16015	Carbonic anh	Ca3	1	1	100	100							241385.9232			21369.01439		
Q9JHH6	Carboxypept	Cpb2	1	1	100	100							22298.98459		74834.64921	55963.64186	120527.1857	
P10605	Cathepsin B	Ctsb	2	1	100							139398.7813	136777.408	16123.17258				
P70269	Cathepsin E	Ctse	1	1	0.975	9.947		11915.98644					11622.56626		29535.51235			475616.5097
O70370	Cathepsin S	Ctss	3	1	8.656	6.803	13157.80999	13803.86139		14494.8952	68052.52052		132266.6843	119486.5335	110240.7631	63527.67667		93905.94749
Q8BH61	Coagulation	F13a1	30	1	0.168	0.208	552653.7234	842045.4397	2608908.334	1458510.624	243005.0227		241223.1794	142820.4308	334037.5027	158959.0652	233923.2526	262736.0702
Q07968	Coagulation	F13b	3	1	0.01	0.01	57067.12499	59645.13568	65466.49387	82771.21378								
Q3SXB8	Collectin-11	Colec11	1	1	100							311167.2188						
P14106	Complement	C1qb	3	1	0.01	0.42	9635715.315	6214768.793	9600257.818	3797197.658				19820.29313	2113639.628	3898948.69	3429757.955	4034995.384
Q8VBV7	COP9 signalo	Cops8	1	1	100	100					22405.93549		21519.94949	120407.1104				142471.34
O08997	Copper trans	Atox1	6	1	1.12	2.194	72867.73081		196082.8731	14831.69612	56191.12939	32168.83594	24141.41505	383633.1096	141508.2459		159861.1219	261714.0284
P14847	C-reactive pr	Crp	2	1	1.05	1.403	50088.07182	74200.61021	103829.7809	98018.53573			110296.9834	51999.00957	50260.9404	119659.3712	168910.3121	
P56395	Cytochrome	Cyb5a	7	1	100	100					80574.52593		22102.57928	195987.4637	116493.6774	110922.3329	104069.442	391480.0756
P10518	Delta-aminol	Alad	1	1	100						43968.83941	64256.71094	38931.37122	16408.24572				
Q8BPB5	EGF-containi	Efemp1	7	1	1.044	0.01	30977.77421	95529.63129	19921.22709	152744.2967			110375.6706	29243.36869				
P20029	Endoplasmic	Hspa5	2	1	100	100							77872.07516	120994.3425	68536.0709	200861.3012	125391.5949	
A3KGK3	Fer-1-like pro	Fer1l4	1	1	100						11944.22007	450993.625						
Q9WVH9	Fibulin-5 OS=	Fbln5	3	1	3.156	3.466	30725.12171			13100.14492	52152.58588		76914.71806	52110.4203	196071.4061	50950.12312		40470.75847
Q02596	Glycosylation	Glycam1	3	1	0.195	0.333	150480.0542	124681.0491	341430.2519	32129.31925	26708.58734							45632.89501
P14426	H-2 class I his	H2-D1	44	1														
P04223	H-2 class I his	H2-K1	44	1														
P14430	H-2 class I his	H2-Q8	76	1	1.889	2.603	103106.5129	100230.5495	206464.6446	48764.39057	252561.1445		78212.77239	305063.3815	293188.4692	429568.5273	353327.8441	88909.20823
Q5EBG6	Heat shock p	Hspb6	1	1	100							78081.83594						
Q80T19	Hepcidin-2 O	Hamp2	5	1	0.217	0.291	290924.342	396891.9419	585783.9078	242365.2335	73752.7904							98767.12035
P70349	Histidine tria	Hint1	5	1	1.231	1.35	147370.7266	101194.5714	75850.68097	163864.282	119980.5764		139568.767	188249.2583	151721.1897	92260.9585	155405.4605	279709.8719
Q60972	Histone-bind	Rbbp4	2	1	0.455	3.745	34969.65983	107577.7673	38041.56475	151198.1355				29120.24372				239585.1081
P01750	Ig heavy chai	1	1	1		100										91895.61604	217253.9465	
P18526	Ig heavy chai	1	4	1		100									270715.772	694686.4609	1074114.945	
P18531	Ig heavy chai	Ighv3-6	1	1		100									51504.25254	146445.5935	240543.7756	
P01800	Ig heavy chai	1	1	1	1.067	0.01	58135.1706	168094.8128	60204.34435	35854.89587	18137.365		193584.9517	63133.82411				
P01662	Ig kappa chai	1	106	1	3.473	14.812	91081.78684	106101.0027	194003.582	173303.3999	616702.7446	379428.0938	651828.471	293900.6234	3530489.17	3110283.036	3623501.997	2750563.7
P01664	Ig kappa chai	1	200	1	4.984	62.672		45674.62854	57118.00661	50474.50232	229754.0114		227645.2487	1149624.459	3080562.844	3248307.233	2341293.293	4231714.128
P01661	Ig kappa chai	1	78	1	2.013	59.746			15793.01642		31797.90537		16772.16699	181935.3965	953020.8118	858294.2917	934217.2362	1515726.431
P01674	Ig kappa chai	1	10	1	2.989	9.386	38527.54168	192080.9493	235765.7623	250156.3548	287342.8298		1063782.72	403556.5615	639333.1306	1843598.727	3321911.203	1763197.459
P01663	Ig kappa chai	1	106	1	0.308	1.529				108949.698			30882.53704	36395.41856	159809.9371	173640.3257	143986.7056	318792.199
P01665	Ig kappa chai	1	265	1	7.498	55.885	586707.751	1655745.976	1123145.927	1467604.405	5970371.451	2009167.32	8499050.927	9691540.539	54235875.03	41644113.04	46671936.26	47772647.69
P01666	Ig kappa chai	1	261	1	4.721	30.386			67220.01474					317350.2029	2149990.7	1940505.603	1254631.912	3218396.852
P01680	Ig kappa chai	4	8	1	1.909	2.124	203069.526	181095.2079	381840.9069	134909.537	766341.7116		19111.1085	368739.1738	308037.198	399698.8159	516992.6141	414947.6014
P01646	Ig kappa chai	1	26	1	2.966	11.592	40119.17689	237697.5229	90459.22625	243971.6473	402967.3951		714352.5848	189368.9663	1245924.757	1346661.919	3608702.944	391381.1597
P01652	Ig kappa chai	1	1	1	100	100							12601.69843		421602.5944	308822.1147	98666.5729	
P01642	Ig kappa chai	Gm10881	12	1	1.334	100		392002.2344	121159.9152	274102.7367	183165.6068		522926.4335	332272.3314	58839298.17	60054595.8	53398010.57	121745.4754
P01636	Ig kappa chai	1	2	1	1.634	1.329	67289.77037	246814.8789		114868.578	187839.9343		381027.073	109958.3589				152644.7506
P04945	Ig kappa chai	2	11	1	4.435	9.78	30513.42203	80564.13429	64442.59471	129814.0454	354521.5948	253657.6094	360088.9142		747126.9855	664608.5834	836962.5066	411015.4816
P47876	Insulin-like g	Igfbp1	1	1	100								171134.6183	71293.77127				
P47877	Insulin-like g	Igfbp2	1	1	0.01	0.01	29696.05317		28377.46782									
P47878	Insulin-like g	Igfbp3	10	1	0.163	0.384	260534.1426	85354.36945	282499.5566	191284.8733	36298.65624				128866.0503	104193.9841	36072.24587	16408.24006
P47879	Insulin-like g	Igfbp4	11	1	1.444	1.551	228942.3586	230969.6277	159253.816	120516.3783	64871.10536		280281.9762	393283.4745	330915.3972	436001.7136	265123.7015	95904.62272
P70389	Insulin-like g	Igfals	7	1	0.01	0.01	37404.61781	58760.50859	27614.15624	64743.96418								
P08730	Keratin, type	Krt13	39	1	7.008	15.758	81305.88846	27027.99873	72409.6506	45554.09051	400339.238	110783.9297	404685.4095	1114599.403	638103.4427	688489.8681	748442.0592	1946837.292
Q6IFX2	Keratin, type	Krt42	17	1	0.529	1.607	20177.27653		30393.31952		13108.33627					17382.27438		91073.27435
Q3UV17	Keratin, type	Krt76	15	1	4.876	8.835	130733.0025	39128.78558	106645.2531	121188.0057	547745.4722	560883.625			1123351.453	1100992.739	571760.5665	550509.018
Q8VED5	Keratin, type	Krt79	31	1	100	100					158885.53	195107.8594		140977.9676				395720.5001
P61110	Kidney andro	Kap	11	1	0.051	0.257	20915.73361	128530.6348	183545.6104	146154.346			6948.841774					35272.41928
Q8VCC2	Liver carboxy	Ces1	102	1	0.311	0.201	425033.8316	943069.4961	1601642.717	1279669.842	11369105.6		36590.74833	342171.5284			10366.37079	2650235.247
P00342	L-lactate deh	Ldhc	2	1	19.92	9.36	18583.18181	5265.674298			173240.7325	224123.375			68566.94081	145877.8736	144351.262	58772.52999
P97873	Lysyl oxidase	Loxl1	6	1	0.402	0.336	224514.7218	24792.29796	271717.3983	85544.14917	46697.48783			66535.41928				46570.71244
P34884	Macrophage	Mif	3	1	3.112	3.997	96042.67196	23666.63061	62619.96933	38944.76143	120769.996	384127	73916.39173	183718.3756				197361.2434
P45700	Mannosyl-oli	Man1a1	1	1	0.647	0.344	66727.58417		109126.217	49740.39126			10696.65602	212213.3096	27604.49288	19085.44406	22943.31631	
P28667	MARCKS-rela	Marcksl1	3	1	100	100							39801.10781	62473.06251	192607.1929	395717.1399	288830.3085	
P02798	Metallothion	Mt2	5	1	100	100							40253.26518	107963.1488	85875.77118	85976.92736		
P62774	Myotrophin	Mtpn	2	1	0.01	1.677	56536.48027	15992.7505	80299.44555	15177.51572							15632.30611	219143.2989
P11672	Neutrophil g	Lcn2	1	1	100	100							224077.9789	52744.94579				22340.77199
P97298	Pigment epit	Serpinf1	2	1	0.231	0.284	52507.36778	66896.07807	69935.36378	46484.58465	11904.15715	28230.00586	10952.91358			13146.50537	19986.4213	
O70570	Polymeric im	Pigr	8	1	0.878	0.77	98244.0855	22880.31741	231679.8753	39562.14814			26996.59826	110860.5698	113194.9596	88826.67133	15006.99573	
Q9R1P4	Proteasome	Psma1	1	1		100									29622.87737	85749.58787	69653.24266	
P99026	Proteasome	Psmb4	1	1	100							46019.57813						
Q8C6C9	Protein LEG1	Leg1	5	1	100	100					40077.52453			136380.3564	122522.4599	90019.77853		172028.1426
Q8BK48	Pyrethroid h	Ces2e	1	1	0.01	0.01	20297.68501	88685.89665	19472.46136	83543.87996								
P52480	Pyruvate kin	Pkm	1	1	3.005	1.207				29177.6311			87664.70208				39551.33319	31346.43773
Q8K1I3	Secreted pho	Spp2	3	1	0.241	1.771	99425.60542	60616.90593	80578.44565	69777.15885			18060.966		160721.5596	79156.74264	156394.8931	
Q63836	Selenium-bin	Selenbp2	1	1	100							64547.10938						
P13609	Serglycin OS	Srgn	3	1	0.368	0.138	440009.9074			329489.4339	140288.867				13170.18802			210194.6496
P09036	Serine protea	Spink1	14	1	2.644	1.208	158511.0398	15457.81035	100157.6434	89124.6848		20397.35547	249780.5358	1464792.115	92145.27918	141462.5412		
P04918	Serum amylo	Saa3	127	1	100	100							95078.51748			25953.08707		
P07214	SPARC OS=M	Sparc	1	1														
Q8CIT9	Suprabasin	Sbsn	3	1	0.01	0.01	51289.87655		59066.85344	5717.82862								
P26039	Talin-1 OS=N	Tln1	1	1		100									50562.3831	44758.37869		
P10639	Thioredoxin	Txn	5	1	4.72	13.698	301215.8318	22757.43729	184029.3722	23480.09325	175126.9296	139765.1719		688774.9885				900413.6394
Q9Z1T2	Thrombospo	Thbs4	1	1														
P17751	Triosephosp	Tpi1	8	1	1.446	2.27	36430.9765		36198.04648	50366.75953	26749.83552		93979.24965	52667.18667	112886.9482	114351.6134	45274.14361	
Q8CBR6	Tsukushin O	Tsku	2	1	1.073	0.974	11123.95477	35087.1528	29022.31766	80682.74717	6336.306585		163221.8147		21620.60636	44725.54683	19288.443	44739.42727
Q6P5E4	UDP-glucose	Uggt1	9	1	0.39	1.345	127107.3089	70793.27329	371619.9696	158106.585	33242.63964	55281.06018	32235.42037	472491.4666	207746.1785	254467.683	217744.1184	52743.83939
P35455	Vasopressin-	Avp	3	1	0.01	2.949			29184.94892						62823.07422	86052.40663		150983.408

Q9CQ60	6-phosphogl	Pgls		5	2	1.765	5.531	44340.29466	10015.55493	48229.17687		83947.00356	47263.17969		127036.5474			17695.17438	318448.3347
P68134	Actin, alpha	Acta1		242	2	7.101	5.631	308708.2778	210066.3728	290936.3207	440953.4108	2513359.87	1853442.742	2688100.963	2673365.076	1530695.49	1860560.943	2128434.473	1440738.276
Q91XV3	Brain acid so	Basp1		3	2	100	100						24204.74805	20511.20974	66254.37939		47581.46408		
Q80YC5	Coagulation	F12		11	2	1.038	0.411	131692.6178	152601.2468	235445.6774	128243.462	216531.9594	117258.0391		45416.22586	36212.94035	49836.63971	19412.51745	25725.71461
Q64739	Collagen alpl	Col11a2		2	2	0.184	0.93	123075.0819	218638.8537	294298.6679	162429.925	28219.77701		15862.23371		125021.6858	77924.4919		133344.8762
P98086	Complement	C1qa		3	2	0.01	0.01	524968.9598	886539.2136	138046.8296	1340497.987								
Q02105	Complement	C1qc		8	2	0.01	0.231	264316.8696	343152.3906	263573.0457	218460.5082								33257.17809
Q8CG16	Complement	C1ra		10	2	0.612	0.397	7096483.873	5815306.612	4831384.666	4677532.42	3147127.344		3876260.124	2909721.467	1262987.309	4143767.98	5561667.319	832127.7846
Q8CG14	Complement	C1sa		3	2	0.01	0.01	89372034.5	86080278.2	114226637.8	78487610.45	26638.50216		51426.56925	19211.35276				
P21180	Complement	C2		7	2	1.709	0.679	72631.76762	48468.74634	43332.9292	17320.16733	61827.6302	19479.31079	86275.3951	65652.89816	14057.87324		23377.07743	
Q64695	Endothelial p	Procr		19	2	1.543	1.019	567765.526	242619.7763	178147.8962	218309.3458	478999.995	46343.21875	270954.3538	379740.9566	185131.7489	242617.8622	237632.0386	430243.1011
P09528	Ferritin heav	Fth1		2	2	2.643	8.256				10733.29956	28363.09558	24111.52539				28166.2955		88615.6207
Q9R098	Hepatocyte g	Hgfac		6	2	0.838	0.696	59766.66613	82099.25568	30857.11382	108709.309			81603.15692	23759.9468		30102.81778	33190.87514	
Q8K0D2	Hyaluronan-l	Habp2		5	2	5.819	3.169	11874.38496		21082.45271		50759.69704		40985.56311	398033.2217	119276.5376	267517.7373	70929.38111	51561.6461
P01786	Ig heavy chai		1	9	2	0.458	2.729	182376.2979	239828.0592	184388.6995	312698.7441	96378.3451				323464.7656	760858.3324	1044945.957	
P01633	Ig kappa chai	Igk-V19-17		27	2	1.484	3.41	981961.6751	721649.2198	1060992.995	486610.7843	1500058.275		699774.973	859883.5684	1207071.167	3510164.912	4503522.238	429694.9131
P01660	Ig kappa chai		1	177	2	5.668	17.701	60071.10348	129510.9463	174372.2751	200349.2216	4241076.205	1091612.563	4477811.015	2101875.744	8880405.659	15047011.65	18148741.43	9087438.829
P01670	Ig kappa chai		1	119	2	4.881	23.696	941253.1433	2406081.823	1287456.894	1090263.178	6311892.481	5363301.648	6341306.804	6578925.56	33169579.13	26233886.29	28177125.89	27636857.03
P01635	Ig kappa chai		1	16	2	3.307	2.209	134844.9555	488303.4603	57048.11874	281614.0738	9703191.36	189446.3906	901873.8749	334117.1658	623461.8164	825003.7243	1201960.111	44378813.55
P01639	Ig kappa chai	Gm5571		25	2	2.78	4.364	57753.74457	263540.8899	151770.221	426395.4332	490079.8498				1046625.375	824688.9623	791630.9484	561893.2437
P01843	Ig lambda-1		1	33	2	3.411	1.417	135954.5342	1040837.525	176023.8035	1093177.844	1741089.393	179360.6094	1211964.118	1062938.804	841724.4802	980747.2918	1493454.412	88547.45046
P01845	Ig lambda-3	Iglc3		8	2	1.73	1.075	67705.16069	116298.3747	41302.23216	209241.0573	92045.56143		129634.5205		67714.61777	166945.4731	104484.5504	22979.29064
O55023	Inositol mon	Impa1		4	2	2.452	6.795	15395.23696				42577.10326	162499.3438	33461.61517		89768.13747	309532.1482	199806.9146	84233.97457
Q3V2T4	Keratinocyte	Krtdap		78	2	0.094	0.058	2194126.737	2289644.382	5065796.652	1685793.096	325299.3675	362545	135492.0662	206436.9317	191870.6779	90651.97182	354465.5674	99080.12066
P39039	Mannose-bir	Mbl1		7	2	0.126	0.78	250577.6349	156260.2087	248958.0115	234731.9799	12803.36464				261907.3533	333108.9175	277852.7961	
P41317	Mannose-bir	Mbl2		3	2	0.01	0.01	263959.5264	206621.3348	273364.6769	160600.7903								
P26645	Myristoylate	Marcks		23	2	1.901	2.252	164243.6339	18078.58221	285802.0215	44792.98901	250955.3374	212478.3633	111360.1434	1015762.445	435132.253	806603.2054	67503.35686	252299.4356
Q9Z126	Platelet fact	Pf4		4	2	0.01	0.471	347722.9595	9379.56162	354888.6446	248763.0309						78073.52846	108010.6499	
Q80XD8	Proline-rich	Prap1		2	2	0.01	0.01		4854.744283	27691.89292									
P43025	Tetranectin	Clec3b		3	2	0.264	0.01	51468.02638	69026.66135	149018.5983	79568.28701	11184.48522	47853.26563						
P63028	Translational	Tpt1		2	2	1.453	1.224	15328.29761		24578.74584	23057.55681	94741.98188		34590.97129		18756.19071		28118.63863	104054.9906
Q3V2D2	Uncharacteri	Gm94		2	2	0.01	0.614	257378.046	209740.3102	591888.3344	148906.4302					74931.95217		618683.5472	134336.6265
P54726	UV excision r	Rad23a		6	2	1.937	3.466	132877.1299		61288.11318		73244.80898	39292.61719		246411.5683		24909.19836	78000.52183	721889.4921
Q9CQW3	Vitamin K-de	Proz		4	2	0.01	5.963	118986.9032	95326.80755	141319.1585	90832.66746					109194.4102	259030.3608	384110.5275	
Q11136	Xaa-Pro dipe	Pepd		6	2	2.432	0.368	64959.44605	3217.872651	59485.97894	11793.46585	46350.44891	174191.0625		89504.98593		11057.93799	8578.963623	
Q62523	Zyxin OS=Mu	Zyx		7	2	0.37	0.753	100493.8956		199810.744	122599.1739	25200.15111	5289.718872		53541.21214	112857.6725	98975.07044	13863.92122	157626.0786
P07743	BPI fold-cont	Bpifa2		18	3	1.819	0.702		51189.77996	35604.75689	42509.71666	88565.96644	9453.863281	578260.4341	68092.96201	1844720.786	11761.58468	77651.85463	24735.88827
P11087	Collagen alpl	Col1a1		5	3	0.01	0.115	123619.8382	329253.0359	396471.0839	420040.3205						27824.75633	25188.50632	
E9PV24	Fibrinogen al	Fga		22	3	1.815	0.505	291678.6279	267992.9137	935113.8392	373075.7001	75930.78809	138133.5625	524671.5655	181063.2622	119525.484	250408.8608	79381.10246	31243.42748
P12804	Fibroleukin C	Fgl2		4	3	100	100						100139.2813	6613838.937		104692.4514	124281.1163	24946.43486	
P05064	Fructose-bisp	Aldoa		4	3	100	100					13651.62181		53736.4443		18427.60232	57193.81639	221985.8977	
P16045	Galectin-1 OS	Lgals1		6	3	3.044	1.489	114720.6406	65566.89505	165748.8307	15065.88339	699883.865		71393.00188	253255.2439	20136.49953	110469.2813	147692.9419	241469.7184
P16858	Glyceraldehy	Gapdh		5	3	3.364	0.995	66392.37377		86034.34263	42532.44272			495832.2782	218972.3269	14890.91096	107316.5356	503511.7559	21191.7586
P04441	H-2 class II hi	Cd74		17	3	3.233	8.86	47632.40234	44655.4586	33696.55442	71503.22153	390048.714		210564.2625	321570.2673	322643.9734	602526.2917	236427.3102	1152778.8
P06330	Ig heavy chai		1	16	3	1.324	7.986	44413.98678	213517.0755	232942.7304	90264.24136	108413.1535		267463.3127		865560.8634	2661344.996	5177819.7	137997.3864
P01631	Ig kappa chai		1	26	3	1.535	1.716	1726520.626	1719438.437	1634214.575	1772619.067	4180425.936	807614.3906	5938675.031	961336.5847	2361490.035	2652014.997	6257839.805	3964814.905
P01630	Ig kappa chai		1	11	3	1.357	1.865	159133.2678	516066.7228	139872.8726	75118.30213	218392.4914		499542.1709	132631.2109	393214.2076	762725.3114	1520549.581	95005.02631
P01638	Ig kappa chai		4	13	3	0.01	2.624	40174.97101	111187.3027	99517.82666	204059.4665					261182.2118	632966.6722	1175710.001	140310.415
P01723	Ig lambda-1		1	15	3	1.25	1.552	61740.80425	368286.7673	138793.9863	329106.9532	276874.7677		191444.4715	495917.8722	348969.9661	288913.6318	687430.3952	103469.9778
Q9Z0M9	Interleukin-1	Il18bp		46	3	37.617	33.461	60078.49559	109400.0947	74140.4975	42780.64047	2621008.435	2752935	2256737.042	5462838.024	4406834.193	4725205	2588558.793	6262221.384
P04104	Keratin, type	Krt1		47	3	4.829	10.052	542095.1502	270289.7683	514829.0324	294139.6355	2028515.303	863223.6094	2048700.155	2623620.666	2699367.689	2158067.522	3079100.934	6314013.533
Q3TTY5	Keratin, type	Krt2		30	3	10.44	18.227	32846.44365	32644.16754	36567.92989	24385.34006	595866.6696	63483.50781	525431.1595	1299835.458	1031159.851	906158.9553	885050.8366	1753604.135
Q922U2	Keratin, type	Krt5		60	3	4.708	7.204	221058.6275	78150.7415	129975.8228	112089.7693	789360.9458	761423.0625	429001.6057	897988.0892	991467.65	963455.2427	918518.6067	2700603.972
P05977	Myosin light	Myl1		6	3	100	100					37970.35022	295528.3359	181551.2933	299469.4381	56928.88147	23444.47493	26250.50842	24551.85388
P10923	Osteopontin	Spp1		17	3	1.584	0.725	220540.2647	48734.65125	231799.3978	141674.4833	316515.0335	67960.54688	153285.2597	396087.6489	67342.96422	67214.41118	26670.59126	358168.568
Q9Z2U1	Proteasome	Psma5		9	3	3.998	8.399	25157.99125	25122.04752		50254.52348	125953.0412		129803.4385	426703.9456	1475142.435	2676704.383	2563174.18	733309.1463
Q8R121	Protein Z-de	Serpina10		12	3	0.858	0.646	72737.9993	167683.2653	94961.33551	120583.0682	111340.6959	94882.13672	368696.7645	58898.35957	27451.90719	44119.07486	92475.74432	17781.66156
Q03734	Serine prote	Serpina3m		1115	3	2.346	2.383	6472.896172	20022.61861	13940.53985		45638.60994	312033.3438	16967.01169	33989.10088		50754.89969	109582.7621	29662.36535
Q8K558	Trem-like tra	Treml1		23	3	0.117	0.275	1148208.63	54500.00553	2560051.121	738403.5933	40248.484	578296.1729	10494.92325	116630.9906	39435.94353		17813.32147	315655.0629
Q06318	Uteroglobin	Scgb1a1		32	3	4.839	3.115	781971.2017	374190.5592	5562838.68	590829.9833	2665717.068	3375249.531	7359448.06	8375236.749	1117182.794	1305049.341	1670826.979	7269111.183
Q00897	Alpha-1-antit	Serpina1d		1846	4	1.673	4.354	7914893.926	3786393.684	4410305.845	3398286.858	7278878.154	12673762.24	5651991.051	14817568.87	17911117.81	12662530.84	12792424.16	21819207.87
P34928	Apolipoprote	Apoc1		63	4	0.172	0.297	13922152.39	12949391.63	15907166.68	8752259.677	4149079.682	525596.2012	2117934.043	1996019.982	3886312.694	4374425.205	3958246.107	1942671.486
P33622	Apolipoprote	Apoc3		3239	4	0.319	0.459	409429549.6	451967462.5	679804884.3	362430798	149833321.2	165258804.7	117990300.3	258163303.1	289697191	248585706.3	168886860.4	285424196.2
Q61268	Apolipoprote	Apoc4		36	4	0.321	0.415	3840107.009	3272787.164	2308368.995	2783210.638	868930.641	1389530.875	723678.7859	1040657.355	540733.9635	604491.6793	322410.3515	909923.4138
Q9Z1R3	Apolipoprote	Apom		40	4	0.618	0.261	832396.0412	1317625.545	1004971.221	1646410.487	792737.4677	467298.0469	686668.763	330786.8744	255836.43	233495.2591	499868.9423	108348.4699
Q8CIF4	Biotinidase C	Btd		28	4	1.393	1.263	222567.8441	385926.3584	233445.3047	344530.9371	413864.805	361643.7422	253592.6798	521946.959	338121.5388	194494.6101	323574.9569	544855.3821
Q61508	Extracellular	Ecm1		22	4	1.245	1.957	1168717.741	193952.0741	860885.4901	235858.6703	2332239.695		8844.381924	470746.4961	927746.4625	777532.6317	1185399.908	3599620.096
P01654	Ig kappa chai		1	200	4	2.176	10.673	383194.2065	6623236.312	5619861.424	7613239.474	2397356.749	1408211.276	3450709.038	3519494.361	9028307.596	7752986.275	10779577.41	10013453.68
Q61730	Interleukin-1	Il1rap		11	4	0.318	0.321	449388.5058	1139312.171	632157.151	1304590.587	42507.41678				27329.03708	207596.7112		47793.69848
P02762	Major urinar	Mup6		13	4	0.116	0.061	566624.0784	612149.7165	963173.2259	342646.3593	64616.90148	13277.63477	7525.661054		12778.35828			
P10404	MLV-related		1	14	4	4.683	1.859	183108.2268	230636.9085	1382905.116	147698.2681	675853.1593	350195.5	1237243.312	457967.935	24652.17859	40242.28916	68683.75457	336982.3175
Q60605	Myosin light	Myl6		17	4	4.602	4.244	339310.5234	75009.3237	113684.0866	42465.74662	649871.5844	267643.3984	165852.2755	590790.7488	294379.3163	398100.4436	286447.9951	1212110.702
Q61171	Peroxiredoxi	Prdx2		10	4	2.332	0.282	153879.7748	37556.99812	63709.8328	48205.61407	32367.91893	282924.4688	1160110.234	408295.5928	10472.78605	55536.0367	13730.96136	
P11680	Properdin OS	Cfp		50	4	0.454	0.579	676685.1812	502957.6898	557360.1641	879146.2897	144410.6046	98856.75	363418.9255	311518.8888	246139.6406	529696.3522	343887.0169	19841.12768
P12399	Protein CTLA	Ctla2a		47	4	0.199	0.534	3080716.232	75558.79325	4536327.945	1294486.828	464296.3493	337183.25	208347.0738	749263.3732	792183.0168	2228706.224	730264.8753	1647003.686
Q05020	Apolipoprote	Apoc2		616	5	0.323	0.462	51957981.09	44647029.93	69331235.39	29865697.44	13208086.67	13799639.55	5227388.112	17374443.53	19884688.45	18510512.22	13229700.16	29182957.92
O88947	Coagulation	F10		11	5	0.337	0.383	545104.4248	427658.5354	507835.6157	319120.1756	148945.3362	96938.59375	136985.8338	56678.92236	22278.9933	87222.33933	164027.0413	58675.65481
Q8VCG4	Complement	C8g		32	5	0.281	0.323	1298359.896	2220142.177	1487253.905	2206227.512	351400.3999	151759.0156	342849.5999	169406.6676	762207.6808	2343063.367	3721322.759	115728.7558
P46412	Glutathione	Gpx3		16	5	1.515	0.928	819861.674	728536.8651	1089816.799	654996.1044	2830418.691	800920.4297	1774170.129	462865.5704	207469.9591	334754.2795	574001.2993	6356577.629
P49182	Heparin cofa	Serpind1		7	5	0.487	0.247	363912.3006	663842.0916	537289.3574	502748.2304	139653.4935	114300.5234	257291.9331	266454.3429	90653.93379	132202.775	99184.23052	49935.53378
P01878	Ig alpha chai		1	45	5	2.296	0.78	174987.3958	1124982.819	347718.7994	792042.1222	715925.8757	422083.8066	2078919.382	1705245.991	740167.6647	503329.0377	394532.733	245137.1156
P01807	Ig heavy chai		1	20	5	0.414	0.833	454932.3253	1013065.619	489027.8136	670478.7035	204349.7175		388226.7884	205683.3108	460601.5498	1273244.133	3201341.42	122330.9471
P04939	Major urinar	Mup3		12	5	0.405	0.074	701941.5011	139244.5562	3129676.841	17599.81166			17513.14351	16782.55582		40198.83895		
Q00724	Retinol-bindi	Rbp4		26	5	0.517	0.236	434366.9712	888714.9679	612863.0488	1228167.396		4386686.93	855398.5968	144461.8098	73929.53296	121345.7295	240445.7942	49752.27188
Q60590	Alpha-1-acid	Orm1		1445	6	5.404	2.975	2466865.878	2128367.965	9535451.426	2468898.934	27496481.97	21876441.08	7530530.288	7617907.094	8597336.076	9168797.245	14720884.96	17543031.37
Q9JJN5	Carboxypept	Cpn1		43	6	0.165	0.407	828137.4037	842383.3434	1283093.351	1352037.408	68589.90938	90712.10938	164353.3136	97891.34412	107079.2698	324027.4432	438634.4106	
Q8R1U2	Cell growth r	Cgref1		22	6	0.624	1.474	2181959.973	2238914.501	3862585.062	1448908.128	616481.88	295698.375	530678.1671	2689109.871	4229739.865	2803681.629	1407009.014	5975642.234
P03953	Complement	Cfd		192	6	0.794	0.399	3957667.186	7758244.933	6882052.369	9196212.346	6488947.836	6861458.695	7969800.632	3695932.212	1865640.592	1936854.664	3162041.863	2468496.756
Q9QUN9	Dickkopf-rel	Dkk3		10	6	2.324	2.286	63074.14436	105992.4599	157680.2126	80223.46182	281644.3916	129142.5469	397795.3601	563637.3479	332097.1827	269868.5394	229301.9041	719611.8377
P01865	Ig gamma-2A	Igh-1a		132	6	4.554	45.508	174341.4681	680292.9238	552886.014	574709.5168	7482743.153	847709.6875	2511689.538	2984288.624	12028105.6	34742541.03	85370720.25	7151920.947
P02535	Keratin, type	Krt10		93	6	8.692	14.024	126034.756	219687.4066	335976.0242	255793.0729	3735683.292	505474.4688	2231330.595	6616916.316	4590828.002	4867350.464	4894835.196	10695001.78
P51885	Lumican OS=	Lum		24	6	0.579	0.186	827138.8738	564851.1033	3157176.538	551169.8125	309861.1428	270249.1797	37382.87139	105808.9291	31656.61643	29715.02452	37575.25007	236504.1711
P70296	Phosphatidyl	Pebp1		34	6	2.02	2.223	665632.9163	178769.1626	1212643.162	390556.3391	934925.7227	487829.3125	607027.2952	1291656.068	721054.0609	930800.8114	1121538.171	2077499.442
Q60963	Platelet-activ	Pla2g7		28	6	8.714	5.32	66575.65799	7417.968394	42523.93179	7009.732039	603458.6385	386850.25	1544888.07	384567.8374	947508.0063	315116.4528	762899.9789	172637.1006
P70663	SPARC-like p	Sparcl1		23	6	0.875	1.095	171566.9603	11800.79882	342288.9058	272150.7452	92969.92828	10890.01074	294397.573	176532.0322	46001.21301	309416.9836	21478.20497	

P20065	Thymosin be	Tmsb4x		123	6	1.051	1.157	2824114.106	763681.2805	3387772.797	994810.1566	2383574.696	3256788.725	519948.2055	2791090.337	3588559.854	5108720.551	2705935.573	1092442.139
Q91V80	Apolipoprote	Apof		156	7	2.187	2.166	598946.5255	811011.4775	1707564.542	550683.5701	1982440.597	2200068.949	1467829.373	3354638.317	1614217.501	1108135.48	650724.1827	3573143.877
P00920	Carbonic anh	Ca2		49	7	4.716	2.819	654456.1528	281561.9641	355214.5459	323530.0486	1821441	1044007.125	2789297.84	1185477.706	931706.8605	1184249.363	1767961.199	437290.5677
O09164	Extracellular	Sod3		22	7	0.52	0.389	1171342.437	777623.7877	840270.6628	704356.7614	301625.1374	444804.5313	115037.6905	189178.3563		25756.95406		293533.1477
P04247	Myoglobin O	Mb		23	7	2.886	0.263	132390.1278		67361.29502	208264.2883	735471.8271	825661.0859	621339.7293	151226.1733				37563.74883
P31532	Serum amylc	Saa4		98	7	0.772	0.542	2240093.004	3497330.319	2257192.035	3231956.727	1475018.896	3273015.574	3895201.692	1693114.368	975290.3484	1750039.736	2718154.723	7167989.767
P07361	Alpha-1-acid	Orm2		1351	8	11.783	6.64	2692460.292	6033416.257	12416857.15	3995045.191	79903007.6	96806547.28	60775037.87	40788970.67	34549314.28	46426419.24	36917877.2	52661408.01
P09813	Apolipoprote	Apoa2		1513	8	0.838	0.866	385543128.1	374517362.2	646128480.1	313462569.9	165372057.3	194104819.2	97495864.07	204404358.7	177615635.9	210948359.4	185612289.5	283378099.7
P01887	Beta-2-micro	B2m		538	8	0.857	0.785	44859091.56	46068942.92	38191372.61	36583989.69	37531487.97	29015998	29518933.85	44118231.17	45409244.5	39498198.89	35340690.9	40432490.94
Q9QWK4	CD5 antigen-	Cd5l		79	8	1.304	0.669	1113648.595	1113689.305	1149668.379	2642060.635	1487301.966	701919.3047	3948920.122	1708025.744	813820.4268	1526405.181	1374512.385	148112.1684
P01869	Ig gamma-1 (	Ighg1		82	8	0.819	3.645	726937.139	3895674.173	1122164.676	1824852.566	968806.2899	2902127.5	1336817.761	713343.125	4097268.287	6654473.319	14026782.3	673909.7904
P01592	Immunoglob	Jchain		265	8	1.397	1.766	14218360.24	17688473.1	15286509.41	15592731.51	17307537.39	11440560.7	10931206.9	29163685.5	23482410.5	26325718.37	18001759.27	25533653.67
P42703	Leukemia inh	Lifr		29	8	0.152	0.256	755935.4206	1135220.833	1205356.585	685704.2	32133.02657	23275.06836	26890.73341	21618.19233		48794.58202		
P70274	Selenoprotei	Selenop		129	8	1.25	0.684	2156962.644	2934135.042	1916323.929	4234917.956	3700704.227	1624514.232	2474848.037	2062636.224	2024482.505	1275757.715	1107981.756	1506287.798
O88783	Coagulation '	F5		126	9	0.818	1.414	3760144.288	2406899.726	4631810.419	2129612.644	2344220.106	2442285.115	991761.4059	4707996.575	4030554.835	4689268.124	3960665.824	5509088.644
Q8BH35	Complement	C8b		58	9	0.276	0.225	1531826.908	2532630.227	1824101.85	2111048.551	363696.6555	138634.1484	494568.3964	268405.791	134209.8495	251767.725	398392.6854	137306.8748
P02089	Hemoglobin	Hbb-b2		1059	9	2.937	1.685	85337429.25	21546124.67	44236231.8	26183575.42	102501880.9	75351342.44	92998805.5	103349143.2	59542637.78	41296969.22	75527996.28	132975750.1
P01867	Ig gamma-2E	Igh-3		60	9	2.8	9.391	119094.8484	503934.4603	139683.379	181618.0729	299219.8869	273275.7744	881371.9282	760102.8842	1521586.042	5190444.242	8249719.592	272162.2663
Q8VCS0	N-acetylmura	Pglyrp2		56	9	1.784	0.542	4749156.744	5129131.12	2730510.969	11207560.65	1829248.384	1305268.495	18042962.04	6519834.37	962867.1827	609364.7946	1302447.353	251006.3937
P26262	Plasma kallik	Klkb1		44	9	0.184	0.353	1250534.719	1753610.159	1450385.333	2338939.792	107106.8522		536497.7317	220353.2105	265657.5586	583630.4475	430090.7473	20146.99494
Q07456	Protein AMB	Ambp		182	9	3.699	2.601	1686267.48	3287915.013	1529242.211	3695300.14	7074134.657	4338938.729	14198787.36	8445874.185	6727124.31	7156415.351	9203163.641	2079889.708
P08228	Superoxide d	Sod1		88	9	1.991	2.241	4289173.088	717227.6554	2366865.688	1043123.771	4196596.646	2125809.77	922874.3965	4092338.553	22429167.55	14649197.71	14197890.04	11233125.84
P51910	Apolipoprote	Apod		690	10	1.579	3.259	58068126.44	39197707.73	79805484.92	20452939.66	46649274.9	66542525.91	16740709.29	86988075.04	129936471.3	142648516.5	100439181.2	128107636.9
P08607	C4b-binding	C4bpa		94	10	0.619	1.055	2189660.281	3589210.193	3461460.287	3007310.176	879330.6885	1545483.906	1835099.269	1992165.899	1932865.031	4586132.448	4724640.041	453223.8503
P06683	Complement	C9		34	10	0.416	0.133	1277003.976	2126326.722	1657343.428	1758360.649	258359.3872	234146.6563	242124.3792	54742.82533				196756.749
Q01279	Epidermal gr	Egfr		74	10	0.631	0.386	1321522.644	1941540.834	2034196.505	2729236.489	295827.0739	882498.7578	1154708.933	364199.7191	423995.4312	813179.5892	1252496.917	21898.46029
P01942	Hemoglobin	Hba		3980	10	2.24	2.332	655657163.3	150663904.5	558337518.4	193717247.4	876089446.5	777238636.7	451448878.4	748195771.9	554952816.7	532876726.5	670297154.7	1220848978
P52430	Serum parao	Pon1		125	10	0.261	0.233	5439907.777	13669687.04	2892148.564	18775214.11	3066442.52	1428097.462	3557863.106	2122804.468	2409990.642	1543514.55	4631911.839	622366.1593
P35441	Thrombospo	Thbs1		30	10	1.437	0.506	1214648.8	151266.9342	1719530.769	919887.0035	8781.384152	202228.3906	193226.4529	1243976.295	83841.41086	1787717.37	1502703.455	77329.36576
P22599	Alpha-1-antit	Serpina1b		3180	11	1.359	0.692	151299441.3	170905129.8	124232197.6	217623274.7	220559925.4	308309075	193508110.3	148306073.3	137049065.3	97279186.55	133683403.5	133646366.6
Q8BND5	Sulfhydryl ox	Qsox1		54	11	1.147	1.327	719378.5439	704252.8981	754638.5041	745738.6971	2284366.64	569364.1094	2318792.974	1452087.285	1017759.4	904701.6509	578351.8997	1016247.481
P60710	Actin, cytopl	Actb		387	12	10.938	4.036	3103590.545	2713883.791	3559309.623	4259680.322	30265289.1	30635171.8	50126670.45	19209103.37	12635610.49	12504036.81	11821939.89	7228691.407
P11859	Angiotensinc	Agt		90	12	1.519	1.165	1502299.624	2030088.452	1031046.434	3316570.02	2858040.194	2439048.469	5517312.539	1268228.218	3054511.42	1843522.609	1438967.034	2544586.402
Q9QXC1	Fetuin-B OS=	Fetub		191	12	1.333	1.404	2956261.815	5702469.337	3872796.382	5927011.834	4878949.735	5151499.086	15401696.24	4523883.145	7253944.754	6189456.538	11562768.16	4359379.708
P02088	Hemoglobin	Hbb-b1		1290	12	2.534	1.668	390989778.8	76989323.53	251963592.9	113582964.4	535486988.7	631722894.1	353711427.9	455717318.5	258043856.8	209726748.5	407525290.7	553364147.6
P03987	Ig gamma-3 (		1	144	12	1.592	4.075	1088737.474	2840640.341	911510.8288	753720.6263	1650842.038	1819624.051	1624993.392	913770.3251	3448209.625	8511379.468	18819856.97	823444.7328
P01837	Immunoglob	Igkc		659	12	2.684	13.034	4118327.533	8527764.313	6742370.482	9162056.189	19435377.13	11968565.99	19120474.15	26772322.34	83760358.56	91234131.11	79094339.62	89727033.25
Q02819	Nucleobindir	Nucb1		63	12	1.459	1.842	520762.4517	283521.4619	652791.7777	1676507.85	911867.2723	737185.1201	989066.61	2012250.592	2226318.221	1111464.588	2229283.483	1404504.384
P16301	Phosphatidyl	Lcat		315	12	1.238	1.622	42304897.37	45498797.13	39659892.84	37751551.29	13576803.72	13370226.45	28932206.94	45696800.43	29515913.36	26475771.25	21093420.01	24212922.42
P97290	Plasma prote	Serping1		164	12	1.641	1.172	1714935.453	2221200.1	1884737.991	1871751.731	2881858.359	2120222.204	3543088.79	2445367.541	1410226.382	3083082.142	3868185.687	460068.2248
P12246	Serum amylc	Apcs		170	12	3.342	2.214	2446886.916	6591351.036	1776596.092	3554154.656	7108117.642	12309220.43	25532093.47	8444430.495	6809614.398	9198042.532	14014482.5	1712428.88
P07309	Transthyretir	Ttr		3154	12	0.722	0.597	647599901.5	660055612.9	336769574.3	709504285.5	414463788.7	450039913.4	325387144.8	474252272.1	389052879.2	355640747.7	199081448.3	394352424.5
Q9ESB3	Histidine-rich	Hrg		202	13	2.791	3.379	1851212.632	2512402.389	785548.2465	2617408.937	3250778.363	15137965.36	7255072.216	5550318.398	5776759.432	5448315.567	9092801.897	8726516.437
O70362	Phosphatidyl	Gpld1		62	14	0.345	0.385	2455608.082	4816329.886	3125767.119	3555510.037	885833.9398	519690.0625	662474.1733	659195.5256	537505.2076	982566.3181	1960024.38	121910.468
Q61207	Prosaposin C	Psap		138	14	1.783	2.715	14762455.23	9001741.943	17853845.21	6795070.249	3681872.921	2655768.285	1918775.157	6343678.255	3732600.83	7155938.705	4321482.755	8511315.986
P05366	Serum amylc	Saa1		1062	14	100	80.805	5647864.245	3278079.334	2988754.742	1483163.113	802271657.6	796746270.7	793807443	1082041329	622048479.8	538950572.8	172693730.1	834274138
P29788	Vitronectin C	Vtn		303	14	0.84	0.613	23288512.02	27220896.41	16763571.1	22045869.52	19973680.46	10252772.97	12300672.51	21606986.99	10415203.2	11671920.35	8821299.665	10052509.05
Q8K182	Complement	C8a		88	15	0.228	0.308	2846678.864	3415876.297	2875614.2	3266912.731	392265.4092	331264.9492	645876.8455	571043.1741	433468.5208	888302.8869	868780.2987	306374.7346
Q61702	Inter-alpha-t	Itih1		91	15	0.334	0.424	2099432.389	3578597.578	3780159.38	3997180.537	654962.3808	841334.8984	536811.5756	682283.5293	478769.6303	1096160.353	1687016.276	176658.1549
P05367	Serum amylc	Saa2		1285	15	100	100	479713.8054	578699.3978	819078.8882	442814.0811	374127084.7	405974427.9	330695633	511245087.5	280501270.6	208646979.8	41023597.63	254260428.7
Q64726	Zinc-alpha-2-	Azgp1		155	15	0.965	0.767	2547736.806	5011271.589	3719126.54	6049268.233	3967145.699	2349692.303	11560928.51	3781247.154	4493647.699	4128232.502	4962192.251	662158.2237
P13020	Gelsolin OS=	Gsn		143	16	0.19	0.317	5552786.779	5053188.902	6709125.58	4288891.503	502482.1945	589744.2539	232787.6509	436796.0802	843429.3997	1811786.269	2827673.066	182865.0903
E9Q414	Apolipoprote	Apob		37	17	2.419	1.371	568356.0984	305660.2393	402448.2165	295532.2993	419109.3593	233378.9297	690065.9304	181086.5379	372609.2762	1582305.192	1470693.457	69365.90313
P08226	Apolipoprote	Apoe		361	17	0.811	0.641	17609833.85	17790656.66	17742340.13	21130911.98	16086317.82	13293922.67	9638583.135	8867217.113	6525567.924	12035890.63	13638713	2227263.544
O89020	Afamin OS=N	Afm		96	18	0.26	0.121	3071964.79	5292499.049	3539620.271	5311718.334	567731.8675	755132.3477	936954.4274	580225.8223	128331.6499	251389.9154	436925.301	182662.9778
Q9DBB9	Carboxypept	Cpn2		144	18	0.396	0.186	4010180.023	8982228.81	5243996.167	12934139.32	1861811.243	2425705.758	3133550.094	2012496.027	1238933.338	1103885.79	1738566.996	678989.4203
P32261	Antithrombir	Serpinc1		213	19	0.559	0.578	4747857.478	9087371.219	5930451.629	7497177.552	1984920.732	9545127.746	3872680.379	2199311.791	8528085.388	13035069.15	9831383.545	2241088.287
Q61129	Complement	Cfi		158	19	0.757	0.497	4039765.872	5008344.557	3364647.573	4964958.491	2220511.301	5121763.875	3976027.91	2030012.216	1282733.282	2246070.888	2878922.227	497745.0141
Q61646	Haptoglobin	Hp		194	19	0.142	0.093	25158777.73	12440324.7	8058625.369	9438915.862	591275.2558	323055.1016	1780935.165	907859.3281	478216.2984	577095.4634	285091.4515	303114.6876
Q61703	Inter-alpha-t	Itih2		179	19	0.641	0.708	3996660.821	6745883.022	6139335.719	8007419.815	3822620.062	2808592.545	5501547.187	1540680.65	2897237.545	4444538.832	7211046.176	569030.6411
Q91WP6	Serine protea	Serpina3n		702	19	5.722	2.537	1347971.935	1856148.606	835352.4413	2169605.277	8622292.002	6699061.451	19096844.5	6182415.053	4441801.674	4132529.143	5286684.909	1216825.817
P01898	H-2 class I his	H2-Q10		664	20	0.472	0.265	28633676.62	40483137.12	38004697.86	50281759.57	19456786.15	21302826.8	16560906.3	20187660.54	12351527.94	10648899.51	11079370.16	7438067.283
Q61247	Alpha-2-antip	Serpinf2		306	21	0.948	0.42	5536117.528	11487464.88	5134692.515	10987589.85	9137741.802	6864866.557	10026588.3	4705639.367	4074352.087	4296380.845	5353704.925	6044384.844
Q01339	Beta-2-glyco	Apoh		324	21	0.712	1.181	10643496.58	11907163.77	8850121.602	11129739.75	5925973.36	7331905.496	9164598.515	8829426.444	9680749.571	15654746.8	16953064.42	4934069.497
P23953	Carboxyleste	Ces1c		1316	21	0.478	0.167	119949976.2	168685073.4	82512344.25	309596852.5	68775573.38	68145611.58	80836133.73	99666814.81	39787623.15	23926321.58	34228481.29	13971595.89
P29699	Alpha-2-HS-g	Ahsg		2268	22	1.644	1.441	324187787.2	309455287.9	208294353.4	220445998	295659919	377478732.7	338079505.3	414135535.6	395623825.5	336425244.1	306417264.3	408870600.6
P04186	Complement	Cfb		218	22	1.094	1.17	3311968.678	5238960.924	4018131.914	4356888.307	3412492.769	3489034.906	7433220.658	5034675.299	5283050.761	9779126.791	13813925.17	1685353.495
Q06770	Corticosteroi	Serpina6		531	22	0.359	0.317	39061472.52	17297046.77	40071730.63	10084104.18	11164272.75	7596749.793	3307320.406	13242849.49	2906924.569	5443242.658	4781559.202	15478060.99
P28798	Progranulin (	Grn		317	22	5.76	5.983	1837835.9	1747530.569	900703.1699	1096855.858	6860605.034	8108926.572	7209436.563	16446382.26	9970088.789	11570215.34	5653979.003	9027824.143
Q06890	Clusterin OS-	Clu		610	23	1.856	1.412	44770051.92	36951700.93	19611887.64	35021466.55	78443924.88	97647156.04	60917421.66	112552327.3	81010059.89	65698632.23	36978504.79	66386264.68
P01872	Immunoglob	Ighm		351	23	0.555	0.792	12029707.46	20518718.82	15786934.04	23182125.85	8224541.917	8821548.141	12476567.81	10116939.79	7722205.679	17723484.14	30420679.15	3763580.95
Q9DBD0	Inhibitor of c	Ica		141	23	0.5	0.848	3131620.976	3869678.9	2843843.429	3715481.678	798000.7888	1200329.723	2347465.432	1255046.103	2079431.947	5175642.336	7512210.17	401848.7416
P19221	Prothrombin	F2		660	25	0.527	0.507	110637587.6	9322567.871	152681596.5	65592101.5	41324019.04	52058116	15884282.54	64594243	10816687.15	20574641.59	48262155.11	65544574.85
O08677	Kininogen-1	Kng1		633	26	0.869	1.028	29044705.92	44023483.88	39048682.53	40105867.36	25891839.71	32068368.61	47943217.67	30832566.48	32806015.93	44736921.03	58827324.27	20461547.2
P07759	Serine protea	Serpina3k		5953	26	0.848	0.453	241051353.1	385311646.3	220237099.7	472508958.7	283409866.5	280796145.8	377420476.9	202103048	211427470.5	130733001.9	224025059.7	57699304.97
Q61704	Inter-alpha-t	Itih3		562	29	5.197	3.691	2707071.876	8386143.608	2718917.78	7640355.61	18062767.87	16767363.73	27383666.91	17366497.6	16047427.18	13844439.4	22673722.24	15245230.69
A6X935	Inter alpha-t	Itih4		426	31	1.264	1	8790132.267	11610177.28	11336482.46	10241627.68	15424408.12	12493043.7	17008831.88	11031991.15	8022448.627	13647701.21	19151067.33	3769393.734
Q00623	Apolipoprote	Apoa1		3501	34	0.86	1.078	940054528.4	904985391.4	799706218.8	758745756.5	874136361.8	584314378.9	545574112.3	723807246.2	1036475879	813490366.4	691906360.6	918243210.4
Q91X72	Hemopexin (	Hpx		1989	38	2.516	1.859	54814807.66	82026844.63	51346391.33	69177397.77	154110057	277210301.7	272399093.4	130835977.8	92161908.99	155084567.1	263399460.8	34365110.89
P06909	Complement	Cfh		390	41	0.797	1.305	6783905.198	10159826.51	9489930.233	13085380.24	3173898.616	6520756.469	8807240.494	6691935.109	5970410.714	15440045.89	19658008.93	1482830.574
P11276	Fibronectin (	Fn1		280	41	0.62	1.038	9630908.967	5314812.377	9816019.323	6526271.295	3066310.068	2279301.448	5553793.504	8722668.987	5427280.303	9564246.57	10754064.66	2410986.695
P01029	Complement	C4b		446	43	0.971	0.598	6400443.727	9960123.283	6719247.504	10216726.4	7152376.179	7785104.827	12659971.5	7900045.774	5005358.167	5837860.886	9026953.15	1263788.239
P06728	Apolipoprote	Apoa4		4434	46	1.079	2.63	602487645.2	559798379.5	792905861	501410019	741180481.6	429655058.3	465551802.7	935774799.3	1975746470	1492113014	759362189.1	2029972117
P21614	Vitamin D-bi	Gc		1038	47	0.915	0.502	33350038.57	49208019.28	34431313.92	57015477.35	27905987.53	27860318.29	96097575.78	40326947.51	16581746.2	23388170.01	33112734.26	15957440.88
P20918	Plasminogen	Plg		625	50	0.371	0.416	18967582.25	30674417.31	19431753.68	26945883.72	4878187.555	8347150.949	11366925.2	6818832.875	5634194.637	12267257.04	18869576.4	1475810.854
Q61147	Ceruloplasmi	Cp		1669	68	3.478	2.034	18148761.47	42087041.46	18828100.2	49240799.33	95112208.04	95456503.94	235570405.7	74405379.11	66797578.11	73651758.28	112482904.2	13720018.66
Q921I1	Serotransferr	Tf		3573	69	0.67	0.782	377931655.6	385924870.4	358914160.6	385322773.8	222923262.6	336764278.8	357729298.7	228969955.3	227773675.1	378135807.5	500435347.8	104771379.1
P28665	Murinoglobu	Mug1		2292	75	0.282	0.324	124703482.1	200335073.8	125637171.1	159205858.1	39865115.71	59449875.73	51241631.45	29460482	40303911.14	56607599.96	112210894.8	11748415.15
P07724	Serum albun	Alb		11262	79	0.547	0.49	2373033744	2745072796	2354401427	2872340203	1724267461	1740391586	2556611587	1272015523	1175471223	1903201536	2524581370	426003158.6
Q61838	Pregnancy zc	Pzp		4644	83	0.39	0.381	307167807.7	529793015	420002539.4	501702579.5	218152366.2	256811161.1	210996401.6	140057403.9	152842785.7	213205908.9	317354171.1	89884862.57
P01027	Complement	C3		1622	100	0.524	0.483	68414278.13	92170626.81	74091671.37	69721151.63	39955517.49	59522943.6	60530225.4	41951005.64	25529270.25	46488882.57	67658818	18720174.19

Supplementary Table 3: list of serum proteins identified using the TCA approach in the conditions control (CTRL), 7dpi and 9dpi.

Accession	Description	Gene name	# PSMs	# Unique Peptides	Abundance Ratio: (7dpi) / (CTRL)	Abundance Ratio: (9dpi) / (CTRL)	Abundances (Normalized): F1: Sample, CTRL	Abundances (Normalized): F2: Sample, CTRL	Abundances (Normalized): F3: Sample, CTRL	Abundances (Normalized): F4: Sample, CTRL	Abundances (Normalized): F5: Sample, 7dpi	Abundances (Normalized): F6: Sample, 7dpi	Abundances (Normalized): F7: Sample, 7dpi	Abundances (Normalized): F8: Sample, 7dpi	Abundances (Normalized): F9: Sample, 9dpi	Abundances (Normalized): F10: Sample, 9dpi	Abundances (Normalized): F11: Sample, 9dpi	Abundances (Normalized): F12: Sample, 9dpi
P63101	14-3-3 protein zeta/delta OS=Mus m	Ywhaz	6	1	2.6	3.306	58352.48471	31399.09612	50886.89237	98026.51	144660.7	104343.3	189143.5	138723.8	174348.8	251294.5	100133.8	201002.6815
P68134	Actin, alpha skeletal muscle OS=Mus	Acta1	235	3	7.799	7.268	1300325.183	812013.3209	841048.7799	1867121.559	11018073	8258952	11673739	13344617	8688236	11025472	6345322	11766290.83
P60710	Actin, cytoplasmic 1 OS=Mus muscu	Actb	355	9	12.467	9.003	12721618.98	5836705.14	7106081.13	11144257.18	98489542	1.45E+08	1.41E+08	92214809	85025078	99031461	64809807	96195635.93
Q9R0Y5	Adenylate kinase isoenzyme 1 OS=M	Ak1	1	1	11.733	4.8		16529.04186			138454.1	271630.3	280382.9	77689.31	50557.28		180568.4	79341.02896
P40124	Adenylyl cyclase-associated protein	Cap1	3	1	2.136	2.602	44894.07037				93423.54	87696.49	99313.71	98406	98821.6	130765.6	104375.2	156047.974
Q60994	Adiponectin OS=Mus musculus OX=1	Adipoq	19	2	0.341	0.581	1597866.466	2158416.233	2401365.648	2447250.245	1682501	326394.1	339466	932925.9	940044.5	1506224	1187519	819922.2648
O89020	Afamin OS=Mus musculus OX=10090	Afm	223	21	0.591	0.296	22597990.88	25959418.34	20776601.59	12571078.85	7767216	25373911	8928417	13325731	5104741	7430736	7064630	5805272.228
Q60590	Alpha-1-acid glycoprotein 1 OS=Mus	Orm1	3043	9	5.937	5.539	18649151.06	22392178.77	23460207.75	24728761.13	1.61E+08	1.07E+08	1.48E+08	1.73E+08	1.46E+08	89083862	1.32E+08	95039514
P07361	Alpha-1-acid glycoprotein 2 OS=Mus	Orm2	2157	5	100	100	59799.50969	105155.0979	30254.86922	56823.8283	17814886	20871969	27944246	21018499	14306880	12563444	15796496	10907382.08
P07758	Alpha-1-antitrypsin 1-1 OS=Mus mus	Serpina1a	12110	2	0.54	0.357	337899594.1	406090799.6	390119924.6	322297175.6	2.54E+08	2.07E+08	1.74E+08	2.49E+08	1.56E+08	1.28E+08	1.62E+08	146357064.8
P22599	Alpha-1-antitrypsin 1-2 OS=Mus mus	Serpina1b	12060	11	0.653	0.531	1372946712	1433694236	1302439258	1082280983	1.09E+09	6.79E+08	8.4E+08	1.21E+09	9.62E+08	9.19E+08	8.77E+08	890669786
Q00896	Alpha-1-antitrypsin 1-3 OS=Mus mus	Serpina1c	13686	1	1.373	0.53	71973.46958	187611.9058	230059.9717	1020158.769	79595.26	2630288	3689771	1016183	278909.3	37619.63	61065.61	94413.40385
Q00897	Alpha-1-antitrypsin 1-4 OS=Mus mus	Serpina1d	8710	4	1.136	1.252	50669294.27	38127933.28	37631658.37	19266938.42	54863355	29722655	33419382	64778291	48080761	58495439	51161480	55914899.03
Q61247	Alpha-2-antiplasmin OS=Mus muscu	Serpinf2	608	19	0.57	0.625	64061022.5	75897483.35	57500523.42	54337258.76	43498174	32788995	42626105	36959758	42364125	47105103	40476984	32590011.47
P29699	Alpha-2-HS-glycoprotein OS=Mus m	Ahsg	1554	18	0.988	0.79	400969212.9	427298052.8	349027492.6	335225533	2.99E+08	5.34E+08	4.79E+08	3.31E+08	2.95E+08	3.15E+08	3.27E+08	341915262.4
Q6GQT1	Alpha-2-macroglobulin-P OS=Mus m	A2m	37	6	0.372	0.435	687983.8273	1037435.447	917070.633	462227.9094	231624.6	97840.67	705258.2	339499.7	577441.9	917936.1	141185.8	277427.0619
P00687	Alpha-amylase 1 OS=Mus musculus	Amy1	9	4	1.963	1.736	451700.5448	417857.637	587089.6477	520770.2592	653170.2	409000.6	1236035	566591.1	2188629	789394	489564.9	618464.8997
P17182	Alpha-enolase OS=Mus musculus OX	Eno1	9	2	14.005	6.73	31039.8709		35312.07544		536753.6	636590.7	630895.4	356452.8	222425.4	197682.5	238426.4	340658.8434
P11859	Angiotensinogen OS=Mus musculus	Agt	131	9	1.329	1.528	3588472.878	3687680.406	3899753.113	3357519.024	4771314	2874890	5696901	4918878	6947878	6072992	6314401	5159763.348
P32261	Antithrombin-III OS=Mus musculus	Serpinc1	1193	31	0.565	0.578	129536977.1	150135491.6	148977172.7	162138525.8	76524999	80908918	99222985	78265373	78325269	81643854	88559738	74995625.04
Q00623	Apolipoprotein A-I OS=Mus musculu	Apoa1	1682	28	0.578	0.655	756712955.9	839139536.8	764367641.5	906068271.6	6.58E+08	4.94E+08	4.56E+08	4.75E+08	8.32E+08	8.24E+08	5.36E+08	682393979
P09813	Apolipoprotein A-II OS=Mus musculu	Apoa2	151	5	0.222	0.137	13580965.43	19647281.8	24568178.29	23108732.54	9195481	14706262	5867400	11497346	4309491	2499792	6341660	3485030.428
P06728	Apolipoprotein A-IV OS=Mus muscu	Apoa4	742	30	0.86	2.085	93178884.32	96519928.77	93347436.9	108231848	1.01E+08	61158239	65193675	87275411	2.3E+08	2.04E+08	82000789	194106041.6
E9Q414	Apolipoprotein B-100 OS=Mus musc	Apob	1167	114	1.329	1.539	46776465.64	44529504.02	39775402	39266244.47	62900923	57718518	43634453	67634093	69846469	77485384	49974254	55028234.63
P34928	Apolipoprotein C-I OS=Mus musculu	Apoc1	20	2	0.316	0.505	2374338.238	2324061.308	1650522.616	479533.4004	1111318	189796.1	73993.16	416776.2	1532257	1963370	1003097	668109.6135
Q05020	Apolipoprotein C-II OS=Mus musculu	Apoc2	60	3	0.099	0.146	4227675.106	4517572.382	5279057.458	4633644.294	731211.2	407605.7	459155.7	466125.2	1159825	1059780	658199.3	612607.8969
P33622	Apolipoprotein C-III OS=Mus muscul	Apoc3	512	4	0.319	0.321	79293542.07	81020573.69	68360316.6	90495748.15	27137967	45181654	24707390	22978842	30549699	30116438	24152574	24993198.92
Q61268	Apolipoprotein C-IV OS=Mus muscul	Apoc4	17	2	0.344	0.411	2401254.842	2206973.266	1685462.696	1689027.995	641605.5	910230.9	335612.6	660766.6	1088031	1228086	459561.1	611129.0043
P51910	Apolipoprotein D OS=Mus musculus	Apod	166	8	1.572	2.587	11537268.04	12333642.68	10776823	12159070.25	16415510	19397722	16709934	16759863	29088084	31053213	34695064	26623572.34
P08226	Apolipoprotein E OS=Mus musculus	Apoe	218	14	1.074	1.148	16826557.26	16712359.15	13989741.32	16321928.2	25337687	13093843	9705025	22338223	20039513	24711652	14177347	21064837.43
Q91V80	Apolipoprotein F OS=Mus musculus	Apof	4	1	2.164	0.809	17915.89062		35014.73298		43727.58			67188.44		11028.32		37249.65733
Q9Z1R3	Apolipoprotein M OS=Mus musculus	Apom	52	3	0.637	0.638	6722721.433	6943749.359	5923357.322	4242589.203	5686700	3917338	2387250	5220647	3367465	4418738	5959607	3830788.131
P04919	Band 3 anion transport protein OS=M	Slc4a1	9	3	2.607	2.155	21826.59208		67435.27245		98978.05			65948.59			29799.1	421763.0598
Q01339	Beta-2-glycoprotein 1 OS=Mus musc	Apoh	785	26	1.139	1.134	148887915	156331032.2	153692956.5	137749612.8	1.53E+08	1.33E+08	1.59E+08	1.99E+08	1.85E+08	1.89E+08	1.77E+08	169677600.4
P01887	Beta-2-microglobulin OS=Mus musc	B2m	79	3	0.64	0.749	19622487.15	17954770.54	15600823.03	18747219.36	10354119	9295300	12469522	12161480	18341134	15186617	11515512	12308693.07
Q8BFZ3	Beta-actin-like protein 2 OS=Mus mu	Actbl2	128	1														
P21550	Beta-enolase OS=Mus musculus OX=	Eno3	9	3	2.848	1.274	176705.2343	86006.22323	198639.5833	92709.80679	446539.1	219736.9	216142.4	270069.3	139646.2	183231.9	496746.6	277559.0389
P15327	Bisphosphoglycerate mutase OS=Mu	Bpgm	2	1	1.33	3.374	225879.6281	147689.3545	192563.0588	76896.80613	313189.1	86691.38	143280.3	288170.5	502997.7	754788.1	568737.3	359515.2937
P07743	BPI fold-containing family A membe	Bpifa2	17	3	3.221	0.689			44242.54947	32854.11191	417300.7	31583.88	1085697	46685.02	2117618	16846.07	88812.29	101694.7464
Q91XV3	Brain acid soluble protein 1 OS=Mus	Basp1	3	1	1.608	1.07	9719.083715				8163.672	15632.71		33806.77			10402.43	
P08607	C4b-binding protein OS=Mus muscul	C4bpa	342	13	1.018	1.117	32878044.95	40207711.91	47093908.86	38238799.57	43119710	37260176	35907035	52655891	36372771	48008297	35972113	40255053.49
P13634	Carbonic anhydrase 1 OS=Mus musc	Ca1	17	6	1.825	2.002	1475909.976	79941.28801	699850.4688	165797.0894	1645981	343774.8	659515	1771341	700017.4	604604.5	1424490	2378970.609
P00920	Carbonic anhydrase 2 OS=Mus musc	Ca2	179	11	6.813	9.939	7740254.87	1121442.028	2995609.048	973674.9326	23883686	11653871	11234667	18861075	22826489	25615838	28658685	33915257.39
P16015	Carbonic anhydrase 3 OS=Mus musc	Ca3	6	3	0.701	0.829	101276.7194		819657.7199	310313.3493	123951.6	1191665	1020949	312921.7	212150.5	35146.84	956607.7	157628.4344
P23953	Carboxylesterase 1C OS=Mus muscu	Ces1c	906	19	0.355	0.222	197901747.3	217803822.2	224666059.6	260090321.5	89145000	1.19E+08	1.05E+08	98114955	67391083	70530154	92059459	48611620.48
Q9JHH6	Carboxypeptidase B2 OS=Mus musc	Cpb2	67	8	1.798	2.628	1285823.766	2110540.768	912483.6731	1161512.264	2739186	911792.8	1496181	2442706	4569096	3742079	3712094	2491197.363
Q9JJN5	Carboxypeptidase N catalytic chain (	Cpn1	136	10	0.47	0.443	10369484.58	12871015.01	14848314.76	14643534.48	5532045	7039464	4467149	5531338	5961491	5631888	5496014	4171604.632
Q9DBB9	Carboxypeptidase N subunit 2 OS=M	Cpn2	218	13	0.492	0.476	22953385.02	27854465.94	27886256.34	29744121.67	11767928	15381217	14548052	11754040	13951554	14252206	13194139	10452358.51
Q9WVJ3	Carboxypeptidase Q OS=Mus muscu	Cpq	17	4	1.279	1.067	1009013.319	817337.0709	1087334.079	1173884.972	1247234	1422617	1446898	1191600	1030097	1220681	1117775	943481.1378
P24270	Catalase OS=Mus musculus OX=1009	Cat	14	4	2.409	2.646	179423.8017	62075.13559	123977.7328	62409.99348	413418	435382	306773.1	431935.7	496697.6	980154.6	712653.7	919122.1108
P10605	Cathepsin B OS=Mus musculus OX=1	Ctsb	31	6	0.714	0.585	861071.841	1271987.748	1165454.771	1184685.539	1312210	1474195	2102860	1057909	932467.6	1022902	972167	887978.8352
O70370	Cathepsin S OS=Mus musculus OX=1	Ctss	2	2	9.038	7.067	13741.88784	25378.06473	20750.54344	28393.33768	304661	219377	473027.3	256294.1	346980.9	300212.6	209900.1	186285.9942
Q9WUU7	Cathepsin Z OS=Mus musculus OX=1	Ctsz	14	2	1.602	1.905	284992.4234	372385.4587	468994.4174	630626.9849	466836.2	261698.6	374735	359087.8	410782.6	542943.2	349187.1	583205.3051
Q9QWK4	CD5 antigen-like OS=Mus musculus	Cd5l	114	9	1.451	0.872	5823804.537	8166999.82	7786479.665	7021542.876	7761710	11158773	8947090	9390450	5346884	7536818	7995471	5175708.511
Q8R1U2	Cell growth regulator with EF hand d	Cgref1	4	1														
Q61147	Ceruloplasmin OS=Mus musculus OX	Cp	4259	80	4.293	4.349	163823569.1	186172395.1	179414537.2	189545842.1	5.83E+08	6.87E+08	7.9E+08	6.11E+08	6.55E+08	6.42E+08	6.67E+08	617660756.9
Q06890	Clusterin OS=Mus musculus OX=100	Clu	585	27	1.661	1.128	84173473.95	115491781.2	119122313.5	127093052.8	1.33E+08	1.9E+08	1.58E+08	1.53E+08	1.08E+08	95693425	87687281	113839563.8

O88783	Coagulation factor V OS=Mus muscu	F5	53	8	1.02	0.944	1746018.742	1856855.656	1403850.572	1344021.636	1761149	1285967	937934.3	1960627	1681919	1952713	1886127	1186833.281
O88947	Coagulation factor X OS=Mus muscu	F10	109	10	0.389	0.398	17007627.32	21394175.66	20692284.31	15150172.21	6607623	4684030	4122672	6386034	4867488	5507060	5957765	5122157.221
Q80YC5	Coagulation factor XII OS=Mus musc	F12	119	8	1.458	1.121	5377659.392	5820090.374	5216865.118	7461479.928	7029451	13658730	8810766	5889716	7255432	6941119	6327089	6847450.148
Q07968	Coagulation factor XIII B chain OS=M	F13b	27	7	0.42	0.384	1210936.677	1691132.544	2055421.141	2183213.841	579957.2	2932065	818281.1	697452.3	677164.1	1698416	1824971	593294.248
P18760	Cofilin-1 OS=Mus musculus OX=1009	Cfl1	1	1	0.958	0.439	27312.52569		68977.34536	84443.1202	53763.44	66061.9	76474.68			30285.12	24538.24	34100.58555
Q3SXB8	Collectin-11 OS=Mus musculus OX=1	Colec11	4	1	2.191	1.62	29484.4037	46261.59434	41971.87918	37175.84417	114909.4	96545.73	54581.81	73415.57	69786.55	70802.44	58663.78	55474.1279
P98086	Complement C1q subcomponent sul	C1qa	18	2	0.332	0.354	1207286.098	1584009.768	1797785.348	1807376.652	639595.7	349583.8	327389	454965.1	491443	385027.7	919337.2	279632.6476
P14106	Complement C1q subcomponent sul	C1qb	29	2	0.198	0.263	5076472.491	5995486.562	6703670.684	6927134.959	1240393	2140643	1367817	1288709	1562350	1766211	3303353	1124625.113
Q02105	Complement C1q subcomponent sul	C1qc	22	1	0.167	0.367	2944690.756	3373669.381	2217192.95	2147485.679	774154.2	276648.2	252722.1	733743.7	895106.7	984273.5	1745314	609982.6403
Q8CG16	Complement C1r-A subcomponent (	C1ra	31	8	0.913	0.433	4369655.089	11930195.03	4835635.336	7560995.99	6651345	4555452	3227806	6420438	2766219	1611964	3486037	2395532.166
Q8CG14	Complement C1s-A subcomponent (	C1sa	18	4	0.922	0.368	704787.856	928526.7702	1082459.517	1004985.931	810409.6	799253.7	620524.2	803997.1	289179.2	348574.3	461234.4	146882.482
P21180	Complement C2 OS=Mus musculus (	C2	127	18	2.135	1.576	5286921.869	5852163.448	6190173.805	6798465.282	9814878	12472251	11986153	10628525	7558999	6840511	7100472	8574694.39
P01027	Complement C3 OS=Mus musculus (	C3	6112	143	0.84	0.662	733865518.4	794265352.9	700807911.4	682540949.8	7.08E+08	7.19E+08	7.65E+08	7.08E+08	5.75E+08	5.35E+08	5.5E+08	420896580.8
P01029	Complement C4-B OS=Mus musculu	C4b	1795	67	1.491	0.957	75074348.47	86395564.5	81263320.46	91472645.47	1.05E+08	1.33E+08	1.26E+08	1.16E+08	89623637	85271408	71787468	67744765.22
Q8K182	Complement component C8 alpha cl	C8a	252	19	0.515	0.352	14480933.36	15442556.62	14558426.79	19585713.63	6670056	12044390	9989191	7592211	5472941	6516791	5890904	4620059.667
Q8BH35	Complement component C8 beta ch	C8b	294	21	0.488	0.397	22903310.96	26171571.62	28124707.41	33318617.32	11206780	12454225	10311954	11030079	9454020	10396354	9456161	7269004.223
Q8VCG4	Complement component C8 gamma	C8g	143	8	0.452	0.418	19358626.65	20941799.26	20491315.77	19813542.26	8963499	7972381	7307695	9701733	7826976	9804978	8251574	6620597.39
P06683	Complement component C9 OS=Mu	C9	292	20	0.822	0.338	21157599.54	21812106.1	22217201.51	20847369.92	15737872	15968630	15128545	16613217	6770132	7460204	7511386	6838318.729
P04186	Complement factor B OS=Mus musc	Cfb	865	37	2.03	1.43	35519618.08	39732816.24	41384888.94	50988876.25	67841469	94469564	1.12E+08	76487950	61717330	67250417	68271464	46702205
P03953	Complement factor D OS=Mus musc	Cfd	98	4	0.955	0.29	4500941.044	8767397.024	7448143.55	10257427.51	6098838	12068231	9947341	7576434	2111096	2096808	3441645	2142292.012
P06909	Complement factor H OS=Mus musc	Cfh	1195	60	1.69	1.452	66467938.1	73560977.04	76443940.52	89025300.03	1.08E+08	1.54E+08	1.38E+08	1.26E+08	1.07E+08	1.22E+08	1.14E+08	91503245.33
Q61129	Complement factor I OS=Mus muscu	Cfi	306	19	0.985	0.977	24866809.47	29431210.69	26987670.74	29292648.81	24496223	24583312	26957962	27185372	26535311	25356054	25657659	21831403.48
O89053	Coronin-1A OS=Mus musculus OX=1	Coro1a	5	3	2.077	1.491	70500.62786	46266.55498	38537.04672	156639.9622	303786.2	94943.84	87221.21	279062.8	188733.2	363445.3	168058.1	250726.2358
Q06770	Corticosteroid-binding globulin OS=I	Serpina6	205	11	0.36	0.195	19356600.13	25375512.42	26631452.73	20869697.1	10652869	854378.2	1552413	9386906	6490834	5664797	8919057	4119705.133
P14847	C-reactive protein OS=Mus musculu	Crp	45	5	1.861	1.232	1753041.629	1634339.656	3079511.571	923727.4765	5439064	4958064	4231857	3558552	3369172	4523215	4187470	2272708.315
P07310	Creatine kinase M-type OS=Mus mu	Ckm	26	10	4.612	2.024	725656.0954	12652.71301	185086.9441	319853.2079	1095938	3588220	3798275	1833939	393552.3	406888	3325112	1373802.041
P62897	Cytochrome c, somatic OS=Mus mus	Cycs	7	3	2.256	2.966	89903.74764	78717.41788	112012.8546	77291.94311	333905	142014.8	109551.5	338234.6	260865.3	357587.5	239419.5	356748.3411
Q9CPY7	Cytosol aminopeptidase OS=Mus mu	Lap3	6	3	6.737	2.744	23386.21442	44026.44677	36018.28304		374543	689085.9	609563.3	407664.6	340905.8	294435.9	272107.9	290548.0658
Q8R0Y6	Cytosolic 10-formyltetrahydrofolate	Aldh1l1	1	1	100							51258.29						
O55188	Dentin matrix acidic phosphoprotein	Dmp1	2	1	100							9652.195	11144.96					
Q8BPB5	EGF-containing fibulin-like extracellu	Efemp1	18	3	0.766	0.605	566458.8309	574223.555	775337.3043	706818.76	423648.1	585229.1	603784.8	266844.1	255276.8	320489.2	357276.7	241302.4658
P10126	Elongation factor 1-alpha 1 OS=Mus	Eef1a1	4	1	10.575	5.045	38579.85651			27831.11611	258172.3	543562.8	425234	282384.9	135534.7	154845.8	201596.2	173453.1279
P20029	Endoplasmic reticulum chaperone B	Hspa5	28	2	2.916	3.525	77331.51046	85761.13727	71304.75966	57232.06894	184361.1	210723.1	240524.2	258657.7	361878.1	282640.5	240269.9	428603.4492
Q64695	Endothelial protein C receptor OS=M	Procr	5	1	1.959	1.306	120324.6842	167983.9949	127937.9989	186003.3935	344361.1	182822.7	254037.9	359501.7	218253.8		244161.7	149888.3249
Q01279	Epidermal growth factor receptor O	Egfr	356	24	0.698	0.568	22545976.78	27103685.87	27559223.95	32027081.87	17592647	23971259	20848773	18869043	18272623	15263905	15604129	13046748.41
O09164	Extracellular superoxide dismutase [	Sod3	13	2	0.768	0.438	539057.421	723190.0957	1326034.54	1133291.88	628229.8	245251.3	208391	413657	225098.5	321363.8	377682.2	231695.9537
P04117	Fatty acid-binding protein, adipocyte	Fabp4	6	2	0.111	0.422			2068642.166	521775.6458		100232.5		87606.09	332416.2	94287.23	881755.5	
P11404	Fatty acid-binding protein, heart OS	Fabp3	11	3	2.724	1.367	507361.6788	27057.8345	617669.8331	669337.9469	2513476		258335.8	1272509	501604.4	775701.8	1497006	317666.1786
Q9QXC1	Fetuin-B OS=Mus musculus OX=1009	Fetub	528	17	1.57	1.587	21031952.97	28106495.35	22423427.18	24461171.76	35775140	44616691	42041246	41000303	36864340	34040333	56892323	38703166.82
E9PV24	Fibrinogen alpha chain OS=Mus mus	Fga	26	7	0.979	0.623	656263.4426	6791559.642	1481957.013	960905.7802	399858.4	2033558	1602842	2384473	862537.6	1098341	352968.7	488043.3802
Q8K0E8	Fibrinogen beta chain OS=Mus musc	Fgb	3	2	1.088	0.493	22722.78056	661525.1769	34943.89557	51130.62062		170078.1	30668.7	187764.6	22853.41	46733.56		12379.89781
Q8VCM7	Fibrinogen gamma chain OS=Mus m	Fgg	10	5	2.353	0.138	56610.5409	4733532.257	39134.14559	161173.0821		286306.2		674722		211250.2		13366.72613
P11276	Fibronectin OS=Mus musculus OX=1	Fn1	1607	72	1.212	1.281	144157500.8	82513873.86	132690956.5	117045190.6	1.55E+08	89164396	1.02E+08	1.8E+08	2.01E+08	1.62E+08	1.36E+08	118316880.8
Q08879	Fibulin-1 OS=Mus musculus OX=1009	Fbln1	22	3	0.351	0.211	1160165.299	1305500.93	1309731.299	1651666.816	483420.9	558285.6	456498.3	489119.2	253415	304271.5	314683.9	239801.6229
O70165	Ficolin-1 OS=Mus musculus OX=1009	Fcn1	15	4	0.333	0.324	801885.8714	1124884.772	1055571.274	1136600.351	267784.9	350814.1	242080.5	196418.7	293507.5	244524.7	300652.4	202197.5081
Q923D2	Flavin reductase (NADPH) OS=Mus r	Blvrb	15	3	2.551	1.392	1146786.036	137961.3655	567260.6109	191276.9631	1265476	490318	466574.3	1265123	327780.9	307754.7	1034751	1780835.476
Q62356	Follistatin-related protein 1 OS=Mus	Fstl1	1	1	100							75648.79	86849.77					
P05064	Fructose-bisphosphate aldolase A O	Aldoa	67	11	5.137	2.39	1232241.884	300409.4157	755404.0808	1068847.328	2818925	11265592	11089446	3501035	1490267	1339441	3829121	2492066.641
Q91Y97	Fructose-bisphosphate aldolase B O	Aldob	1	1	3.609	5.702			26022.71816		93917.34		56912.4	222006.3	133318.6	165153.9	52268.92	212083.006
P13020	Gelsolin OS=Mus musculus OX=1009	Gsn	432	24	0.463	0.355	65549241.84	66110473.2	79126914.28	91496344.76	38328599	12643027	25426945	33459093	23172276	27781046	28847405	22840457.47
P46412	Glutathione peroxidase 3 OS=Mus m	Gpx3	153	7	1.327	1.629	6975713.683	10234289.76	8528792.492	9720097.151	13104651	10876198	12466834	14164263	13719664	17710365	11490311	11189637.07
P16858	Glyceraldehyde-3-phosphate dehydı	Gapdh	46	7	5.606	2.48	1864384.685	214089.6513	839776.8757	1031290.458	4208226	10356368	8730706	4371355	1216355	1541843	5999755	3252352.641
Q9Z0E6	Guanylate-binding protein 2 OS=Mu	Gbp2	1	1	100	100					52303.63	29344.18	39346.92	27965.79	14847.11	17537.69	14026.9	26627.87061
P14426	H-2 class I histocompatibility antiger	H2-D1	35	1	10.909	8.471	37936.24502	28789.26316	39459.06578	33974.46149	331192.2	419023.6	425174.5	359738.1	349888.6	306389.6	232398.9	306977.73
P01902	H-2 class I histocompatibility antiger	H2-K1	26	2	5.377	10.864	134426.8569	143109.6545	165553.2309	119677.8259	1271256	626844.2	650655.7	1365832	1607995	2140195	933969.6	1411922.193
P04223	H-2 class I histocompatibility antiger	H2-K1	37	2	37.488	0.731				75406.40157		2826840	3288353	22164.33	318821.3	9519.887		
P01897	H-2 class I histocompatibility antiger	H2-L	67	1	16.61	17.96	85631.74524		73365.21434		1368214	822728.4	1266861	1693412	1499075	1711688	1135751	1351872.825
P01898	H-2 class I histocompatibility antiger	H2-Q10	434	11	0.333	0.341	68933493.05	74735097.56	73722088.88	70333036.07	36401963	36390782	26229722	39183857	29774076	28919917	27255132	25367812.44
P14430	H-2 class I histocompatibility antiger	H2-Q8	69	1	8.702	15.666	119020.6003	71526.04904	74312.68056	63182.25236	960913	438488.5	630723	1022079	1119881	1551242	990336.3	1407483.529
Q61646	Haptoglobin OS=Mus musculus OX=	Hp	188	14	0.095	0.072	75173759.12	85531162.4	35865518.16	56247462.94	5303309	5586110	6114817	5424769	5060214	5632982	2971262	4364620.23
P16627	Heat shock 70 kDa protein 1-like OS	Hspa1l	37	1														112687.5188
Q61316	Heat shock 70 kDa protein 4 OS=Mu	Hspa4	12	3	0.941	1.486	732059.0893	493167.5418	477579.3872	167042.6341	870801	75389.34	108177.9	714380.6	517224.5	1035166	820346.6	798205.2891
P63017	Heat shock cognate 71 kDa protein (	Hspa8	97	11	14.582	10.331	968038.4778	44093.03362	451481.9129	471781.9596	5944775	8196405	7900875	6813853	6977208	6845386	3725825	7497903.623
Q9CQN1	Heat shock protein 75 kDa, mitochoı	Trap1	1	1	8.026	8.282	29961.56923				240306.7	240637.3	275850.9	222536.7	216648.7	284240	118913.2	310054.7328
P07901	Heat shock protein HSP 90-alpha OS	Hsp90aa1	5	2	100	100					446895.5	326734.4	373828.6	270707.6	62853.53	169480.4	799583.7	151354.3562

P01942	Hemoglobin subunit alpha OS=Mus r	Hba	2021	7	2.097	1.937	774663892.2	199878500.8	421842053.8	190249719.7	6.74E+08	5.06E+08	4.06E+08	6.17E+08	3.75E+08	3.98E+08	6.71E+08	1150502875
P02088	Hemoglobin subunit beta-1 OS=Mus	Hbb-b1	1013	9	1.622	2.06	1029240868	244770393.3	533699193.1	228955662.6	1.1E+09	3.6E+08	3.47E+08	7.05E+08	5.87E+08	6.46E+08	8.65E+08	1578984684
P02089	Hemoglobin subunit beta-2 OS=Mus	Hbb-b2	719	8	1.963	2.443	175540127.7	34977962.15	99329923.77	40998249.11	1.74E+08	1.02E+08	86189873	1.36E+08	96658535	1.08E+08	1.51E+08	293747527
Q91X72	Hemopexin OS=Mus musculus OX=1	Hpx	5949	47	3.559	2.984	594639003.8	659736582.7	526791062.9	549495762.6	1.85E+09	2.15E+09	2.42E+09	2E+09	1.89E+09	1.83E+09	1.84E+09	1646069514
P49182	Heparin cofactor 2 OS=Mus musculu	Serpind1	212	11	0.811	1.005	16600452.54	16376569.32	17629219.25	14486204.78	16089822	7739671	8509668	14010731	13765914	19399203	15892670	12197148.67
Q9R098	Hepatocyte growth factor activator	Hgfac	81	9	0.567	0.503	2153281.564	3036533.723	2770255.636	2669014.216	1748536	1785884	1441813	1542664	1272771	1638498	1274620	1219501.905
P26928	Hepatocyte growth factor-like prote	Mst1	3	2	1.384	1.657	90200.57848	143397.1875	103375.7404	168191.8391	204275.4		173716.7	263948.6	178286	245624.8	276733.4	140193.3226
Q9ESB3	Histidine-rich glycoprotein OS=Mus r	Hrg	371	19	3.489	2.825	11699846.9	14105915.09	1517959.511	12366247.31	23921019	33011638	61816214	31069693	29416639	23185656	43845650	34508549.84
Q8K0D2	Hyaluronan-binding protein 2 OS=M	Habp2	9	2	0.662	0.579	565554.0678	450192.7128	355599.9097	352548.0326	284060.1	163941.7	331447	257952	267772.4	202680.7	346033.4	223390.71
P01878	Ig alpha chain C region OS=Mus mus	1	152	5	3.128	2.364	1398180.709	2047685.415	1900832.292	1836408.609	3999439	8948211	9717611	4868181	4333086	3443224	3730515	3233162.452
P01869	Ig gamma-1 chain C region, membra	Ighg1	365	11	1.367	4.006	14867861.02	32840837	20262320.34	35829313.75	25437666	35217379	54431189	22116873	76055050	92183965	1.09E+08	68134325.72
P01865	Ig gamma-2A chain C region, membr	Igh-1a	605	8	5.86	30.294	8473976.693	23025859.55	17818661.87	23586489.81	1.22E+08	97327303	1.34E+08	1.17E+08	3.81E+08	5.05E+08	5.48E+08	358011372
P01867	Ig gamma-2B chain C region OS=Mu	Igh-3	242	14	7.574	18.142	2465228.335	3324598.742	1310980.281	1818742.79	16112561	17744595	44066465	15745323	32342271	49502094	52446952	44859338.81
P03987	Ig gamma-3 chain C region OS=Mus	1	459	11	4.278	9.026	8933083.729	27397553.77	7586067.076	7920239.857	62280079	40214101	42217836	36763454	80416624	89289130	1.19E+08	61556307.43
P01750	Ig heavy chain V region 102 OS=Mus	1	25	2	4.781	27.552	77695.59796	255033.7073	32975.87382	115016.3616	1216648	733010.3	1030101	1224534	7160145	9425418	9817620	4356160.427
P18531	Ig heavy chain V region 3-6 OS=Mus	Ighv3-6	1	1	0.916	1.381	506571.4758	377954.5682	413961.8439	403231.4353	345024.5	306974.7	531230.6	465430.3	564048.6	617287.4	492073.9	664117.5044
P18528	Ig heavy chain V region 6.96 OS=Mu	4	20	2	2.495	20.778	123972.043	146462.7874	139216.6374	171642.7204	511306.2	466120.7	362402	590671	3115139	5233266	5194779	2858373.023
P06330	Ig heavy chain V region AC38 205.12	1	92	4	1.677	7.911	2310501.913	9683941.776	5579786.625	2844067.801	12229598	5315236	6157278	11734553	36630967	51229209	56956889	32654362.58
P01803	Ig heavy chain V region AMPC1 OS=I	1	18	1	1.378	2.636	395512.6051	930488.7251	372958.237	599089.7035	6682140	81461.43	905096.5	664041.6	1364554	1342733	1510014	965370.2451
P01759	Ig heavy chain V region BCL1 OS=Mu	4	10	1	8.318	43.556		20177.14742	19056.52362	13326.41831	234730.9	46289.67		158509.1	657260.2	854082.8	1176153	539515.5001
P01791	Ig heavy chain V region HPCM6 OS=I	1	17	1	0.64	0.892	168035.8436	195208.6499	373901.3606	306481.058	163085.6			150454.8	150262.3	332681.1	529620.8	149127.0264
P01786	Ig heavy chain V region MOPC 47A C	1	44	1	1.772	4.341	1889905.222	2150624.41	1823632.497	1583749.466	1957213	1742762	2400126	3034915	7614770	9414061	8937461	4732921.157
P01741	Ig heavy chain V region OS=Mus mu	1	3	1	100	100					86557.96			82790.9	89245.94	366507.2	363132.9	406099.4624
P18524	Ig heavy chain V region RF OS=Mus r	1	19	1	6.054	26.588	92262.25447	97052.64325	38664.90194	61301.27744	2472230	285155.2	318326	432656.7	1729241	2701520	2343066	894274.3028
P01807	Ig heavy chain V region X44 OS=Mus	1	82	5	0.897	2.669	6260023.786	6722805.319	6752118.564	5105765.364	6413500	5302690	7402507	4857065	12758518	18455963	22741072	10664636.48
P01797	Ig heavy chain V-III region U61 OS=M	1	40	1	0.625	1.646	1491532.438	4389557.1	1296859.455	823293.3722	1095194	91090.39	84349.91	569818.4	1500614	3959971	2512679	1985054.638
P01633	Ig kappa chain V19-17 OS=Mus musc	Igk-V19-17	6	1	3.553	6.28	22049.56369	205168.9225	53836.73136	39364.02338	162129.5	79121.36	189449	423473.3	312985.4	108471.8	593254.3	365271.9811
P03976	Ig kappa chain V-II region 17S29.1 O	1	10	1	0.983	2.611	391193.3313	1142979.109	507276.7962	318400.0548	802308.1	546119	398311.5	301908.1	1393343	1193289	1258934	939256.9663
P01631	Ig kappa chain V-II region 26-10 OS=	1	51	5	3.041	3.995	4233320.82	6070474.662	5537237.011	5369347.541	16526870	14741004	18517292	11929756	18725572	26787148	27040144	15937278.19
P01630	Ig kappa chain V-II region 7S34.1 OS	1	39	3	1.687	3.53	3319206.433	6281426.832	3551952.733	3841462.112	7718141	4832059	5235688	6783923	10003237	12733000	13821372	8299984.938
P01661	Ig kappa chain V-III region MOPC 63	1	78	1	0.01	5.071		11736.81958		15663.71799					42451.61	64162.3	148549.3	73672.61047
P01674	Ig kappa chain V-III region PC 2154 C	1	16	1	3.752	15.298	313895.3077	799144.3448	1116276.659	1430861.86	2190655	4127455	4370969	3043030	9827756	18386066	17987313	11606405.01
P01657	Ig kappa chain V-III region PC 2413 C	1	129	1	2.266	3.751	51558.15185	147087.1024	95515.51247	109154.5891	453792.5	231387.8	194975.3	156045.1	382998.6	303764.1	806676.7	324550.4284
P01654	Ig kappa chain V-III region PC 2880/I	1	172	4	2.557	6.517	342780.6528	2069405.275	430694.911	478618.4256	1232486	2303578	2689013	1423911	4252141	5716081	7768455	3273872.783
P01660	Ig kappa chain V-III region PC 3741/T	1	202	2	14.896	41.911	195582.7369	698042.5734	892435.3555	1561983.071	15971923	17668852	14211004	9395339	37661343	46571264	59759330	33838833.09
P01663	Ig kappa chain V-III region PC 4050 C	1	111	1	7.32	41.31	105876.4986	149837.4932	234228.7803	235295.9622	1237865	1718538	1537452	1027890	6777172	7191953	9697953	4701670.155
P01665	Ig kappa chain V-III region PC 7043 C	1	189	2	8.879	38.647	203118.586	297080.826	218769.4311	276308.3576	1115585	2526129	3069968	2032040	8371470	13067035	12428113	7875886.094
P01671	Ig kappa chain V-III region PC 7175 C	1	156	3	5.506	14.174	503644.0665	1548389.779	861826.7694	915607.8084	7758830	8492235	8248073	7511392	23376415	23068135	31354856	16160942.5
P01644	Ig kappa chain V-V region HP R16.7 (	1	88	4	4.217	16.033	418296.8743	1386468.87	688315.0646	1026461.655	3851724	6656793	6656005	3212305	15969509	22309414	22101267	9516208.973
P01635	Ig kappa chain V-V region K2 (Fragm	1	26	3	2.678	9.64	677487.8678	1996272.201	1647819.773	1828357.9	4512053	4334717	5462095	4854276	15221783	12806248	22643542	11520829.26
P01638	Ig kappa chain V-V region L6 (Fragm	4	29	2	2.737	7.978	497911.9041	702340.3293	598426.0823	1418834.745	1595987	1632502	2918010	1532585	4783097	6433662	7090811	3006799.37
P01642	Ig kappa chain V-V region L7 (Fragm	Gm10881	23	2	2.277	5.678	567098.1777	1183926.688	764944.4371	1024676.772	1614275	1464141	2194082	2297587	4955887	6720626	6157401	3515520.398
P01636	Ig kappa chain V-V region MOPC 149	1	12	2	4.702	11.313	271125.5395	466680.2838	35780.26219	504267.2022	1121729	1807610	2691812	1473905	4406340	5343426	5651084	3546173.431
P01639	Ig kappa chain V-V region MOPC 41 (	Gm5571	11	3	2.263	5.094	158396.5695	425768.7997	309884.7653	789775.2397	1002337	1284824	599906.3	721881.5	2582305	2866838	2922265	1764229.335
P01679	Ig kappa chain V-VI region J539 OS=I	1	3	1	0.441	1.007	769457.233	484538.0205	472688.5669	102154.924	182028.9	149925.4	255333.8	245216.9	343888	534681.5	484541.2	479095.1099
P04945	Ig kappa chain V-VI region NQ2-6.1 (	2	7	1	8.249	23.9	109447.8084	125098.425	96370.87145	258370.4802	1040336	1014582	970139.2	895520.7	2796593	3820986	3606717	1952327.394
P01843	Ig lambda-1 chain C region OS=Mus	1	51	3	0.951	2.443	1502783.902	4417926.634	2288071.262	3926556.643	3441838	1499486	3448206	2182655	7183302	8413984	6780141	5114248.752
P01723	Ig lambda-1 chain V region OS=Mus	1	22	2	2.444	2.702	318907.7422	834461.6997	451795.3923	676152.1954	1803186	650979.1	1087101	1208831	1837001	1616042	1881834	920992.8642
P01844	Ig lambda-2 chain C region OS=Mus	Iglc2	16	3	0.871	1.082	336100.7466	398060.7259	508895.9434	891060.2419	1323575	1174529	654607.8	288746.4	551212.5	1295895	821397	622011.8543
P01872	Immunoglobulin heavy constant mu	Ighm	618	23	1.318	1.154	82833265.19	95066612.18	117890649.5	135371327.2	1.22E+08	1.62E+08	1.42E+08	1.24E+08	1.14E+08	1.36E+08	1.33E+08	82024757.03
P01592	Immunoglobulin J chain OS=Mus mu	Jchain	60	3	0.844	1.031	5519936.25	6536769.153	7858625.1	6265777.557	7242956	4958027	4259901	8625802	5003695	7014683	7738176	4702161.632
P01837	Immunoglobulin kappa constant OS=	Igkc	1263	13	1.227	3.917	16454323.56	20078259.19	20935225.34	20565769.79	40282136	28998432	29255441	36462327	77286959	1.23E+08	1.33E+08	95824788.33
Q9DBD0	Inhibitor of carbonic anhydrase OS=I	Ica	740	41	1.455	1.456	44226177.81	42219399.04	41299172.14	54491287.24	54629872	75913962	79057848	52957894	64484991	66674541	66502760	58339893.52
P70389	Insulin-like growth factor-binding pr	Igfals	36	11	0.166	0.196	3251365.877	2852430.588	2741572.059	2525176.15	337602.7	254041.1	83534.07	326296.7	467227	361215	678796.4	108400.6048
A6X935	Inter alpha-trypsin inhibitor, heavy c	Itih4	1747	50	2.625	1.907	86959635.68	101838811.5	98109023.3	92990312.84	1.98E+08	2.2E+08	2.9E+08	2.07E+08	2E+08	1.94E+08	1.64E+08	155492543.3
Q61702	Inter-alpha-trypsin inhibitor heavy cl	Itih1	418	24	0.78	0.582	22428405.97	25670634.49	29384922.57	28657106.78	17417725	31986927	27807089	19695856	20281221	28532050	26829104	18434395.76
Q61703	Inter-alpha-trypsin inhibitor heavy cl	Itih2	715	27	1.054	1.078	43590510.43	49949490.09	53774467.52	49595488.09	48901394	60217617	54333580	45111670	59717816	66918835	61439572	44737930.07
Q61704	Inter-alpha-trypsin inhibitor heavy cl	Itih3	3158	39	4.582	4.62	41637350.24	51445292.97	44992037.03	45813510.75	1.55E+08	1.46E+08	1.4E+08	1.47E+08	1.77E+08	1.63E+08	1.54E+08	147850065.8
Q61730	Interleukin-1 receptor accessory pro	Il1rap	40	6	0.321	0.305	3555326.618	3596893.973	4755456.971	6086585.436	1782384	1512040	1949909	1747295	1243213	1883888	1817548	1142826.306
Q9Z0M9	Interleukin-18-binding protein OS=M	Il18bp	2	1	100	100					709497.1	969135.2	1357148	895473.2	651940	705431.5	491632.3	389808.6121
P02535	Keratin, type I cytoskeletal 10 OS=M	Krt10	11	3	0.906	0.892	322276.3861	439134.6007	632476.0802	471417.7036	299254.3	150567.1	244151.2	831730.2	325256	383071.4	319593.6	386778.1028
Q922U2	Keratin, type II cytoskeletal 5 OS=Mu	Krt5	10	2	0.636	0.369	75960.69465	579299.4382	338136.9328	968850.5393	216239.5	108352.3	176067.4	517949.3	120425.5	87399.51	167944.8	136416.0838
P61110	Kidney androgen-regulated protein (	Kap	8	1	0.217	0.079	116060.8158	338351.9566	361447.8855	281368.807	42715			75993.01	14365.06	14756.48	24271.57	35488.58618
O08677	Kininogen-1 OS=Mus musculus OX=1	Kng1	1051	33	0.972	1.085	265545985.4	335187627.8	230798020.4	295812881.1	2.87E+08	2.86E+08	3.56E+08	3.28E+08	3.45E+08	3.29E+08	3.49E+08	285652545.5
P42703	Leukemia inhibitory factor receptor	Lifr	164	17	0.161	0.062	11995057.45	17317181.34	16676419.39	19443628.65	2491153	1645842	1250234	2582814	977019	887158.2	711181.3	762989.276

Q61805	Lipopolysaccharide-binding protein (	Lbp	19	7	5.373	6.195	178359.0043	369108.8278	227778.9527	147770.4564	2160402	1276768	1628517	2134886	2295729	2740602	1610627	1582623.271
Q8VCC2	Liver carboxylesterase 1 OS=Mus mu	Ces1	81	1	1.048	1.316	1185741.631	1159140.312	1383830.906	1921990.427	1837513	864694.8	1342430	1676371	1609232	2005048	2452164	1468199.413
P06151	L-lactate dehydrogenase A chain OS	Ldha	66	8	8.87	7.792	467970.3165	75158.54834	175456.4794	257077.3142	4921338	5163982	4891951	4495958	3647249	3258291	3695862	4497391.882
P16125	L-lactate dehydrogenase B chain OS	Ldhb	10	2	2.953	2.175	222728.4054	80977.70983	108809.1786	203952.3337	1494667	436509.3	287450.5	397840.1	241017.7	276696.6	333770	263913.1308
P18337	L-selectin OS=Mus musculus OX=100	Sell	5	3	8.893	2.703		18275.12228		51356.65001	213722	347288.8	342120.2	245169.4	132292.6	131547.5		51827.30308
P51885	Lumican OS=Mus musculus OX=1009	Lum	76	10	0.875	0.48	8239851.945	7827572.506	6847224.866	8237499.057	7529645	5255817	5992458	17465583	7627399	10056049	9017269	7500393.403
P09581	Macrophage colony-stimulating fact	Csf1r	23	3	1.376	1.446	723801.4581	710941.3973	1068760.745	709743.0677	1420140	411431.3	454860.6	1330566	1162166	1218504	1381362	940750.8529
B5X0G2	Major urinary protein 17 OS=Mus m	Mup17	51	1	0.145	0.041	296796.9797	823993.4704	576935.5359	250586.4132	54618.27			66124.96	53333.63	9948.911	12760.54	12923.34706
P11589	Major urinary protein 2 OS=Mus mu	Mup2	44	1	0.129	0.046	8931160.018	12496599.93	8838523.741	4614722.595	937104.2	106696.1	167103.3	1148615	539949.9	106933	296831.7	268178.7885
P14152	Malate dehydrogenase, cytoplasmic	Mdh1	25	4	4.378	2.877	550364.936	27843.75752	339225.6284	542481.4533	2266256	1754654	2081086	1731919	1005284	1424607	1466128	1640865.69
P98064	Mannan-binding lectin serine protea	Masp1	29	5	0.777	0.637	1886336.266	2024165.318	1298459.82	1882206.373	1331225	1380551	1078475	1455141	1503195	1515036	1218721	1080638.924
P39039	Mannose-binding protein A OS=Mus	Mbl1	24	3	0.604	0.36	4089876.283	4394567.488	4702310.756	4126965.02	1965376	3006959	2434708	2157051	1735134	1867280	1727013	1022962.915
P41317	Mannose-binding protein C OS=Mus	Mbl2	27	4	0.294	0.232	4346753.333	4815787.407	4991254.052	4945704.295	835675.1	1073888	299052.9	1184414	1025621	1104869	1252370	732396.2157
P39876	Metalloproteinase inhibitor 3 OS=M	Timp3	2	1	0.121	0.412	248104.1603		271414.7682	118190.2818	28094.66			31499.99		111798.3	98272.85	64708.42578
P10404	MLV-related proviral Env polyprotei	1	15	4	5.117	1.339	592015.0487	529396.3168	430880.5081	421752.6374	1436058	3573113	3332565	1604173	642816.2	483859.8	433055.4	953053.5324
P26041	Moesin OS=Mus musculus OX=1009	Msn	7	2	4.573	3.969	6639.628644				138596.9	159431.4	166399.1	91621.29	139065.9	108158.7	55048.64	136258.0986
B2RPV6	Multimerin-1 OS=Mus musculus OX=	Mmrn1	4	2	0.295	0.278	186320.1064	83859.77689	405068.8948	259334.8292	91553.48	20892.43	13604.94	86597.06	23197.23	91720.51	56410.69	83625.29591
P28665	Murinoglobulin-1 OS=Mus musculus	Mug1	9679	84	0.498	0.575	1177639084	1220004671	1176243079	1192057846	6.31E+08	6.51E+08	5.87E+08	6.27E+08	7.96E+08	6.08E+08	8.21E+08	606836608.2
P04247	Myoglobin OS=Mus musculus OX=10	Mb	33	5	0.745	0.689	314016.439	25988.03125	464227.8474	558742.5652	1027461	230658	280604.7	292314.1	137837.7	316293.7	465220.7	275237.3736
P26645	Myristoylated alanine-rich C-kinase	Marcks	6	2	1.027	0.561	94366.61278	174154.1217	167217.5073	85302.4973	192358.8	28128.51	40038.9	202819.3	66708.52	54934.84	53039.23	118538.5436
Q8VCS0	N-acetylmuramoyl-L-alanine amidas	Pglyrp2	125	8	1.518	1.712	5455345.852	5122678.571	7355745.415	6891331.715	7543598	8564383	9030617	7140234	9775394	9782821	7994801	7784739.667
Q9CR61	NADH dehydrogenase [ubiquinone]	Ndufb7	10	1	0.411	0.839	1050163.095	1589658.388	2071319.949	1055755.758	3090113	56820.14	139319.9	2637899	883237.2	1183064	1527179	1095339.837
P11672	Neutrophil gelatinase-associated lip	Lcn2	25	3	100	100					2915010	861434.9	4236590	2707074	426304.2	375099.6	187921.1	370648.0494
Q01768	Nucleoside diphosphate kinase B OS	Nme2	17	2	9.382	6.787	133599.1615		65654.29422	92932.18085	1151048	895766	846536.5	673977.6	787784.8	898435.4	490824.7	1164260.599
Q64288	Olfactory marker protein OS=Mus m	Omp	2	1	0.088	0.306	1113254.191	1618057.773	1297947.397	1467186.473	419609.7	33123.02	32953.82	460454.5	390019.8	432323.7	433027.9	457095.4565
P10923	Osteopontin OS=Mus musculus OX=	Spp1	5	1	1.068	0.66	59885.77959		176378.6246	162278.3483	127001.2	178052.4	183398.7	167813.9	87494.68	115532.1	65410.1	108041.6041
P35700	Peroxiredoxin-1 OS=Mus musculus (	Prdx1	5	1	2.693	1.801	60946.65423				228232.6		55774.98	164111.7	40112.1	78217.68	154070.8	201974.6302
Q61171	Peroxiredoxin-2 OS=Mus musculus (	Prdx2	18	3	3.655	2.886	2471048.362	223589.9952	853004.9283	220626.055	3064236	1380639	1839968	2901411	1550722	1660546	2472266	4861483.406
P16301	Phosphatidylcholine-sterol acyltrans	Lcat	122	9	1.467	1.548	50294286.03	54753341.91	75638920.19	58685495.16	58035047	68669264	62299419	56513741	63214353	54384244	53150164	46911373.64
O70362	Phosphatidylinositol-glycan-specific	Gpld1	355	20	0.716	1.056	42432291.5	38386428.15	37232118.41	34320643.65	27715016	31412277	26112436	26209591	40482393	43592379	44475372	34443964.48
Q9D0F9	Phosphoglucomutase-1 OS=Mus mu	Pgm1	5	1	2.877	1.425	31199.83579		49299.54496	61162.94548	118633.9	182175.4	169973.7	81668.62	43980.94	43718.97	147704.8	88127.35803
P09411	Phosphoglycerate kinase 1 OS=Mus	Pgk1	6	2	4.688	2.33	45098.12088			49095.73781	354810.6	598328.4	543094.9	175739.4	46375.28	28456.09	387907.6	167736.4782
P55065	Phospholipid transfer protein OS=M	Pltp	25	5	0.929	0.829	2084204.768	2326974.758	3235442.876	3013950.227	2081133	971243.1	831966.1	1802917	1892748	2060377	2545685	1624360.83
P26262	Plasma kallikrein OS=Mus musculus	Klkb1	182	15	0.387	0.46	17810758.44	21515240.43	19206551.6	22550325.42	8371571	6087815	5511939	8096547	9236063	8602885	9008262	8216415.763
P97290	Plasma protease C1 inhibitor OS=Mu	Serping1	605	20	2.013	2.052	36029125.62	38659405.66	37482350.76	33192733.6	99616405	51296603	59715348	88935057	77480100	83982412	71577741	65148108.97
P20918	Plasminogen OS=Mus musculus OX=	Plg	1378	51	0.671	0.661	191677833.2	211148816.6	204359507.2	236018428.2	1.29E+08	1.75E+08	1.86E+08	1.31E+08	1.17E+08	1.39E+08	1.43E+08	124636032.7
Q61233	Plastin-2 OS=Mus musculus OX=100	Lcp1	12	4	3.458	1.313	134376.6534	207723.869	104895.0659	140985.5642	603816.2	797478.9	667986.4	812241.5	500520.7	263792.1	361367.4	244400.9109
Q9Z126	Platelet factor 4 OS=Mus musculus (	Pf4	25	2	0.074	0.433	9558736.745	174287.9209	6869362.801	2753741.304	848283.9	126114.5	148384.7	799216.4	542080.4	2739956	2407521	2608080.091
O35930	Platelet glycoprotein Ib alpha chain (	Gp1ba	16	3	0.327	0.378	725205.543	711863.6973	1346731.699	969504.1102	388895.6	160351.6	96044.78	290541.9	369152.1	462432.1	365328.8	481699.1129
O08742	Platelet glycoprotein V OS=Mus mus	Gp5	5	1	0.124	0.185	80769.31768		244459.6311	153374.0098	22666.43			15893.55	17060.98	24906.26	20064.18	56334.30325
Q60963	Platelet-activating factor acetylhydro	Pla2g7	94	10	6.376	8.979	793057.7789	1002284.372	858376.4697	815293.2532	7267360	3864773	4067621	6439063	10484264	9147776	7353147	9566379.169
Q61838	Pregnancy zone protein OS=Mus mu	Pzp	17874	98	0.707	0.727	2684978067	3171617132	3246471729	3037339152	2.48E+09	2.11E+09	1.64E+09	2.37E+09	2.65E+09	2.46E+09	2.37E+09	2218122925
Q9CQF9	Prenylcysteine oxidase OS=Mus mus	Pcyox1	1	1														
P28798	Progranulin OS=Mus musculus OX=1	Grn	24	4	2.695	1.274	139340.209	246935.7746	139661.3152	166438.3151	567073.7	591329.2	389786.7	610502.7	248460.7	343314.3	220987.6	410893.3001
P11680	Properdin OS=Mus musculus OX=10	Cfp	131	7	0.31	0.275	11518367.95	22198621.57	18391227.99	16803038.96	6909908	4319108	3921300	5308456	4139149	4646549	4598124	4043897.832
Q61207	Prosaposin OS=Mus musculus OX=1	Psap	4	2	0.854	0.01	30866.51662	99634.73238	69619.87999	137389.3478	51965.92	363903.4	128832.6	90584.52				
Q9R1P4	Proteasome subunit alpha type-1 OS	Psma1	7	2	2.132	3.851	29596.3655	94086.29946	52745.6697	151224.0146	132376.7	334706.6	251116	205539.9	534504	587525.5	240636.7	364408.1238
Q9R1P0	Proteasome subunit alpha type-4 OS	Psma4	1	1	5.462	8.735		22999.39977	15437.23402		125772.9		41246.12	125492.4	215722.3	187078.4		123231.3993
Q9Z2U1	Proteasome subunit alpha type-5 OS	Psma5	4	2	4.933	5.743	98936.59563	99538.85013	111261.0851	88940.97602	324879.4	555938.9	436155.6	355459.6	536551.2	571423.8	407956.8	618548.6618
Q9QUM9	Proteasome subunit alpha type-6 OS	Psma6	12	3	5.109	7.147	205365.8334	183739.8687	207551.8456	143977.6961	874265.9	947109.6	710690.2	972847.9	1443717	1518392	1031096	1123058.313
Q9Z2U0	Proteasome subunit alpha type-7 OS	Psma7	10	3	1.791	3.202	83579.17531	83899.59605	286055.5968	308000.3146	517494	182225.4	6526.934	329345	749108.1	807981.4	354212.6	620199.5654
O09061	Proteasome subunit beta type-1 OS	Psmb1	5	3	4.817	5.859	103262.9967		68191.2041		339685	198125.5	146509.8	358441.2	375926.9	824877.5	87612.58	522925.1925
P99026	Proteasome subunit beta type-4 OS	Psmb4	3	2	2.948	6.392	39583.50451		44996.21244		234922.7	91247.77	82478.24	169648.2	355708.7	287308.7	207951.1	253317.6841
O55234	Proteasome subunit beta type-5 OS	Psmb5	3	1	2.828	5.184	38489.74124	31209.79571	25351.67422		87371.2			89130.9	169635.4	167992.2	144127.4	155807.9393
Q60692	Proteasome subunit beta type-6 OS	Psmb6	2	1	2.575	3.673	73992.50799	77054.56899	56140.75964	55681.05923	171929.7	247639.3	145964.3	142014.6	244376	213519.9	220226.8	262435.9634
Q07456	Protein AMBP OS=Mus musculus OX	Ambp	216	10	3.201	3.439	19729133.51	21999998.56	16667635.85	18511276.1	48581924	66890189	66630930	44257885	69191480	74488758	62390572	58604698.35
Q8R121	Protein Z-dependent protease inhibi	Serpina10	201	19	1.412	0.921	6646767.175	9305471.404	8786380.798	8850530.989	11321908	12607834	13105164	11401692	8558939	8323264	8529344	5531479.665
Q9JM99	Proteoglycan 4 OS=Mus musculus O	Prg4	6	2	5.65	10.986			29616.6995		434282.4	280773.8	223822	727414.3	406512.1	476952.9	282804.4	426596.1404
P19221	Prothrombin OS=Mus musculus OX=	F2	662	34	0.646	0.706	72802649.8	89132295.54	75558251.97	69549719.89	54731606	44232913	50567270	56646718	76732884	49696843	49758217	45623461.18
P52480	Pyruvate kinase PKM OS=Mus muscu	Pkm	34	10	4.245	3.56	262380.7941	162214.0255	301028.6849	657211.16	2810106	3923586	3261260	2107378	1893322	2090576	1515589	1972032.785
Q00724	Retinol-binding protein 4 OS=Mus m	Rbp4	86	5	0.79	0.555	3430428.732	3656012.837	4749806.716	5612386.176	7290421	2699861	4194839	2601366	3229402	2084269	2420071	2492170.873
Q8K1I3	Secreted phosphoprotein 24 OS=Mu	Spp2	29	3	0.617	0.722	2141166.435	2193383.83	1767504.663	2615397.605	1255377	1298068	1208148	1414249	1584977	1792730	1238592	1390216.183
P70274	Selenoprotein P OS=Mus musculus (	Selenop	177	8	0.469	0.463	4557362.767	7000839.268	7919361.744	7249945.113	5280349	1036622	2088362	4246228	3698383	3240891	3849713	2812677.548
Q5I2A0	Serine protease inhibitor A3G OS=M	Serpina3g	400	1	100	100					80573.33		63450.75	60293.91		42515.45	31385.72	48691.82824
P07759	Serine protease inhibitor A3K OS=M	Serpina3k	18858	28	0.861	0.754	1340222829	1337395784	1515173204	1756090195	1.05E+09	1.2E+09	1.25E+09	1.2E+09	1.3E+09	1.06E+09	1.09E+09	1013970734

Q03734	Serine protease inhibitor A3M OS=M	Serpina3m	6791	9	3.282	2.795	2056904.9	2207924.84	2125818.382	1750080.635	8268142	5047596	5807971	7336474	6438836	5561715	7006902	6106682.165
Q91WP6	Serine protease inhibitor A3N OS=M	Serpina3n	3250	18	9.688	6.474	13565655.87	12521960.29	11356646.95	10380789.99	91998250	76903594	1.22E+08	92932534	80000264	58460643	55197576	74907382.39
Q921I1	Serotransferrin OS=Mus musculus O	Tf	6660	81	1.132	0.943	2745125691	2620965481	2515522518	2637736893	2.59E+09	3.46E+09	3.35E+09	2.59E+09	2.61E+09	2.83E+09	2.66E+09	2601907656
P07724	Serum albumin OS=Mus musculus O	Alb	11823	76	0.602	0.552	3540250647	3857320772	3820362810	4241305197	2.68E+09	2.76E+09	2.72E+09	2.77E+09	2.53E+09	2.81E+09	2.69E+09	2721695790
P05366	Serum amyloid A-1 protein OS=Mus	Saa1	322	12	100	46.755	1237298.431	1188705.634	754433.12	1306595.226	1.27E+08	1.86E+08	1.97E+08	1.5E+08	87650605	74852770	21184251	119246237.5
P05367	Serum amyloid A-2 protein OS=Mus	Saa2	264	14	23.723	3.068	14070.35623	70259.40422	25401.76547	67242.5807	1.43E+08	1.91E+08	1.91E+08	1.72E+08	51908372	39632736	16092797	73903990.19
P04918	Serum amyloid A-3 protein OS=Mus	Saa3	22	2	100	100					691804.7	397121.1	368899.8	820637.7	230384.1	278395.3	224866.3	344655.2254
P31532	Serum amyloid A-4 protein OS=Mus	Saa4	129	10	1.434	1.674	7936968.382	8835039.365	8140406.079	6098117.565	17079354	9124496	6396544	10437554	10569829	15662303	7709001	12105755.99
P12246	Serum amyloid P-component OS=M	Apcs	651	14	6.202	5.088	24791551.42	26853883.98	17429068.85	15745190.89	1.18E+08	1.37E+08	1.39E+08	1.2E+08	1.4E+08	1.2E+08	1.08E+08	93831339.61
P52430	Serum paraoxonase/arylesterase 1 (	Pon1	233	10	0.29	0.524	26806856.94	27058717.66	28519406.43	29333662.27	10011307	6357838	6040866	7981786	11742260	16020868	22914138	8125097.376
P70663	SPARC-like protein 1 OS=Mus muscu	Sparcl1	48	6	0.754	0.77	375091.0567	954728.0521	579675.9424	623374.1064	373791.8	354930	463511.7	637236.2	503728	514542.4	411343.6	481584.9484
Q8BND5	Sulfhydryl oxidase 1 OS=Mus muscu	Qsox1	200	19	2.439	2.087	15262562.78	15238077.48	15342991.61	17963129.98	24014850	18032461	21618630	21239500	23561927	23671552	21625085	16703762.34
P08228	Superoxide dismutase [Cu-Zn] OS=M	Sod1	10	2	0.731	1.043	202574.0971	69181.23124	122112.8854	113401.2715	202073.1	254844.5	205110.7	155787.8	63980.91	95630.14	211659	210923.665
P26039	Talin-1 OS=Mus musculus OX=10090	Tln1	1	1	0.598	0.768	60827.70933	33235.57013		117679.2824	46625.62			28414.06	23599.95	57026.74	50548.43	62136.79752
P06332	T-cell surface glycoprotein CD4 OS=I	Cd4	3	1	5.1	15.849	17026.79071	38502.80352	21802.42776	20339.59977	116412			99096.16	333745.2	449378.5	467007.6	198775.4825
P43025	Tetranectin OS=Mus musculus OX=1	Clec3b	2	1	0.526	0.01		385752.3916	226051.9788		155305							
P35441	Thrombospondin-1 OS=Mus muscul	Thbs1	160	23	0.15	0.507	21264107.77	412715.7316	27040017.16	18017203.35	3146823	1189288	2203234	3718584	2459741	15026486	8100733	14329297.68
Q9Z1T2	Thrombospondin-4 OS=Mus muscul	Thbs4	2	1	0.533	0.343	153150.8382	149192.3852	234159.0245	413585.4329	103717.7			98371.22	76142.37	76170.57	62029.64	55423.52757
P61939	Thyroxine-binding globulin OS=Mus	Serpina7	6	1	0.486	0.461	143793.8679	184913.9131	441171.098	551996.9663	211825.5	70266.25	164640.1	266636.6	106425	140070.1	163113.8	113570.1071
P82198	Transforming growth factor-beta-inc	Tgfbi	3	2	2.454	3.513	54285.51552	66244.23971	46579.34181	40203.55773	133767.8	161899.1	91199.1	119735.5	180513.2	206261.2	338952.2	129469.9467
P40142	Transketolase OS=Mus musculus OX	Tkt	8	3	3.202	1.134			14753.20547		355056.7	634166.1	573191.9	311672.2	72812.52	205018.1	82692.29	231643.8143
P07309	Transthyretin OS=Mus musculus OX=	Ttr	723	9	0.725	0.446	271130676	253554480.6	326787628.2	250823354.8	1.81E+08	1.54E+08	1.56E+08	2.13E+08	1.28E+08	1.15E+08	1.56E+08	136638566.5
P17751	Triosephosphate isomerase OS=Mus	Tpi1	12	4	5.128	1.926	51077.89667		35995.40471	134181.5173	941199.3	1740011	1139796	899827.8	73424.48	175693.8	719481	229855.4147
Q7TMM9	Tubulin beta-2A chain OS=Mus musc	Tubb2a	1	1	2.35	1.613	53776.73514	42335.05796	59962.85533	56003.34348	120065.3	159824.4	128976.3	112401.5	80900.89	88067.69	143661.5	85411.76027
P62984	Ubiquitin-60S ribosomal protein L40	Uba52	5	3	4.357	2.946	333231.7343	66472.52665	158623.7471	17423.20912	781002.5			524688	385614.8	378638	565114.1	972130.4587
P29533	Vascular cell adhesion protein 1 OS=	Vcam1	12	4	4.746	3.802	2564056.472	3681057.378	2619005.107	3511673.468	3162483	1505082	1587022	2175554	2055234	1899601	2861590	1662404.398
P21614	Vitamin D-binding protein OS=Mus r	Gc	857	36	0.838	0.778	73287900.71	94701617.53	93620441.6	97409215.8	69754114	1E+08	85132221	63001624	61682934	74965758	57119826	71542132.65
Q9CQW3	Vitamin K-dependent protein Z OS=I	Proz	54	6	0.158	0.067	2866647.28	5066706.821	4567244.097	4317889.753	632837.1	355721	173540	779713.9	432756.3	138720.9	263539.1	209506.1693
P29788	Vitronectin OS=Mus musculus OX=1(	Vtn	377	12	1.191	0.855	41126842.85	48249387.21	44655581.58	46209789.6	53531085	57581641	48853339	45370674	38683998	38833939	38156017	31402840.1
Q64726	Zinc-alpha-2-glycoprotein OS=Mus n	Azgp1	288	17	1.405	1.454	15723882.06	16642768.97	17941678.33	20754143.3	21544780	28058747	32107151	27720284	27457369	25220324	25521905	23494619.77

Supplementary Table 4: compiled list of serum proteins identifiedand quantified using the three approachs in the conditions control (CTRL), 7dpi and 9dpi.

Accession	Description	Gene name	Abundance Ratio (LOG2): (7dpi) / (CTRL)	Abundance Ratio (LOG2): (9dpi) / (CTRL)	Abundances (Normalized): F1: Sample, CTRL	Abundances (Normalized): F2: Sample, CTRL	Abundances (Normalized): F3: Sample, CTRL	Abundances (Normalized): F4: Sample, CTRL	Abundances (Normalized): F5: Sample, 7dpi	Abundances (Normalized): F6: Sample, 7dpi	Abundances (Normalized): F7: Sample, 7dpi	Abundances (Normalized): F8: Sample, 7dpi	Abundances (Normalized): F9: Sample, 9dpi	Abundances (Normalized): F10: Sample, 9dpi	Abundances (Normalized): F11: Sample, 9dpi	Abundances (Normalized): F12: Sample, 9dpi	Method
P10404	MLV-related proviral Env polyprotein OS=Mus musculus OX=10090 PE=1 SV=3	1	2.23	0.89	183108.2268	230636.9085	1382905.116	147698.2681	675853.1593	350195.5	1237243.312	457967.935	24652.17859	40242.28916	68683.75457	336982.3175	DTT
P10404	MLV-related proviral Env polyprotein OS=Mus musculus OX=10090 PE=1 SV=3	1	2.08	0.33	647727.1172	280701.239	513750.1778	524001.494	1101348.005	1623772.485	1158896.44	1605367.064	620831.1941	611596.3462	465596.2044	753249.4967	DD
Q6GQT1	Alpha-2-macroglobulin-P OS=Mus musculus OX=10090 GN=A2m PE=2 SV=2	A2m	-1.43	-1.20	687983.8273	1037435.447	917070.633	462227.9094	231624.5977	97840.67349	705258.1891	339499.7038	577441.9351	917936.0741	141185.8072	277427.0619	TCA
Q6GQT1	Alpha-2-macroglobulin-P OS=Mus musculus OX=10090 GN=A2m PE=2 SV=2	A2m	-0.99	0.08	183436.6152	316661.9107	293525.4181	175913.2081	78644.75153	77648.81573	111195.8508	528767.8361	233338.7489	418586.4339		33253.71635	DD
P68134	Actin, alpha skeletal muscle OS=Mus musculus OX=10090 GN=Acta1 PE=1 SV=1	Acta1	2.96	2.86	1300325.183	812013.3209	841048.7799	1867121.559	11018072.59	8258951.762	11673738.56	13344616.7	8688236.212	11025471.61	6345321.838	11766290.83	TCA
P68134	Actin, alpha skeletal muscle OS=Mus musculus OX=10090 GN=Acta1 PE=1 SV=1	Acta1	2.83	2.49	308708.2778	210066.3728	290936.3207	440953.4108	2513359.87	1853442.742	2688100.963	2673365.076	1530695.49	1860560.943	2128434.473	1440738.276	DTT
P68134	Actin, alpha skeletal muscle OS=Mus musculus OX=10090 GN=Acta1 PE=1 SV=1	Acta1	6.10	5.57	28914.98633	122722.0727		176143.8327	5297135.001	10451491.72	7845191.507	9086485.031	5302339.029	6405241.767	3971733.191	6899359.438	DD
P60710	Actin, cytoplasmic 1 OS=Mus musculus OX=10090 GN=Actb PE=1 SV=1	Actb	3.64	3.17	12721618.98	5836705.14	7106081.13	11144257.18	98489541.95	145051784.6	140774287.6	92214808.6	85025078.35	99031461.17	64809807.44	96195635.93	TCA
P60710	Actin, cytoplasmic 1 OS=Mus musculus OX=10090 GN=Actb PE=1 SV=1	Actb	3.45	2.01	3103590.545	2713883.791	3559309.623	4259680.322	30265289.1	30635171.8	50126670.45	19209103.37	12635610.49	12504036.81	11821939.89	7228691.407	DTT
P60710	Actin, cytoplasmic 1 OS=Mus musculus OX=10090 GN=Actb PE=1 SV=1	Actb	3.62	3.07	6435612.229	3615196.92	3572337.836	5420398.016	61434086.02	82901806.74	58398338.79	74806000.38	49271819.35	56765371.6	34688825.88	70434293.96	DD
Q60994	Adiponectin OS=Mus musculus OX=10090 GN=Adipoq PE=1 SV=2	Adipoq	-1.55	-0.78	1597866.466	2158416.233	2401365.648	2447250.245	1682501.219	326394.0784	339466.0222	932925.9393	940044.5	1506223.925	1187518.63	819922.2648	TCA
O89020	Afamin OS=Mus musculus OX=10090 GN=Afm PE=1 SV=2	Afm	-0.76	-1.76	22597990.88	25959418.34	20776601.59	12571078.85	7767215.921	25373911.2	8928416.938	13325730.56	5104741.395	7430736.107	7064629.832	5805272.228	TCA
O89020	Afamin OS=Mus musculus OX=10090 GN=Afm PE=1 SV=2	Afm	-1.94	-3.05	3071964.79	5292499.049	3539620.271	5311718.334	567731.8675	755132.3477	936954.4274	580225.8223	128331.6499	251389.9154	436925.301	182662.9778	DTT
O89020	Afamin OS=Mus musculus OX=10090 GN=Afm PE=1 SV=2	Afm	-1.18	-1.74	34317617.57	35999746	37708679.7	33989085.09	16941343.72	18375308.14	14685395.49	15279644.63	9373985.881	12965122.38	12485448.88	11229629.65	DD
P11859	Angiotensinogen OS=Mus musculus OX=10090 GN=Agt PE=1 SV=1	Agt	0.41	0.61	3588472.878	3687680.406	3899753.113	3357519.024	4771313.539	2874889.657	5696900.583	4918878.008	6947878.14	6072992.308	6314400.65	5159763.348	TCA
P11859	Angiotensinogen OS=Mus musculus OX=10090 GN=Agt PE=1 SV=1	Agt	0.60	0.22	1502299.624	2030088.452	1031046.434	3316570.02	2858040.194	2439048.469	5517312.539	1268228.218	3054511.42	1843522.609	1438967.034	2544586.402	DTT
P11859	Angiotensinogen OS=Mus musculus OX=10090 GN=Agt PE=1 SV=1	Agt	0.68	0.57	2672901.82	3027763.966	2794248.998	3068194.87	4019909.411	4450530.569	3723267.273	6421505.501	4984247.864	3908395.26	3608629.105	3377382.491	DD
P29699	Alpha-2-HS-glycoprotein OS=Mus musculus OX=10090 GN=Ahsg PE=1 SV=1	Ahsg	-0.02	-0.34	400969212.9	427298052.8	349027492.6	335225533	299322060.3	533707273.9	479134321.1	331009463.7	294576518	314773521.6	327239269.5	341915262.4	TCA
P29699	Alpha-2-HS-glycoprotein OS=Mus musculus OX=10090 GN=Ahsg PE=1 SV=1	Ahsg	0.72	0.53	324187787.2	309455287.9	208294353.4	220445998	295695919	377478732.7	338079505.3	414135535.6	395623825.5	336425244.1	306417264.3	408870600.6	DTT
P29699	Alpha-2-HS-glycoprotein OS=Mus musculus OX=10090 GN=Ahsg PE=1 SV=1	Ahsg	0.26	0.28	227788522.1	244891248.3	207500744.1	192052210.8	202875622.7	245470994.1	224157255.3	240602531.8	252515374.3	231797459	244931323.3	266798007.8	DD
Q9R0Y5	Adenylate kinase isoenzyme 1 OS=Mus musculus OX=10090 GN=Ak1 PE=1 SV=1	Ak1	3.55	2.26		16529.04186			138454.1094	271630.3064	280382.9318	77689.30977	50557.27834		180568.3939	79341.02896	TCA
Q9R0Y5	Adenylate kinase isoenzyme 1 OS=Mus musculus OX=10090 GN=Ak1 PE=1 SV=1	Ak1	0.87	0.42	61538.10938				91408.83327	241376.0816	59135.55954	138705.4827	24437.72362		119561.0485	82259.55301	DD
P10518	Delta-aminolevulinic acid dehydratase OS=Mus musculus OX=10090 GN=Alad PE=1 SV=1	Alad	6.64	#VALUE!					43968.83941	64256.71094	38931.37122	16408.24572					DTT
P07724	Serum albumin OS=Mus musculus OX=10090 GN=Alb PE=1 SV=3	Alb	-0.73	-0.86	3540250647	3857320772	3820362810	4241305197	2676367229	2755284690	2724268276	2772666284	2532301644	2812950799	2688821902	2721695790	TCA
P07724	Serum albumin OS=Mus musculus OX=10090 GN=Alb PE=1 SV=3	Alb	-0.87	-1.03	2373033744	2745072796	2354401427	2872340203	1724267461	1740391586	2556611587	1272015523	1175471223	1903201536	2524581370	426003158.6	DTT
P07724	Serum albumin OS=Mus musculus OX=10090 GN=Alb PE=1 SV=3	Alb	-0.30	-0.25	9740703554	10718702761	10847554410	11075203438	9383542396	8620893924	8459157346	9468039650	9140567834	9265994471	9297634337	8455899698	DD
P05064	Fructose-bisphosphate aldolase A OS=Mus musculus OX=10090 GN=Aldoa PE=1 SV=2	Aldoa	2.36	1.26	1232241.884	300409.4157	755404.0808	1068847.328	2818924.688	11265591.68	11089445.72	3501034.728	1490266.723	1339440.732	3829120.752	2492066.641	TCA
P05064	Fructose-bisphosphate aldolase A OS=Mus musculus OX=10090 GN=Aldoa PE=1 SV=2	Aldoa	6.64	6.64					13651.62181		53736.4443		18427.60232	57193.81639	221985.8977		DTT
P05064	Fructose-bisphosphate aldolase A OS=Mus musculus OX=10090 GN=Aldoa PE=1 SV=2	Aldoa	2.40	1.50	449698.75	28566.75041	199182.31	163885.5928	1426449.01	3269672.518	1480461.086	2214550.786	1593725.567	1308863.781	2183324.724	1046684.002	DD
Q91Y97	Fructose-bisphosphate aldolase B OS=Mus musculus OX=10090 GN=Aldob PE=1 SV=3	Aldob	1.85	2.51			26022.71816		93917.33594		56912.39646	222006.3454	133318.5954	165153.8727	52268.92016	212083.006	TCA
Q07456	Protein AMBP OS=Mus musculus OX=10090 GN=Ambp PE=1 SV=2	Ambp	1.68	1.78	19729133.51	21999998.56	16667635.85	18511276.1	48581924.02	66890189.44	66630929.85	44257885.21	69191480.49	74488757.81	62390572.06	58604698.35	TCA
Q07456	Protein AMBP OS=Mus musculus OX=10090 GN=Ambp PE=1 SV=2	Ambp	1.89	1.38	1686267.48	3287915.013	1529242.211	3695300.14	7074134.657	4338938.729	14198787.36	8445874.185	6727124.31	7156415.351	9203163.641	2079889.708	DTT
Q07456	Protein AMBP OS=Mus musculus OX=10090 GN=Ambp PE=1 SV=2	Ambp	1.44	1.63	17817273.8	22290144.43	19322955.48	18272193.87	50357102.88	54507355.27	53003790.02	52000423.5	65371221.49	65476516.84	60871795.9	60197585.15	DD
P00687	Alpha-amylase 1 OS=Mus musculus OX=10090 GN=Amy1 PE=1 SV=2	Amy1	0.97	0.80	451700.5448	417857.637	587089.6477	520770.2592	653170.1641	409000.63	1236034.738	566591.1466	2188629.052	789394.0443	489564.8689	618464.8997	TCA
P00687	Alpha-amylase 1 OS=Mus musculus OX=10090 GN=Amy1 PE=1 SV=2	Amy1	0.56	-0.83	37564.86895	129151.7214	48505.05022	100999.1059			333236.741	31743.74941	202212.1875	16290.43403		35503.3319	DTT
P00687	Alpha-amylase 1 OS=Mus musculus OX=10090 GN=Amy1 PE=1 SV=2	Amy1	0.61	0.46	223758.1875	234806.6154	249272.1787	216451.8991	269187.2413	164302.9085	186708.9001	647323.7129	909345.2978	367055.9828	232617.3541	264622.2183	DD
Q02357	Ankyrin-1 OS=Mus musculus OX=10090 GN=Ank1 PE=1 SV=2	Ank1	6.64	6.64						4527.237262	8014.480306					66623.04716	DD
P12246	Serum amyloid P-component OS=Mus musculus OX=10090 GN=Apcs PE=1 SV=2	Apcs	2.63	2.35	24791551.42	26853883.98	17429068.85	15745190.89	118449466.6	136642896.6	138865008.9	119732301.3	140229620.1	120115211.5	107991085.1	93831339.61	TCA
P12246	Serum amyloid P-component OS=Mus musculus OX=10090 GN=Apcs PE=1 SV=2	Apcs	1.74	1.15	2446886.916	6591351.036	1776596.092	3554154.656	7108117.642	12309220.43	25532093.47	8444430.495	6809614.398	9198042.532	14014482.5	1712428.88	DTT
P12246	Serum amyloid P-component OS=Mus musculus OX=10090 GN=Apcs PE=1 SV=2	Apcs	2.78	2.52	17145999.26	19787925.87	12638036.02	12167062.88	79254297.28	99218116	88000690.83	96792387.11	101560589.9	91687624.74	74215011.56	78073130.2	DD
Q00623	Apolipoprotein A-I OS=Mus musculus OX=10090 GN=Apoa1 PE=1 SV=2	Apoa1	-0.79	-0.61	756712955.9	839139536.8	764367641.5	906068271.6	657812757.7	493658808.6	455768940.9	475002358	831680125.7	823602017.2	535635788.2	682393979	TCA
Q00623	Apolipoprotein A-I OS=Mus musculus OX=10090 GN=Apoa1 PE=1 SV=2	Apoa1	-0.22	0.11	940054528.4	904985391.4	799706218.8	758745756.5	874136361.8	584314378.9	545574112.3	723807246.2	1036475879	813490366.4	691906360.6	918243210.4	DTT
Q00623	Apolipoprotein A-I OS=Mus musculus OX=10090 GN=Apoa1 PE=1 SV=2	Apoa1	-0.46	-0.01	1408570363	1481638380	1481863063	1375446593	1250265554	1068899128	1117671638	1002700051	1452390421	1449501055	1333945044	1154303975	DD
P09813	Apolipoprotein A-II OS=Mus musculus OX=10090 GN=Apoa2 PE=1 SV=2	Apoa2	-2.17	-2.87	13580965.43	19647281.8	24568178.29	23108732.54	9195481.495	14706261.8	5867400.101	11497346.03	4309490.74	2499791.948	6341659.903	3485030.428	TCA
P09813	Apolipoprotein A-II OS=Mus musculus OX=10090 GN=Apoa2 PE=1 SV=2	Apoa2	-0.25	-0.21	385543128.1	374517362.2	646128480.1	313462569.9	165372057.3	194104819.2	97495864.07	204404358.7	177615635.9	210948359.4	185612289.5	283378099.7	DTT
P09813	Apolipoprotein A-II OS=Mus musculus OX=10090 GN=Apoa2 PE=1 SV=2	Apoa2	-1.28	-1.47	136380135.1	138133107.3	137013911.6	123769081.2	61162544.7	45683890.2	53089994.28	50783406.2	42819088.22	47918993.84	33035564.26	11979373.57	DD
P06728	Apolipoprotein A-IV OS=Mus musculus OX=10090 GN=Apoa4 PE=1 SV=3	Apoa4	-0.22	1.06	93178884.32	96519928.77	93347436.9	108231848	101435250.8	61158239.02	65193674.67	87275411.09	229757522.5	203947463.5	82000789.16	194106041.6	TCA
P06728	Apolipoprotein A-IV OS=Mus musculus OX=10090 GN=Apoa4 PE=1 SV=3	Apoa4	0.11	1.40	602487645.2	559798379.5	792905861	501410019	741180481.6	429655058.3	465551802.7	935774799.3	1975746470	1492113014	759362189.1	2029972117	DTT
P06728	Apolipoprotein A-IV OS=Mus musculus OX=10090 GN=Apoa4 PE=1 SV=3	Apoa4	0.11	1.26	188590001.7	177016326.8	178706098.4	153393550.8	212980685.6	160989117.8	232527629.8	166037029.8	490486031.8	393954861.5	251040240.9	444959655.9	DD
Q8C7G5	Apolipoprotein A-V OS=Mus musculus OX=10090 GN=Apoa5 PE=1 SV=1	Apoa5	6.64	6.64					265332.8149	480675.7577	490512.9687	274453.5147	338531.7242	317760.4517		190101.37	DD
E9Q414	Apolipoprotein B-100 OS=Mus musculus OX=10090 GN=Apob PE=1 SV=1	Apob	0.41	0.62	46776465.64	44529504.02	39775402	39266244.47	62900922.79	57718517.76	43634452.96	67634092.82	69846469.05	77485383.76	49974253.64	55028234.63	TCA
E9Q414	Apolipoprotein B-100 OS=Mus musculus OX=10090 GN=Apob PE=1 SV=1	Apob	1.27	0.46	568356.0984	305660.2393	402448.2165	295532.2993	419109.3593	233378.9297	690065.9304	181086.5379	372609.2762	1582305.192	1470693.457	69365.90313	DTT
E9Q414	Apolipoprotein B-100 OS=Mus musculus OX=10090 GN=Apob PE=1 SV=1	Apob	0.78	0.68	87890108.56	89307534.56	69149020.01	73918217.77	74203943.22	82818543.11	89944082.29	64685742.88	76101792.14	79783802.54	59184805.06	58158167.17	DD
P34928	Apolipoprotein C-I OS=Mus musculus OX=10090 GN=Apoc1 PE=1 SV=1	Apoc1	-1.66	-0.99	2374338.238	2324061.308	1650522.616	479533.4004	1111317.891	189796.0751	73993.15837	416776.2129	1532256.665	1963370.433	1003096.869	668109.6135	TCA
P34928	Apolipoprotein C-I OS=Mus musculus OX=10090 GN=Apoc1 PE=1 SV=1	Apoc1	-2.54	-1.75	13922152.39	12949391.63	15907166.68	8752259.677	4149079.682	525596.2012	2117934.043	1996019.982	3886312.694	4374425.205	3958246.107	1942671.486	DTT
P34928	Apolipoprotein C-I OS=Mus musculus OX=10090 GN=Apoc1 PE=1 SV=1	Apoc1	-0.37	-0.77	12239038.91	7716930.842	6790520.729	6135500.218	4248003.58	5820389.621	4764523.877	3411429.61	2928313.274	4917073.361	3717058.533	3128373.509	DD
Q05020	Apolipoprotein C-II OS=Mus musculus OX=10090 GN=Apoc2 PE=2 SV=1	Apoc2	-3.34	-2.78	4227675.106	4517572.382	5279057.458	4633644.294	731211.1953	407605.7476	459155.6696	466125.1718	1159824.598	1059780.075	658199.2554	612607.8969	TCA
Q05020	Apolipoprotein C-II OS=Mus musculus OX=10090 GN=Apoc2 PE=2 SV=1	Apoc2	-1.63	-1.11	51957981.09	44647029.93	69331235.39	29865697.44	13208086.67	13799639.55	5227388.112	17374443.53	19884688.45	18510512.22	13229700.16	29182957.92	DTT
Q05020	Apolipoprotein C-II OS=Mus musculus OX=10090 GN=Apoc2 PE=2 SV=1	Apoc2	-3.21	-2.19	6187958.57	4397269.875	4754837.359	3922689.367	465058.4893	277970.9514	747921.9705	621814.3402	1448807.536	679774.9842	1506761.542	663727.8759	DD
P33622	Apolipoprotein C-III OS=Mus musculus OX=10090 GN=Apoc3 PE=1 SV=2	Apoc3	-1.65	-1.64	79293542.07	81020573.69	68360316.6	90495748.15	27137966.99	45181654.34	24707390.39	22978842.41	30549698.52	30116438.35	24152573.62	24993198.92	TCA
P33622	Apolipoprotein C-III OS=Mus musculus OX=10090 GN=Apoc3 PE=1 SV=2	Apoc3	-1.65	-1.12	409429549.6	451967462.5	679804884.3	362430798	149833321.2	165258804.7	117990300.3	258163303.1	289697191	248585706.3	168886860.4	285424196.2	DTT
P33622	Apolipoprotein C-III OS=Mus musculus OX=10090 GN=Apoc3 PE=1 SV=2	Apoc3	-1.57	-1.65	69007220.78	62267417.91	63589707.18	62041842.01	22108968.48	28292645.31	21066167.09	17980008.18	20060117.71	22096774.14	18806205.44	22601369.77	DD
Q61268	Apolipoprotein C-IV OS=Mus musculus OX=10090 GN=Apoc4 PE=1 SV=1	Apoc4	-1.54	-1.28	2401254.842	2206973.266	1685462.696	1689027.995	641605.4727	910230.9072	335612.5874	660766.5843	1088030.551	1228086.07	459561.1447	611129.0043	TCA
Q61268	Apolipoprotein C-IV OS=Mus musculus OX=10090 GN=Apoc4 PE=1 SV=1	Apoc4	-1.64	-1.27	3840107.009	3272787.164	2308368.995	2783210.638	868930.641	1389530.875	723678.7859	1040657.355	540733.9635	604491.6793	322410.3515	909923.4138	DTT
Q61268	Apolipoprotein C-IV OS=Mus musculus OX=10090 GN=Apoc4 PE=1 SV=1	Apoc4	-1.40	-1.40	7024802.828	6322107.557	6068588.678	5498890.63	2304164.675	2887101.793	2262026.803	1298202.575	2885628.132	3546477.978	1764718.199	1933430.393	DD
P51910	Apolipoprotein D OS=Mus musculus OX=10090 GN=Apod PE=1 SV=1	Apod	0.65	1.37	11537268.04	12333642.68	10776823	12159070.25	16415509.53	19397722.31	16709933.89	16759863.01	29088083.7	31053213.28	34695064.19	26623572.34	TCA
P51910	Apolipoprotein D OS=Mus musculus OX=10090 GN=Apod PE=1 SV=1	Apod	0.66	1.70	58068126.44	39197707.73	79805484.92	20452939.66	46649274.9	66542525.91	16740709.29	86988075.04	129936471.3	142648516.5	100439181.2	128107636.9	DTT
P51910	Apolipoprotein D OS=Mus musculus OX=10090 GN=Apod PE=1 SV=1	Apod	1.01	1.67	3152205.648	4338537.395	3559049.8	4532264.026	8483419.248	8018152.955	7043008.204	7050386.874	12646765.73	12877562.95	14629406.74	13125902.24	DD
P08226	Apolipoprotein E OS=Mus musculus OX=10090 GN=Apoe PE=1 SV=2	Apoe	0.10	0.20	16826557.26	16712359.15	13989741.32	16321928.2	25337686.99	13093842.9	9705025.377	22338223.23	20039513.16	24711651.59	14177346.51	21064837.43	TCA
P08226	Apolipoprotein E OS=Mus musculus OX=10090 GN=Apoe PE=1 SV=2	Apoe	-0.30	-0.64	17609833.85	17790656.66	17742340.13	21130911.98	16086317.82	13293922.97	9638583.135	8867217.113	6525567.924	12035890.63	13638713	2227263.544	DTT
P08226	Apolipoprotein E OS=Mus musculus OX=10090 GN=Apoe PE=1 SV=2	Apoe	0.84	0.49	56197201.97	46262919.97	42596821.98	50493603.43	78648303.06	70490156.36	85398520.08	52542570.16	67138711.5	80822007.24	55341870.24	57711699.2	DD
Q91V80	Apolipoprotein F OS=Mus musculus OX=10090 GN=Apof PE=2 SV=2	Apof	1.11	-0.31	17915.89062		35014.73298		43727.58008			67188.44047		11028.32148		37249.65733	TCA
Q91V80	Apolipoprotein F OS=Mus musculus OX=10090 GN=Apof PE=2 SV=2	Apof	1.13	1.12	598946.5255	811011.4775	1707564.542	550683.5701	1982440.597	2200068.949	1467829.373	3354638.317	1614217.501	1108135.48	650724.1827	3573143.877	DTT
Q01339	Beta-2-glycoprotein 1 OS=Mus musculus OX=10090 GN=Apoh PE=1 SV=1	Apoh	0.19	0.18	148887915	156331032.2	153692956.5	137749612.8	152599197.4	132580610.3	159430032.2	199039797.6	184978302	189128251.5	177488271.1	169677600.4	TCA
Q01339	Beta-2-glycoprotein 1 OS=Mus musculus OX=10090 GN=Apoh PE=1 SV=1	Apoh	-0.49	0.24	10643496.58	11907163.77	8850121.602	11129739.75	5925973.36	7331905.496	9164598.515	8829426.444	9680749.571	15654746.8	16953064.42	4934069.497	DTT
Q01339	Beta-2-glycoprotein 1 OS=Mus musculus OX=10090 GN=Apoh PE=1 SV=1	Apoh	0.46	0.56	93723561.85	98888318.13	99354758.54	105206789	140356046.4	136055640.6	155543042.3	138562599.6	151633282.5	159770874.1	138833804.6	154321218.3	DD
Q9Z1R3	Apolipoprotein M OS=Mus musculus OX=10090 GN=Apom PE=1 SV=1	Apom	-0.65	-0.65	6722721.433	6943749.359	5923357.322	4242589.203	5686700.063	3917338.056	2387250.048	5220646.881	3367464.584	4418737.503	5959606.702	3830788.131	TCA
Q9Z1R3	Apolipoprotein M OS=Mus musculus OX=10090 GN=Apom PE=1 SV=1	Apom	-0.69	-1.94	832396.0412	1317625.545	1004971.221	1646410.487	792737.4677	467298.0469	686668.763	330786.8744	255836.43	233495.2591	499868.9423	108348.4699	DTT
Q9Z1R3	Apolipoprotein M OS=Mus musculus OX=10090 GN=Apom PE=1 SV=1	Apom	-0.65	-0.92	4536157.344	4391665.915	4077401.976	4064440.898	2967196.676	3413019.406	3978972.348	1671581.112	1919624.717	2523522.869	2616460.016	1317937.366	DD
O08997	Copper transport protein ATOX1 OS=Mus musculus OX=10090 GN=Atox1 PE=1 SV=1	Atox1	0.16	1.13	72867.73081		196082.8731	14831.69612	56191.12939	32168.83594	24141.41505	383633.1096	141508.2459		159861.1219	261714.0284	DTT
P35455	Vasopressin-neurophysin 2-copeptin OS=Mus musculus OX=10090 GN=Avp PE=2 SV=1	Avp	-6.64	1.56			29184.94892						62823.07422	86052.40663		150983.408	DTT
Q64726	Zinc-alpha-2-glycoprotein OS=Mus musculus OX=10090 GN=Azgp1 PE=1 SV=2	Azgp1	0.49	0.54	15723882.06	16642768.97	17941678.33	20754143.3	21544779.62	28058746.93	32107150.61	27720284.1	27457368.96	25220324.45	25521905.49	23494619.77	TCA
Q64726	Zinc-alpha-2-glycoprotein OS=Mus musculus OX=10090 GN=Azgp1 PE=1 SV=2	Azgp1	-0.05	-0.38	2547736.806	5011271.589	3719126.54	6049268.223	3967145.699	2349692.303	11560928.51	3781247.154	4493647.699	4128232.502	4962192.251	662158.2237	DTT
Q64726	Zinc-alpha-2-glycoprotein OS=Mus musculus OX=10090 GN=Azgp1 PE=1 SV=2	Azgp1	0.67	0.70	13914496.47	14616224.12	13230906.53	12268913.98	16382077.16	19353001.18	21487474.31	19288121.31	22431922.26	21553568.59	22336854.25	21067564.68	DD
P01887	Beta-2-microglobulin OS=Mus musculus OX=10090 GN=B2m PE=1 SV=2	B2m	-0.64	-0.42	19622487.15	17954770.54	15600823.03	18747219.36	10354118.7	9295300.048	12469522.19	12161479.61	18341134.4	15186616.87	11515511.73	12308693.07	TCA
P01887	Beta-2-microglobulin OS=Mus musculus OX=10090 GN=B2m PE=1 SV=2	B2m	-0.22	-0.35	44859091.56	46068942.92	38191372.61	36583989.69	37531487.97	29015998	29518933.85	44118231.17	45409244.5	39498198.89	35340690.9	40432490.94	DTT
P01887	Beta-2-microglobulin OS=Mus musculus OX=10090 GN=B2m PE=1 SV=2	B2m	-0.39	0.12	14175102.22	16131725.51	15932574.85	15703038.05	12030609.77	11049458.15	8021765.852	14115880.78	14908827.64	17002057.15	15737187.28	16719908.88	DD
Q91XV3	Brain acid soluble protein 1 OS=Mus musculus OX=10090 GN=Basp1 PE=1 SV=3	Basp1	0.69	0.10	9719.083715				8163.671875	15632.71471		33806.77233			10402.42741		TCA
Q91XV3	Brain acid soluble protein 1 OS=Mus musculus OX=10090 GN=Basp1 PE=1 SV=3	Basp1	6.64	6.64						24204.74805	20511.20974	66254.37939		47581.46408			DTT

O35490	Betaine--homocysteine S-methyltransferase 1 OS=Mus musculus OX=10090 GN=Bhmt PE=1 SV=1	Bhmt	6.64	6.64					54836.27665	64377.8489	102476.0736	81278.63622	36294.63812				DD
D3Z6Q9	Bridging integrator 2 OS=Mus musculus OX=10090 GN=Bin2 PE=1 SV=1	Bin2	-2.16	-0.39	137455.1146		183153.2371	88229.27316	30602.95962		10722.91013	53701.61829	97266.93375	139781.2553	104951.2711		DTT
Q923D2	Flavin reductase (NADPH) OS=Mus musculus OX=10090 GN=Blvrb PE=1 SV=3	Blvrb	1.35	0.48	1146786.036	137961.3655	567260.6109	191276.9631	1265476.406	490317.9504	466574.2877	1265122.504	327780.8777	307754.6631	1034751.053	1780835.476	TCA
Q923D2	Flavin reductase (NADPH) OS=Mus musculus OX=10090 GN=Blvrb PE=1 SV=3	Blvrb	0.68	-0.15	139829.5469			99620.5116	449534.8497	465290.2961	565964.6737	340272.8051	48677.63501	241328.0262	437735.355	781729.2995	DD
P15327	Bisphosphoglycerate mutase OS=Mus musculus OX=10090 GN=Bpgm PE=1 SV=2	Bpgm	0.41	1.75	225879.6281	147689.3545	192563.0588	76896.80613	313189.125	86691.38006	143280.275	288170.452	502997.65	754788.1155	568737.3428	359515.2937	TCA
P15327	Bisphosphoglycerate mutase OS=Mus musculus OX=10090 GN=Bpgm PE=1 SV=2	Bpgm	0.68	1.44	142564.3594		58032.33099		173162.6928	182533.6199	161707.3848	116552.1089	156020.1751	210356.2957	209866.4191	389086.1671	DD
P07743	BPI fold-containing family A member 2 OS=Mus musculus OX=10090 GN=Bpifa2 PE=1 SV=1	Bpifa2	1.69	-0.54			44242.54947	32854.11191	417300.7344	31583.88194	1085696.954	46685.01506	2117617.614	16846.07207	88812.28848	101694.7464	TCA
P07743	BPI fold-containing family A member 2 OS=Mus musculus OX=10090 GN=Bpifa2 PE=1 SV=1	Bpifa2	0.86	-0.51		51189.77996	35604.75689	42509.71666	88565.96644	9453.863281	578260.4341	68092.96201	1844720.786	11761.58468	77651.85463	24735.88827	DTT
Q8CIF4	Biotinidase OS=Mus musculus OX=10090 GN=Btd PE=1 SV=2	Btd	0.48	0.34	222567.8441	385926.3584	233445.3047	344530.9371	413864.805	361643.7422	253592.6798	521946.959	338121.5388	194494.6101	323574.9569	544855.3821	DTT
P98086	Complement C1q subcomponent subunit A OS=Mus musculus OX=10090 GN=C1qa PE=1 SV=2	C1qa	-1.59	-1.50	1207286.098	1584009.768	1797785.348	1807376.652	639595.6797	349583.7512	327388.9506	454965.1156	491442.9776	385027.7229	919337.1744	279632.6476	TCA
P98086	Complement C1q subcomponent subunit A OS=Mus musculus OX=10090 GN=C1qa PE=1 SV=2	C1qa	-6.64	-6.64	524968.9598	886539.2136	138046.8296	1340497.987									DTT
P98086	Complement C1q subcomponent subunit A OS=Mus musculus OX=10090 GN=C1qa PE=1 SV=2	C1qa	-1.51	-1.43	2489190.781	2747666.54	1961119.606	1442122.257	445998.1781	710998.8555	459239.8597	544128.9991	684794.9305	783093.9971	1337653.826	622172.8868	DD
P14106	Complement C1q subcomponent subunit B OS=Mus musculus OX=10090 GN=C1qb PE=1 SV=2	C1qb	-2.34	-1.93	5076472.491	5995486.562	6703670.684	6927134.959	1240393.047	2140642.856	1367816.637	1288709.159	1562350.455	1766210.784	3303352.662	1124625.113	TCA
P14106	Complement C1q subcomponent subunit B OS=Mus musculus OX=10090 GN=C1qb PE=1 SV=2	C1qb	-6.64	-1.25	9635715.315	6214768.793	9600257.818	3797197.658				19820.29313	2113639.628	3898948.69	3429757.955	4034995.384	DTT
P14106	Complement C1q subcomponent subunit B OS=Mus musculus OX=10090 GN=C1qb PE=1 SV=2	C1qb	-1.35	-1.09	1781894.25	2406946.551	1941421.333	1326937.541	658026.631	717119.1516	576667.0327	403228.7709	635766.9312	757924.4146	1368074.628	742272.9196	DD
Q02105	Complement C1q subcomponent subunit C OS=Mus musculus OX=10090 GN=C1qc PE=1 SV=2	C1qc	-2.58	-1.45	2944690.756	3373669.381	2217192.95	2147485.679	774154.2188	276648.2209	252722.0825	733743.685	895106.6933	984273.4897	1745313.788	609982.6403	TCA
Q02105	Complement C1q subcomponent subunit C OS=Mus musculus OX=10090 GN=C1qc PE=1 SV=2	C1qc	-6.64	-2.11	264316.8696	343152.3906	263573.0457	218460.5082								33257.17809	DTT
Q02105	Complement C1q subcomponent subunit C OS=Mus musculus OX=10090 GN=C1qc PE=1 SV=2	C1qc	-1.33	-0.78	865870.9531	1769655.727	1134087.218	751455.7641	587611.7581	571023.5786	308858.5732	545146.2907	508976.4847	438644.1941	815821.0532	434415.6825	DD
Q8CG16	Complement C1r-A subcomponent OS=Mus musculus OX=10090 GN=C1ra PE=1 SV=1	C1ra	-0.13	-1.21	4369655.089	11930195.03	4835635.336	7560995.99	6651345.156	4555452.237	3227806.226	6420437.814	2766219.051	1611964.085	3486037.06	2395532.166	TCA
Q8CG16	Complement C1r-A subcomponent OS=Mus musculus OX=10090 GN=C1ra PE=1 SV=1	C1ra	-0.71	-1.33	7096483.873	5815306.612	4831384.666	4677532.42	3147127.344		3876260.124	2909721.467	1262987.309	4143767.98	5561667.319	832127.7846	DTT
Q8CG16	Complement C1r-A subcomponent OS=Mus musculus OX=10090 GN=C1ra PE=1 SV=1	C1ra	0.04	-0.95	21617408.93	25642357.62	26125197.24	27798455.87	27327901.64	22684113.86	26191697.21	26533192.76	20608240.39	23321639.9	21923840.48	11051288.72	DD
Q8CG14	Complement C1s-A subcomponent OS=Mus musculus OX=10090 GN=C1sa PE=2 SV=2	C1sa	-0.12	-1.44	704787.856	928526.7702	1082459.517	1004985.931	810409.6406	799253.7469	620524.2098	803997.094	289179.1856	348574.3499	461234.3575	146882.482	TCA
Q8CG14	Complement C1s-A subcomponent OS=Mus musculus OX=10090 GN=C1sa PE=2 SV=2	C1sa	-6.64	-6.64	89372034.5	86080278.2	114226637.8	78487610.45	26638.50216		51426.56925	19211.35276					DTT
Q8CG14	Complement C1s-A subcomponent OS=Mus musculus OX=10090 GN=C1sa PE=2 SV=2	C1sa	-0.44	-1.78	39186718.23	53129447.85	39353382.47	64848639.85	81767510.99	74803850.98	56249598.17	92208668.03	40831838.1	49902627.93	44079868.54	11576120.52	DD
P21180	Complement C2 OS=Mus musculus OX=10090 GN=C2 PE=1 SV=2	C2	1.09	0.66	5286921.869	5852163.448	6190173.805	6798465.282	9814878.134	12472251.04	11986153.44	10628524.98	7558999.04	6840510.815	7100471.788	8574694.39	TCA
P21180	Complement C2 OS=Mus musculus OX=10090 GN=C2 PE=1 SV=2	C2	0.77	-0.56	72631.76762	48468.74634	43332.9292	17320.16733	61827.6302	19479.31079	86275.3951	65652.89816	14057.87324		23377.07743		DTT
P21180	Complement C2 OS=Mus musculus OX=10090 GN=C2 PE=1 SV=2	C2	1.11	0.74	2761420.094	2577647.941	2481794.088	2441081.198	5165039.89	6221772.844	5587675.012	4483335.572	4878740.976	5132734.554	3944845.163	5590794.423	DD
P01027	Complement C3 OS=Mus musculus OX=10090 GN=C3 PE=1 SV=3	C3	-0.25	-0.60	733865518.4	794265352.9	700807911.4	682540949.8	708311457.8	719370961.8	764791208.5	708408313.6	574992429.9	535148488.2	550296273.5	420896580.8	TCA
P01027	Complement C3 OS=Mus musculus OX=10090 GN=C3 PE=1 SV=3	C3	-0.93	-1.05	68414278.13	92170626.81	74091671.37	69721151.63	39955517.49	59522943.6	60530225.4	41951005.64	25529270.25	46488882.57	67658818	18720174.19	DTT
P01027	Complement C3 OS=Mus musculus OX=10090 GN=C3 PE=1 SV=3	C3	0.23	-0.37	531160321.9	572985870.1	453667852.9	465118097.2	529233810.6	588903793.3	550490011.9	610088109.8	438371776.2	415226313.1	383898387.1	307444527.3	DD
P01029	Complement C4-B OS=Mus musculus OX=10090 GN=C4b PE=1 SV=3	C4b	0.58	-0.06	75074348.47	86395564.5	81263320.46	91472645.47	104878447.5	133101508	125969170.5	116086884.7	89623636.87	85271407.83	71787467.74	67744765.22	TCA
P01029	Complement C4-B OS=Mus musculus OX=10090 GN=C4b PE=1 SV=3	C4b	-0.04	-0.74	6400443.727	9960123.283	6719247.504	10216726.4	7152376.179	7785104.827	12659971.5	7900045.774	5005358.167	5837860.886	9026953.15	1263788.239	DTT
P01029	Complement C4-B OS=Mus musculus OX=10090 GN=C4b PE=1 SV=3	C4b	0.63	-0.15	58237041.85	62981067.56	52408823.18	62264562.44	77731784.52	81174693.21	86805482.57	95074436.42	61408145.41	57250630.48	51333010.01	56891438.29	DD
P08607	C4b-binding protein OS=Mus musculus OX=10090 GN=C4bpa PE=1 SV=3	C4bpa	0.03	0.16	32878044.95	40207711.91	47093908.86	38238799.57	43119710.29	37260176.27	35907035.09	52655891.25	36372771.06	48008296.88	35972112.86	40255053.49	TCA
P08607	C4b-binding protein OS=Mus musculus OX=10090 GN=C4bpa PE=1 SV=3	C4bpa	-0.69	0.08	2189660.281	3589210.193	3461460.287	3007310.176	879330.6885	1545483.906	1835099.269	1992165.899	1932865.031	4586132.448	4724640.041	453223.8503	DTT
P08607	C4b-binding protein OS=Mus musculus OX=10090 GN=C4bpa PE=1 SV=3	C4bpa	0.38	0.33	32717129.66	39065727.75	40473199.73	35746747.75	50366877.26	47706602.72	52826117.22	37494915.04	52562095.69	56648050.99	39276433.48	47897636.16	DD
Q8K182	Complement component C8 alpha chain OS=Mus musculus OX=10090 GN=C8a PE=1 SV=1	C8a	-0.96	-1.51	14480933.36	15442556.62	14558426.79	19585713.63	6670056.23	12044390.02	9989191.373	7592210.556	5472940.922	6516791.11	5890904.128	4620059.667	TCA
Q8K182	Complement component C8 alpha chain OS=Mus musculus OX=10090 GN=C8a PE=1 SV=1	C8a	-2.13	-1.70	2846678.864	3415876.297	2875614.2	3266912.731	392265.4092	331264.9492	645876.8455	571043.1741	433468.5208	888302.8869	868780.2987	306374.7346	DTT
Q8K182	Complement component C8 alpha chain OS=Mus musculus OX=10090 GN=C8a PE=1 SV=1	C8a	-0.81	-1.49	9525158.191	11774481.76	9621522.47	11688051.85	6288708.022	5871643.059	6847053.238	7026888.654	3597045.807	4171822.037	3731103.56	2911544.524	DD
Q8BH35	Complement component C8 beta chain OS=Mus musculus OX=10090 GN=C8b PE=1 SV=1	C8b	-1.04	-1.33	22903310.96	26171571.62	28124707.41	33318617.32	11206779.61	12454224.79	10311954.17	11030078.79	9454020.033	10396353.93	9456160.535	7269004.223	TCA
Q8BH35	Complement component C8 beta chain OS=Mus musculus OX=10090 GN=C8b PE=1 SV=1	C8b	-1.86	-2.15	1531826.908	2532630.227	1824101.85	2111048.551	363696.6555	138634.1484	494568.3964	268405.791	134209.8495	251767.725	398392.6854	137306.8748	DTT
Q8BH35	Complement component C8 beta chain OS=Mus musculus OX=10090 GN=C8b PE=1 SV=1	C8b	-0.87	-1.32	20897585.97	25007731.32	21080232.43	25516162.38	9981965.605	13387523.68	10882118.45	9524453.339	8237597.085	9544675.49	9190784.07	7588899.938	DD
Q8VCG4	Complement component C8 gamma chain OS=Mus musculus OX=10090 GN=C8g PE=1 SV=1	C8g	-1.15	-1.26	19358626.65	20941799.26	20491315.77	19813542.26	8963498.625	7972381.331	7307695.37	9701732.796	7826975.774	9804977.9	8251573.961	6620597.39	TCA
Q8VCG4	Complement component C8 gamma chain OS=Mus musculus OX=10090 GN=C8g PE=1 SV=1	C8g	-1.83	-1.63	1298359.896	2220142.177	1487253.905	2206227.512	351400.3999	151759.0156	342849.5999	169406.6676	762207.6808	2343063.367	3721322.759	115728.7558	DTT
Q8VCG4	Complement component C8 gamma chain OS=Mus musculus OX=10090 GN=C8g PE=1 SV=1	C8g	-1.00	-1.31	12056281.56	13162280.89	11792629.08	13252270.87	5796163.969	7656782.477	6648557.813	5507105.799	4821046.524	5106831.791	5141445.889	4988087.424	DD
P06683	Complement C9 OS=Mus musculus OX=10090 GN=C9 PE=1 SV=2	C9	-0.28	-1.56	21157599.54	21812106.1	22217201.51	20847369.92	15737872.01	15968630.46	15128544.93	16613216.95	6770131.572	7460203.817	7511386.124	6838318.729	TCA
P06683	Complement C9 OS=Mus musculus OX=10090 GN=C9 PE=1 SV=2	C9	-1.27	-2.91	1277003.976	2126326.722	1657343.428	1758360.649	258359.3872	234146.6563	242124.3792	54742.82533				196756.749	DTT
P06683	Complement C9 OS=Mus musculus OX=10090 GN=C9 PE=1 SV=2	C9	-0.30	-1.58	12148350.48	11635557.3	11113921.05	12767983.35	8009013.993	11380231.57	10968996.62	8876489.927	3982004.632	3300018.569	3578720.955	3477758.135	DD
P13634	Carbonic anhydrase 1 OS=Mus musculus OX=10090 GN=Ca1 PE=1 SV=4	Ca1	0.87	1.00	1475909.976	79941.28801	699850.4688	165797.0894	1645981.484	343774.8147	659515.0437	1771341.333	700017.3937	604604.5442	1424490.125	2378970.609	TCA
P00920	Carbonic anhydrase 2 OS=Mus musculus OX=10090 GN=Ca2 PE=1 SV=4	Ca2	2.77	3.31	7740254.87	1121442.028	2995609.048	973674.9326	23883686.32	11653870.6	11234667.29	18861074.65	22826488.81	25615838.18	28658684.64	33915257.39	TCA
P00920	Carbonic anhydrase 2 OS=Mus musculus OX=10090 GN=Ca2 PE=1 SV=4	Ca2	2.24	1.50	654456.1528	281561.9641	355214.5459	323530.4486	1821441	1044007.125	2789297.84	1185477.706	931706.8605	1184249.363	1767961.199	437290.5677	DTT
P00920	Carbonic anhydrase 2 OS=Mus musculus OX=10090 GN=Ca2 PE=1 SV=4	Ca2	3.24	3.02	3468086.105	540436.962	1510708.637	805596.6265	9325114.884	12431387.17	11295994.68	11219529.19	10138522.23	9787528.227	12319342.81	17094238.04	DD
P16015	Carbonic anhydrase 3 OS=Mus musculus OX=10090 GN=Ca3 PE=1 SV=3	Ca3	-0.51	-0.27	101276.7194		819657.7199	310313.3493	123951.6094	1191664.802	1020948.604	312921.7161	212150.5463	35146.83979	956607.749	157628.4344	TCA
P16015	Carbonic anhydrase 3 OS=Mus musculus OX=10090 GN=Ca3 PE=1 SV=3	Ca3	6.64	6.64							241385.9232			21369.01439			DTT
P16015	Carbonic anhydrase 3 OS=Mus musculus OX=10090 GN=Ca3 PE=1 SV=3	Ca3	1.18	0.07	39420.9375		173219.766	65914.39821	48934.08735	509659.0824	200168.3958	416961.9612	39192.78714	32416.72211	273661.9714	121193.885	DD
P0DP27	Calmodulin-2 OS=Mus musculus OX=10090 GN=Calm2 PE=1 SV=1	Calm2	-0.42	-0.59	82316.02016		265701.3258	15427.75349	61645.64319	61368.26172	10005.34516	259901.5449	25326.66807	110501.5229	129568.6944	411478.0793	DTT
P40124	Adenylyl cyclase-associated protein 1 OS=Mus musculus OX=10090 GN=Cap1 PE=1 SV=4	Cap1	1.09	1.38	44894.07037				93423.53906	87696.49044	99313.70878	98406	98821.59963	130765.5846	104375.1503	156047.974	TCA
P40124	Adenylyl cyclase-associated protein 1 OS=Mus musculus OX=10090 GN=Cap1 PE=1 SV=4	Cap1	2.14	2.01	24228.74805				104280.2598	112408.7347	108820.8393	91073.63095	90724.81566	105320.2484	76023.55051	157378.6707	DD
P24270	Catalase OS=Mus musculus OX=10090 GN=Cat PE=1 SV=4	Cat	1.27	1.40	179423.8017	62075.13559	123977.7328	62409.99348	413417.9609	435381.9748	306773.0766	431935.668	496697.6073	980154.5606	712653.6567	919122.1108	TCA
P24270	Catalase OS=Mus musculus OX=10090 GN=Cat PE=1 SV=4	Cat	1.24	1.37	53986.84961				112335.8806	179006.3557	173768.7347	115111.0365	119596.6012	125739.1149		317220.7513	DD
P06332	T-cell surface glycoprotein CD4 OS=Mus musculus OX=10090 GN=Cd4 PE=1 SV=1	Cd4	2.35	3.99	17026.79071	38502.80352	21802.42776	20339.59977	116411.9766			99096.15609	333745.1546	449378.452	467007.5567	198775.4825	TCA
Q9QWK4	CD5 antigen-like OS=Mus musculus OX=10090 GN=Cd5l PE=1 SV=3	Cd5l	0.54	-0.20	5823804.537	8166999.82	7786479.665	7021542.876	7761710.326	11158773.47	8947089.743	9390449.878	5346884.237	7536817.982	7995471.061	5175708.511	TCA
Q9QWK4	CD5 antigen-like OS=Mus musculus OX=10090 GN=Cd5l PE=1 SV=3	Cd5l	0.38	-0.58	1113648.595	1113689.305	1149668.379	2642060.635	1487301.966	701919.3047	3948920.122	1708025.744	813820.4268	1526405.181	1374512.385	148112.1684	DTT
Q9QWK4	CD5 antigen-like OS=Mus musculus OX=10090 GN=Cd5l PE=1 SV=3	Cd5l	0.35	0.19	5127821.542	8650816.837	9237937.949	8665108.572	12051497.77	9845921.364	11004364.31	9403212.234	8857416.115	11320244.73	10591620.52	4469427.406	DD
P04441	H-2 class II histocompatibility antigen gamma chain OS=Mus musculus OX=10090 GN=Cd74 PE=1 SV=3	Cd74	1.69	3.15	47632.40234	44655.4586	33696.55442	71503.22153	390048.714		210564.2625	321570.2673	322643.9734	602526.2917	236427.3102	1152778.8	DTT
Q8VCC2	Liver carboxylesterase 1 OS=Mus musculus OX=10090 GN=Ces1 PE=1 SV=1	Ces1	0.07	0.40	1185741.631	1159140.312	1383830.906	1921990.427	1837512.75	864694.771	1342430.374	1676370.764	1609232.359	2005047.686	2452163.841	1468199.413	TCA
Q8VCC2	Liver carboxylesterase 1 OS=Mus musculus OX=10090 GN=Ces1 PE=1 SV=1	Ces1	-1.69	-2.31	425033.8316	943069.4961	1601642.717	1279669.842	11369105.6		36590.74833	342171.5284			10366.37079	2650235.247	DTT
Q8VCC2	Liver carboxylesterase 1 OS=Mus musculus OX=10090 GN=Ces1 PE=1 SV=1	Ces1	0.32	0.27	1030488.875	1006670.8	1076828.893	1144522.919	1366468.724	1403250.821	1221523.518	1319163.443	1009909.91	1600862.808	1718719.503	1050571.496	DD
P23953	Carboxylesterase 1C OS=Mus musculus OX=10090 GN=Ces1c PE=1 SV=4	Ces1c	-1.49	-2.17	197901747.3	217803822.2	224666059.6	260090321.5	89144999.65	119476746.8	105078149.4	98114955.13	67391083.19	70530154.46	92059458.61	48611620.48	TCA
P23953	Carboxylesterase 1C OS=Mus musculus OX=10090 GN=Ces1c PE=1 SV=4	Ces1c	-1.06	-2.58	119949976.2	168685073.4	82512344.25	309596852.5	68775573.38	68145611.58	80836133.73	99666814.81	39787623.15	23926321.58	34228481.29	13971595.89	DTT
P23953	Carboxylesterase 1C OS=Mus musculus OX=10090 GN=Ces1c PE=1 SV=4	Ces1c	-1.54	-2.19	219695635.5	240735064.5	226062202.3	241380370.6	92348879.27	87278607.19	78817766.32	79287975.11	51384099.86	62907011.92	69024930.42	34455742.7	DD
Q8BK48	Pyrethroid hydrolase Ces2e OS=Mus musculus OX=10090 GN=Ces2e PE=1 SV=1	Ces2e	-6.64	-6.64	20297.68501	88685.89665	19472.46136	83543.87996									DTT
P04186	Complement factor B OS=Mus musculus OX=10090 GN=Cfb PE=1 SV=2	Cfb	1.02	0.52	35519618.08	39732816.24	41384888.94	50988876.25	67841469.39	94469564.12	112151048.8	76487950.39	61717330.27	67250417.13	68271464.18	46702205	TCA
P04186	Complement factor B OS=Mus musculus OX=10090 GN=Cfb PE=1 SV=2	Cfb	0.13	0.23	3311968.678	5238960.924	4018131.914	4356888.307	3412492.769	3489034.906	7433220.658	5034675.299	5283050.761	9779126.791	13813925.17	1685353.495	DTT
P04186	Complement factor B OS=Mus musculus OX=10090 GN=Cfb PE=1 SV=2	Cfb	1.05	0.52	25177763.82	27853127.73	29164884.55	30319464.15	51562757.08	55698519.3	58785022.26	65985014.76	42222677.19	48443160.13	47354879.38	36146030.55	DD
P03953	Complement factor D OS=Mus musculus OX=10090 GN=Cfd PE=1 SV=1	Cfd	-0.07	-1.79	4500941.044	8767397.024	7448143.55	10257427.51	6098838.27	12068231.45	9947340.983	7576434.446	2111096.307	2096807.982	3441645.034	2142292.012	TCA
P03953	Complement factor D OS=Mus musculus OX=10090 GN=Cfd PE=1 SV=1	Cfd	-0.33	-1.33	3957667.186	7758244.933	6882052.369	9196212.346	6488947.836	6861458.695	7969800.632	3695932.212	1865640.592	1936854.664	3162041.863	2468496.756	DTT
P03953	Complement factor D OS=Mus musculus OX=10090 GN=Cfd PE=1 SV=1	Cfd	-0.35	-1.10	10094164.28	8782451.416	7989332.33	8854966.394	6766105.847	7610434.475	5774960.479	7738978.19	3756285.586	4473963.065	4427445.102	3099184.196	DD
P06909	Complement factor H OS=Mus musculus OX=10090 GN=Cfh PE=1 SV=2	Cfh	0.76	0.54	66467938.1	73560977.04	76443940.52	89025300.03	108334579.6	154276983.2	138146955.3	126108357	106580782.9	122273736	113506649.9	91503245.33	TCA
P06909	Complement factor H OS=Mus musculus OX=10090 GN=Cfh PE=1 SV=2	Cfh	-0.33	0.38	6783905.198	10159826.51	9489930.223	13085380.24	3173898.616	6520756.469	8807240.494	6691935.109	5970410.714	15440045.89	19658008.93	1482830.574	DTT
P06909	Complement factor H OS=Mus musculus OX=10090 GN=Cfh PE=1 SV=2	Cfh	0.85	0.63	70527506.85	88973402.2	81899285.41	94408614.71	151554104.2	160909471.5	171432037.5	160068411.8	143225117	145259254	129923449.8	108102427.1	DD
Q61129	Complement factor I OS=Mus musculus OX=10090 GN=Cfi PE=1 SV=3	Cfi	-0.02	-0.03	24866809.47	29431210.69	26987670.74	29292648.81	24496223.4	24583312.43	26957961.69	27185372.21	26535311.28	25356053.71	25657659.42	21831403.48	TCA
Q61129	Complement factor I OS=Mus musculus OX=10090 GN=Cfi PE=1 SV=3	Cfi	-0.40	-1.01	4039765.872	5008344.557	3364647.573	4964958.491	2220511.301	5121763.875	3976027.91	2030012.216	1282733.282	2246070.888	2878922.227	497745.0141	DTT
Q61129	Complement factor I OS=Mus musculus OX=10090 GN=Cfi PE=1 SV=3	Cfi	0.03	0.08	23866810.88	25043663.76	22161044.61	23972695.51	24094101.42	25022535.91	22231594.51	24083923.86	25491802.34	26655352.68	25040678.32	22931808.38	DD
P18760	Cofilin-1 OS=Mus musculus OX=10090 GN=Cfl1 PE=1 SV=3	Cfl1	-0.06	-1.19	27312.52569		68977.34536	84443.1202	53763.4375	66061.90007	76474.67796			30285.12279	24538.23913	34100.58555	TCA
P11680	Properdin OS=Mus musculus OX=10090 GN=Cfp PE=2 SV=2	Cfp	-1.69	-1.86	11518367.95	22198621.57	18391227.99	16803038.96	6909907.867	4319108.342	3921300.447	5308455.519	4139148.703	4646549.464	4598124.054	4043897.832	TCA
P11680	Properdin OS=Mus musculus OX=10090 GN=Cfp PE=2 SV=2	Cfp	-1.14	-0.79	676685.1812	502957.6898	557360.1641	879146.2897	144410.6046	98856.75	363418.9255	311518.8888	246139.6406	529696.3522	343887.0169	19841.12768	DTT
P11680	Properdin OS=Mus musculus OX=10090 GN=Cfp PE=2 SV=2	Cfp	-0.54	-0.67	4518828.791	6349554.121	5235554.462	4992463.894	3586245.773	3562742.161	3333429.034	2901225.33	3246057.812	2997264.682	3179886.335	2722592.857	DD
Q8R1U2	Cell growth regulator with EF hand domain protein 1 OS=Mus musculus OX=10090 GN=Cgref1 PE=1 SV=1	Cgref1	-0.68	0.56	2181959.973	2238914.501	3862585.062	1448908.128	616481.88	295698.375	530678.1671	2689109.871	4229739.865	2803681.629	1407009.014	5975642.234	DTT
Q8R1U2	Cell growth regulator with EF hand domain protein 1 OS=Mus musculus OX=10090 GN=Cgref1 PE=1 SV=1	Cgref1	6.64	#VALUE!						12312.30867	10529.91061	12413.15696					DD
P07310	Creatine kinase M-type OS=Mus musculus OX=10090 GN=Ckm PE=1 SV=1	Ckm	2.21	1.02	725656.0954	12652.71301	185086.9441	319853.2079	1095938.047	3588219.566	3798275.314	1833938.977	393552.254	406887.9958	3325112.487	1373802.041	TCA
P07310	Creatine kinase M-type OS=Mus musculus OX=10090 GN=Ckm PE=1 SV=1	Ckm	2.83	1.19	396092.2578		31377.86912	201564.4448	1574551.158	6356282.587	2053791.894	4216903.775	1574041.107	1636446.285	3972671.749	1830487.373	DD
O88200	C-type lectin domain family 11 member A OS=Mus musculus OX=10090 GN=Clec11a PE=1 SV=1	Clec11a	-1.20	-1.09	79889.19531	115373.7297	97486.79158	77993.55445	32657.7338	59318.14968	49074.5697	34375.39317				41511.67306	DD
P43025	Tetranectin OS=Mus musculus OX=10090 GN=Clec3b PE=1 SV=2	Clec3b	-0.93	-6.64		385752.3916	226051.9788		155305.0156								TCA
P43025	Tetranectin OS=Mus musculus OX=10090 GN=Clec3b PE=1 SV=2	Clec3b	-1.92	-6.64	51468.02638	69026.66135	149018.5983	79568.28701	11184.48522	47853.26563							DTT

Q06890	Clusterin OS=Mus musculus OX=10090 GN=Clu PE=1 SV=1	Clu	0.73	0.17	84173473.95	115491781.2	119122313.5	127093052.8	132987111.5	190095760.4	158086135.3	152608655.2	108369045.1	95693425.26	87687280.71	113839563.8	TCA
Q06890	Clusterin OS=Mus musculus OX=10090 GN=Clu PE=1 SV=1	Clu	0.89	0.50	44770051.92	36951700.93	19611887.64	35021466.55	78443924.88	97647156.04	60917421.66	112552327.3	81010059.89	65698632.23	36978504.79	66386264.68	DTT
Q06890	Clusterin OS=Mus musculus OX=10090 GN=Clu PE=1 SV=1	Clu	1.19	0.64	49146079.23	54044871.34	47688369.3	50698339.79	97396547.52	105520424.5	102222672.8	96619019.67	84255711.29	77055746.62	59957763.6	73801232.72	DD
Q64739	Collagen alpha-2(XI) chain OS=Mus musculus OX=10090 GN=Col11a2 PE=2 SV=3	Col11a2	-2.44	-0.10	123075.0819	218638.8537	294298.6679	162429.925	28219.77701		15862.23371		125021.6858	77924.4919		133344.8762	DTT
P11087	Collagen alpha-1(I) chain OS=Mus musculus OX=10090 GN=Col1a1 PE=1 SV=4	Col1a1	-6.64	-3.12	123619.8382	329253.0359	396471.0839	420040.3205						27824.75633	25188.50632		DTT
Q3SXB8	Collectin-11 OS=Mus musculus OX=10090 GN=Colec11 PE=2 SV=2	Colec11	1.13	0.70	29484.4037	46261.59434	41971.87918	37175.84417	114909.375	96545.7279	54581.81426	73415.57347	69786.54505	70802.44094	58663.77825	55474.1279	TCA
Q8VBV7	COP9 signalosome complex subunit 8 OS=Mus musculus OX=10090 GN=Cops8 PE=1 SV=1	Cops8	6.64	6.64					22405.93549		21519.94949	120407.1104				142471.34	DTT
O89053	Coronin-1A OS=Mus musculus OX=10090 GN=Coro1a PE=1 SV=5	Coro1a	1.05	0.58	70500.62786	46266.55498	38537.04672	156639.9622	303786.2344	94943.83574	87221.2097	279062.8406	188733.178	363445.2998	168058.1206	250726.2358	TCA
O89053	Coronin-1A OS=Mus musculus OX=10090 GN=Coro1a PE=1 SV=5	Coro1a	2.51	1.68	11187.86426	13320.11075		12347.50763	69620.07419	76510.19048	140291.977	208021.7508	49850.86293	46663.9758	32714.46033	130621.3742	DD
Q61147	Ceruloplasmin OS=Mus musculus OX=10090 GN=Cp PE=1 SV=2	Cp	2.10	2.12	163823569.1	186172395.1	179414537.2	189545842.1	583346462.8	686692881.6	789814911.7	610882535.1	655003086.4	641557715.5	667284249.4	617660756.9	TCA
Q61147	Ceruloplasmin OS=Mus musculus OX=10090 GN=Cp PE=1 SV=2	Cp	1.80	1.02	18148761.47	42087041.46	18828100.2	49240799.33	95112208.04	95456503.94	235570405.7	74405379.11	66797578.11	73651758.28	112482904.2	13720018.66	DTT
Q61147	Ceruloplasmin OS=Mus musculus OX=10090 GN=Cp PE=1 SV=2	Cp	2.11	2.17	128318721.5	134345206	126978603.1	121710722.7	452629109.6	509483809	497945151.8	534351631.6	576434938.9	538608865.3	554014381.8	560011750.4	DD
Q9JHH6	Carboxypeptidase B2 OS=Mus musculus OX=10090 GN=Cpb2 PE=1 SV=1	Cpb2	0.85	1.39	1285823.766	2110540.768	912483.6731	1161512.264	2739185.711	911792.796	1496180.826	2442706.482	4569095.615	3742079.365	3712094.181	2491197.363	TCA
Q9JHH6	Carboxypeptidase B2 OS=Mus musculus OX=10090 GN=Cpb2 PE=1 SV=1	Cpb2	6.64	6.64							22298.98459		74834.64921	55963.64186	120527.1857		DTT
Q9JHH6	Carboxypeptidase B2 OS=Mus musculus OX=10090 GN=Cpb2 PE=1 SV=1	Cpb2	0.97	1.09	1325579.313	1694666.397	887024.8101	1625228.48	2339952.662	3140177.682	3081323.231	3224164.344	3603896.65	3038529.664	3059476.574	2366533.191	DD
Q9JJN5	Carboxypeptidase N catalytic chain OS=Mus musculus OX=10090 GN=Cpn1 PE=1 SV=1	Cpn1	-1.09	-1.17	10369484.58	12871015.01	14848314.76	14643534.48	5532044.906	7039463.53	4467149.472	5531337.503	5961491.069	5631887.541	5496014.139	4171604.632	TCA
Q9JJN5	Carboxypeptidase N catalytic chain OS=Mus musculus OX=10090 GN=Cpn1 PE=1 SV=1	Cpn1	-2.60	-1.30	828137.4037	842383.3434	1283093.351	1352037.408	68589.90938	90712.10938	164353.3136	97891.34412	107079.2698	324027.4432	438634.4106		DTT
Q9JJN5	Carboxypeptidase N catalytic chain OS=Mus musculus OX=10090 GN=Cpn1 PE=1 SV=1	Cpn1	-0.88	-1.11	4748400.891	5881707.418	5386002.404	5680345.479	1873758.759	2696185.775	2625740.901	2577714.838	2249946.624	1983972.405	2263185.599	2182610.565	DD
Q9DBB9	Carboxypeptidase N subunit 2 OS=Mus musculus OX=10090 GN=Cpn2 PE=1 SV=2	Cpn2	-1.02	-1.07	22953385.02	27854465.94	27886256.34	29744121.67	11767928.03	15381216.87	14548052.37	11754040.21	13951554.17	14252205.58	13194139	10452358.51	TCA
Q9DBB9	Carboxypeptidase N subunit 2 OS=Mus musculus OX=10090 GN=Cpn2 PE=1 SV=2	Cpn2	-1.34	-2.43	4010180.023	8982228.81	5243996.167	12934139.32	1861811.243	2425705.758	3133550.094	2012496.027	1238933.338	1103885.79	1738566.996	678989.4203	DTT
Q9DBB9	Carboxypeptidase N subunit 2 OS=Mus musculus OX=10090 GN=Cpn2 PE=1 SV=2	Cpn2	-1.21	-1.21	22247252.16	25097050.48	25098335.97	24353426.1	9344085.959	11094327.45	10381780.93	8836472.089	11346636.8	11749411.25	10733775.14	9707666.042	DD
Q9WVJ3	Carboxypeptidase Q OS=Mus musculus OX=10090 GN=Cpq PE=1 SV=1	Cpq	0.36	0.09	1009013.319	817337.0709	1087334.079	1173884.972	1247233.688	1422616.728	1446897.786	1191599.644	1030096.507	1220681.294	1117774.665	943481.1378	TCA
Q9WVJ3	Carboxypeptidase Q OS=Mus musculus OX=10090 GN=Cpq PE=1 SV=1	Cpq	0.31	-0.11	322274.6094	348336.9811	389786.0565	457515.2603	511916.404	493032.0344	421376.8788	409165.5172	366269.8934	403885.388	340709.928	317646.5859	DD
P14847	C-reactive protein OS=Mus musculus OX=10090 GN=Crp PE=1 SV=2	Crp	0.90	0.30	1753041.629	1634339.656	3079511.571	923727.4765	5439064.313	4958064.185	4231856.951	3558551.601	3369172.115	4523215.071	4187470.392	2272708.315	TCA
P14847	C-reactive protein OS=Mus musculus OX=10090 GN=Crp PE=1 SV=2	Crp	0.07	0.49	50088.07182	74200.61021	103829.7809	98018.53573			110296.9834	51999.00957	50260.9404	119659.3712	168910.3121		DTT
P14847	C-reactive protein OS=Mus musculus OX=10090 GN=Crp PE=1 SV=2	Crp	0.92	-0.12	872635.3555	688475.5787	764325.47	777855.6819	2157325.383	1915057.02	2243607.836	2317540.847	1081300.527	1650369.795	2513336.053	1170270.862	DD
P09581	Macrophage colony-stimulating factor 1 receptor OS=Mus musculus OX=10090 GN=Csf1r PE=1 SV=3	Csf1r	0.46	0.53	723801.4581	710941.3973	1068760.745	709743.0677	1420139.719	411431.2583	454860.6203	1330565.984	1162166.029	1218504.249	1381361.807	940750.8529	TCA
P09581	Macrophage colony-stimulating factor 1 receptor OS=Mus musculus OX=10090 GN=Csf1r PE=1 SV=3	Csf1r	1.04	0.55	366170.8535	558479.9574	485440.2103	671948.6899	1111308.001	1077386.899	1355314.645	1045459.768	788691.7358	773344.1777	1015248.5	662058.4137	DD
P12399	Protein CTLA-2-alpha OS=Mus musculus OX=10090 GN=Ctla2a PE=2 SV=2	Ctla2a	-2.33	-0.91	3080716.232	75558.79325	4536327.945	1294486.828	464296.3493	337183.25	208347.0738	749263.3732	792183.0168	2228706.224	730264.8753	1647003.686	DTT
P10605	Cathepsin B OS=Mus musculus OX=10090 GN=Ctsb PE=1 SV=2	Ctsb	-0.49	-0.77	861071.841	1271987.748	1165454.771	1184685.539	1312210.129	1474195.283	2102860.113	1057908.894	932467.595	1022902.404	972167.0372	887978.8352	TCA
P10605	Cathepsin B OS=Mus musculus OX=10090 GN=Ctsb PE=1 SV=2	Ctsb	6.64	#VALUE!						139398.7813	136777.408	16123.17258					DTT
P10605	Cathepsin B OS=Mus musculus OX=10090 GN=Ctsb PE=1 SV=2	Ctsb	1.10	0.61	572504.9458	529345.455	526319.654	554953.386	951441.0583	1282159.181	742817.7526	1151718.591	776306.8314	928869.7039	874741.9513	760581.1668	DD
P70269	Cathepsin E OS=Mus musculus OX=10090 GN=Ctse PE=1 SV=2	Ctse	-0.04	3.31		11915.98644					11622.56626		29535.51235			475616.5097	DTT
O70370	Cathepsin S OS=Mus musculus OX=10090 GN=Ctss PE=1 SV=2	Ctss	3.18	2.82	13741.88784	25378.06473	20750.54344	28393.33768	304660.9648	219377.0002	473027.2782	256294.0921	346980.9286	300212.6326	209900.1307	186285.9942	TCA
O70370	Cathepsin S OS=Mus musculus OX=10090 GN=Ctss PE=1 SV=2	Ctss	3.11	2.77	13157.80999	13803.86139		14494.8952	68052.52052		132266.6843	119486.5335	110240.7631	63527.67667		93905.94749	DTT
Q9WUU7	Cathepsin Z OS=Mus musculus OX=10090 GN=Ctsz PE=1 SV=1	Ctsz	0.68	0.93	284992.4234	372385.4587	468994.4174	630626.9849	466836.207	261698.6218	374735.0391	359087.8477	410782.6194	542943.155	349187.0926	583205.3051	TCA
Q9WUU7	Cathepsin Z OS=Mus musculus OX=10090 GN=Ctsz PE=1 SV=1	Ctsz	1.42	1.51	89061.46289	101380.5226	119067.2274	116050.6663	342211.6719	339175.7367	358009.1703	355877.1709	387116.7011	337180.0323	256370.8667	275100.2805	DD
P56395	Cytochrome b5 OS=Mus musculus OX=10090 GN=Cyb5a PE=1 SV=2	Cyb5a	6.64	6.64					80574.52593		22102.57928	195987.4637	116493.6774	110922.3329	104069.442	391480.0756	DTT
P62897	Cytochrome c, somatic OS=Mus musculus OX=10090 GN=Cycs PE=1 SV=2	Cycs	1.17	1.57	89903.74764	78717.41788	112012.8546	77291.94311	333904.9688	142014.799	109551.5249	338234.5968	260865.2982	357587.5072	239419.485	356748.3411	TCA
P62897	Cytochrome c, somatic OS=Mus musculus OX=10090 GN=Cycs PE=1 SV=2	Cycs	2.32	2.01	93346.58594	61869.00938	33454.00366	179835.8834	668741.6044	1069162.434	607845.7761	948521.4212	573751.7671	745787.4487	371135.5787	471656.4912	DD
Q9QUN9	Dickkopf-related protein 3 OS=Mus musculus OX=10090 GN=Dkk3 PE=2 SV=1	Dkk3	1.22	1.19	63074.14436	105992.4599	157680.2126	80223.46182	281644.3916	129142.5469	397795.3601	563637.3479	332097.1827	269868.5394	229301.9041	719611.8377	DTT
O55188	Dentin matrix acidic phosphoprotein 1 OS=Mus musculus OX=10090 GN=Dmp1 PE=2 SV=2	Dmp1	6.64	#VALUE!						9652.195169	11144.96386						TCA
Q61508	Extracellular matrix protein 1 OS=Mus musculus OX=10090 GN=Ecm1 PE=1 SV=2	Ecm1	0.32	0.97	1168717.741	193952.0741	860885.4901	235858.6703	2332239.695		8844.381924	470746.4961	927746.4625	777532.6317	1185399.908	3599620.096	DTT
Q61508	Extracellular matrix protein 1 OS=Mus musculus OX=10090 GN=Ecm1 PE=1 SV=2	Ecm1	1.83	0.77	9087.005859	16939.19574	7953.494023	16362.27828	40990.26752	31785.09958	58045.7188	58624.76692	17691.44963	24622.32725	32789.98989	5733.059115	DD
P10126	Elongation factor 1-alpha 1 OS=Mus musculus OX=10090 GN=Eef1a1 PE=1 SV=3	Eef1a1	3.40	2.33	38579.85651			27831.11611	258172.2656	543562.8277	425233.9873	282384.8963	135534.6558	154845.7591	201596.1626	173453.1279	TCA
Q8BPB5	EGF-containing fibulin-like extracellular matrix protein 1 OS=Mus musculus OX=10090 GN=Efemp1 PE=1 SV=1	Efemp1	-0.38	-0.72	566458.8309	574223.555	775337.3043	706818.76	423648.1406	585229.1172	603784.8396	266844.0546	255276.8217	320489.1572	357276.7227	241302.4658	TCA
Q8BPB5	EGF-containing fibulin-like extracellular matrix protein 1 OS=Mus musculus OX=10090 GN=Efemp1 PE=1 SV=1	Efemp1	0.06	-6.64	30977.77421	95529.63129	19921.22709	152744.2967			110375.6706	29243.36869					DTT
Q8BPB5	EGF-containing fibulin-like extracellular matrix protein 1 OS=Mus musculus OX=10090 GN=Efemp1 PE=1 SV=1	Efemp1	1.21	-6.64	91418.25781	41716.063	108257.0566	71061.57235	89968.06601	109864.8433	177060.5281	105834.0566					DD
Q01279	Epidermal growth factor receptor OS=Mus musculus OX=10090 GN=Egfr PE=1 SV=1	Egfr	-0.52	-0.82	22545976.78	27103685.87	27559223.95	32027081.87	17592646.7	23971258.57	20848772.99	18869043.35	18272622.54	15263904.74	15604128.9	13046748.41	TCA
Q01279	Epidermal growth factor receptor OS=Mus musculus OX=10090 GN=Egfr PE=1 SV=1	Egfr	-0.66	-1.37	1321522.644	1941540.834	2034196.505	2729236.489	295827.0739	882498.7578	1154708.933	364199.7191	423995.4312	813179.5892	1252496.917	21898.46029	DTT
Q01279	Epidermal growth factor receptor OS=Mus musculus OX=10090 GN=Egfr PE=1 SV=1	Egfr	-0.51	-0.89	25085551.6	26795136.54	28268271.28	28730131.98	16176871.4	20663655.41	20603572.77	17059343.09	17726264.22	14351618.07	13706390.8	12809275.38	DD
P17182	Alpha-enolase OS=Mus musculus OX=10090 GN=Eno1 PE=1 SV=3	Eno1	3.81	2.75	31039.8709		35312.07544		536753.5977	636590.6802	630895.4208	356452.8254	222425.3666	197682.5323	238426.377	340658.8434	TCA
P21550	Beta-enolase OS=Mus musculus OX=10090 GN=Eno3 PE=1 SV=3	Eno3	1.51	0.35	176705.2343	86006.22323	198639.5833	92709.80679	446539.1445	219736.9164	216142.3818	270069.2989	139646.2268	183231.9018	496746.6116	277559.0389	TCA
P21550	Beta-enolase OS=Mus musculus OX=10090 GN=Eno3 PE=1 SV=3	Eno3	6.64	6.64					136806.369	298474.4621	123229.9178	244397.7777	17292.23396		194001.4562	12784.56468	DD
O88947	Coagulation factor X OS=Mus musculus OX=10090 GN=F10 PE=1 SV=1	F10	-1.36	-1.33	17007627.32	21394175.66	20692284.31	15150172.21	6607622.695	4684029.677	4122671.715	6386034.405	4867488.027	5507060.441	5957764.84	5122157.221	TCA
O88947	Coagulation factor X OS=Mus musculus OX=10090 GN=F10 PE=1 SV=1	F10	-1.57	-1.38	545104.4248	427658.5354	507835.6157	319120.1756	148945.3362	96938.59375	136985.8338	56678.92236	22278.9933	87222.33933	164027.0413	58675.65481	DTT
O88947	Coagulation factor X OS=Mus musculus OX=10090 GN=F10 PE=1 SV=1	F10	-1.14	-1.01	10262094.75	11455458.28	10339116.6	10402275.56	4186229.446	5549224.75	5214396.722	4482168.261	4543870.894	5134222.14	4759448.604	4568681.908	DD
Q80YC5	Coagulation factor XII OS=Mus musculus OX=10090 GN=F12 PE=1 SV=2	F12	0.54	0.16	5377659.392	5820090.374	5216865.118	7461479.928	7029450.797	13658730.39	8810766.492	5889715.967	7255432.436	6941118.879	6327088.868	6847450.148	TCA
Q80YC5	Coagulation factor XII OS=Mus musculus OX=10090 GN=F12 PE=1 SV=2	F12	0.05	-1.28	131692.6178	152601.2468	235445.6774	128243.462	216531.9594	117258.0391		45416.22586	36212.94035	49836.63971	19412.51745	25725.71461	DTT
Q80YC5	Coagulation factor XII OS=Mus musculus OX=10090 GN=F12 PE=1 SV=2	F12	0.23	0.40	5556845.061	3794732.16	4414320.998	4264332.638	3983618.509	5341234.757	5362496.75	4520672.951	5498608.581	5816182.791	5589318.471	6652844.521	DD
Q8BH61	Coagulation factor XIII A chain OS=Mus musculus OX=10090 GN=F13a1 PE=1 SV=3	F13a1	-2.57	-2.27	552653.7234	842045.4397	2608908.334	1458510.624	243005.0227		241223.1794	142820.4308	334037.5027	158959.0652	233923.2526	262736.0702	DTT
Q07968	Coagulation factor XIII B chain OS=Mus musculus OX=10090 GN=F13b PE=1 SV=2	F13b	-1.25	-1.38	1210936.677	1691132.544	2055421.141	2183213.841	579957.1797	2932065.323	818281.0734	697452.337	677164.084	1698415.953	1824971.174	593294.248	TCA
Q07968	Coagulation factor XIII B chain OS=Mus musculus OX=10090 GN=F13b PE=1 SV=2	F13b	-6.64	-6.64	57067.12499	59645.13568	65466.49387	82771.21378									DTT
Q07968	Coagulation factor XIII B chain OS=Mus musculus OX=10090 GN=F13b PE=1 SV=2	F13b	-1.45	-1.45	1926868.625	2223896.881	2221399.583	2209058.813	673008.7911	723187.7051	792648.8042	746875.8369	794318.1529	834883.1371	917103.9381	479783.2221	DD
P19221	Prothrombin OS=Mus musculus OX=10090 GN=F2 PE=1 SV=1	F2	-0.63	-0.50	72802649.8	89132295.54	75558251.97	69549719.89	54731606.34	44232913.21	50567270.31	56646718.05	76732884.01	49696843.13	49758216.68	45623461.18	TCA
P19221	Prothrombin OS=Mus musculus OX=10090 GN=F2 PE=1 SV=1	F2	-0.92	-0.98	110637587.6	9322567.871	152681596.5	65592101.5	41324019.04	52058116	15884282.54	64594243	10816687.15	20574641.59	48262155.11	65544574.85	DTT
P19221	Prothrombin OS=Mus musculus OX=10090 GN=F2 PE=1 SV=1	F2	-0.31	-0.47	76309388.92	64457542.9	54649572.17	68843152.46	44297659.14	48499837.41	47893814.45	38623157.33	41225469.28	44233319.34	50325088.81	39002507.72	DD
O88783	Coagulation factor V OS=Mus musculus OX=10090 GN=F5 PE=1 SV=1	F5	0.03	-0.08	1746018.742	1856855.656	1403850.572	1344021.636	1761149.32	1285966.995	937934.294	1960627.384	1681918.634	1952713.281	1886127.023	1186833.281	TCA
O88783	Coagulation factor V OS=Mus musculus OX=10090 GN=F5 PE=1 SV=1	F5	-0.29	0.50	3760144.288	2406899.726	4631810.419	2129612.644	2344220.106	2442285.115	991761.4059	4707996.575	4030554.835	4689268.124	3960665.824	5509088.644	DTT
O88783	Coagulation factor V OS=Mus musculus OX=10090 GN=F5 PE=1 SV=1	F5	-0.51	0.10	446670.6406	533376.2734	471618.5554	393080.5421	282625.705	436809.8996	288649.3539	53988.38149	615430.8364	546270.1686	433290.3965	398362.2275	DD
P11404	Fatty acid-binding protein, heart OS=Mus musculus OX=10090 GN=Fabp3 PE=1 SV=5	Fabp3	1.45	0.45	507361.6788	27057.8345	617669.8331	669337.9469	2513476.313		258335.822	1272508.957	501604.4313	775701.8109	1497006.383	317666.1786	TCA
P11404	Fatty acid-binding protein, heart OS=Mus musculus OX=10090 GN=Fabp3 PE=1 SV=5	Fabp3	-0.60	0.10	6924962.5	8371436.919	10164375.23	8011373.839	6120327.421	4185691.134	2081752.719	5275843.971	8346725.924	9064737.644	8780334.062		DD
P04117	Fatty acid-binding protein, adipocyte OS=Mus musculus OX=10090 GN=Fabp4 PE=1 SV=3	Fabp4	-3.17	-1.24			2068642.166	521775.6458		100232.5483		87606.09367	332416.2194	94287.23338	881755.5199		TCA
Q08879	Fibulin-1 OS=Mus musculus OX=10090 GN=Fbln1 PE=1 SV=2	Fbln1	-1.51	-2.24	1160165.299	1305500.93	1309731.299	1651666.816	483420.9063	558285.5936	456498.3484	489119.1565	253414.9983	304271.4787	314683.9079	239801.6229	TCA
Q08879	Fibulin-1 OS=Mus musculus OX=10090 GN=Fbln1 PE=1 SV=2	Fbln1	-1.16	-1.85	278014.1797	501984.772	637710.6319	560657.7575	99764.906	162252.2991	160013.5895	150924.7622	32168.61506	61240.98532	174931.3969	49032.73788	DD
Q9WVH9	Fibulin-5 OS=Mus musculus OX=10090 GN=Fbln5 PE=1 SV=1	Fbln5	1.66	1.79	30725.12171			13100.14492	52152.58588		76914.71806	52110.4203	196071.4061	50950.12312		40470.75847	DTT
O70165	Ficolin-1 OS=Mus musculus OX=10090 GN=Fcn1 PE=1 SV=1	Fcn1	-1.59	-1.63	801885.8714	1124884.772	1055571.274	1136600.351	267784.9219	350814.0802	242080.5055	196418.6582	293507.4856	244524.7104	300652.441	202197.5081	TCA
O70165	Ficolin-1 OS=Mus musculus OX=10090 GN=Fcn1 PE=1 SV=1	Fcn1	-0.77	-0.92	203049.7422	338328.6826	203524.7975	706166.8886	65357.29024	134199.77	49456.76672	82448.0609	553540.0254	110229.0401	49832.10037	50726.55796	DD
A3KGK3	Fer-1-like protein 4 OS=Mus musculus OX=10090 GN=Fer1l4 PE=2 SV=3	Fer1l4	6.64	#VALUE!					11944.22007	450993.625							DTT
Q9QXC1	Fetuin-B OS=Mus musculus OX=10090 GN=Fetub PE=1 SV=1	Fetub	0.65	0.67	21031952.97	28106495.35	22423427.18	24461171.76	35775140.2	44616690.5	42041246.44	41000303.49	36864339.91	34040333.43	56892323.24	38703166.82	TCA
Q9QXC1	Fetuin-B OS=Mus musculus OX=10090 GN=Fetub PE=1 SV=1	Fetub	0.41	0.49	2956261.815	5702469.337	3872796.382	5927011.834	4878949.735	5151499.086	15401696.24	4523883.145	7253944.754	6189456.538	11562768.16	4359379.708	DTT
Q9QXC1	Fetuin-B OS=Mus musculus OX=10090 GN=Fetub PE=1 SV=1	Fetub	0.60	0.80	19508931.99	21544181.15	16656550.75	18594990.87	28175170.52	29275183.21	26567248.56	30992308.74	33922722.33	31187465.27	39080443.56	25391576.16	DD
E9PV24	Fibrinogen alpha chain OS=Mus musculus OX=10090 GN=Fga PE=1 SV=1	Fga	-0.03	-0.68	656263.4426	6791559.642	1481957.013	960905.7802	399858.375	2033557.51	1602841.652	2384473.102	862537.6225	1098341.202	352968.6805	488043.3802	TCA
E9PV24	Fibrinogen alpha chain OS=Mus musculus OX=10090 GN=Fga PE=1 SV=1	Fga	0.86	-0.99	291678.6279	267992.9137	935113.8392	373075.7001	75930.78809	138133.5625	524671.5655	181063.2622	119525.484	250408.8608	79381.10246	31243.42748	DTT
E9PV24	Fibrinogen alpha chain OS=Mus musculus OX=10090 GN=Fga PE=1 SV=1	Fga	0.23	-0.63	92363.89844	1183886.974	234263.2296	202589.7084	130790.3767	592352.5447	440282.2706	503863.2981	72241.75741	251275.9578	73146.13348	86841.90766	DD
Q8K0E8	Fibrinogen beta chain OS=Mus musculus OX=10090 GN=Fgb PE=1 SV=1	Fgb	0.12	-1.02	22722.78056	661525.1769	34943.89557	51130.62062		170078.0651	30668.69918	187764.6035	22853.40618	46733.55742		12379.89781	TCA
Q8K0E8	Fibrinogen beta chain OS=Mus musculus OX=10090 GN=Fgb PE=1 SV=1	Fgb	-0.60	0.26		1799308.096	31155.06087	152260.6825	92555.09133	455581.9853	395879.705	62546.6792	35394.47712	255885.7428			DD
Q8VCM7	Fibrinogen gamma chain OS=Mus musculus OX=10090 GN=Fgg PE=1 SV=1	Fgg	1.23	-2.86	56610.5409	4733532.257	39134.14559	161173.0821		286306.1789		674722.011		211250.2131		13366.72613	TCA
Q8VCM7	Fibrinogen gamma chain OS=Mus musculus OX=10090 GN=Fgg PE=1 SV=1	Fgg	0.06	-1.47	1088224.875	5626138.733	1235054.967	2287993.579	3224100.873	1991108.164	2839298.016	2689241.841	965581.6082	1165475.9	446971.7874	6788.329232	DD
P12804	Fibroleukin OS=Mus musculus OX=10090 GN=Fgl2 PE=1 SV=1	Fgl2	6.64	6.64						100139.2813	6613838.937		104692.4514	124281.1163	24946.43486		DTT
P35917	Vascular endothelial growth factor receptor 3 OS=Mus musculus OX=10090 GN=Flt4 PE=1 SV=1	Flt4	-1.76	-6.64	40536.43066	40294.88454	34608.27744	30552.99141	15211.2152			7107.288674					DD
P11276	Fibronectin OS=Mus musculus OX=10090 GN=Fn1 PE=1 SV=4	Fn1	0.28	0.36	144157500.8	82513873.86	132690956.5	117045190.6	154952485.1	89164396.39	102414944.5	179801474.4	200899312.8	162401244.4	136492218.1	118316880.8	TCA
P11276	Fibronectin OS=Mus musculus OX=10090 GN=Fn1 PE=1 SV=4	Fn1	-0.69	0.05	9630908.967	5314812.377	9816019.323	6526271.295	3066310.068	2279301.448	5553793.504	8722668.987	5427280.303	9564246.57	10754064.66	2410986.695	DTT
P11276	Fibronectin OS=Mus musculus OX=10090 GN=Fn1 PE=1 SV=4	Fn1	0.80	0.61	83315146.53	62868130.81	67891367.68	65862295.19	125066799.6	105283542.8	136412024	96588917.8	133043044.4	106799688.8	83978907.24	80939558.15	DD
Q62356	Follistatin-related protein 1 OS=Mus musculus OX=10090 GN=Fstl1 PE=2 SV=2	Fstl1	6.64	#VALUE!						75648.78573	86849.76849						TCA

P09528	Ferritin heavy chain OS=Mus musculus OX=10090 GN=Fth1 PE=1 SV=2	Fth1	1.40	3.05				10733.29956	28363.09558	24111.52539				28166.2955		88615.6207	DTT
P16858	Glyceraldehyde-3-phosphate dehydrogenase OS=Mus musculus OX=10090 GN=Gapdh PE=1 SV=2	Gapdh	2.49	1.31	1864384.685	214089.6513	839776.8757	1031290.458	4208226.301	10356367.63	8730706.07	4371354.735	1216354.736	1541842.719	5999754.72	3252352.641	TCA
P16858	Glyceraldehyde-3-phosphate dehydrogenase OS=Mus musculus OX=10090 GN=Gapdh PE=1 SV=2	Gapdh	1.75	-0.01	66392.37377		86034.34263	42532.44272			495832.2782	218972.3269	14890.91096	107316.5356	503511.7559	21191.7586	DTT
P16858	Glyceraldehyde-3-phosphate dehydrogenase OS=Mus musculus OX=10090 GN=Gapdh PE=1 SV=2	Gapdh	1.85	1.14	17043325.1	4373595.497	12609305.36	5299573.431	20728248.65	22003709.05	21032730.82	14173832.26	11723827.23	13569854.4	23703579.55	36656993.15	DD
Q9Z0E6	Guanylate-binding protein 2 OS=Mus musculus OX=10090 GN=Gbp2 PE=1 SV=1	Gbp2	6.64	6.64					52303.62891	29344.18454	39346.91986	27965.78544	14847.10685	17537.69083	14026.90278	26627.87061	TCA
P21614	Vitamin D-binding protein OS=Mus musculus OX=10090 GN=Gc PE=1 SV=2	Gc	-0.25	-0.36	73287900.71	94701617.53	93620441.6	97409215.8	69754114.18	100230295.1	85132221.36	63001623.71	61682933.71	74965757.62	57119826.08	71542132.65	TCA
P21614	Vitamin D-binding protein OS=Mus musculus OX=10090 GN=Gc PE=1 SV=2	Gc	-0.13	-0.99	33350038.57	49208019.28	34431313.92	57015477.35	27905987.53	27860318.29	96097575.78	40326947.51	16581746.2	23388170.01	33112734.26	15957440.88	DTT
P21614	Vitamin D-binding protein OS=Mus musculus OX=10090 GN=Gc PE=1 SV=2	Gc	0.18	-0.35	186277194.1	208318356.5	216401445.3	203761283.4	185923553.5	215723365.8	207426299.6	254533981.5	159478704	173613143.7	169988564.1	159577653	DD
Q02596	Glycosylation-dependent cell adhesion molecule 1 OS=Mus musculus OX=10090 GN=Glycam1 PE=1 SV=1	Glycam1	-2.36	-1.59	150480.0542	124681.0491	341430.2519	32129.31925	26708.58734							45632.89501	DTT
P01642	Ig kappa chain V-V region L7 (Fragment) OS=Mus musculus OX=10090 GN=Gm10881 PE=1 SV=1	Gm10881	1.19	2.51	567098.1777	1183926.688	764944.4371	1024676.772	1614275.172	1464141.452	2194081.834	2297587.284	4955887.126	6720625.728	6157400.974	3515520.398	TCA
P01639	Ig kappa chain V-V region MOPC 41 OS=Mus musculus OX=10090 GN=Gm5571 PE=1 SV=1	Gm5571	1.18	2.35	158396.5695	425768.7997	309884.7653	789775.2397	1002336.922	1284824.364	599906.3057	721881.4531	2582305.343	2866838.168	2922265.349	1764229.335	TCA
Q3V2D2	Uncharacterized protein C5orf46 homolog OS=Mus musculus OX=10090 GN=Gm94 PE=3 SV=1	Gm94	-6.64	-0.70	257378.046	209740.3102	591888.3344	148906.4302					74931.95217		618683.5472	134336.6265	DTT
O35930	Platelet glycoprotein Ib alpha chain OS=Mus musculus OX=10090 GN=Gp1ba PE=1 SV=2	Gp1ba	-1.61	-1.40	725205.543	711863.6973	1346731.699	969504.1102	388895.6016	160351.6343	96044.77977	290541.87	369152.0808	462432.0854	365328.7683	481699.1129	TCA
O35930	Platelet glycoprotein Ib alpha chain OS=Mus musculus OX=10090 GN=Gp1ba PE=1 SV=2	Gp1ba	-1.39	-1.29	569814.5938	492621.9501	888133.0036	746423.0573	169844.0566	205529.4592	265369.7593	120236.6968	266809.456	397389.5948	208602.3409	269809.3847	DD
O08742	Platelet glycoprotein V OS=Mus musculus OX=10090 GN=Gp5 PE=1 SV=1	Gp5	-3.01	-2.43	80769.31768		244459.6311	153374.0098	22666.42969			15893.55432	17060.98086	24906.25601	20064.18212	56334.30325	TCA
O08742	Platelet glycoprotein V OS=Mus musculus OX=10090 GN=Gp5 PE=1 SV=1	Gp5	-3.24	-1.50	259152.9688		165596.9277	88310.08892	17629.96599					44382.53514	42291.39391	91537.06407	DD
P06745	Glucose-6-phosphate isomerase OS=Mus musculus OX=10090 GN=Gpi PE=1 SV=4	Gpi	6.64	6.64					118300.4926	135653.6294	130699.4312	200329.316	75286.10956	105726.8465	52326.157	35044.70308	DD
O70362	Phosphatidylinositol-glycan-specific phospholipase D OS=Mus musculus OX=10090 GN=Gpld1 PE=1 SV=1	Gpld1	-0.48	0.08	42432291.5	38386428.15	37232118.41	34320643.65	27715016.2	31412277.04	26112436.29	26209591.27	40482393.26	43592378.5	44475371.66	34443964.48	TCA
O70362	Phosphatidylinositol-glycan-specific phospholipase D OS=Mus musculus OX=10090 GN=Gpld1 PE=1 SV=1	Gpld1	-1.54	-1.38	2455608.082	4816329.886	3125767.119	3555510.037	885833.9398	519690.0625	662474.1733	659195.5256	537505.2076	982566.3181	1960024.38	121910.468	DTT
O70362	Phosphatidylinositol-glycan-specific phospholipase D OS=Mus musculus OX=10090 GN=Gpld1 PE=1 SV=1	Gpld1	-0.43	-0.06	24473483.63	26254823.58	21830141.44	20762113.92	17391874.66	18623864.06	17918674.05	12947873.13	23804495.96	22716468.38	23935660.47	20500479.78	DD
P46412	Glutathione peroxidase 3 OS=Mus musculus OX=10090 GN=Gpx3 PE=1 SV=2	Gpx3	0.41	0.70	6975713.683	10234289.76	8528792.492	9720097.151	13104651.44	10876197.93	12466833.55	14164262.53	13719664.35	17710364.66	11490311.29	11189637.07	TCA
P46412	Glutathione peroxidase 3 OS=Mus musculus OX=10090 GN=Gpx3 PE=1 SV=2	Gpx3	0.60	-0.11	819861.674	728536.8651	1089816.799	654996.1044	2830418.691	800920.4297	1774170.129	462865.5704	207469.9591	334754.2795	574001.2993	6356577.629	DTT
P46412	Glutathione peroxidase 3 OS=Mus musculus OX=10090 GN=Gpx3 PE=1 SV=2	Gpx3	0.45	0.40	5958154.938	6844888.774	5063439.915	4755854.468	6247880.352	9880821.907	7879237.211	7799525.122	7033941.336	8255365.361	6946010.505	9041018.173	DD
P28798	Progranulin OS=Mus musculus OX=10090 GN=Grn PE=1 SV=2	Grn	1.43	0.35	139340.209	246935.7746	139661.3152	166438.3151	567073.6992	591329.2408	389786.7215	610502.7405	248460.7102	343314.2777	220987.5901	410893.3001	TCA
P28798	Progranulin OS=Mus musculus OX=10090 GN=Grn PE=1 SV=2	Grn	2.53	2.58	1837835.9	1747530.569	900703.1699	1096855.858	6860605.034	8108926.572	7209436.563	16446382.26	9970088.789	11570215.34	5653979.003	9027824.143	DTT
P28798	Progranulin OS=Mus musculus OX=10090 GN=Grn PE=1 SV=2	Grn	2.41	1.74	309372.9688	463812.6883	411783.2806	391301.5679	2591202.046	2962620.514	2328590.418	2800536.081	2007519.735	1900406.569	751127.6918	1212376.492	DD
P13020	Gelsolin OS=Mus musculus OX=10090 GN=Gsn PE=1 SV=3	Gsn	-1.11	-1.49	65549241.84	66110473.2	79126914.28	91496344.76	38328598.77	12643026.73	25426945.07	33459093.44	23172275.54	27781046.29	28847404.8	22840457.47	TCA
P13020	Gelsolin OS=Mus musculus OX=10090 GN=Gsn PE=1 SV=3	Gsn	-2.40	-1.66	5552786.779	5053188.902	6709125.58	4288891.503	502482.1945	589744.2539	232787.6509	436796.0802	843429.3997	1811786.269	2827673.066	182865.0903	DTT
P13020	Gelsolin OS=Mus musculus OX=10090 GN=Gsn PE=1 SV=3	Gsn	-0.62	-1.39	48038998.94	45781078.96	49141334.88	50802473.98	27026971.79	32038865.86	25589445.65	30625460.39	19394606.4	22854440.9	23533398.16	23870482.61	DD
P14426	H-2 class I histocompatibility antigen, D-K alpha chain OS=Mus musculus OX=10090 GN=H2-D1 PE=1 SV=1	H2-D1	3.45	3.08	37936.24502	28789.26316	39459.06578	33974.46149	331192.1875	419023.5714	425174.5457	359738.1397	349888.5543	306389.555	232398.8923	306977.73	TCA
P14426	H-2 class I histocompatibility antigen, D-K alpha chain OS=Mus musculus OX=10090 GN=H2-D1 PE=1 SV=1	H2-D1	6.64	6.64					387478.3968	316855.8151	271301.7669	307750.044	253253.7883	282804.727	244937.9851	331576.9987	DD
P01902	H-2 class I histocompatibility antigen, K-D alpha chain OS=Mus musculus OX=10090 GN=H2-K1 PE=1 SV=1	H2-K1	2.43	3.44	134426.8569	143109.6545	165553.2309	119677.8259	1271256.332	626844.2223	650655.6844	1365831.996	1607995.453	2140195.231	933969.5789	1411922.193	TCA
P04223	H-2 class I histocompatibility antigen, K-K alpha chain OS=Mus musculus OX=10090 GN=H2-K1 PE=1 SV=1	H2-K1	5.23	-0.45				75406.40157		2826840.178	3288352.766	22164.33044	318821.3129	9519.887205			TCA
P01902	H-2 class I histocompatibility antigen, K-D alpha chain OS=Mus musculus OX=10090 GN=H2-K1 PE=1 SV=1	H2-K1	3.73	3.46	47239.35547	86915.04215	60221.10858	77940.04864	1237478.52	1010713.824	786714.0148	637636.0462	672198.7315	944094.024	592618.3243	768511.3412	DD
P01897	H-2 class I histocompatibility antigen, L-D alpha chain OS=Mus musculus OX=10090 GN=H2-L PE=1 SV=2	H2-L	4.05	4.17	85631.74524		73365.21434		1368214.375	822728.3828	1266861.147	1693411.784	1499074.601	1711687.58	1135750.731	1351872.825	TCA
P01897	H-2 class I histocompatibility antigen, L-D alpha chain OS=Mus musculus OX=10090 GN=H2-L PE=1 SV=2	H2-L	3.96	3.75	60967.59766				1138208.353	1060481.478	784091.9789	932431.8321	834070.8919	1076498.677	686240.6431	808482.344	DD
P01898	H-2 class I histocompatibility antigen, Q10 alpha chain OS=Mus musculus OX=10090 GN=H2-Q10 PE=1 SV=3	H2-Q10	-1.59	-1.55	68933493.05	74735097.56	73722088.88	70333036.07	36401963.21	36390781.81	26229722.04	39183857.23	29774075.87	28919916.88	27255132.14	25367812.44	TCA
P01898	H-2 class I histocompatibility antigen, Q10 alpha chain OS=Mus musculus OX=10090 GN=H2-Q10 PE=1 SV=3	H2-Q10	-1.08	-1.92	28633676.62	40483137.12	38004697.86	50281759.57	19456786.15	21302826.8	16560906.3	20187660.54	12351527.94	10648899.51	11079370.16	7438067.283	DTT
P01898	H-2 class I histocompatibility antigen, Q10 alpha chain OS=Mus musculus OX=10090 GN=H2-Q10 PE=1 SV=3	H2-Q10	-0.91	-1.08	46077334.16	39284714.67	37117998.14	31027424.34	24776368.1	19552762.89	18964149.61	12914868.6	18503159.55	19697309.93	16647335.24	17069073.72	DD
P14430	H-2 class I histocompatibility antigen, Q8 alpha chain OS=Mus musculus OX=10090 GN=H2-Q8 PE=3 SV=1	H2-Q8	3.12	3.97	119020.6003	71526.04904	74312.68056	63182.25236	960913	438488.4567	630723.02	1022079.275	1119881.078	1551242.436	990336.3089	1407483.529	TCA
P14430	H-2 class I histocompatibility antigen, Q8 alpha chain OS=Mus musculus OX=10090 GN=H2-Q8 PE=3 SV=1	H2-Q8	0.92	1.38	103106.5129	100230.5495	206464.6446	48764.39057	252561.1445		78212.77239	305063.3815	293188.4692	429568.5273	353327.8441	88909.20823	DTT
P14430	H-2 class I histocompatibility antigen, Q8 alpha chain OS=Mus musculus OX=10090 GN=H2-Q8 PE=3 SV=1	H2-Q8	3.82	3.73	94766.45313	126242.1898	110025.6094	137110.2363	1643195.011	1594412.753	1654925.119	1747855.597	1567467.961	1926517.992	1099871.332	1577439.457	DD
Q8K0D2	Hyaluronan-binding protein 2 OS=Mus musculus OX=10090 GN=Habp2 PE=1 SV=2	Habp2	-0.60	-0.79	565554.0678	450192.7128	355599.9097	352548.0326	284060.1406	163941.6511	331446.9507	257951.9791	267772.4078	202680.7445	346033.367	223390.71	TCA
Q8K0D2	Hyaluronan-binding protein 2 OS=Mus musculus OX=10090 GN=Habp2 PE=1 SV=2	Habp2	2.54	1.66	11874.38496		21082.45271		50759.69704		40985.56311	398033.2217	119276.5376	267517.7373	70929.38111	51561.6461	DTT
Q8K0D2	Hyaluronan-binding protein 2 OS=Mus musculus OX=10090 GN=Habp2 PE=1 SV=2	Habp2	-0.51	-0.72	491959.9063	515428.9897	348827.0394	386679.3716	259051.8583	290370.6696	231488.4126	341914.29	261783.9665	120163.3283	289405.5159	216808.7196	DD
Q80T19	Hepcidin-2 OS=Mus musculus OX=10090 GN=Hamp2 PE=2 SV=1	Hamp2	-2.20	-1.78	290924.342	396891.9419	585783.9078	242365.2335	73752.7904							98767.12035	DTT
P01942	Hemoglobin subunit alpha OS=Mus musculus OX=10090 GN=Hba PE=1 SV=2	Hba	1.07	0.95	774663892.2	199878500.8	421842053.8	190249719.7	673897096.9	505759104.1	406319936.3	617423874.1	374872348.4	397703985.8	670657588.9	1150502875	TCA
P01942	Hemoglobin subunit alpha OS=Mus musculus OX=10090 GN=Hba PE=1 SV=2	Hba	1.16	1.22	655657163.3	150663904.5	558337518.4	193717247.4	876089446.5	777238636.7	451448878.4	748195771.9	554952816.7	532876726.5	670297154.7	1220848978	DTT
P01942	Hemoglobin subunit alpha OS=Mus musculus OX=10090 GN=Hba PE=1 SV=2	Hba	1.14	0.98	550433661.3	102160815.1	286753414.5	118250751.5	440388281.4	442784685.1	499170869	340895482.4	279265435.3	286034927	503586385.5	908107013.4	DD
P02088	Hemoglobin subunit beta-1 OS=Mus musculus OX=10090 GN=Hbb-b1 PE=1 SV=2	Hbb-b1	0.70	1.04	1029240868	244770393.3	533699193.1	228955662.6	1098078393	359525998.2	347267271	704548923.8	587434976.7	646032765.6	864541642.3	1578984684	TCA
P02088	Hemoglobin subunit beta-1 OS=Mus musculus OX=10090 GN=Hbb-b1 PE=1 SV=2	Hbb-b1	1.34	0.74	390989778.8	76989323.53	251963592.9	113582964.4	535486988.7	631722894.1	353711427.9	455717318.5	258043856.8	209726748.5	407525290.7	553364147.6	DTT
P02088	Hemoglobin subunit beta-1 OS=Mus musculus OX=10090 GN=Hbb-b1 PE=1 SV=2	Hbb-b1	1.36	1.21	441999712.3	85806164.04	221895777.7	95269795.71	371196718.6	382707023.9	451615330.2	258327834.6	229035755.2	250430123.7	407986314.9	777976174.3	DD
P02089	Hemoglobin subunit beta-2 OS=Mus musculus OX=10090 GN=Hbb-b2 PE=1 SV=2	Hbb-b2	0.97	1.29	175540127.7	34977962.15	99329923.77	40998249.11	173996334.8	101748937.9	86189873	135672663	96658535.43	107666786.7	151418937	293747527	TCA
P02089	Hemoglobin subunit beta-2 OS=Mus musculus OX=10090 GN=Hbb-b2 PE=1 SV=2	Hbb-b2	1.55	0.75	85337429.25	21546124.67	44236231.8	26183575.42	102501880.9	75351342.44	92998805.5	103349143.2	59542637.78	41296969.22	75527996.28	132975750.1	DTT
P02089	Hemoglobin subunit beta-2 OS=Mus musculus OX=10090 GN=Hbb-b2 PE=1 SV=2	Hbb-b2	1.52	1.57	529533016.6	87247385.43	233573541.8	96704902.68	453391089.3	539344494.9	664324198.5	346585125.4	269199201.1	292481136.4	532012279.2	1038519721	DD
P02104	Hemoglobin subunit epsilon-Y2 OS=Mus musculus OX=10090 GN=Hbb-y PE=1 SV=2	Hbb-y	1.52	2.26	44923.70313				134171.7501	123535.9996	240793.6576	54777.67659				215795.9582	DD
Q9R098	Hepatocyte growth factor activator OS=Mus musculus OX=10090 GN=Hgfac PE=1 SV=1	Hgfac	-0.82	-0.99	2153281.564	3036533.723	2770255.636	2669014.216	1748536.221	1785883.771	1441812.533	1542664.125	1272770.721	1638498.363	1274619.739	1219501.905	TCA
Q9R098	Hepatocyte growth factor activator OS=Mus musculus OX=10090 GN=Hgfac PE=1 SV=1	Hgfac	-0.25	-0.52	59766.66613	82099.25568	30857.11382	108709.309			81603.15692	23759.9468		30102.81778	33190.87514		DTT
Q9R098	Hepatocyte growth factor activator OS=Mus musculus OX=10090 GN=Hgfac PE=1 SV=1	Hgfac	-0.33	-0.45	1800418.435	1951731.848	1800932.465	1568191.034	1754820.202	1945848.439	1640813.316	1460581.179	1206989.672	1495671.638	1492290.869	1343106.358	DD
P70349	Histidine triad nucleotide-binding protein 1 OS=Mus musculus OX=10090 GN=Hint1 PE=1 SV=3	Hint1	0.30	0.43	147370.7266	101194.5714	75850.68097	163864.282	119980.5764		139568.767	188249.2583	151721.1897	92260.9585	155405.4605	279709.8719	DTT
Q9ERH7	Homeodomain-interacting protein kinase 3 OS=Mus musculus OX=10090 GN=Hipk3 PE=1 SV=3	Hipk3	-2.05	-1.13	4951189.338	253601.3996	662246.2225	1009406.675	1077569.313	177361.4275	25656.76229	320447.2048	96922.06355			2704499.417	DD
P84228	Histone H3.2 OS=Mus musculus OX=10090 GN=Hist1h3b PE=1 SV=2	Hist1h3b	6.64	6.64						84665.89311						6858.702559	DD
P62806	Histone H4 OS=Mus musculus OX=10090 GN=Hist1h4a PE=1 SV=2	Hist1h4a	6.64	6.64					185520.4141	974458.1931	232614.6818	92970.08265	136282.9009	109307.2996		230130.2488	DD
Q64523	Histone H2A type 2-C OS=Mus musculus OX=10090 GN=Hist2h2ac PE=1 SV=3	Hist2h2ac	1.24	0.59	36732.19141				73880.34878	285001.6698	65850.06927	102395.9892	62520.89971	58986.92038	29342.68964	51610.60542	DD
Q61646	Haptoglobin OS=Mus musculus OX=10090 GN=Hp PE=1 SV=1	Hp	-3.40	-3.80	75173759.12	85531162.4	35865518.16	56247462.94	5303308.756	5586109.564	6114816.833	5424768.705	5060213.586	5632982.025	2971262.336	4364620.23	TCA
Q61646	Haptoglobin OS=Mus musculus OX=10090 GN=Hp PE=1 SV=1	Hp	-2.82	-3.43	25158777.73	12440324.7	8058625.369	9438915.862	591275.2558	323055.1016	1780935.165	907859.3281	478216.2984	577095.4634	285091.4515	303114.6876	DTT
Q61646	Haptoglobin OS=Mus musculus OX=10090 GN=Hp PE=1 SV=1	Hp	-2.73	-3.35	24487545.55	21425583.55	9198642.544	18792159.75	1820682.992	3187662.431	2392771.078	2678282.716	1639391.936	1970048.19	1034678.181	1625638.399	DD
Q91X72	Hemopexin OS=Mus musculus OX=10090 GN=Hpx PE=1 SV=2	Hpx	1.83	1.58	594639003.8	659736582.7	526791062.9	549495762.6	1846314748	2151143367	2422695782	1996861252	1889868139	1833010452	1839616104	1646069514	TCA
Q91X72	Hemopexin OS=Mus musculus OX=10090 GN=Hpx PE=1 SV=2	Hpx	1.33	0.89	54814807.66	82026844.63	51346391.33	69177397.77	154110057	277210301.7	272399093.4	130835977.8	92161908.99	155084567.1	263399460.8	34365110.89	DTT
Q91X72	Hemopexin OS=Mus musculus OX=10090 GN=Hpx PE=1 SV=2	Hpx	1.92	1.77	489082332.7	492956539.5	375561167.9	455596041.1	1677092294	1808483535	1740545981	1886089006	1492605438	1497244428	1531661004	1447306759	DD
Q9ESB3	Histidine-rich glycoprotein OS=Mus musculus OX=10090 GN=Hrg PE=1 SV=2	Hrg	1.80	1.50	11699846.9	14105915.09	1517959.511	12366247.31	23921018.56	33011638.47	61816213.86	31069692.99	29416639.07	23185656.41	43845649.85	34508549.84	TCA
Q9ESB3	Histidine-rich glycoprotein OS=Mus musculus OX=10090 GN=Hrg PE=1 SV=2	Hrg	1.48	1.76	1851212.632	2512402.389	785548.2465	2617408.937	3250778.363	15137965.36	7255072.216	5550318.398	5776759.432	5448315.567	9092801.897	8726516.437	DTT
Q9ESB3	Histidine-rich glycoprotein OS=Mus musculus OX=10090 GN=Hrg PE=1 SV=2	Hrg	1.41	1.30	12402563.18	13268181.03	1806679.714	11842094.38	22471576.86	20293525.53	26546070.71	47512483.68	27137084.59	20481505.89	37409202.16	30302012.67	DD
P07901	Heat shock protein HSP 90-alpha OS=Mus musculus OX=10090 GN=Hsp90aa1 PE=1 SV=4	Hsp90aa1	6.64	6.64					446895.4844	326734.3703	373828.6129	270707.5854	62853.53188	169480.4041	799583.7203	151354.3562	TCA
P07901	Heat shock protein HSP 90-alpha OS=Mus musculus OX=10090 GN=Hsp90aa1 PE=1 SV=4	Hsp90aa1	6.64	6.64						500532.5065	63702.03947			281597.3753			DD
Q61316	Heat shock 70 kDa protein 4 OS=Mus musculus OX=10090 GN=Hspa4 PE=1 SV=1	Hspa4	-0.09	0.57	732059.0893	493167.5418	477579.3872	167042.6341	870801.043	75389.3417	108177.93	714380.6463	517224.5466	1035165.625	820346.5694	798205.2891	TCA
P20029	Endoplasmic reticulum chaperone BiP OS=Mus musculus OX=10090 GN=Hspa5 PE=1 SV=3	Hspa5	1.54	1.82	77331.51046	85761.13727	71304.75966	57232.06894	184361.0781	210723.1494	240524.1917	258657.6782	361878.1241	282640.4528	240269.9155	428603.4492	TCA
P20029	Endoplasmic reticulum chaperone BiP OS=Mus musculus OX=10090 GN=Hspa5 PE=1 SV=3	Hspa5	6.64	6.64							77872.07516	120994.3425	68536.0709	200861.3012	125391.5949		DTT
P63017	Heat shock cognate 71 kDa protein OS=Mus musculus OX=10090 GN=Hspa8 PE=1 SV=1	Hspa8	3.87	3.37	968038.4778	44093.03362	451481.9129	471781.9596	5944775.066	8196404.958	7900875.191	6813852.684	6977207.691	6845385.836	3725824.929	7497903.623	TCA
P63017	Heat shock cognate 71 kDa protein OS=Mus musculus OX=10090 GN=Hspa8 PE=1 SV=1	Hspa8	3.36	2.98	488108.1094	75055.26947	135682.7089	259095.4758	3457406.987	5378944.909	5176668.551	3670721.822	3609188.279	4273459.045	1883222.192	4797746.71	DD
Q9DBD0	Inhibitor of carbonic anhydrase OS=Mus musculus OX=10090 GN=Ica PE=1 SV=1	Ica	0.54	0.54	44226177.81	42219399.04	41299172.14	54491287.24	54629872.11	75913962	79057848.23	52957894.34	64484991.48	66674540.56	66502759.73	58339893.52	TCA
Q9DBD0	Inhibitor of carbonic anhydrase OS=Mus musculus OX=10090 GN=Ica PE=1 SV=1	Ica	-1.00	-0.24	3131620.976	3869678.9	2843843.429	3715481.678	798000.7888	1200329.723	2347465.432	1255046.103	2079431.947	5175642.336	7512210.17	401848.7416	DTT
Q9DBD0	Inhibitor of carbonic anhydrase OS=Mus musculus OX=10090 GN=Ica PE=1 SV=1	Ica	0.40	0.56	36961411.05	34942268.64	36341897.21	36186448.67	41580588.93	52246641.05	44266287.71	48748102.02	51525548.46	53893308.37	53888220.83	61062055.61	DD
O35664	Interferon alpha/beta receptor 2 OS=Mus musculus OX=10090 GN=Ifnar2 PE=1 SV=2	Ifnar2	-0.39	-1.25	73391.70313	17996.55069	41964.74228	49978.84505	31950.72369	37856.32666	13730.22295	86545.10944	43375.80321	17435.0612	17840.22563	9174.687641	DD
P70389	Insulin-like growth factor-binding protein complex acid labile subunit OS=Mus musculus OX=10090 GN=Igfals PE=1 S	Igfals	-2.59	-2.35	3251365.877	2852430.588	2741572.059	2525176.15	337602.6641	254041.1402	83534.07295	326296.6543	467226.9847	361215.0308	678796.4027	108400.6048	TCA
P70389	Insulin-like growth factor-binding protein complex acid labile subunit OS=Mus musculus OX=10090 GN=Igfals PE=1 S	Igfals	-6.64	-6.64	37404.61781	58760.50859	27614.15624	64743.96418									DTT
P70389	Insulin-like growth factor-binding protein complex acid labile subunit OS=Mus musculus OX=10090 GN=Igfals PE=1 S	Igfals	-2.77	-2.78	2806830.844	2191311.853	2055282.097	2154392.919	185457.9152	320694.4463	172538.5544	131396.6865	297906.1356	166978.1796	340085.1047	126076.5931	DD
P47876	Insulin-like growth factor-binding protein 1 OS=Mus musculus OX=10090 GN=Igfbp1 PE=2 SV=2	Igfbp1	6.64	#VALUE!							171134.6183	71293.77127					DTT
P47877	Insulin-like growth factor-binding protein 2 OS=Mus musculus OX=10090 GN=Igfbp2 PE=1 SV=2	Igfbp2	-6.64	-6.64	29696.05317		28377.46782										DTT
P47878	Insulin-like growth factor-binding protein 3 OS=Mus musculus OX=10090 GN=Igfbp3 PE=2 SV=2	Igfbp3	-2.62	-1.38	260534.1426	85354.36945	282499.5566	191284.8733	36298.65624				128866.0503	104193.9841	36072.24587	16408.24006	DTT
P47878	Insulin-like growth factor-binding protein 3 OS=Mus musculus OX=10090 GN=Igfbp3 PE=2 SV=2	Igfbp3	-0.73	-1.56	122139.7578	136500.8895	116673.6307	127936.0572				75241.29898	49076.61673		37441.49027		DD
P47879	Insulin-like growth factor-binding protein 4 OS=Mus musculus OX=10090 GN=Igfbp4 PE=1 SV=2	Igfbp4	0.53	0.63	228942.3586	230969.6277	159253.816	120516.3783	64871.10536		280281.9762	393283.4745	330915.3972	436001.7136	265123.7015	95904.62272	DTT
P01872	Immunoglobulin heavy constant mu OS=Mus musculus OX=10090 GN=Ighm PE=1 SV=2	Ighm	0.43	0.10	98617947.23	99246996.78	115205326	108880995.9	135345926.9	106157966.3	123971521.9	95401482.69	111253620.2	139817317.4	131648331.6	94027335.17	DD
P01837	Immunoglobulin kappa constant OS=Mus musculus OX=10090 GN=Igkc PE=1 SV=2	Igkc	1.25	2.76	7896101.607	9246627.836	9490793.426	9789381.232	28472418.87	19028317.14	27042620.95	20331762.86	49592276.81	62175374.23	70314005.79	30159212.26	DD
Q9Z0M9	Interleukin-18-binding protein OS=Mus musculus OX=10090 GN=Il18bp PE=1 SV=2	Il18bp	6.64	6.64					709497.0625	969135.1586	1357147.842	895473.2498	651940.0057	705431.4869	491632.3102	389808.6121	TCA

Q9Z0M9	Interleukin-18-binding protein OS=Mus musculus OX=10090 GN=Il18bp PE=1 SV=2	Il18bp	5.23	5.06	60078.49559	109400.0947	74140.4975	42780.64047	2621008.435	2752935	2256737.042	5462838.024	4406834.193	4725205	2588558.793	6262221.384	DTT
Q61730	Interleukin-1 receptor accessory protein OS=Mus musculus OX=10090 GN=Il1rap PE=1 SV=1	Il1rap	-1.64	-1.71	3555326.618	3596893.973	4755456.971	6086585.436	1782384.053	1512039.964	1949908.623	1747294.793	1243213.413	1883887.699	1817548.243	1142826.306	TCA
Q61730	Interleukin-1 receptor accessory protein OS=Mus musculus OX=10090 GN=Il1rap PE=1 SV=1	Il1rap	-1.65	-1.64	449388.5058	1139312.171	632157.151	1304590.587	42507.41678				27329.03708	207596.7112		47793.69848	DTT
Q61730	Interleukin-1 receptor accessory protein OS=Mus musculus OX=10090 GN=Il1rap PE=1 SV=1	Il1rap	-0.98	-1.34	4327583.641	5581318.319	5102577.787	6127819.544	1709632.862	2150833.357	1428192.404	1209959.244	1949182.047	1600361.533	2609976.823	1992239.947	DD
O55023	Inositol monophosphatase 1 OS=Mus musculus OX=10090 GN=Impa1 PE=1 SV=1	Impa1	1.29	2.76	15395.23696				42577.10326	162499.3438	33461.61517		89768.13747	309532.1482	199806.9146	84233.97457	DTT
Q61702	Inter-alpha-trypsin inhibitor heavy chain H1 OS=Mus musculus OX=10090 GN=Itih1 PE=1 SV=2	Itih1	-0.36	-0.78	22428405.97	25670634.49	29384922.57	28657106.78	17417725.01	31986927.49	27807088.54	19695855.73	20281220.86	28532049.67	26829104.24	18434395.76	TCA
Q61702	Inter-alpha-trypsin inhibitor heavy chain H1 OS=Mus musculus OX=10090 GN=Itih1 PE=1 SV=2	Itih1	-1.58	-1.24	2099432.389	3578597.578	3780159.38	3997180.537	654962.3808	841334.8984	536811.5756	682283.5293	478769.6303	1096160.353	1687016.276	176658.1549	DTT
Q61702	Inter-alpha-trypsin inhibitor heavy chain H1 OS=Mus musculus OX=10090 GN=Itih1 PE=1 SV=2	Itih1	-0.35	-0.66	18485997.03	19242911.55	18370428.81	19683436.29	12898465.15	17844044.33	15052122.91	14920705.13	14549499.52	17228771.54	17113447.45	14576640.51	DD
Q61703	Inter-alpha-trypsin inhibitor heavy chain H2 OS=Mus musculus OX=10090 GN=Itih2 PE=1 SV=1	Itih2	0.08	0.11	43590510.43	49949490.09	53774467.52	49595488.09	48901393.75	60217616.91	54333580.21	45111670.12	59717815.83	66918834.97	61439571.58	44737930.07	TCA
Q61703	Inter-alpha-trypsin inhibitor heavy chain H2 OS=Mus musculus OX=10090 GN=Itih2 PE=1 SV=1	Itih2	-0.64	-0.50	3996660.821	6745883.022	6139335.719	8007419.815	3822620.062	2808592.545	5501547.187	1540680.65	2897237.545	4444538.832	7211046.176	569030.6411	DTT
Q61703	Inter-alpha-trypsin inhibitor heavy chain H2 OS=Mus musculus OX=10090 GN=Itih2 PE=1 SV=1	Itih2	-0.16	0.19	35474536.72	36174293.91	34225280.55	34971863.08	34401581.24	36418794.08	34074038.07	33015314.87	46674657.1	48463138.69	40225872.78	29551090.61	DD
Q61704	Inter-alpha-trypsin inhibitor heavy chain H3 OS=Mus musculus OX=10090 GN=Itih3 PE=1 SV=3	Itih3	2.20	2.21	41637350.24	51445292.97	44992037.03	45813510.75	154888886.4	146081981.6	139948036	146674151.3	177182525.1	162851975.8	153655884.6	147850065.8	TCA
Q61704	Inter-alpha-trypsin inhibitor heavy chain H3 OS=Mus musculus OX=10090 GN=Itih3 PE=1 SV=3	Itih3	2.38	1.88	2707071.876	8386143.608	2718917.78	7640355.61	18062767.87	16767363.73	27383666.91	17366497.6	16047427.18	13844439.4	22673722.24	15245230.69	DTT
Q61704	Inter-alpha-trypsin inhibitor heavy chain H3 OS=Mus musculus OX=10090 GN=Itih3 PE=1 SV=3	Itih3	2.49	2.55	16605246.32	20294829.02	11993568.37	13343317.34	77214980.46	85679710.22	90506079.47	88940980.09	105137206.5	99842438.92	86247447.27	79560775.27	DD
A6X935	Inter alpha-trypsin inhibitor, heavy chain 4 OS=Mus musculus OX=10090 GN=Itih4 PE=1 SV=2	Itih4	1.39	0.93	86959635.68	101838811.5	98109023.3	92990312.84	197684765.4	219616570.7	290225002.2	207231971.9	199916891.3	193948173.1	163541057.1	155492543.3	TCA
A6X935	Inter alpha-trypsin inhibitor, heavy chain 4 OS=Mus musculus OX=10090 GN=Itih4 PE=1 SV=2	Itih4	0.34	0.00	8790132.267	11610177.28	11336482.46	10241627.68	15424408.12	12493043.7	17008831.88	11031991.15	8022448.627	13647701.21	19151067.33	3769393.734	DTT
A6X935	Inter alpha-trypsin inhibitor, heavy chain 4 OS=Mus musculus OX=10090 GN=Itih4 PE=1 SV=2	Itih4	1.34	0.93	61917197.53	68831803.3	54597404.68	55405944.13	119351782.1	135121278	141395140.3	157822972.4	126128353	120794195	98252998.98	102337569.5	DD
P01592	Immunoglobulin J chain OS=Mus musculus OX=10090 GN=Jchain PE=1 SV=4	Jchain	0.84	0.61	1273887.25	1749567.274	1868918.049	1873129.196	2964244.322	2249762.322	2929969.123	2829782.32	2086171.47	2759276.119	2771770.598	1653974.809	DD
Q02257	Junction plakoglobin OS=Mus musculus OX=10090 GN=Jup PE=1 SV=3	Jup	-6.64	-6.64	3223.902588	36012.14263											DD
P61110	Kidney androgen-regulated protein OS=Mus musculus OX=10090 GN=Kap PE=2 SV=1	Kap	-2.20	-3.66	116060.8158	338351.9566	361447.8855	281368.807	42715			75993.00825	14365.05877	14756.48194	24271.5729	35488.58618	TCA
P61110	Kidney androgen-regulated protein OS=Mus musculus OX=10090 GN=Kap PE=2 SV=1	Kap	-4.29	-1.96	20915.73361	128530.6348	183545.6104	146154.346			6948.841774					35272.41928	DTT
P26262	Plasma kallikrein OS=Mus musculus OX=10090 GN=Klkb1 PE=1 SV=2	Klkb1	-1.37	-1.12	17810758.44	21515240.43	19206551.6	22550325.42	8371570.914	6087815.167	5511938.991	8096546.558	9236063.417	8602884.915	9008261.639	8216415.763	TCA
P26262	Plasma kallikrein OS=Mus musculus OX=10090 GN=Klkb1 PE=1 SV=2	Klkb1	-2.44	-1.50	1250534.719	1753610.159	1450385.333	2338939.792	107106.8522		536497.7317	220353.2105	265657.5586	583630.4475	430090.7473	20146.99494	DTT
P26262	Plasma kallikrein OS=Mus musculus OX=10090 GN=Klkb1 PE=1 SV=2	Klkb1	-1.28	-1.33	14378000.3	17143809.97	14561251.75	18037312.82	6212104.397	7411315.817	6350374.841	5468219.135	6860336.309	7086491.927	6318182.364	6777062.418	DD
O08677	Kininogen-1 OS=Mus musculus OX=10090 GN=Kng1 PE=1 SV=1	Kng1	-0.04	0.12	265545985.4	335187627.8	230798020.4	295812881.1	287292942	286019101.5	356395800.1	328121585.5	345383585.6	328685516.8	349090030.1	285652545.5	TCA
O08677	Kininogen-1 OS=Mus musculus OX=10090 GN=Kng1 PE=1 SV=1	Kng1	-0.20	0.04	29044705.92	44023483.88	39048682.53	40105867.36	25891839.71	32068368.61	47943217.67	30832566.48	32806015.93	44736921.03	58827324.27	20461547.2	DTT
O08677	Kininogen-1 OS=Mus musculus OX=10090 GN=Kng1 PE=1 SV=1	Kng1	0.47	0.47	161794877.4	187560101	143936879.1	171142105	203622037.5	221761235	204192494	262592239.9	253029600.8	234336363.9	260995940.7	231801463.6	DD
P04104	Keratin, type II cytoskeletal 1 OS=Mus musculus OX=10090 GN=Krt1 PE=1 SV=4	Krt1	2.27	3.33	542095.1502	270289.7683	514829.0324	294139.6355	2028515.303	863223.6094	2048700.155	2623620.666	2699367.689	2158067.522	3079100.934	6314013.533	DTT
P04104	Keratin, type II cytoskeletal 1 OS=Mus musculus OX=10090 GN=Krt1 PE=1 SV=4	Krt1	-1.86	-1.28	1835027.711	40912852.97	9183888.807	3874849.564	2532498.69	831180.0635	2237273.388	2206517.741	1895049.911	2203288.973	9721070.463	1381154.936	DD
P02535	Keratin, type I cytoskeletal 10 OS=Mus musculus OX=10090 GN=Krt10 PE=1 SV=3	Krt10	-0.14	-0.16	322276.3861	439134.6007	632476.0802	471417.7036	299254.3281	150567.1085	244151.1753	831730.1779	325255.9561	383071.3617	319593.568	386778.1028	TCA
P02535	Keratin, type I cytoskeletal 10 OS=Mus musculus OX=10090 GN=Krt10 PE=1 SV=3	Krt10	3.12	3.81	126034.756	219687.4066	335976.0242	255793.0729	3735683.292	505474.4688	2231330.595	6616916.316	4590828.002	4867350.464	4894835.196	10695001.78	DTT
P02535	Keratin, type I cytoskeletal 10 OS=Mus musculus OX=10090 GN=Krt10 PE=1 SV=3	Krt10	-1.52	-0.64	2059063.367	7901874.823	9119239.044	3311897.336	2639472.25	611792.1741	1519338.061	2045416.925	1872931.696	2237792.156	11115742.98	1517655.834	DD
P08730	Keratin, type I cytoskeletal 13 OS=Mus musculus OX=10090 GN=Krt13 PE=1 SV=2	Krt13	2.81	3.98	81305.88846	27027.99873	72409.6506	45554.09051	400339.238	110783.9297	404685.4095	1114599.403	638103.4427	688489.8681	748442.0592	1946837.292	DTT
P08730	Keratin, type I cytoskeletal 13 OS=Mus musculus OX=10090 GN=Krt13 PE=1 SV=2	Krt13	-6.64	0.16	210601.6328	1132565.531	200435.3649	42951.25072					101557.7205		601557.4872		DD
Q61781	Keratin, type I cytoskeletal 14 OS=Mus musculus OX=10090 GN=Krt14 PE=1 SV=2	Krt14	-1.70	-2.65		943918.2542	2864906.205	118929.6667	130916.0554		364513.8367	37041.31738	178268.832	86408.74517	194155.7328		DD
Q9Z2K1	Keratin, type I cytoskeletal 16 OS=Mus musculus OX=10090 GN=Krt16 PE=1 SV=3	Krt16	-2.12	-1.20	643709.1035	8377691.41	14609326.18	1597057.499	770702.0612	157300.6844	628800.0977	394329.0764	735385.6555	746897.3922	8749981.48	263307.7958	DD
Q3TTY5	Keratin, type II cytoskeletal 2 epidermal OS=Mus musculus OX=10090 GN=Krt2 PE=1 SV=1	Krt2	3.38	4.19	32846.44365	32644.16754	36567.92989	24385.34006	595866.6696	63483.50781	525431.1595	1299835.458	1031159.851	906158.9553	885050.8366	1753604.135	DTT
Q3TTY5	Keratin, type II cytoskeletal 2 epidermal OS=Mus musculus OX=10090 GN=Krt2 PE=1 SV=1	Krt2	-1.72	-1.27	415814.5977	8170305.046	2965457.927	1152727.172	550441.3646	106512.6249	389389.1665	608116.9581	320937.1423	684894.7238	5075835.827	285369.1561	DD
Q6IFX2	Keratin, type I cytoskeletal 42 OS=Mus musculus OX=10090 GN=Krt42 PE=1 SV=1	Krt42	-0.92	0.68	20177.27653		30393.31952		13108.33627					17382.27438		91073.27435	DTT
Q6IFX2	Keratin, type I cytoskeletal 42 OS=Mus musculus OX=10090 GN=Krt42 PE=1 SV=1	Krt42	-4.41	-4.24			612976.3146		28872.63573						44061.41307	23596.25986	DD
Q922U2	Keratin, type II cytoskeletal 5 OS=Mus musculus OX=10090 GN=Krt5 PE=1 SV=1	Krt5	-0.65	-1.44	75960.69465	579299.4382	338136.9328	968850.5393	216239.4688	108352.3223	176067.3695	517949.3162	120425.5265	87399.51302	167944.834	136416.0838	TCA
Q922U2	Keratin, type II cytoskeletal 5 OS=Mus musculus OX=10090 GN=Krt5 PE=1 SV=1	Krt5	2.24	2.85	221058.6275	78150.7415	129975.8228	112089.7693	789360.9458	761423.0625	429001.6057	897988.0892	991467.65	963455.2427	918518.6067	2700603.972	DTT
Q922U2	Keratin, type II cytoskeletal 5 OS=Mus musculus OX=10090 GN=Krt5 PE=1 SV=1	Krt5	-2.24	-1.57	342979.8135	9382878.238	1304739.495	791879.1854	737373.3318	174020.3687	651114.2233	241984.1539	363587.5704	352192.038	1429842.749	124095.6413	DD
P50446	Keratin, type II cytoskeletal 6A OS=Mus musculus OX=10090 GN=Krt6a PE=1 SV=3	Krt6a	-1.86	-0.89	1065305.35	12064817.31	6909685.974	1269602.751	842517.2161	304435.331	628748.5993	789598.8172	815156.0733	773867.9414	1511994.277	595357.0509	DD
Q6IME9	Keratin, type II cytoskeletal 72 OS=Mus musculus OX=10090 GN=Krt72 PE=3 SV=1	Krt72	-6.64	-0.35		306789.7108	80129.16925								122815.5081		DD
Q6NXH9	Keratin, type II cytoskeletal 73 OS=Mus musculus OX=10090 GN=Krt73 PE=1 SV=1	Krt73	-2.08	-1.62	74250.92188	942557.5327	147273.8732	158315.3705	32861.60782	25866.46359	39722.69286	47784.84911	49265.6644	42507.98493	101723.1403	50009.64113	DD
Q3UV17	Keratin, type II cytoskeletal 2 oral OS=Mus musculus OX=10090 GN=Krt76 PE=1 SV=1	Krt76	2.29	3.14	130733.0025	39128.78558	106645.2531	121188.0057	547745.4722	560883.625			1123351.453	1100992.739	571760.5665	550509.018	DTT
Q3UV17	Keratin, type II cytoskeletal 2 oral OS=Mus musculus OX=10090 GN=Krt76 PE=1 SV=1	Krt76	-1.93	-3.01	663732.3125	17694965.48	3979183.643	1336178.258	1032915	176386.9211	911217.7768	1107416.264	456848.6682	529634.6583	1314172.653	19960.30339	DD
Q8VED5	Keratin, type II cytoskeletal 79 OS=Mus musculus OX=10090 GN=Krt79 PE=1 SV=2	Krt79	6.64	6.64					158885.53	195107.8594		140977.9676				395720.5001	DTT
Q8VED5	Keratin, type II cytoskeletal 79 OS=Mus musculus OX=10090 GN=Krt79 PE=1 SV=2	Krt79	-2.28	-0.90	326172.4688	1862036.553	1964129.239	471627.9854	422402.1558	64183.07758	213410.6985	230895.9633	291068.9143		1933509.919	151506.943	DD
Q3V2T4	Keratinocyte differentiation-associated protein OS=Mus musculus OX=10090 GN=Krtdap PE=2 SV=1	Krtdap	-3.41	-4.11	2194126.737	2289644.382	5065796.652	1685793.096	325299.3675	362545	135492.0662	206436.9317	191870.6779	90651.97182	354465.5674	99080.12066	DTT
Q3V2T4	Keratinocyte differentiation-associated protein OS=Mus musculus OX=10090 GN=Krtdap PE=2 SV=1	Krtdap	-3.06	-3.18	165842.3125	126199.325	140164.7364	145511.8461	17112.12066	22475.26351		13698.6369	16100.0966	15220.54061	26243.6706	8743.587986	DD
Q9CPY7	Cytosol aminopeptidase OS=Mus musculus OX=10090 GN=Lap3 PE=1 SV=3	Lap3	2.75	1.46	23386.21442	44026.44677	36018.28304		374543.0039	689085.8784	609563.2787	407664.599	340905.8041	294435.9245	272107.9399	290548.0658	TCA
Q61805	Lipopolysaccharide-binding protein OS=Mus musculus OX=10090 GN=Lbp PE=1 SV=2	Lbp	2.43	2.63	178359.0043	369108.8278	227778.9527	147770.4564	2160401.855	1276768.386	1628516.71	2134885.599	2295729.447	2740601.685	1610627.101	1582623.271	TCA
Q61805	Lipopolysaccharide-binding protein OS=Mus musculus OX=10090 GN=Lbp PE=1 SV=2	Lbp	2.54	2.14	115396.2773	189682.5781	217978.7423	186022.6965	1310699.004	1827334.234	1553529.231	1496968.949	1200352.892	1575176.782	932451.7762	834789.4579	DD
P16301	Phosphatidylcholine-sterol acyltransferase OS=Mus musculus OX=10090 GN=Lcat PE=1 SV=2	Lcat	0.55	0.63	50294286.03	54753341.91	75638920.19	58685495.16	58035047.35	68669263.58	62299419.18	56513740.96	63214352.9	54384244.43	53150163.69	46911373.64	TCA
P16301	Phosphatidylcholine-sterol acyltransferase OS=Mus musculus OX=10090 GN=Lcat PE=1 SV=2	Lcat	0.31	0.70	42304897.37	45498797.13	39659892.84	37751551.29	13576803.72	13370226.45	28932206.94	45696800.43	29515913.36	26475771.25	21093420.01	24212922.42	DTT
P16301	Phosphatidylcholine-sterol acyltransferase OS=Mus musculus OX=10090 GN=Lcat PE=1 SV=2	Lcat	1.82	1.09	2480363.919	2780598.589	2456780.155	2412213.408	8085477.227	7663657.304	9710880.652	8256508.374	5975843.039	6190730.65	4655362.548	3978644.641	DD
P11672	Neutrophil gelatinase-associated lipocalin OS=Mus musculus OX=10090 GN=Lcn2 PE=1 SV=1	Lcn2	6.64	6.64					2915010.25	861434.9044	4236590.427	2707074.115	426304.1676	375099.562	187921.1032	370648.0494	TCA
P11672	Neutrophil gelatinase-associated lipocalin OS=Mus musculus OX=10090 GN=Lcn2 PE=1 SV=1	Lcn2	6.64	6.64							224077.9789	52744.94579				22340.77199	DTT
P11672	Neutrophil gelatinase-associated lipocalin OS=Mus musculus OX=10090 GN=Lcn2 PE=1 SV=1	Lcn2	3.14	1.38	48260.21094	66444.9649			2026640.383	1877508.829	1672083.733	4338117.213	345244.9754	550345.9346	122508.3894	314126.3051	DD
Q61233	Plastin-2 OS=Mus musculus OX=10090 GN=Lcp1 PE=1 SV=4	Lcp1	1.79	0.39	134376.6534	207723.869	104895.0659	140985.5642	603816.2344	797478.9231	667986.4434	812241.519	500520.6649	263792.0692	361367.4205	244400.9109	TCA
Q61233	Plastin-2 OS=Mus musculus OX=10090 GN=Lcp1 PE=1 SV=4	Lcp1	6.64	6.64					414381.0111	474243.1633	407783.3255	443446.6246	279098.7698	324333.2667	241443.4477	293403.7022	DD
P06151	L-lactate dehydrogenase A chain OS=Mus musculus OX=10090 GN=Ldha PE=1 SV=3	Ldha	3.15	2.96	467970.3615	75158.54834	175456.4794	257077.3142	4921337.742	5163982.256	4891951.424	4495958.07	3647249.305	3258291.06	3695861.956	4497391.882	TCA
P06151	L-lactate dehydrogenase A chain OS=Mus musculus OX=10090 GN=Ldha PE=1 SV=3	Ldha	3.10	2.62	290441.3496		85029.3463	162122.4594	2731306.273	4695694.128	2899935.926	4242646.235	3120848.964	3508345.408	2215372.535	3837250.658	DD
P16125	L-lactate dehydrogenase B chain OS=Mus musculus OX=10090 GN=Ldhb PE=1 SV=2	Ldhb	1.56	1.12	222728.4054	80977.70983	108809.1786	203952.3337	1494667.219	436509.2651	287450.5156	397840.0501	241017.7328	276696.6426	333769.991	263913.1308	TCA
P16125	L-lactate dehydrogenase B chain OS=Mus musculus OX=10090 GN=Ldhb PE=1 SV=2	Ldhb	-0.40	-0.64	10846322.95	12666409.07	12088508.36	12894510.13	10767591.77	6645714.702	7809932.212	10303658.08	8418114.711	9151279.477	6825454.723	1736848.77	DD
P00342	L-lactate dehydrogenase C chain OS=Mus musculus OX=10090 GN=Ldhc PE=1 SV=2	Ldhc	4.32	3.23	18583.18181	5265.674298			173240.7325	224123.375			68566.94081	145877.8736	144351.262	58772.52999	DTT
Q8C6C9	Protein LEG1 homolog OS=Mus musculus OX=10090 GN=Leg1 PE=2 SV=2	Leg1	6.64	6.64					40077.52453			136380.3564	122522.4599	90019.77853		172028.1426	DTT
P16045	Galectin-1 OS=Mus musculus OX=10090 GN=Lgals1 PE=1 SV=3	Lgals1	1.61	0.57	114720.6406	65566.89505	165748.8307	15065.88339	699883.865		71393.00188	253255.2439	20136.49953	110469.2813	147692.9419	241469.7184	DTT
P42703	Leukemia inhibitory factor receptor OS=Mus musculus OX=10090 GN=Lifr PE=1 SV=1	Lifr	-2.63	-4.01	11995057.45	17317181.34	16676419.39	19443628.65	2491152.711	1645841.866	1250233.585	2582813.727	977019.0286	887158.1639	711181.2551	762989.276	TCA
P42703	Leukemia inhibitory factor receptor OS=Mus musculus OX=10090 GN=Lifr PE=1 SV=1	Lifr	-2.72	-1.97	755935.4206	1135220.833	1205356.585	685704.2	32133.02657	23275.06836	26890.73341	21618.19233		48794.58202			DTT
P42703	Leukemia inhibitory factor receptor OS=Mus musculus OX=10090 GN=Lifr PE=1 SV=1	Lifr	-2.05	-3.61	9260455.648	9790133.071	8098544.22	9670017.053	1552409.013	2153979.415	1738071.113	1305889.076	746335.4247	796959.7408	627957.2587	603712.2355	DD
P97873	Lysyl oxidase homolog 1 OS=Mus musculus OX=10090 GN=Loxl1 PE=2 SV=3	Loxl1	-1.31	-1.57	224514.7218	24792.29796	271717.3983	85544.14917	46697.48783			66535.41928				46570.71244	DTT
Q8CIM1	Leucine-rich repeat-containing protein 45 OS=Mus musculus OX=10090 GN=Lrrc45 PE=2 SV=2	Lrrc45	-0.07	0.38	63033.80469	52351.1853	49695.11619	44915.04836	58936.08846	60856.30596	35390.50189	46025.27568	67816.94843	59846.1819	85787.53626	58637.43101	DD
P51885	Lumican OS=Mus musculus OX=10090 GN=Lum PE=1 SV=2	Lum	-0.19	-1.06	8239851.945	7827572.506	6847224.866	8237499.057	7529645.273	5255816.561	5992457.95	17465583.16	7627398.574	10056048.78	9017268.921	7500393.403	TCA
P51885	Lumican OS=Mus musculus OX=10090 GN=Lum PE=1 SV=2	Lum	-0.79	-2.43	827138.8738	564851.1033	3157176.538	551169.8125	309861.1428	270249.1797	37382.87139	105808.9291	31656.61643	29715.02452	37575.25007	236504.1711	DTT
P51885	Lumican OS=Mus musculus OX=10090 GN=Lum PE=1 SV=2	Lum	-0.76	-1.35	2443908.414	2840827.914	2301784.918	2575842.808	1919369.746	1811774.073	1340089.601	1793250.093	1078515.2	869832.7628	915743.4577	1484960.845	DD
P45700	Mannosyl-oligosaccharide 1,2-alpha-mannosidase IA OS=Mus musculus OX=10090 GN=Man1a1 PE=1 SV=1	Man1a1	-0.63	-1.54	66727.58417		109126.217	49740.39126			10696.65602	212213.3096	27604.49288	19085.44406	22943.31631		DTT
P26645	Myristoylated alanine-rich C-kinase substrate OS=Mus musculus OX=10090 GN=Marcks PE=1 SV=2	Marcks	0.04	-0.83	94366.61278	174154.1217	167217.5073	85302.4973	192358.7578	28128.51157	40038.89622	202819.3289	66708.52484	54934.84058	53039.23205	118538.5436	TCA
P26645	Myristoylated alanine-rich C-kinase substrate OS=Mus musculus OX=10090 GN=Marcks PE=1 SV=2	Marcks	0.93	1.17	164243.6339	18078.58221	285802.0215	44792.98901	250955.3374	212478.3633	111360.1434	1015762.445	435132.253	806603.2054	67503.35686	252299.4356	DTT
P26645	Myristoylated alanine-rich C-kinase substrate OS=Mus musculus OX=10090 GN=Marcks PE=1 SV=2	Marcks	6.64	6.64					82224.27025	87714.47443	66638.83386	68416.36929	38074.3779	60121.5823	48173.33655	53071.72135	DD
P28667	MARCKS-related protein OS=Mus musculus OX=10090 GN=Marcksl1 PE=1 SV=2	Marcksl1	6.64	6.64							39801.10781	62473.06251	192607.1929	395717.1399	288830.3085		DTT
P98064	Mannan-binding lectin serine protease 1 OS=Mus musculus OX=10090 GN=Masp1 PE=1 SV=2	Masp1	-0.36	-0.65	1886336.266	2024165.318	1298459.82	1882206.373	1331224.625	1380551.281	1078474.629	1455140.616	1503194.738	1515035.667	1218721.212	1080638.924	TCA
P98064	Mannan-binding lectin serine protease 1 OS=Mus musculus OX=10090 GN=Masp1 PE=1 SV=2	Masp1	0.08	-0.24	1079210.921	1434616.981	1302814.156	1454450.199	1201846.089	1492745.346	1572479.25	1091645.807	1237183.923	1186479.064	813990.5874	912435.6829	DD
Q91WP0	Mannan-binding lectin serine protease 2 OS=Mus musculus OX=10090 GN=Masp2 PE=1 SV=1	Masp2	-0.56	-1.52	109191.0391	125365.7638	54097.06115	62131.67273	89304.75569	70304.26318	40114.08434	41893.28481	23236.45542	23542.51484			DD
P04247	Myoglobin OS=Mus musculus OX=10090 GN=Mb PE=1 SV=3	Mb	-0.42	-0.54	314016.439	25988.03125	464227.8474	558742.5652	1027461.417	230657.979	280604.7266	292314.0617	137837.6735	316293.672	465220.692	275237.3736	TCA
P04247	Myoglobin OS=Mus musculus OX=10090 GN=Mb PE=1 SV=3	Mb	1.53	-1.93	132390.1278		67361.29502	208264.2883	735471.8271	825661.0859	621339.7293	151226.1733				37563.74883	DTT
P04247	Myoglobin OS=Mus musculus OX=10090 GN=Mb PE=1 SV=3	Mb	1.65	0.30	2274905.619	713436.3791	1643230.521	2951965.233	8613118.127	7489498.608	5423773.102	9209151.255	2009036.628	2802440.603	5786174.906	2254217.922	DD
P39039	Mannose-binding protein A OS=Mus musculus OX=10090 GN=Mbl1 PE=1 SV=1	Mbl1	-0.73	-1.47	4089876.283	4394567.488	4702310.756	4126965.02	1965376.406	3006959.066	2434707.961	2157051.474	1735134.201	1867280.054	1727012.56	1022962.915	TCA
P39039	Mannose-binding protein A OS=Mus musculus OX=10090 GN=Mbl1 PE=1 SV=1	Mbl1	-2.99	-0.36	250577.6349	156260.2087	248958.0115	234731.9799	12803.36464				261907.3533	333108.9175	277852.7961		DTT
P39039	Mannose-binding protein A OS=Mus musculus OX=10090 GN=Mbl1 PE=1 SV=1	Mbl1	-0.97	-1.33	1723044.344	2003053.908	1939731.86	2118201.017	1099525.873	1219844.184	1072683.951	741580.0123	743212.3181	651092.7245	837214.484	518241.4282	DD
P41317	Mannose-binding protein C OS=Mus musculus OX=10090 GN=Mbl2 PE=1 SV=2	Mbl2	-1.77	-2.11	4346753.333	4815787.407	4991254.052	4945704.295	835675.1016	1073888.276	299052.8601	1184413.566	1025621.378	1104868.858	1252370.326	732396.2157	TCA
P41317	Mannose-binding protein C OS=Mus musculus OX=10090 GN=Mbl2 PE=1 SV=2	Mbl2	-6.64	-6.64	263959.5264	206621.3348	273364.6769	160600.7903									DTT
P41317	Mannose-binding protein C OS=Mus musculus OX=10090 GN=Mbl2 PE=1 SV=2	Mbl2	-1.64	-2.12	3396889.313	3008603.39	3272666.469	3233603.325	1630503.377	1463889.211	1015903.636	1549633.672	553984.9969	1257868.627	765352.3271	376309.2823	DD

P14152	Malate dehydrogenase, cytoplasmic OS=Mus musculus OX=10090 GN=Mdh1 PE=1 SV=3	Mdh1	2.13	1.52	550364.936	27843.75752	339225.6284	542481.4533	2266256.281	1754654.46	2081086.059	1731919.333	1005284.236	1424606.509	1466127.646	1640865.69	TCA
P14152	Malate dehydrogenase, cytoplasmic OS=Mus musculus OX=10090 GN=Mdh1 PE=1 SV=3	Mdh1	2.52	1.50	121442.1094	35228.46005	46305.75227	249632.6656	1163816.751	1176971.619	922785.9236	1374457.566	236840.5292	591236.7092	610501.8505	732837.3343	DD
P34884	Macrophage migration inhibitory factor OS=Mus musculus OX=10090 GN=Mif PE=1 SV=2	Mif	1.64	2.00	96042.67196	23666.63061	62619.96933	38944.76143	120769.996	384127	73916.39173	183718.3756				197361.2434	DTT
B2RPV6	Multimerin-1 OS=Mus musculus OX=10090 GN=Mmrn1 PE=1 SV=2	Mmrn1	-1.76	-1.85	186320.1064	83859.77689	405068.8948	259334.8292	91553.47852	20892.42967	13604.94057	86597.05608	23197.22527	91720.50607	56410.69052	83625.29591	TCA
P26041	Moesin OS=Mus musculus OX=10090 GN=Msn PE=1 SV=3	Msn	2.19	1.99	6639.628644				138596.9336	159431.4358	166399.0995	91621.28518	139065.8975	108158.6711	55048.63778	136258.0986	TCA
P26041	Moesin OS=Mus musculus OX=10090 GN=Msn PE=1 SV=3	Msn	3.24	2.58	7080.12793				183497.4692	197201.31	169649.3616	245841.78	183582.7871	211266.393	77004.16052	129767.6627	DD
P26928	Hepatocyte growth factor-like protein OS=Mus musculus OX=10090 GN=Mst1 PE=2 SV=2	Mst1	0.47	0.73	90200.57848	143397.1875	103375.7404	168191.8391	204275.4297		173716.6947	263948.6494	178285.9655	245624.7931	276733.423	140193.3226	TCA
P26928	Hepatocyte growth factor-like protein OS=Mus musculus OX=10090 GN=Mst1 PE=2 SV=2	Mst1	6.64	6.64							139493.5934	150643.5468	66223.86997				DD
P02798	Metallothionein-2 OS=Mus musculus OX=10090 GN=Mt2 PE=1 SV=1	Mt2	6.64	6.64							40253.26518	107963.1488	85875.77118	85976.92736			DTT
P62774	Myotrophin OS=Mus musculus OX=10090 GN=Mtpn PE=1 SV=2	Mtpn	-6.64	0.75	56536.48027	15992.7505	80299.44555	15177.51572							15632.30611	219143.2989	DTT
P28665	Murinoglobulin-1 OS=Mus musculus OX=10090 GN=Mug1 PE=1 SV=3	Mug1	-1.01	-0.80	1177639084	1220004671	1176243079	1192057846	631126563.4	651402254.8	586994192.3	626862942.7	795806975.7	608489399.4	821311457.4	606836608.2	TCA
P28665	Murinoglobulin-1 OS=Mus musculus OX=10090 GN=Mug1 PE=1 SV=3	Mug1	-1.83	-1.63	124703482.1	200335073.8	125637171.1	159205858.1	39865115.71	59449875.73	51241631.45	29460482	40303911.14	56607599.96	112210894.8	11748415.15	DTT
P28665	Murinoglobulin-1 OS=Mus musculus OX=10090 GN=Mug1 PE=1 SV=3	Mug1	-0.98	-0.75	982944053	961776456.5	882318201.5	823450094.8	426215514.7	433493092.2	467582693.3	372640982	628974843.8	516614835.3	620000836.6	522097057.3	DD
P28666	Murinoglobulin-2 OS=Mus musculus OX=10090 GN=Mug2 PE=1 SV=2	Mug2	2.04	0.39	25016.37305	93311.92923	32597.98091	80515.03845	2734808.819	131403.5481	336707.4786	72558.89537	82102.1666	44569.00979	109500.1562	71473.55086	DD
B5X0G2	Major urinary protein 17 OS=Mus musculus OX=10090 GN=Mup17 PE=2 SV=2	Mup17	-2.79	-4.61	296796.9797	823993.4704	576935.5359	250586.4132	54618.26563			66124.95921	53333.63363	9948.911319	12760.54293	12923.34706	TCA
B5X0G2	Major urinary protein 17 OS=Mus musculus OX=10090 GN=Mup17 PE=2 SV=2	Mup17	-3.43	-5.38	9528259.625	5958032.335	5108957.445	5662480.859	281544.1002	290773.1999	304680.0173	370902.8873	224169.1998	16091.42673	41917.06194	365927.2345	DD
P11589	Major urinary protein 2 OS=Mus musculus OX=10090 GN=Mup2 PE=1 SV=1	Mup2	-2.95	-4.44	8931160.018	12496599.93	8838523.741	4614722.595	937104.1914	106696.1104	167103.3427	1148614.64	539949.9011	106933.0081	296831.7342	268178.7885	TCA
P11589	Major urinary protein 2 OS=Mus musculus OX=10090 GN=Mup2 PE=1 SV=1	Mup2	-2.95	-5.21	39091180.98	48831844.96	30492485.2	37978027.38	2902353.031	5127552.916	4754475.322	5175111.003	1630845.343	309927.806	529322.4825	729782.5046	DD
P04939	Major urinary protein 3 OS=Mus musculus OX=10090 GN=Mup3 PE=1 SV=1	Mup3	-1.30	-3.76	701941.5011	139244.5562	3129676.841	17599.81166			17513.14351	16782.55582		40198.83895			DTT
P02762	Major urinary protein 6 OS=Mus musculus OX=10090 GN=Mup6 PE=1 SV=2	Mup6	-3.11	-4.04	566624.0784	612149.7165	963173.2259	342646.3593	64616.90148	13277.63477	7525.661054		12778.35828				DTT
P05977	Myosin light chain 1/3, skeletal muscle isoform OS=Mus musculus OX=10090 GN=Myl1 PE=1 SV=2	Myl1	6.64	6.64					37970.35022	295528.3359	181551.2933	299469.4381	56928.88147	23444.47493	26250.50842	24551.85388	DTT
Q60605	Myosin light polypeptide 6 OS=Mus musculus OX=10090 GN=Myl6 PE=1 SV=3	Myl6	2.20	2.09	339310.5234	75009.3237	113684.0866	42465.74662	649871.5844	267643.3984	165852.2755	590790.7488	294379.3163	398100.4436	286447.9951	1212110.702	DTT
Q9CR61	NADH dehydrogenase [ubiquinone] 1 beta subcomplex subunit 7 OS=Mus musculus OX=10090 GN=Ndufb7 PE=1 SV	Ndufb7	-1.28	-0.25	1050163.095	1589658.388	2071319.949	1055755.758	3090112.5	56820.13553	139319.9114	2637898.888	883237.1531	1183063.623	1527178.834	1095339.837	TCA
Q01768	Nucleoside diphosphate kinase B OS=Mus musculus OX=10090 GN=Nme2 PE=1 SV=1	Nme2	3.23	2.76	133599.1615		65654.29422	92932.18085	1151047.75	895765.9597	846536.5488	673977.5512	787784.7554	898435.4413	490824.6935	1164260.599	TCA
Q01768	Nucleoside diphosphate kinase B OS=Mus musculus OX=10090 GN=Nme2 PE=1 SV=1	Nme2	2.73	2.44	103000.5313		58665.72514		832468.0397	1100629.792	1055636.793	776237.2086	585957.9828	617780.5347	495484.9992	905861.688	DD
Q02819	Nucleobindin-1 OS=Mus musculus OX=10090 GN=Nucb1 PE=1 SV=2	Nucb1	0.54	0.88	520762.4517	283521.4619	652791.7777	1676507.85	911867.2723	737185.1201	989066.61	2012250.592	2226318.221	1111464.588	2229283.483	1404504.384	DTT
Q64288	Olfactory marker protein OS=Mus musculus OX=10090 GN=Omp PE=1 SV=3	Omp	-3.51	-1.71	1113254.191	1618057.773	1297947.397	1467186.473	419609.6563	33123.01968	32953.82245	460454.4771	390019.7558	432323.6721	433027.8607	457095.4565	TCA
Q60590	Alpha-1-acid glycoprotein 1 OS=Mus musculus OX=10090 GN=Orm1 PE=1 SV=1	Orm1	2.57	2.47	18649151.06	22392178.77	23460207.75	24728761.13	160769414.8	107134754.5	148107046.8	173348891.5	146089720.4	89083862.17	132127136.4	95039514	TCA
Q60590	Alpha-1-acid glycoprotein 1 OS=Mus musculus OX=10090 GN=Orm1 PE=1 SV=1	Orm1	2.43	1.57	2466865.878	2128367.965	9535451.426	2468898.934	27496481.97	21876441.08	7530530.288	7617907.094	8597336.076	9168797.245	14720884.96	17543031.37	DTT
Q60590	Alpha-1-acid glycoprotein 1 OS=Mus musculus OX=10090 GN=Orm1 PE=1 SV=1	Orm1	2.60	2.38	25783710.93	21632350.95	18406632.08	22188984.55	115813228.5	129577156.4	100827239.6	141474284.6	122464255.1	101224327.1	128407926.3	129733576	DD
P07361	Alpha-1-acid glycoprotein 2 OS=Mus musculus OX=10090 GN=Orm2 PE=1 SV=1	Orm2	6.64	6.64	59799.50969	105155.0979	30254.86922	56823.8283	17814885.75	20871968.86	27944245.57	21018498.9	14306880.12	12563443.72	15796496.39	10907382.08	TCA
P07361	Alpha-1-acid glycoprotein 2 OS=Mus musculus OX=10090 GN=Orm2 PE=1 SV=1	Orm2	3.56	2.73	2692460.292	6033416.257	12416857.15	3995045.191	79903007.6	96806547.28	60775037.87	40788970.67	34549314.28	46426419.24	36917877.2	52661408.01	DTT
P07361	Alpha-1-acid glycoprotein 2 OS=Mus musculus OX=10090 GN=Orm2 PE=1 SV=1	Orm2	6.64	6.64	60549.91406	83216.72748	38766.44844		8911610.2	13787649.14	12388114.45	19945088.71	8074871.958	4682306.633	6968504.937	9011965.289	DD
P70296	Phosphatidylethanolamine-binding protein 1 OS=Mus musculus OX=10090 GN=Pebp1 PE=1 SV=3	Pebp1	1.01	1.15	665632.9163	178769.1626	1212643.162	390556.3391	934925.7227	487829.3125	607027.2952	1291656.068	721054.0609	930800.8114	1121538.171	2077499.442	DTT
Q11136	Xaa-Pro dipeptidase OS=Mus musculus OX=10090 GN=Pepd PE=1 SV=3	Pepd	1.28	-1.44	64959.44605	3217.872651	59485.97894	11793.46585	46350.44891	174191.0625		89504.98593		11057.93799	8578.963623		DTT
Q9Z126	Platelet factor 4 OS=Mus musculus OX=10090 GN=Pf4 PE=1 SV=1	Pf4	-3.76	-1.21	9558736.745	174287.9209	6869362.801	2753741.304	848283.8906	126114.5123	148384.6757	799216.3993	542080.3579	2739955.955	2407520.892	2608080.091	TCA
Q9Z126	Platelet factor 4 OS=Mus musculus OX=10090 GN=Pf4 PE=1 SV=1	Pf4	-6.64	-1.09	347722.9595	9379.56162	354888.6446	248763.0309						78073.52846	108010.6499		DTT
Q9Z126	Platelet factor 4 OS=Mus musculus OX=10090 GN=Pf4 PE=1 SV=1	Pf4	-3.63	-1.56	8001302	162195.4263	4852993.585	3289473.974	427608.7697	135532.02	607058.4155	198644.4565	331761.0662	1981100.135	1298822.497	1505407.434	DD
P09411	Phosphoglycerate kinase 1 OS=Mus musculus OX=10090 GN=Pgk1 PE=1 SV=4	Pgk1	2.23	1.22	45098.12088			49095.73781	354810.6328	598328.3863	543094.9122	175739.3718	46375.27835	28456.08852	387907.637	167736.4782	TCA
Q9CQ60	6-phosphogluconolactonase OS=Mus musculus OX=10090 GN=Pgls PE=1 SV=1	Pgls	0.82	2.47	44340.29466	10015.55493	48229.17687		83947.00356	47263.17969		127036.5474			17695.17438	318448.3347	DTT
Q8VCS0	N-acetylmuramoyl-L-alanine amidase OS=Mus musculus OX=10090 GN=Pglyrp2 PE=1 SV=1	Pglyrp2	0.60	0.78	5455345.852	5122678.571	7355745.415	6891331.715	7543597.721	8564382.64	9030616.632	7140233.602	9775393.638	9782821.409	7994800.51	7784739.667	TCA
Q8VCS0	N-acetylmuramoyl-L-alanine amidase OS=Mus musculus OX=10090 GN=Pglyrp2 PE=1 SV=1	Pglyrp2	0.84	-0.88	4749156.744	5129131.12	2730510.969	11207560.65	1829248.384	1305268.495	18042962.04	6519834.37	962867.1827	609364.7946	1302447.353	251006.3937	DTT
Q8VCS0	N-acetylmuramoyl-L-alanine amidase OS=Mus musculus OX=10090 GN=Pglyrp2 PE=1 SV=1	Pglyrp2	0.45	0.75	13436899.34	14250033.57	11694547.55	12970711.64	7329995.972	9411463.273	6632952.322	9341724.141	9697402.85	9999013.692	11002389.25	12554082.69	DD
Q9D0F9	Phosphoglucomutase-1 OS=Mus musculus OX=10090 GN=Pgm1 PE=1 SV=4	Pgm1	1.52	0.51	31199.83579		49299.54496	61162.94548	118633.9219	182175.3726	169973.6743	81668.61555	43980.93801	43718.96516	147704.7826	88127.35803	TCA
O70570	Polymeric immunoglobulin receptor OS=Mus musculus OX=10090 GN=Pigr PE=1 SV=1	Pigr	-0.19	-0.38	98244.0855	22880.31741	231679.8753	39562.14814			26996.59826	110860.5698	113194.9596	88826.67133	15006.99573		DTT
P52480	Pyruvate kinase PKM OS=Mus musculus OX=10090 GN=Pkm PE=1 SV=4	Pkm	2.09	1.83	262380.7941	162214.0255	301028.6849	657211.16	2810105.773	3923585.741	3261259.761	2107377.959	1893322.406	2090576.281	1515589.247	1972032.785	TCA
P52480	Pyruvate kinase PKM OS=Mus musculus OX=10090 GN=Pkm PE=1 SV=4	Pkm	1.59	0.27				29177.6311			87664.70208				39551.33319	31346.43773	DTT
P52480	Pyruvate kinase PKM OS=Mus musculus OX=10090 GN=Pkm PE=1 SV=4	Pkm	2.34	1.37	158653.4453	138055.3401	178236.7861	223558.252	724057.1296	993050.3752	1133647.739	948747.6502	498361.8105	481098.1271	479343.3842	608377.1745	DD
Q60963	Platelet-activating factor acetylhydrolase OS=Mus musculus OX=10090 GN=Pla2g7 PE=2 SV=2	Pla2g7	2.67	3.17	793057.7789	1002284.372	858376.4697	815293.2532	7267360.294	3864772.613	4067620.653	6439063.307	10484264.36	9147776.391	7353146.736	9566379.169	TCA
Q60963	Platelet-activating factor acetylhydrolase OS=Mus musculus OX=10090 GN=Pla2g7 PE=2 SV=2	Pla2g7	3.12	2.41	66575.65799	7417.968394	42523.93179	7009.732039	603458.6385	386850.25	1544888.07	384567.8374	947508.0063	315116.4528	762899.9789	172637.1006	DTT
Q60963	Platelet-activating factor acetylhydrolase OS=Mus musculus OX=10090 GN=Pla2g7 PE=2 SV=2	Pla2g7	2.34	2.56	604596.6748	688848.4428	729188.8895	689817.7305	3868012.726	3633210.984	3847739.527	3828195.835	6097225.322	5870459.626	4191810.018	5950987.946	DD
P20918	Plasminogen OS=Mus musculus OX=10090 GN=Plg PE=1 SV=3	Plg	-0.58	-0.60	191677833.2	211148816.6	204359507.2	236018428.2	129288411.8	175033838.3	185934530.8	130914150.7	117001147.1	139374443.7	143320160.2	124636032.7	TCA
P20918	Plasminogen OS=Mus musculus OX=10090 GN=Plg PE=1 SV=3	Plg	-1.43	-1.27	18967582.25	30674417.31	19431753.68	26945883.72	4878187.555	8347150.949	11366925.2	6818832.875	5634194.637	12267257.04	18869574.6	1475810.854	DTT
P20918	Plasminogen OS=Mus musculus OX=10090 GN=Plg PE=1 SV=3	Plg	-0.34	-0.49	191321361.7	220006852	189869881	200009826.5	159285817	152698242.7	151267012.3	156694342.3	128185158.4	153275454.2	149191065.3	134908534.4	DD
P55065	Phospholipid transfer protein OS=Mus musculus OX=10090 GN=Pltp PE=1 SV=1	Pltp	-0.11	-0.27	2084204.768	2326974.758	3235442.876	3013950.227	2081132.516	971243.1423	831966.1197	1802916.56	1892747.759	2060377.367	2545684.722	1624360.83	TCA
P55065	Phospholipid transfer protein OS=Mus musculus OX=10090 GN=Pltp PE=1 SV=1	Pltp	0.14	0.28	612574.0938	799702.3896	708926.1146	832376.8369	783303.4253	1038905.668	814130.2777	515030.0315	907376.6566	890306.1882	917721.3552	679290.3311	DD
P52430	Serum paraoxonase/arylesterase 1 OS=Mus musculus OX=10090 GN=Pon1 PE=1 SV=2	Pon1	-1.79	-0.93	26806856.94	27058717.66	28519406.43	29333662.27	10011306.98	6357837.522	6040865.568	7981785.558	11742259.73	16020868.02	22914137.51	8125097.376	TCA
P52430	Serum paraoxonase/arylesterase 1 OS=Mus musculus OX=10090 GN=Pon1 PE=1 SV=2	Pon1	-1.94	-2.10	5439907.777	13669687.04	2892148.564	18775214.11	3066442.52	1428097.462	3557863.106	2122804.468	2409990.642	1543514.55	4631911.839	622366.1593	DTT
P52430	Serum paraoxonase/arylesterase 1 OS=Mus musculus OX=10090 GN=Pon1 PE=1 SV=2	Pon1	-1.20	-0.88	12479986.63	14002017.49	12247748.92	14029975.92	5472107.55	6358020.109	5309061.97	4373096.234	5660594.581	7444924.184	10754514.93	5179045.602	DD
P17742	Peptidyl-prolyl cis-trans isomerase A OS=Mus musculus OX=10090 GN=Ppia PE=1 SV=2	Ppia	0.28	0.09			139649.1083		139510.1735	221027.4227	171287.2555	166770.3611	111274.2018	147123.056	149914.9407	176166.1276	DD
Q80XD8	Proline-rich acidic protein 1 OS=Mus musculus OX=10090 GN=Prap1 PE=2 SV=1	Prap1	-6.64	-6.64		4854.744283	27691.89292										DTT
P35700	Peroxiredoxin-1 OS=Mus musculus OX=10090 GN=Prdx1 PE=1 SV=1	Prdx1	1.43	0.85	60946.65423				228232.5938		55774.98395	164111.738	40112.09765	78217.67522	154070.7626	201974.6302	TCA
Q61171	Peroxiredoxin-2 OS=Mus musculus OX=10090 GN=Prdx2 PE=1 SV=3	Prdx2	1.87	1.53	2471048.362	223589.9952	853004.9283	220626.055	3064236.219	1380639.283	1839967.553	2901411.368	1550721.771	1660546.142	2472266.293	4861483.406	TCA
Q61171	Peroxiredoxin-2 OS=Mus musculus OX=10090 GN=Prdx2 PE=1 SV=3	Prdx2	1.22	-1.83	153879.7748	37556.99812	63709.8328	48205.61407	32367.91893	282924.4688	1160110.234	408295.5928	10472.78605	55536.0367	13730.96136		DTT
Q61171	Peroxiredoxin-2 OS=Mus musculus OX=10090 GN=Prdx2 PE=1 SV=3	Prdx2	1.49	1.39	1625839.75	344126.8064	379356.2914	178888.0975	1548600.421	1995810.551	2916172.417	1512117.684	534778.9711	1535421.924	1551624.013	3338460.625	DD
Q9JM99	Proteoglycan 4 OS=Mus musculus OX=10090 GN=Prg4 PE=1 SV=2	Prg4	2.50	3.46			29616.6995		434282.4063	280773.7552	223821.9544	727414.2905	406512.1404	476952.8992	282804.3757	426596.1404	TCA
Q9JM99	Proteoglycan 4 OS=Mus musculus OX=10090 GN=Prg4 PE=1 SV=2	Prg4	2.60	2.02	47240.46094	40433.42389		51055.63733	348616.8838	491933.8753	464506.8802	314439.2146	252846.7761	257309.4763	176117.5126	263018.9984	DD
P33587	Vitamin K-dependent protein C OS=Mus musculus OX=10090 GN=Proc PE=1 SV=2	Proc	1.44	-1.50		55049.78526		40511.25066	109005.5957		110266.6356	184499.1967		36630.44736	7606.202312		DD
Q64695	Endothelial protein C receptor OS=Mus musculus OX=10090 GN=Procr PE=1 SV=3	Procr	0.97	0.39	120324.6842	167983.9949	127937.9989	186003.3935	344361.0938	182822.7415	254037.8595	359501.6933	218253.8083		244161.6557	149888.3249	TCA
Q64695	Endothelial protein C receptor OS=Mus musculus OX=10090 GN=Procr PE=1 SV=3	Procr	0.63	0.03	567765.526	242619.7763	178147.8962	218309.3458	478999.995	46343.21875	270954.3538	379740.9566	185131.7489	242617.8622	237632.0386	430243.1011	DTT
Q9CQW3	Vitamin K-dependent protein Z OS=Mus musculus OX=10090 GN=Proz PE=1 SV=1	Proz	-2.66	-3.90	2866647.28	5066706.821	4567244.097	4317889.753	632837.1104	355720.9708	173539.9666	779713.8645	432756.263	138720.8886	263539.0849	209506.1693	TCA
Q9CQW3	Vitamin K-dependent protein Z OS=Mus musculus OX=10090 GN=Proz PE=1 SV=1	Proz	-6.64	2.58	118986.9032	95326.80755	141319.1585	90832.66746					109194.4102	259030.3608	384110.5275		DTT
Q9CQW3	Vitamin K-dependent protein Z OS=Mus musculus OX=10090 GN=Proz PE=1 SV=1	Proz	-2.62	-3.06	1187354.063	2071159.334	1640127.863	1416514.192	142729.4518	140818.1886	372843.9432	61282.91897	77435.8235	12180.43041	147744.5768	176171.2909	DD
Q61207	Prosaposin OS=Mus musculus OX=10090 GN=Psap PE=1 SV=2	Psap	-0.23	-6.64	30866.51662	99634.73238	69619.87999	137389.3478	51965.92383	363903.3578	128832.6311	90584.52091					TCA
Q61207	Prosaposin OS=Mus musculus OX=10090 GN=Psap PE=1 SV=2	Psap	0.83	1.44	14762455.23	9001741.943	17853845.21	6795070.249	3681872.921	2655768.285	1918775.157	6343678.255	3732600.83	7155938.705	4321482.755	8511315.986	DTT
Q61207	Prosaposin OS=Mus musculus OX=10090 GN=Psap PE=1 SV=2	Psap	0.61	0.34	309454.7578	295045.9211	283969.9731	292205.8454	465883.8101	590671.7222	252054.3736	426016.8251	398465.8871	524562.1748	420168.5052	358756.0737	DD
Q9R1P4	Proteasome subunit alpha type-1 OS=Mus musculus OX=10090 GN=Psma1 PE=1 SV=1	Psma1	1.09	1.95	29596.3655	94086.29946	52745.6697	151224.0146	132376.7188	334706.6304	251116.0077	205539.8838	534504.0397	587525.5491	240636.6618	364408.1238	TCA
Q9R1P4	Proteasome subunit alpha type-1 OS=Mus musculus OX=10090 GN=Psma1 PE=1 SV=1	Psma1	#VALUE!	6.64									29622.87737	85749.58787	69653.24266		DTT
Q9R1P0	Proteasome subunit alpha type-4 OS=Mus musculus OX=10090 GN=Psma4 PE=1 SV=1	Psma4	2.45	3.13		22999.39977	15437.23402		125772.9375		41246.12441	125492.432	215722.3425	187078.365		123231.3993	TCA
Q9Z2U1	Proteasome subunit alpha type-5 OS=Mus musculus OX=10090 GN=Psma5 PE=1 SV=1	Psma5	2.30	2.52	98936.59563	99538.85013	111261.0851	88940.97602	324879.4453	555938.9325	436155.6036	355459.5714	536551.1566	571423.8413	407956.8387	618548.6618	TCA
Q9Z2U1	Proteasome subunit alpha type-5 OS=Mus musculus OX=10090 GN=Psma5 PE=1 SV=1	Psma5	2.00	3.07	25157.99125	25122.04752		50254.52348	125953.0412		129803.4385	426703.9456	1475142.435	2676704.383	2563174.18	733309.1463	DTT
Q9QUM9	Proteasome subunit alpha type-6 OS=Mus musculus OX=10090 GN=Psma6 PE=1 SV=1	Psma6	2.35	2.84	205365.8334	183739.8687	207551.8456	143977.6961	874265.9219	947109.5899	710690.165	972847.8829	1443717.341	1518392.295	1031096.312	1123058.313	TCA
Q9QUM9	Proteasome subunit alpha type-6 OS=Mus musculus OX=10090 GN=Psma6 PE=1 SV=1	Psma6	2.15	2.17	69415.15625	96894.59151	70898.99935	66764.42079	387746.321	400985.6366	310426.6533	258522.6273	316621.6417	403261.5606	297252.2031	319931.2136	DD
Q9Z2U0	Proteasome subunit alpha type-7 OS=Mus musculus OX=10090 GN=Psma7 PE=1 SV=1	Psma7	0.84	1.68	83579.17531	83899.59605	286055.5968	308000.3146	517493.9531	182225.4366	6526.933969	329344.9756	749108.1213	807981.3507	354212.556	620199.5654	TCA
Q9Z2U0	Proteasome subunit alpha type-7 OS=Mus musculus OX=10090 GN=Psma7 PE=1 SV=1	Psma7	1.84	2.32			44042.77237		223268.1059	279114.0076	181365.7691	160570.0915	329553.0008	385103.4836	230159.7733	401415.3248	DD
O09061	Proteasome subunit beta type-1 OS=Mus musculus OX=10090 GN=Psmb1 PE=1 SV=1	Psmb1	2.27	2.55	103262.9967		68191.2041		339685	198125.5091	146509.8424	358441.22	375926.8595	824877.5473	87612.58295	522925.1925	TCA
O35955	Proteasome subunit beta type-10 OS=Mus musculus OX=10090 GN=Psmb10 PE=1 SV=1	Psmb10	2.61	3.48		25606.89813			166035.0256	221803.1578	146795.6149	133664.3137	287515.0614	283429.9459	264747.7764	369865.2791	DD
P99026	Proteasome subunit beta type-4 OS=Mus musculus OX=10090 GN=Psmb4 PE=1 SV=1	Psmb4	1.56	2.68	39583.50451		44996.21244		234922.6563	91247.77111	82478.24089	169648.1592	355708.6536	287308.7086	207951.1399	253317.6841	TCA
O55234	Proteasome subunit beta type-5 OS=Mus musculus OX=10090 GN=Psmb5 PE=1 SV=3	Psmb5	1.50	2.37	38489.74124	31209.79571	25351.67422		87371.20313			89130.89907	169635.4164	167992.222	144127.3544	155807.9393	TCA
Q60692	Proteasome subunit beta type-6 OS=Mus musculus OX=10090 GN=Psmb6 PE=1 SV=3	Psmb6	1.36	1.88	73992.50799	77054.56899	56140.75964	55681.05923	171929.6719	247639.2575	145964.3351	142014.5826	244375.9674	213519.9325	220226.7757	262435.9634	TCA
P28076	Proteasome subunit beta type-9 OS=Mus musculus OX=10090 GN=Psmb9 PE=1 SV=1	Psmb9	1.34	0.85	7433.475586		8820.278575		6815.728682	8173.111178	51158.5909	77016.87049	71416.15946	16450.67151	10117.12519	12942.00771	DD
Q61838	Pregnancy zone protein OS=Mus musculus OX=10090 GN=Pzp PE=1 SV=3	Pzp	-0.50	-0.46	2684978067	3171617132	3246471729	3037339152	2477938301	2108136474	1643614149	2373276282	2649675188	2462654254	2370882020	2218122925	TCA
Q61838	Pregnancy zone protein OS=Mus musculus OX=10090 GN=Pzp PE=1 SV=3	Pzp	-1.36	-1.39	307167807.7	529793015	420002539.4	501702579.5	218152366.2	256811161.1	210996401.6	140057403.9	152842785.7	213205908.9	317354171.1	89884862.87	DTT
Q61838	Pregnancy zone protein OS=Mus musculus OX=10090 GN=Pzp PE=1 SV=3	Pzp	-0.39	-0.34	2034165711	2292462602	2280751586	2337234868	1945773846	1970173079	1950716802	1452069250	2012503461	1858209352	1679499552	1758059048	DD
Q8BND5	Sulfhydryl oxidase 1 OS=Mus musculus OX=10090 GN=Qsox1 PE=1 SV=1	Qsox1	1.29	1.06	15262562.78	15238077.48	15342991.61	17963129.98	24014849.56	18032460.54	21618629.72	21239499.75	23561927.24	23671551.84	21625084.58	16703762.34	TCA

Q8BND5	Sulfhydryl oxidase 1 OS=Mus musculus OX=10090 GN=Qsox1 PE=1 SV=1	Qsox1	0.20	0.41	719378.5439	704252.8981	754638.5041	745738.6971	2284366.64	569364.1094	2318792.974	1452087.285	1017759.4	904701.6509	578351.8997	1016247.481	DTT
Q8BND5	Sulfhydryl oxidase 1 OS=Mus musculus OX=10090 GN=Qsox1 PE=1 SV=1	Qsox1	1.16	0.88	16298039.72	14631098.07	5628660.909	14083380.08	16121612.7	16062224.52	16459588.2	19487177.63	13352879.04	15424691.24	13862136.49	13036741.33	DD
P54726	UV excision repair protein RAD23 homolog A OS=Mus musculus OX=10090 GN=Rad23a PE=1 SV=2	Rad23a	0.95	1.79	132877.1299		61288.11318		73244.80898	39292.61719		246411.5683		24909.19836	78000.52183	721889.4921	DTT
Q60972	Histone-binding protein RBBP4 OS=Mus musculus OX=10090 GN=Rbbp4 PE=1 SV=5	Rbbp4	-1.14	1.90	34969.65983	107577.7673	38041.56475	151198.1355				29120.24372				239585.1081	DTT
Q00724	Retinol-binding protein 4 OS=Mus musculus OX=10090 GN=Rbp4 PE=1 SV=2	Rbp4	-0.34	-0.85	3430428.732	3656012.837	4749806.716	5612386.176	7290421.078	2699861.371	4194838.941	2601366.262	3229401.704	2084269.272	2420071.258	2492170.873	TCA
Q00724	Retinol-binding protein 4 OS=Mus musculus OX=10090 GN=Rbp4 PE=1 SV=2	Rbp4	-0.95	-2.08	434366.9712	888714.9679	612863.0488	1228167.396		4386686.93	855398.5968	144461.8098	73929.53296	121345.7295	240445.7942	49752.27188	DTT
Q00724	Retinol-binding protein 4 OS=Mus musculus OX=10090 GN=Rbp4 PE=1 SV=2	Rbp4	-1.38	-1.45	13466281.38	8141648.122	9352704.821	8334312.373	4179059.529	2798393.734	3447007.7	4961908.095	3376631.436	3136204.037	3631199.397	3563688.612	DD
P05366	Serum amyloid A-1 protein OS=Mus musculus OX=10090 GN=Saa1 PE=1 SV=2	Saa1	6.64	5.55	1237298.431	1188705.634	754433.12	1306595.226	127234229.6	185831540.5	197473285.7	149916813.4	87650605.23	74852770.08	21184251.33	119246237.5	TCA
P05366	Serum amyloid A-1 protein OS=Mus musculus OX=10090 GN=Saa1 PE=1 SV=2	Saa1	6.64	6.34	5647864.245	3278079.334	2988754.742	1483163.113	802271657.6	796746270.7	793807443	1082041329	622048479.8	538950572.8	172693730.1	834274138	DTT
P05366	Serum amyloid A-1 protein OS=Mus musculus OX=10090 GN=Saa1 PE=1 SV=2	Saa1	6.64	6.64	663547.7104	389750.339	186373.1897	147627.4905	246640841.2	200300567.5	294272200.3	274873779.1	140047110.1	124347780.1	38601560.72	116137411.4	DD
P05367	Serum amyloid A-2 protein OS=Mus musculus OX=10090 GN=Saa2 PE=1 SV=1	Saa2	4.57	1.62	14070.35623	70259.40422	25401.76547	67242.5807	142962563.5	190844239.6	190534945.1	172499544.1	51908371.86	39632736.34	16092797.29	73903990.19	TCA
P05367	Serum amyloid A-2 protein OS=Mus musculus OX=10090 GN=Saa2 PE=1 SV=1	Saa2	6.64	6.64	479713.8054	578699.3978	819078.8882	442814.0811	374127084.7	405974427.9	330695633	511245087.5	280501270.6	208646979.8	41023597.63	254260428.7	DTT
P05367	Serum amyloid A-2 protein OS=Mus musculus OX=10090 GN=Saa2 PE=1 SV=1	Saa2	3.96	1.88	77788.83691	89549.60451	93641.02032		65588429.88	66553402.84	83127140.01	72455499.52	38662629.42	28525856.03	8634833.556	59004799.18	DD
P04918	Serum amyloid A-3 protein OS=Mus musculus OX=10090 GN=Saa3 PE=1 SV=1	Saa3	6.64	6.64					691804.6875	397121.0942	368899.8169	820637.7416	230384.0593	278395.2875	224866.3496	344655.2254	TCA
P04918	Serum amyloid A-3 protein OS=Mus musculus OX=10090 GN=Saa3 PE=1 SV=1	Saa3	6.64	6.64							95078.51748			25953.08707			DTT
P04918	Serum amyloid A-3 protein OS=Mus musculus OX=10090 GN=Saa3 PE=1 SV=1	Saa3	4.64	3.32		27319.36184		18770.89649	946497.1051	1259470.595	1400708.959	1400606.891	367147.4572	526766.3995	91945.08774	803575.724	DD
P31532	Serum amyloid A-4 protein OS=Mus musculus OX=10090 GN=Saa4 PE=1 SV=2	Saa4	0.52	0.74	7936968.382	8835039.365	8140406.079	6098117.565	17079353.91	9124495.655	6396543.518	10437554.07	10569828.6	15662303.02	7709001.191	12105755.99	TCA
P31532	Serum amyloid A-4 protein OS=Mus musculus OX=10090 GN=Saa4 PE=1 SV=2	Saa4	-0.37	-0.88	2240093.004	3497330.319	2257192.035	3231956.727	1475018.896	3273015.574	3895201.692	1693114.368	975290.3484	1750039.736	2718154.723	7167989.767	DTT
P31532	Serum amyloid A-4 protein OS=Mus musculus OX=10090 GN=Saa4 PE=1 SV=2	Saa4	1.11	0.50	20837337.3	22689872.79	16066009.54	13832996.31	35488038.54	42012332.07	43223651.11	30656130.86	25663738.95	31163656.49	24030411.16	20286700.66	DD
Q8CIT9	Suprabasin OS=Mus musculus OX=10090 GN=Sbsn PE=2 SV=1	Sbsn	-6.64	-6.64	51289.87655		59066.85344	5717.82862									DTT
Q06318	Uteroglobin OS=Mus musculus OX=10090 GN=Scgb1a1 PE=1 SV=1	Scgb1a1	2.27	1.64	781971.2017	374190.5592	5562838.68	590829.9833	2665717.068	3375249.531	7359448.06	8375236.749	1117182.794	1305049.341	1670826.979	7269111.183	DTT
Q06318	Uteroglobin OS=Mus musculus OX=10090 GN=Scgb1a1 PE=1 SV=1	Scgb1a1	1.89	-0.52			120964.2753		311384.6901	383379.5796	526031.0405	1028908.408	84380.65838		84247.94234	160652.5297	DD
P70274	Selenoprotein P OS=Mus musculus OX=10090 GN=Selenop PE=1 SV=3	Selenop	-1.09	-1.11	4557362.767	7000839.268	7919361.744	7249945.113	5280348.914	1036622.437	2088361.97	4246228.397	3698383.277	3240890.523	3849713.008	2812677.548	TCA
P70274	Selenoprotein P OS=Mus musculus OX=10090 GN=Selenop PE=1 SV=3	Selenop	0.32	-0.55	2156962.644	2934135.042	1916323.929	4234917.956	3700704.227	1624514.232	2474848.037	2062636.224	2024482.505	1275757.715	1107981.756	1506287.798	DTT
P70274	Selenoprotein P OS=Mus musculus OX=10090 GN=Selenop PE=1 SV=3	Selenop	0.19	-0.57	2145245.015	1994326.782	2007767.546	2599180.262	1812349.785	2447388.395	2387556.769	2696066.542	1487152.527	1723268.181	1884163.984	1187951.849	DD
P18337	L-selectin OS=Mus musculus OX=10090 GN=Sell PE=1 SV=1	Sell	3.15	1.43		18275.12228		51356.65001	213721.9531	347288.8457	342120.1593	245169.3619	132292.5892	131547.4834		51827.30308	TCA
P18337	L-selectin OS=Mus musculus OX=10090 GN=Sell PE=1 SV=1	Sell	2.79	0.97	7819.941895			11778.05901	74646.16347	23547.56571	59332.82905	92861.25072	15364.36238	22889.55565			DD
Q8R121	Protein Z-dependent protease inhibitor OS=Mus musculus OX=10090 GN=Serpina10 PE=1 SV=1	Serpina10	0.50	-0.12	6646767.175	9305471.404	8786380.798	8850530.989	11321907.73	12607834.06	13105163.77	11401692.03	8558939.068	8323264.036	8529343.591	5531479.665	TCA
Q8R121	Protein Z-dependent protease inhibitor OS=Mus musculus OX=10090 GN=Serpina10 PE=1 SV=1	Serpina10	-0.22	-0.63	72737.9993	167683.2653	94961.33551	120583.0682	111340.6959	94882.13672	368696.7645	58898.35957	27451.90719	44119.07486	92475.74432	17781.66156	DTT
Q8R121	Protein Z-dependent protease inhibitor OS=Mus musculus OX=10090 GN=Serpina10 PE=1 SV=1	Serpina10	0.58	-0.24	3727074.449	4725861.96	2986681.128	3939444.051	4458829.587	6206945.738	5753568.602	6381826.918	3242025.06	3874008.905	3731508.509	3070813.406	DD
P07758	Alpha-1-antitrypsin 1-1 OS=Mus musculus OX=10090 GN=Serpina1a PE=1 SV=4	Serpina1a	-0.89	-1.49	337899594.1	406090799.6	390119924.6	322297175.6	254450331.5	207330857.2	174140538.5	249401051	156132829.7	128246603.9	161911600.2	146357064.8	TCA
P07758	Alpha-1-antitrypsin 1-1 OS=Mus musculus OX=10090 GN=Serpina1a PE=1 SV=4	Serpina1a	1.53	-0.33	20415.528	75700.34526	39617.57793	147040.2126	64493.93561	96249.09277	388694.5442	243438.0957	46939.15617	13302.07608	119589.6889	58633.10598	DTT
P07758	Alpha-1-antitrypsin 1-1 OS=Mus musculus OX=10090 GN=Serpina1a PE=1 SV=4	Serpina1a	-0.74	-1.45	90170495.61	95018884.5	74918264.11	77287694.96	52324701.92	52061293.46	56459039.62	73967246.21	27690220.72	34948674.31	30284870.16	23004710.72	DD
P22599	Alpha-1-antitrypsin 1-2 OS=Mus musculus OX=10090 GN=Serpina1b PE=1 SV=2	Serpina1b	-0.61	-0.91	1372946712	1433694236	1302439258	1082280983	1090061690	678593007.2	839592596.9	1206157422	962412818.7	918573209.4	876807520.5	890669786	TCA
P22599	Alpha-1-antitrypsin 1-2 OS=Mus musculus OX=10090 GN=Serpina1b PE=1 SV=2	Serpina1b	0.44	-0.53	151299441.3	170905129.8	124232197.6	217623274.7	220559925.4	308309075	193508110.3	148306073.3	137049065.3	97279186.55	133683403.5	133646366.6	DTT
P22599	Alpha-1-antitrypsin 1-2 OS=Mus musculus OX=10090 GN=Serpina1b PE=1 SV=2	Serpina1b	-0.33	-0.67	649557135.7	572351475.6	543127880	613512321.6	412715354.1	477726932.1	536538399.9	515638825	363827938	409395033.4	348854268.8	367594889.9	DD
Q00896	Alpha-1-antitrypsin 1-3 OS=Mus musculus OX=10090 GN=Serpina1c PE=1 SV=2	Serpina1c	0.46	-0.92	71973.46958	187611.9058	230059.9717	1020158.769	79595.25842	2630287.655	3689770.572	1016182.699	278909.3495	37619.63249	61065.60688	94413.40385	TCA
Q00896	Alpha-1-antitrypsin 1-3 OS=Mus musculus OX=10090 GN=Serpina1c PE=1 SV=2	Serpina1c	0.39	-1.09	26557518.95	25500142.75	20872970.64	34331226.61	31505936.22	65422016.98	41470382.79	20862654.97	14012295.23	8231205.94	15679879.12	8968771.367	DTT
Q00896	Alpha-1-antitrypsin 1-3 OS=Mus musculus OX=10090 GN=Serpina1c PE=1 SV=2	Serpina1c	-2.47	-2.56	1000186.039	616015.4385	814291.2185	772208.0481	112215.1015	110231.4684	163793.7167	210783.166	167600.8029	131954.3616	61043.91791	100026.4513	DD
Q00897	Alpha-1-antitrypsin 1-4 OS=Mus musculus OX=10090 GN=Serpina1d PE=1 SV=1	Serpina1d	0.18	0.32	50669294.27	38127933.28	37631658.37	19266938.42	54863355.11	29722654.77	33419381.52	64778290.77	48080761.28	58495438.55	51161480.14	55914899.03	TCA
Q00897	Alpha-1-antitrypsin 1-4 OS=Mus musculus OX=10090 GN=Serpina1d PE=1 SV=1	Serpina1d	0.74	2.12	7914893.926	3786393.684	4410305.845	3398286.858	7278878.154	12673762.24	5651991.051	14817568.87	17911117.81	12662530.84	12792424.16	21819207.87	DTT
Q00897	Alpha-1-antitrypsin 1-4 OS=Mus musculus OX=10090 GN=Serpina1d PE=1 SV=1	Serpina1d	1.41	0.83	10207073.09	9616661.117	8905330.241	9103212.671	16669162.01	21524945.84	28621688.47	17722853.12	13664256.72	13955375.75	13144566.87	14191346.83	DD
Q5I2A0	Serine protease inhibitor A3G OS=Mus musculus OX=10090 GN=Serpina3g PE=1 SV=2	Serpina3g	6.64	6.64					80573.32813		63450.75296	60293.91015		42515.447	31385.71588	48691.82824	TCA
Q5I2A0	Serine protease inhibitor A3G OS=Mus musculus OX=10090 GN=Serpina3g PE=1 SV=2	Serpina3g	1.81	1.64	5608.762207	4253.960353	4763.338957	6127.932588	4366.18232	18146.09683		33213.54403	17881.24449	15848.72398	11123.03846	18780.38694	DD
P07759	Serine protease inhibitor A3K OS=Mus musculus OX=10090 GN=Serpina3k PE=1 SV=2	Serpina3k	-0.22	-0.41	1340222829	1337395784	1515173204	1756090195	1046621434	1200202800	1254337997	1204108936	1304294157	1061659439	1087591228	1013970734	TCA
P07759	Serine protease inhibitor A3K OS=Mus musculus OX=10090 GN=Serpina3k PE=1 SV=2	Serpina3k	-0.24	-1.14	241051353.1	385311646.3	220237099.7	472508958.7	283409866.5	280796145.8	377420476.9	202103048	211427470.5	130733001.9	224025059.7	57699304.97	DTT
P07759	Serine protease inhibitor A3K OS=Mus musculus OX=10090 GN=Serpina3k PE=1 SV=2	Serpina3k	-0.50	-0.60	1404396271	1171534679	1374055316	1474402685	818963664.6	924391992.2	940038185.3	990203637.4	1029805304	876922546.2	911334022.1	1007647220	DD
Q03734	Serine protease inhibitor A3M OS=Mus musculus OX=10090 GN=Serpina3m PE=1 SV=2	Serpina3m	1.71	1.48	2056904.9	2207924.84	2125818.382	1750080.635	8268141.912	5047595.873	5807970.867	7336473.792	6438836.05	5561714.952	7006901.941	6106682.165	TCA
Q03734	Serine protease inhibitor A3M OS=Mus musculus OX=10090 GN=Serpina3m PE=1 SV=2	Serpina3m	1.23	1.25	6472.896172	20022.61861	13940.53985		45638.60994	312033.3438	16967.01169	33989.10088		50754.89969	109582.7621	29662.36535	DTT
Q03734	Serine protease inhibitor A3M OS=Mus musculus OX=10090 GN=Serpina3m PE=1 SV=2	Serpina3m	1.65	1.53	639522.4746	1058469.876	848560.7507	1227634.05	2902216.95	3883928.347	2714236.466	2739785.349	2845300.404	2700639.43	2435367.198	2868669.183	DD
Q91WP6	Serine protease inhibitor A3N OS=Mus musculus OX=10090 GN=Serpina3n PE=1 SV=1	Serpina3n	3.28	2.69	13565655.87	12521960.29	11356646.95	10380789.99	91998249.76	76903593.89	122351968.2	92932534.08	80000263.51	58460643.41	55197576.23	74907382.39	TCA
Q91WP6	Serine protease inhibitor A3N OS=Mus musculus OX=10090 GN=Serpina3n PE=1 SV=1	Serpina3n	2.52	1.34	1347971.935	1856148.606	835352.4413	2169605.277	8622292.002	6699061.451	19096844.5	6182415.053	4441801.674	4132529.143	5286684.909	1216825.817	DTT
Q91WP6	Serine protease inhibitor A3N OS=Mus musculus OX=10090 GN=Serpina3n PE=1 SV=1	Serpina3n	3.04	2.37	8071971.922	8225740.339	7510160.657	8420650.606	50276707.33	40910615.12	52455923.01	65758935.3	38716205.98	33164671.16	24294871.27	35050450.25	DD
Q06770	Corticosteroid-binding globulin OS=Mus musculus OX=10090 GN=Serpina6 PE=1 SV=1	Serpina6	-1.47	-2.36	19356600.13	25375512.42	26631452.73	20869697.1	10652869.46	854378.204	1552412.809	9386906.011	6490833.996	5664797.138	8919057.167	4119705.133	TCA
Q06770	Corticosteroid-binding globulin OS=Mus musculus OX=10090 GN=Serpina6 PE=1 SV=1	Serpina6	-1.48	-1.66	39061472.52	17297046.77	40071730.63	10084104.18	11164272.75	7596749.793	3307320.406	13242849.49	2906924.569	5443242.658	4781559.202	15478060.99	DTT
Q06770	Corticosteroid-binding globulin OS=Mus musculus OX=10090 GN=Serpina6 PE=1 SV=1	Serpina6	-0.81	-2.40	13407260.64	14784241.47	12562712.94	15500200.01	6311073.718	5911232.574	6521013.006	5718254.972	1859542.518	5785069.992	5596834.75	2477170.178	DD
P61939	Thyroxine-binding globulin OS=Mus musculus OX=10090 GN=Serpina7 PE=2 SV=1	Serpina7	-1.04	-1.12	143793.8679	184913.9131	441171.098	551996.9663	211825.4844	70266.24657	164640.0717	266636.6164	106425.0453	140070.1148	163113.8417	113570.1071	TCA
P61939	Thyroxine-binding globulin OS=Mus musculus OX=10090 GN=Serpina7 PE=2 SV=1	Serpina7	0.69	0.00	85316.88281	119878.7461		150728.1545	120125.0231			279098.7246	92825.81716	114663.3292	158077.8922	103768.925	DD
P32261	Antithrombin-III OS=Mus musculus OX=10090 GN=Serpinc1 PE=1 SV=1	Serpinc1	-0.82	-0.79	129536977.1	150135491.6	148977172.7	162138525.8	76524999.13	80908917.91	99222985.14	78265372.98	78325269.24	81643854.03	88559738.21	74995625.04	TCA
P32261	Antithrombin-III OS=Mus musculus OX=10090 GN=Serpinc1 PE=1 SV=1	Serpinc1	-0.84	-0.79	4747857.478	9087371.219	5930451.629	7497177.552	1984920.732	9545127.746	3872680.379	2199311.791	8528085.388	13035069.15	9831383.545	2241088.287	DTT
P32261	Antithrombin-III OS=Mus musculus OX=10090 GN=Serpinc1 PE=1 SV=1	Serpinc1	-0.40	-0.30	45458073.69	61680917.16	33095475.5	44771119.22	29620286.74	36063450.15	35108936.3	40019194.9	38925193.54	37400734.36	36718398.67	32158340.88	DD
P49182	Heparin cofactor 2 OS=Mus musculus OX=10090 GN=Serpind1 PE=1 SV=1	Serpind1	-0.30	0.01	16600452.54	16376569.32	17629219.25	14486204.78	16089821.91	7739671.342	8509668.29	14010730.62	13765913.98	19399202.8	15892670	12197148.67	TCA
P49182	Heparin cofactor 2 OS=Mus musculus OX=10090 GN=Serpind1 PE=1 SV=1	Serpind1	-1.04	-2.02	363912.3006	663842.0916	537289.3574	502748.2304	139653.4935	114300.5234	257291.9331	266454.3429	90653.93379	132202.775	99184.23052	49935.53378	DTT
P49182	Heparin cofactor 2 OS=Mus musculus OX=10090 GN=Serpind1 PE=1 SV=1	Serpind1	0.09	-0.10	9339895.277	8695961.43	6767588.917	8240998.463	7994409.524	9362091.719	9886126.399	8551637.834	6801019.972	9752125.516	7016754.384	7743740.629	DD
P97298	Pigment epithelium-derived factor OS=Mus musculus OX=10090 GN=Serpinf1 PE=1 SV=2	Serpinf1	-2.11	-1.82	52507.36778	66896.07807	69935.36378	46484.58465	11904.15715	28230.00586	10952.91358			13146.50537	19986.4213		DTT
P97298	Pigment epithelium-derived factor OS=Mus musculus OX=10090 GN=Serpinf1 PE=1 SV=2	Serpinf1	-0.31	-1.27	168499.0313	183563.6292	180697.5758	188896.5524	163123.1194	126920.5846	85712.56664	200999.9047	72562.88608	86700.55546	71924.80415		DD
Q61247	Alpha-2-antiplasmin OS=Mus musculus OX=10090 GN=Serpinf2 PE=1 SV=1	Serpinf2	-0.81	-0.68	64061022.5	75897483.35	57500523.42	54337258.76	43498173.59	32788995.08	42626104.92	36959758.28	42364124.66	47105102.92	40476983.87	32590011.47	TCA
Q61247	Alpha-2-antiplasmin OS=Mus musculus OX=10090 GN=Serpinf2 PE=1 SV=1	Serpinf2	-0.08	-1.25	5536117.528	11487464.88	5134692.515	10987589.85	9137741.802	6864866.557	10026588.3	4705639.367	4074352.087	4296380.845	5353704.925	6044384.844	DTT
Q61247	Alpha-2-antiplasmin OS=Mus musculus OX=10090 GN=Serpinf2 PE=1 SV=1	Serpinf2	-0.19	-0.61	36545532.22	41850666.96	29001936.69	25998321.33	28733103.93	28911726.39	21372924.11	28358554.38	24831597.93	27760705.81	25088885.29	23400230.95	DD
P97290	Plasma protease C1 inhibitor OS=Mus musculus OX=10090 GN=Serping1 PE=1 SV=3	Serping1	1.01	1.04	36029125.62	38659405.66	37482350.76	33192733.6	99616405.25	51296603.23	59715347.68	88935056.51	77480099.67	83982412.16	71577740.74	65148108.97	TCA
P97290	Plasma protease C1 inhibitor OS=Mus musculus OX=10090 GN=Serping1 PE=1 SV=3	Serping1	0.71	0.23	1714935.453	2221200.1	1884737.991	1871751.731	2881858.359	2120222.204	3543088.79	2445367.541	1410226.382	3083082.142	3868185.687	460068.2248	DTT
P97290	Plasma protease C1 inhibitor OS=Mus musculus OX=10090 GN=Serping1 PE=1 SV=3	Serping1	1.59	1.00	19865883.18	22444875.89	17314745.4	20716566.81	53965218.1	64330329.18	56309269.16	57385764.54	42342438.47	50214989.52	33207412.31	36173967.26	DD
Q9ES07	Solute carrier family 15 member 2 OS=Mus musculus OX=10090 GN=Slc15a2 PE=1 SV=1	Slc15a2	-0.44	-2.87		87411.98767	339692.7561	318507.3011	166982.2922	114857.5971	120392.7017	352285.3628	43692.7236				DD
P04919	Band 3 anion transport protein OS=Mus musculus OX=10090 GN=Slc4a1 PE=1 SV=1	Slc4a1	1.38	1.11	21826.59208		67435.27245		98978.05469			65948.59484			29799.10257	421763.0598	TCA
P04919	Band 3 anion transport protein OS=Mus musculus OX=10090 GN=Slc4a1 PE=1 SV=1	Slc4a1	-0.25	-0.54	55131.00391		10765.57336		41654.39321	56315.80633	69977.55083	34718.62278	4423.119392	6667.899494	7406.085105	508785.955	DD
P08228	Superoxide dismutase [Cu-Zn] OS=Mus musculus OX=10090 GN=Sod1 PE=1 SV=2	Sod1	-0.45	0.06	202574.0971	69181.23124	122112.8854	113401.2715	202073.125	254844.486	205110.7231	155787.8425	63980.91249	95630.13672	211659.0444	210923.665	TCA
P08228	Superoxide dismutase [Cu-Zn] OS=Mus musculus OX=10090 GN=Sod1 PE=1 SV=2	Sod1	0.99	1.16	4289173.088	717227.6554	2366865.688	1043123.771	4196596.646	2125809.77	922874.3965	4092338.553	22429167.55	14649197.71	14197890.04	11233125.84	DTT
P08228	Superoxide dismutase [Cu-Zn] OS=Mus musculus OX=10090 GN=Sod1 PE=1 SV=2	Sod1	1.64	-0.41	87121.10938		44938.6885	37410.4406	167386.6166	77954.85999	153203.5664	247818.8963	33757.66655	40055.87626	33722.00392		DD
O09164	Extracellular superoxide dismutase [Cu-Zn] OS=Mus musculus OX=10090 GN=Sod3 PE=1 SV=1	Sod3	-0.38	-1.19	539057.421	723190.0957	1326034.54	1133291.88	628229.8281	245251.2535	208391.0213	413657.0332	225098.5474	321363.8478	377682.1523	231695.9537	TCA
O09164	Extracellular superoxide dismutase [Cu-Zn] OS=Mus musculus OX=10090 GN=Sod3 PE=1 SV=1	Sod3	-0.94	-1.36	1171342.437	777623.7877	840270.6628	704356.7614	301625.1374	444804.5313	115037.6905	189178.3563		25756.95406		293533.1477	DTT
O09164	Extracellular superoxide dismutase [Cu-Zn] OS=Mus musculus OX=10090 GN=Sod3 PE=1 SV=1	Sod3	-1.25	-1.32		266276.7611		417515.5798	147787.0491	198130.4478	81997.33837	156023.5019	155279.8817	115055.7971	124239.1324		DD
P70663	SPARC-like protein 1 OS=Mus musculus OX=10090 GN=Sparcl1 PE=1 SV=3	Sparcl1	-0.41	-0.38	375091.0567	954728.0521	579675.9424	623374.1064	373791.7837	354930.0485	463511.7191	637236.2062	503727.986	514542.4172	411343.6361	481584.9484	TCA
P70663	SPARC-like protein 1 OS=Mus musculus OX=10090 GN=Sparcl1 PE=1 SV=3	Sparcl1	-0.19	0.13	171566.9603	11800.79882	342288.9058	272150.7452	92969.92828	10890.01074	294397.573	176532.0322	46001.21301	309416.9836	21478.20497		DTT
P70663	SPARC-like protein 1 OS=Mus musculus OX=10090 GN=Sparcl1 PE=1 SV=3	Sparcl1	1.42	1.45	230529.2991	302991.5115	278109.9773	360638.6203	564280.1975	937725.0369	517812.7376	1188899.16	944258.1231	1367545.282	664255.9573	475236.9757	DD
P09036	Serine protease inhibitor Kazal-type 1 OS=Mus musculus OX=10090 GN=Spink1 PE=1 SV=1	Spink1	1.40	0.27	158511.0398	15457.81035	100157.6434	89124.6848		20397.35547	249780.5358	1464792.115	92145.27918	141462.5412			DTT
P10923	Osteopontin OS=Mus musculus OX=10090 GN=Spp1 PE=1 SV=1	Spp1	0.09	-0.60	59885.77959		176378.6246	162278.3483	127001.2422	178052.4305	183398.7288	167813.9441	87494.67941	115532.1441	65410.10293	108041.6041	TCA
P10923	Osteopontin OS=Mus musculus OX=10090 GN=Spp1 PE=1 SV=1	Spp1	0.66	-0.46	220540.2647	48734.65125	231799.3978	141674.4833	316515.0335	67960.54688	153285.2597	396087.6489	67342.96422	67214.41118	26670.59126	358168.568	DTT
Q8K1I3	Secreted phosphoprotein 24 OS=Mus musculus OX=10090 GN=Spp2 PE=1 SV=2	Spp2	-0.70	-0.47	2141166.435	2193383.83	1767504.663	2615397.605	1255377.086	1298068.392	1208148.086	1414248.735	1584977.382	1792729.75	1238591.661	1390216.183	TCA
Q8K1I3	Secreted phosphoprotein 24 OS=Mus musculus OX=10090 GN=Spp2 PE=1 SV=2	Spp2	-2.05	0.82	99425.60542	60616.90593	80578.44565	69777.15885			18060.966		160721.5596	79156.74264	156394.8931		DTT
Q8K1I3	Secreted phosphoprotein 24 OS=Mus musculus OX=10090 GN=Spp2 PE=1 SV=2	Spp2	-0.48	-0.09	1104352.313	1134329.755	1106192.056	986252.1718	815007.684	771605.8461	917366.4942	570448.1113	1094713.32	1060276.648	773854.8901	1051606.374	DD
P13609	Serglycin OS=Mus musculus OX=10090 GN=Srgn PE=1 SV=1	Srgn	-1.44	-2.86	440009.9074			329489.4339	140288.867				13170.18802			210194.6496	DTT
Q921I1	Serotransferrin OS=Mus musculus OX=10090 GN=Tf PE=1 SV=1	Tf	0.18	-0.08	2745125691	2620965481	2515522518	2637736893	2591288408	3457041570	3353639025	2593396459	2611061249	2825768827	2661477821	2601907656	TCA
Q921I1	Serotransferrin OS=Mus musculus OX=10090 GN=Tf PE=1 SV=1	Tf	-0.58	-0.35	377931655.6	385924870.4	358914160.6	385322773.8	222923262.6	336764278.8	357729298.7	228969955.3	227773675.1	378135807.5	500435347.8	104771379.1	DTT
Q921I1	Serotransferrin OS=Mus musculus OX=10090 GN=Tf PE=1 SV=1	Tf	0.29	0.09	2221774722	2136314615	2100542392	2125230854	2306686306	2689651471	2447462174	2477463309	2259836225	2428950775	2309284243	2287871916	DD
Q62351	Transferrin receptor protein 1 OS=Mus musculus OX=10090 GN=Tfrc PE=1 SV=1	Tfrc	-2.18	-2.29		135864.6939		125914.3506		35255.76126		23661.81789	26782.97604				DD

P82198	Transforming growth factor-beta-induced protein ig-h3 OS=Mus musculus OX=10090 GN=Tgfbi PE=1 SV=1	Tgfbi	1.30	1.81	54285.51552	66244.23971	46579.34181	40203.55773	133767.7656	161899.0941	91199.10303	119735.5341	180513.2071	206261.2091	338952.2104	129469.9467	TCA
P35441	Thrombospondin-1 OS=Mus musculus OX=10090 GN=Thbs1 PE=1 SV=1	Thbs1	-2.74	-0.98	21264107.77	412715.7316	27040017.16	18017203.35	3146822.657	1189287.553	2203234.421	3718584.461	2459741.395	15026486.44	8100732.573	14329297.68	TCA
P35441	Thrombospondin-1 OS=Mus musculus OX=10090 GN=Thbs1 PE=1 SV=1	Thbs1	0.52	-0.98	1214648.8	151266.9342	1719530.769	919887.0035	8781.384152	202228.3906	193226.4529	1243976.295	83841.41086	1787711.37	1502703.455	77329.36576	DTT
P35441	Thrombospondin-1 OS=Mus musculus OX=10090 GN=Thbs1 PE=1 SV=1	Thbs1	-2.29	-0.60	11261025.06	1341647.783	15102752.36	11711262.76	2852577.509	2089719.854	3951156.162	2811019.04	2882586.706	9691302.406	5380580.494	10196889.64	DD
Q9Z1T2	Thrombospondin-4 OS=Mus musculus OX=10090 GN=Thbs4 PE=1 SV=1	Thbs4	-0.91	-1.54	153150.8382	149192.3852	234159.0245	413585.4329	103717.7188			98371.21758	76142.37252	76170.57365	62029.64082	55423.52757	TCA
Q9Z1T2	Thrombospondin-4 OS=Mus musculus OX=10090 GN=Thbs4 PE=1 SV=1	Thbs4	-0.37	-1.02	160462.1016	172820.7057	200822.6342	219563.4368	82662.91518	145892.0162	87682.506	108228.9511		62932.64338		58182.38592	DD
P39876	Metalloproteinase inhibitor 3 OS=Mus musculus OX=10090 GN=Timp3 PE=1 SV=1	Timp3	-3.05	-1.28	248104.1603		271414.7682	118190.2818	28094.66406			31499.99458		111798.254	98272.85455	64708.42578	TCA
P40142	Transketolase OS=Mus musculus OX=10090 GN=Tkt PE=1 SV=1	Tkt	1.68	0.18			14753.20547		355056.7031	634166.0564	573191.8728	311672.1722	72812.52349	205018.084	82692.29152	231643.8143	TCA
P40142	Transketolase OS=Mus musculus OX=10090 GN=Tkt PE=1 SV=1	Tkt	2.62	1.26	9757.011719		5750.20456		60491.64752	68596.83	80272.81209	73106.72774	24142.4675	22542.92361	13324.33722	16395.41729	DD
P26039	Talin-1 OS=Mus musculus OX=10090 GN=Tln1 PE=1 SV=2	Tln1	-0.74	-0.38	60827.70933	33235.57013		117679.2824	46625.62109			28414.06114	23599.95265	57026.74378	50548.43164	62136.79752	TCA
P26039	Talin-1 OS=Mus musculus OX=10090 GN=Tln1 PE=1 SV=2	Tln1	#VALUE!	6.64									50562.3831	44758.37869			DTT
P20065	Thymosin beta-4 OS=Mus musculus OX=10090 GN=Tmsb4x PE=1 SV=1	Tmsb4x	0.07	0.21	2824114.106	763681.2805	3387772.797	994810.1566	2383574.696	3256788.725	519948.2055	2791090.337	3588559.854	5108720.551	2705935.573	1092442.139	DTT
P17751	Triosephosphate isomerase OS=Mus musculus OX=10090 GN=Tpi1 PE=1 SV=4	Tpi1	2.36	0.95	51077.89667		35995.40471	134181.5173	941199.25	1740011.48	1139795.808	899827.7692	73424.4844	175693.7639	719480.9972	229855.4147	TCA
P17751	Triosephosphate isomerase OS=Mus musculus OX=10090 GN=Tpi1 PE=1 SV=4	Tpi1	0.53	1.18	36430.9765		36198.04648	50366.75953	26749.83552		93979.24965	52667.18667	112886.9482	114351.6134	45274.14361		DTT
P17751	Triosephosphate isomerase OS=Mus musculus OX=10090 GN=Tpi1 PE=1 SV=4	Tpi1	1.29	0.03	91518.63281			68768.42437	3410854.272	2818402.745	203982.6556	383340.3709	51887.77819	56053.12934	188949.3131	116770.1433	DD
P63028	Translationally-controlled tumor protein OS=Mus musculus OX=10090 GN=Tpt1 PE=1 SV=1	Tpt1	0.54	0.29	15328.29761		24578.74584	23057.55681	94741.98188		34590.97129		18756.19071		28118.63863	104054.9906	DTT
Q9CQN1	Heat shock protein 75 kDa, mitochondrial OS=Mus musculus OX=10090 GN=Trap1 PE=1 SV=1	Trap1	3.00	3.05	29961.56923				240306.7344	240637.3368	275850.8658	222536.7391	216648.6914	284239.9647	118913.1665	310054.7328	TCA
Q8K558	Trem-like transcript 1 protein OS=Mus musculus OX=10090 GN=Treml1 PE=1 SV=2	Treml1	-3.10	-1.86	1148208.63	54500.00553	2560051.121	738403.5933	40248.484	578296.1729	10494.92325	116630.9906	39435.94353		17813.32147	315655.0629	DTT
Q8CBR6	Tsukushin OS=Mus musculus OX=10090 GN=Tsku PE=2 SV=2	Tsku	0.10	-0.04	11123.95477	35087.1528	29022.31766	80682.74717	6336.306585		163221.8147		21620.60636	44725.54683	19288.443	44739.42727	DTT
P07309	Transthyretin OS=Mus musculus OX=10090 GN=Ttr PE=1 SV=1	Ttr	-0.46	-1.16	271130676	253554480.6	326787628.2	250823354.8	180554601.5	154412259.6	156430594.1	213435094.1	128494954.4	115370349.7	155984692.7	136638566.5	TCA
P07309	Transthyretin OS=Mus musculus OX=10090 GN=Ttr PE=1 SV=1	Ttr	-0.47	-0.74	647599901.5	660055612.9	336769574.3	709504285.5	414463788.7	450039913.4	325387144.8	474252272.1	389052879.2	355640747.7	199081448.3	394352424.5	DTT
P07309	Transthyretin OS=Mus musculus OX=10090 GN=Ttr PE=1 SV=1	Ttr	-1.23	-1.52	279406384.6	270236617.9	312376955.2	295258219.3	125096530.1	126522764.5	130860422.8	140210122.7	103359221.1	110501110.4	113104387.6	129399762.2	DD
Q7TMM9	Tubulin beta-2A chain OS=Mus musculus OX=10090 GN=Tubb2a PE=1 SV=1	Tubb2a	1.23	0.69	53776.73514	42335.05796	59962.85533	56003.34348	120065.25	159824.4206	128976.3261	112401.5244	80900.88983	88067.69003	143661.4857	85411.76027	TCA
P10639	Thioredoxin OS=Mus musculus OX=10090 GN=Txn PE=1 SV=3	Txn	2.24	3.78	301215.8318	22757.43729	184029.3722	23480.09325	175126.9296	139765.1719		688774.9885				900413.6394	DTT
P10639	Thioredoxin OS=Mus musculus OX=10090 GN=Txn PE=1 SV=3	Txn	1.45	0.45	87782.42188		59239.87389		158180.1243	244611.134	232292.177	194100.3302	58979.57509	76907.62024	125509.7906	138426.1368	DD
P62984	Ubiquitin-60S ribosomal protein L40 OS=Mus musculus OX=10090 GN=Uba52 PE=1 SV=2	Uba52	2.12	1.56	333231.7343	66472.52665	158623.7471	17423.20912	781002.5313			524688.0262	385614.81	378637.9815	565114.1407	972130.4587	TCA
Q6P5E4	UDP-glucose:glycoprotein glucosyltransferase 1 OS=Mus musculus OX=10090 GN=Uggt1 PE=1 SV=4	Uggt1	-1.36	0.43	127107.3089	70793.27329	371619.9696	158106.585	33242.63964	55281.06018	32235.42037	472491.4666	207746.1785	254467.683	217744.1184	52743.83939	DTT
P29533	Vascular cell adhesion protein 1 OS=Mus musculus OX=10090 GN=Vcam1 PE=1 SV=1	Vcam1	2.25	1.93	2564056.472	3681057.378	2619005.107	3511673.468	3162482.625	1505082.205	1587022.124	2175554.499	2055233.626	1899601.248	2861590.038	1662404.398	TCA
P29788	Vitronectin OS=Mus musculus OX=10090 GN=Vtn PE=1 SV=2	Vtn	0.25	-0.23	41126842.85	48249387.21	44655581.58	46209789.6	53531085.08	57581641	48853338.64	45370673.98	38683998.24	38833938.58	38156016.78	31402840.1	TCA
P29788	Vitronectin OS=Mus musculus OX=10090 GN=Vtn PE=1 SV=2	Vtn	-0.25	-0.71	23288512.02	27220896.41	16763571.1	22045869.52	19973680.46	10252772.97	12300672.51	21606986.99	10415203.2	11671920.35	8821299.665	10052509.05	DTT
P29788	Vitronectin OS=Mus musculus OX=10090 GN=Vtn PE=1 SV=2	Vtn	0.03	-0.08	45071349.46	68267324.8	67440710.12	39492166.22	54699199.35	35766038.97	32776527.72	46833274.05	41238763.78	39859031.19	58362012.66	39274358.55	DD
P62259	14-3-3 protein epsilon OS=Mus musculus OX=10090 GN=Ywhae PE=1 SV=1	Ywhae	6.64	6.64						5122.587265		8880.506601	11914.1612		16384.13616		DD
P68254	14-3-3 protein theta OS=Mus musculus OX=10090 GN=Ywhaq PE=1 SV=1	Ywhaq	6.64	6.64					43324.15077	92553.79094	57758.85227	69843.48329	42983.06957			75198.74855	DD
P63101	14-3-3 protein zeta/delta OS=Mus musculus OX=10090 GN=Ywhaz PE=1 SV=1	Ywhaz	1.38	1.73	58352.48471	31399.09612	50886.89237	98026.51	144660.7031	104343.335	189143.51	138723.8383	174348.8401	251294.4506	100133.7828	201002.6815	TCA
P63101	14-3-3 protein zeta/delta OS=Mus musculus OX=10090 GN=Ywhaz PE=1 SV=1	Ywhaz	6.64	6.64							145789.2442	43303.16782	40758.95122	65032.95548	53153.45949		DTT
P63101	14-3-3 protein zeta/delta OS=Mus musculus OX=10090 GN=Ywhaz PE=1 SV=1	Ywhaz	3.17	2.60	89707.27734	29184.61828	59757.36119	86360.60144	714148.2374	984992.7918	692169.0584	1027587.931	570418.4522	669153.1563	406365.3804	748779.8826	DD
Q62523	Zyxin OS=Mus musculus OX=10090 GN=Zyx PE=1 SV=2	Zyx	-1.43	-0.41	100493.8956		199810.744	122599.1739	25200.15111	5289.718872		53541.21214	112857.6725	98975.07044	13863.92122	157626.0786	DTT

Supplementary Table 5: serum proteins differentially regulated in the conditions control (CTRL), 7dpi and 9dpi.

Abundances (Normalized): F1: Sample, CTRL	Abundances (Normalized): F2: Sample, CTRL	Abundances (Normalized): F3: Sample, CTRL	Abundances (Normalized): F4: Sample, CTRL	Abundances (Normalized): F5: Sample, 7dpi	Abundances (Normalized): F6: Sample, 7dpi	Abundances (Normalized): F7: Sample, 7dpi	Abundances (Normalized): F8: Sample, 7dpi	Abundances (Normalized): F9: Sample, 9dpi	Abundances (Normalized): F10: Sample, 9dpi	Abundances (Normalized): F11: Sample, 9dpi	Abundances (Normalized): F12: Sample, 9dpi	-Log ANOVA p value	ANOVA q-value	Accession	Description	T: Gene name	Abundance Ratio (LOG2): (7dpi) / (CTRL)	Abundance Ratio (LOG2): (9dpi) / (CTRL)	Significant pairs	Method
25.8838	26.0366	25.7023	25.7235	26.8306	27.0097	27.0752	27.2337	26.9103	26.848	26.55	26.6088	5.07825	6.36E-05	A6X935	Inter alpha-trypsin inhibitor, heavy chain 4 OS=Mus musc	Itih4	1.34	0.93	9dpi_CTRL;7dpi_CTRL	DD
26.3738	26.6017	26.5479	26.4706	27.5586	27.7104	28.1126	27.6267	27.5748	27.5311	27.2851	27.2123	4.81935	0.000105653	A6X935	Inter alpha-trypsin inhibitor, heavy chain 4 OS=Mus musc	Itih4	1.39	0.93	9dpi_CTRL;7dpi_CTRL	TCA
23.1838	22.5064	22.2846	22.433	18.103	18.1495	18.2169	18.5007	17.7742	13.974	15.3553	18.4812	3.77473	0.000736378	B5X0G2	Major urinary protein 17 OS=Mus musculus OX=10090 GI	Mup17	-3.43	-5.38	CTRL_9dpi;CTRL_7dpi	DD
18.1791	19.6523	19.1381	17.9349	15.7371	NaN	NaN	16.0129	15.7028	13.2803	13.6394	13.6577	3.30072	0.00188513	B5X0G2	Major urinary protein 17 OS=Mus musculus OX=10090 GI	Mup17	-2.79	-4.61	CTRL_9dpi;CTRL_7dpi	TCA
17.0686	NaN	17.4827	16.429	14.9014	NaN	13.3884	15.7127	16.5697	17.0928	16.6794	NaN	1.75137	0.0359208	D3Z6Q9	Bridging integrator 2 OS=Mus musculus OX=10090 GN=Bi	Bin2	-2.16	-0.39	9dpi_7dpi;CTRL_7dpi	DTT
27.2696	27.4828	27.1009	27.3506	27.6013	27.7244	27.6054	27.9682	27.9147	27.804	27.9595	27.7883	3.03254	0.00302192	O08677	Kininogen-1 OS=Mus musculus OX=10090 GN=Kng1 PE=1	Kng1	0.47	0.47	7dpi_CTRL;9dpi_CTRL	DD
16.3015	NaN	17.8992	17.2267	14.4683	NaN	NaN	13.9562	14.0584	14.6042	14.2923	15.7817	2.1982	0.0151235	O08742	Platelet glycoprotein V OS=Mus musculus OX=10090 GN=	Gp5	-3.01	-2.43	CTRL_7dpi;CTRL_9dpi	TCA
20.1597	19.5687	19.6805	19.4259	18.2024	18.7628	16.8117	17.5294	NaN	14.6527	NaN	18.1632	1.597	0.0495206	O09164	Extracellular superoxide dismutase [Cu-Zn] OS=Mus mus	Sod3	-0.94	-1.36	CTRL_9dpi	DTT
19.0401	19.464	20.3387	20.1121	19.2609	17.9039	17.6689	18.6581	17.7802	18.2938	18.5268	17.8219	2.15617	0.0165469	O09164	Extracellular superoxide dismutase [Cu-Zn] OS=Mus mus	Sod3	-0.38	-1.19	CTRL_9dpi;CTRL_7dpi	TCA
19.1201	18.9101	19.7604	19.5096	17.3739	17.649	18.0176	16.8755	18.0254	18.6002	17.6704	18.0416	3.33127	0.00177627	O35930	Platelet glycoprotein Ib alpha chain OS=Mus musculus O	Gp1ba	-1.39	-1.29	CTRL_7dpi;CTRL_9dpi	DD
19.468	19.4412	20.361	19.8869	18.569	17.2909	16.5514	18.1484	18.4939	18.8189	18.4788	18.8778	2.6903	0.00592549	O35930	Platelet glycoprotein Ib alpha chain OS=Mus musculus O	Gp1ba	-1.61	-1.4	CTRL_7dpi	TCA
NaN	14.6442	NaN	NaN	17.3411	17.7589	17.1635	17.0283	18.1333	18.1126	18.0143	18.4966	2.40546	0.0103793	O35955	Proteasome subunit beta type-10 OS=Mus musculus OX=	Psmb10	2.61	3.48	7dpi_CTRL	DD
15.2322	14.9297	14.6298	NaN	16.4149	NaN	NaN	16.4436	17.3721	17.358	17.137	17.2494	4.94765	8.36E-05	O55234	Proteasome subunit beta type-5 OS=Mus musculus OX=1	Psmb5	1.5	2.37	7dpi_CTRL;9dpi_CTRL;9dpi_7dpi	TCA
19.613	20.1013	20.0096	20.1163	18.0307	18.4203	17.8851	17.5836	18.163	17.8996	18.1977	17.6254	5.31039	4.21E-05	O70165	Ficolin-1 OS=Mus musculus OX=10090 GN=Fcn1 PE=1 SV=	Fcn1	-1.59	-1.63	CTRL_9dpi;CTRL_7dpi	TCA
24.5447	24.6461	24.3798	24.3074	24.0519	24.1507	24.095	23.6262	24.5047	24.4372	24.5127	24.2892	2.24796	0.0138902	O70362	Phosphatidylinositol-glycan-specific phospholipase D OS=	Gpld1	-0.43	-0.06	9dpi_7dpi;CTRL_7dpi	DD
21.2276	22.1995	21.5758	21.7616	19.7567	18.9873	19.3375	19.3303	19.0359	19.9062	20.9024	16.8955	1.90372	0.0273581	O70362	Phosphatidylinositol-glycan-specific phospholipase D OS=	Gpld1	-1.54	-1.38	CTRL_9dpi;CTRL_7dpi	DTT
25.3387	25.1941	25.15	25.0326	24.7242	24.9048	24.6382	24.6436	25.2708	25.3776	25.4065	25.0377	3.06867	0.0028472	O70362	Phosphatidylinositol-glycan-specific phospholipase D OS=	Gpld1	-0.48	0.08	CTRL_7dpi;9dpi_7dpi	TCA
13.6836	13.7528	NaN	13.8233	16.0544	NaN	17.0131	16.8665	16.7503	15.9551	NaN	16.5189	3.80985	0.000687838	O70370	Cathepsin S OS=Mus musculus OX=10090 GN=Ctss PE=1 S	Ctss	3.11	2.77	9dpi_CTRL;7dpi_CTRL	DTT
13.7463	14.6313	14.3409	14.7933	18.2168	17.7431	18.8516	17.9674	18.4045	18.1956	17.6793	17.5072	5.91314	1.49E-05	O70370	Cathepsin S OS=Mus musculus OX=10090 GN=Ctss PE=1 S	Ctss	3.18	2.82	9dpi_CTRL;7dpi_CTRL	TCA
16.2857	16.816	16.5729	16.2511	14.9951	15.8562	15.5827	15.0691	NaN	NaN	NaN	15.3412	2.3871	0.0107461	O88200	C-type lectin domain family 11 member A OS=Mus muscu	Clec11a	-1.2	-1.09	CTRL_7dpi	DD
23.2908	23.4495	23.3016	23.3104	21.9972	22.4039	22.3141	22.0958	22.1155	22.2917	22.1824	22.1233	6.33308	8.52E-06	O88947	Coagulation factor X OS=Mus musculus OX=10090 GN=F1	F10	-1.14	-1.01	CTRL_9dpi;CTRL_7dpi	DD
19.0562	18.7061	18.954	18.2837	17.1844	16.5648	17.0637	15.7905	14.4434	16.4124	17.3236	15.8405	2.59868	0.00708117	O88947	Coagulation factor X OS=Mus musculus OX=10090 GN=F1	F10	-1.57	-1.38	CTRL_9dpi;CTRL_7dpi	DTT
24.0197	24.3507	24.3026	23.8528	22.6557	22.1593	21.9751	22.6065	22.2147	22.3929	22.5063	22.2883	5.45896	3.38E-05	O88947	Coagulation factor X OS=Mus musculus OX=10090 GN=F1	F10	-1.36	-1.33	CTRL_7dpi;CTRL_9dpi	TCA
25.0324	25.1015	25.1684	25.0186	24.014	24.1313	23.8079	23.8651	23.1602	23.6281	23.5737	23.4208	6.48432	6.18E-06	O89020	Afamin OS=Mus musculus OX=10090 GN=Afm PE=1 SV=2	Afm	-1.18	-1.74	7dpi_9dpi;CTRL_9dpi;CTRL_7dpi	DD
21.5507	22.3355	21.7552	22.3407	19.1149	19.5264	19.8376	19.1463	16.9695	17.9396	18.737	17.4788	5.32747	4.15E-05	O89020	Afamin OS=Mus musculus OX=10090 GN=Afm PE=1 SV=2	Afm	-1.94	-3.05	7dpi_9dpi;CTRL_9dpi;CTRL_7dpi	DTT
24.4297	24.6298	24.3085	23.5836	22.889	24.5968	23.09	23.6677	22.2834	22.8251	22.7522	22.4689	2.24568	0.0138902	O89020	Afamin OS=Mus musculus OX=10090 GN=Afm PE=1 SV=2	Afm	-0.76	-1.76	CTRL_9dpi	TCA
13.4496	13.7013	NaN	13.5919	16.0872	16.2234	17.0981	17.6664	15.6053	15.51	14.9976	16.995	2.97988	0.00332203	O89053	Coronin-1A OS=Mus musculus OX=10090 GN=Coro1a PE=	Coro1a	2.51	1.68	9dpi_CTRL;7dpi_CTRL	DD
14.1817	12.3624	NaN	NaN	17.4024	17.7739	NaN	NaN	16.0652	17.1544	17.1392	15.8429	2.21133	0.0148255	P00342	L-lactate dehydrogenase C chain OS=Mus musculus OX=1	Ldhc	4.32	3.23	9dpi_CTRL;7dpi_CTRL	DTT
21.7257	19.0438	20.5268	19.6197	23.1527	23.5675	23.4293	23.4195	23.2733	23.2225	23.5544	24.027	3.81397	0.000685705	P00920	Carbonic anhydrase 2 OS=Mus musculus OX=10090 GN=(	Ca2	3.24	3.02	7dpi_CTRL;9dpi_CTRL	DD
19.3199	18.1031	18.4383	18.3035	20.7966	19.9937	21.4115	20.177	19.8295	20.1755	20.7537	18.7382	2.1702	0.0160757	P00920	Carbonic anhydrase 2 OS=Mus musculus OX=10090 GN=(	Ca2	2.24	1.5	7dpi_CTRL	DTT
22.8839	20.0969	21.5144	19.8931	24.5095	23.4743	23.4215	24.1689	24.4442	24.6105	24.7725	25.0154	3.22001	0.00218738	P00920	Carbonic anhydrase 2 OS=Mus musculus OX=10090 GN=(	Ca2	2.77	3.31	7dpi_CTRL;9dpi_CTRL	TCA
28.9846	29.0939	28.7571	28.793	28.9793	29.1335	29.0361	29.1844	28.7076	28.6293	28.5161	28.1958	2.55236	0.00778392	P01027	Complement C3 OS=Mus musculus OX=10090 GN=C3 PE=	C3	0.23	-0.37	CTRL_9dpi;7dpi_9dpi	DD
29.4509	29.565	29.3844	29.3463	29.3998	29.4222	29.5105	29.4	29.099	28.9954	29.0356	28.6489	3.16678	0.00243452	P01027	Complement C3 OS=Mus musculus OX=10090 GN=C3 PE=	C3	-0.25	-0.6	7dpi_9dpi;CTRL_9dpi	TCA
25.7954	25.9084	25.6433	25.8919	26.212	26.2745	26.3713	26.5026	25.8719	25.7708	25.6134	25.7617	3.96684	0.000526089	P01029	Complement C4-B OS=Mus musculus OX=10090 GN=C4b	C4b	0.63	-0.15	7dpi_9dpi;7dpi_CTRL	DD
26.1618	26.3645	26.2761	26.4468	26.6441	26.988	26.9085	26.7906	26.4174	26.3456	26.0972	26.0136	3.10827	0.00268913	P01029	Complement C4-B OS=Mus musculus OX=10090 GN=C4b	C4b	0.58	-0.06	7dpi_9dpi;7dpi_CTRL	TCA
23.7569	23.9434	23.9255	23.9045	23.5202	23.3975	22.9355	23.7508	23.8297	24.0192	23.9077	23.9951	1.98022	0.0236846	P01887	Beta-2-microglobulin OS=Mus musculus OX=10090 GN=B	B2m	-0.39	0.12	CTRL_7dpi;9dpi_7dpi	DD
24.226	24.0979	23.8951	24.1602	23.3037	23.1481	23.5719	23.5358	24.1286	23.8563	23.4571	23.5532	2.24654	0.0138902	P01887	Beta-2-microglobulin OS=Mus musculus OX=10090 GN=B	B2m	-0.64	-0.42	CTRL_7dpi	TCA
16.3859	NaN	16.1628	NaN	20.3839	19.6501	20.2728	20.6915	20.5156	20.707	20.1152	20.3665	5.39212	3.77E-05	P01897	H-2 class I histocompatibility antigen, L-D alpha chain OS=	H2-L	4.05	4.17	7dpi_CTRL;9dpi_CTRL	TCA
25.4576	25.2275	25.1456	24.887	24.5625	24.2209	24.1768	23.6225	24.1413	24.2315	23.9888	24.0249	3.34976	0.00171212	P01898	H-2 class I histocompatibility antigen, Q10 alpha chain OS	H2-Q10	-0.91	-1.08	CTRL_9dpi;CTRL_7dpi	DD
24.7712	25.2708	25.1797	25.5835	24.2138	24.3445	23.9813	24.267	23.5582	23.3442	23.4014	22.8265	4.74793	0.00012091	P01898	H-2 class I histocompatibility antigen, Q10 alpha chain OS	H2-Q10	-1.08	-1.92	7dpi_9dpi;CTRL_9dpi;CTRL_7dpi	DTT
26.0387	26.1553	26.1356	26.0677	25.1175	25.1171	24.6447	25.2238	24.8276	24.7856	24.7	24.5965	5.67459	2.38E-05	P01898	H-2 class I histocompatibility antigen, Q10 alpha chain OS	H2-Q10	-1.59	-1.55	CTRL_9dpi;CTRL_7dpi	TCA
15.5277	16.4073	15.878	16.2501	20.239	19.9469	19.5855	19.2824	19.3585	19.8486	19.1767	19.5517	6.59499	5.53E-06	P01902	H-2 class I histocompatibility antigen, K-D alpha chain OS:	H2-K1	3.73	3.46	9dpi_CTRL;7dpi_CTRL	DD
17.0365	17.1268	17.3369	16.8688	20.2778	19.2577	19.3115	20.3813	20.6168	21.0293	19.833	20.4292	5.17852	5.31E-05	P01902	H-2 class I histocompatibility antigen, K-D alpha chain OS:	H2-K1	2.43	3.44	7dpi_CTRL;9dpi_CTRL	TCA
16.9435	17.7451	18.358	17.9646	21.8329	18.9473	21.0895	22.6577	22.1303	22.2147	22.2228	23.3504	3.46953	0.00138264	P02535	Keratin, type I cytoskeletal 10 OS=Mus musculus OX=100	Krt10	3.12	3.81	7dpi_CTRL;9dpi_CTRL	DTT
19.112	19.2235	19.8774	18.3864	15.9796	13.6967	12.8776	NaN	13.6414	NaN	NaN	NaN	2.66717	0.00617247	P02762	Major urinary protein 6 OS=Mus musculus OX=10090 GN	Mup6	-3.11	-4.04	CTRL_7dpi	DTT
23.267	23.0662	22.9296	23.0781	22.6899	22.8595	22.4614	22.8837	21.8409	22.0931	22.078	21.5635	4.3695	0.000240082	P03953	Complement factor D OS=Mus musculus OX=10090 GN=(	Cfd	-0.35	-1.1	7dpi_9dpi;CTRL_9dpi	DD
21.9162	22.8873	22.7144	23.1326	22.6296	22.7101	22.9261	21.8175	20.8312	20.8853	21.5924	21.2352	2.69801	0.00587004	P03953	Complement factor D OS=Mus musculus OX=10090 GN=(	Cfd	-0.33	-1.33	7dpi_9dpi;CTRL_9dpi	DTT
22.1018	23.0637	22.8284	23.2902	22.5401	23.5247	23.2459	22.8531	21.0096	20.9998	21.7147	21.0307	3.41542	0.00153909	P03953	Complement factor D OS=Mus musculus OX=10090 GN=(	Cfd	-0.07	-1.79	CTRL_9dpi;7dpi_9dpi	TCA
19.0482	18.0441	18.9737	18.1661	20.952	19.7194	20.9663	21.3231	21.3642	21.0413	21.5541	22.5901	3.65591	0.000950177	P04104	Keratin, type II cytoskeletal 1 OS=Mus musculus OX=100	Krt1	2.27	3.33	7dpi_CTRL;9dpi_CTRL	DTT
24.5856	24.7313	24.7977	24.8537	25.6198	25.7311	25.8089	25.9756	25.3315	25.5298	25.497	25.1073	4.69093	0.000130278	P04186	Complement factor B OS=Mus musculus OX=10090 GN=(	Cfb	1.05	0.52	9dpi_CTRL;7dpi_CTRL;7dpi_9dpi	DD
25.0821	25.2438	25.3026	25.6037	26.0157	26.4933	26.7409	26.1887	25.8792	26.003	26.0248	25.477	2.90342	0.0038693	P04186	Complement factor B OS=Mus musculus OX=10090 GN=(	Cfb	1.02	0.52	9dpi_CTRL;7dpi_CTRL	TCA
21.1174	19.4444	20.6481	21.4932	23.0381	22.8364	22.3709	23.1346	20.9381	21.4183	22.4642	21.1042	2.34924	0.0115227	P04247	Myoglobin OS=Mus musculus OX=10090 GN=Mb PE=1 S	Mb	1.65	0.3	7dpi_CTRL;7dpi_9dpi	DD
15.5397	15.4465	15.0403	16.1257	18.5733	NaN	17.6839	18.2948	18.2996	19.2007	17.851	20.1367	3.36602	0.001667	P04441	H-2 class II histocompatibility antigen gamma chain OS=M	Cd74	1.69	3.15	7dpi_CTRL;9dpi_CTRL	DTT
NaN	14.7376	NaN	14.1962	19.8522	20.2644	20.4177	20.4176	18.486	19.0068	16.4885	19.6161	3.26532	0.00201046	P04918	Serum amyloid A-3 protein OS=Mus musculus OX=10090	Saa3	4.64	3.32	9dpi_CTRL;7dpi_CTRL	DD
NaN	NaN	NaN	NaN	19.4	18.5992	18.4929	19.6464	17.8137	18.0868	17.7787	18.3948	1.71152	0.0389163	P04918	Serum amyloid A-3 protein OS=Mus musculus OX=10090	Saa3	6.64	6.64	CTRL_7dpi	TCA
18.7786	14.802	17.6037	17.3223	20.444	21.6407	20.4976	21.0786	20.604	20.3199	21.0581	19.9974	2.92437	0.00370349	P05064	Fructose-bisphosphate aldolase A OS=Mus musculus OX=	Aldoa	2.4	1.5	9dpi_CTRL;7dpi_CTRL	DD
20.2329	18.1966	19.5269	20.0276	21.4267	23.4254	23.4027	21.7393	20.5071	20.3532	21.8686	21.2489	2.41363	0.0102636	P05064	Fructose-bisphosphate aldolase A OS=Mus musculus OX=	Aldoa	2.36	1.26	7dpi_CTRL	TCA
19.3398	18.5722	17.5078	17.1716	27.8778	27.5776	28.1326	28.0342	27.0613	26.8898	25.2022	26.7913	7.32137	2.00E-06	P05366	Serum amyloid A-1 protein OS=Mus musculus OX=10090	Saa1	6.64	6.64	9dpi_CTRL;7dpi_CTRL	DD
22.4293	21.6444	21.5111	20.5002	29.5795	29.5695	29.5642	30.0111	29.2125	29.0056	27.3636	29.6359	6.83887	4.12E-06	P05366	Serum amyloid A-1 protein OS=Mus musculus OX=10090	Saa1	6.64	6.34	9dpi_CTRL;7dpi_CTRL	DTT
20.2388	20.181	19.525	20.3174	26.9229	27.4694	27.5571	27.1596	26.3853	26.1576	24.3365	26.8294	6.55352	5.90E-06	P05366	Serum amyloid A-1 protein OS=Mus musculus OX=10090	Saa1	6.64	5.55	9dpi_CTRL;7dpi_CTRL	TCA
16.2473	16.4504	16.5149	NaN	25.9669	25.988	26.3088	26.1106	25.2044	24.7658	23.0417	25.8143	6.50746	6.18E-06	P05367	Serum amyloid A-2 protein OS=Mus musculus OX=10090	Saa2	3.96	1.88	9dpi_CTRL;7dpi_CTRL	DD
18.8718	19.1425	19.6436	18.7563	28.479	28.5968	28.3009	28.9294	28.0634	27.6365	25.29	27.9217	7.07511	2.87E-06	P05367	Serum amyloid A-2 protein OS=Mus musculus OX=10090	Saa2	6.64	6.64	9dpi_CTRL;7dpi_CTRL	DTT
13.7804	16.1004	14.6326	16.0371	27.0911	27.5078	27.5055	27.362	25.6295	25.2402	23.9399	26.1391	7.7498	1.03E-06	P05367	Serum amyloid A-2 protein OS=Mus musculus OX=10090	Saa2	4.57	1.62	9dpi_CTRL;7dpi_CTRL;7dpi_9dpi	TCA
NaN	NaN	NaN	NaN	15.2126	18.1729	17.47	18.192	15.7969	14.517	14.6801	14.5835	1.67068	0.0423881	P05977	Myosin light chain 1/3, skeletal muscle isoform OS=Mus r	Myl1	6.64	6.64	CTRL_7dpi	DTT
18.1479	NaN	16.3757	17.3067	21.3812	22.1629	21.4676	22.0165	21.5735	21.7424	21.0791	21.8716	5.07626	6.36E-05	P06151	L-lactate dehydrogenase A chain OS=Mus musculus OX=1	Ldha	3.1	2.62	9dpi_CTRL;7dpi_CTRL	DD
18.8361	16.1976	17.4208	17.9718	22.2306	22.3001	22.222	22.1002	21.7984	21.6357	21.8175	22.1007	5.26458	4.55E-05	P06151	L-lactate dehydrogenase A chain OS=Mus musculus OX=1	Ldha	3.15	2.96	9dpi_CTRL;7dpi_CTRL	TCA
14.0555	15.2327	14.4122	14.312	16.8289	NaN	NaN	16.5965	18.3484	18.7776	18.8331	17.6008	4.40033	0.00022727	P06332	T-cell surface glycoprotein CD4 OS=Mus musculus OX=10	Cd4	2.35	3.99	7dpi_CTRL;9dpi_CTRL;9dpi_7dpi	TCA
23.5343	23.472	23.4059	23.606	22.9332	23.44	23.3869	23.0816	21.9251	21.654	21.771	21.7297	6.64139	5.16E-06	P06683	Complement component C9 OS=Mus musculus OX=1009	C9	-0.3	-1.58	7dpi_9dpi;CTRL_9dpi	DD
20.2843	21.0199	20.6604	20.7458	17.979	17.8371	17.8854	15.7404	NaN	NaN	NaN	17.5861	2.97941	0.00332203	P06683	Complement component C9 OS=Mus musculus OX=1009	C9	-1.27	-2.91	CTRL_7dpi	DTT
24.3347	24.3786	24.4052	24.3134	23.9077	23.9287	23.8508	23.9858	22.6908	22.8308	22.8406	22.7052	9.93667	4.03E-08	P06683	Complement component C9 OS=Mus musculus OX=1009	C9	-0.28	-1.56	7dpi_9dpi;CTRL_9dpi;CTRL_7dpi	TCA
27.4907	27.3993	27.413	27.1927	27.6661	27.2624	27.7928	27.3069	28.8696	28.5535	27.9033	28.7291	3.09451	0.00274694	P06728	Apolipoprotein A-IV OS=Mus musculus OX=10090 GN=Ap	Apoa4	0.11	1.26	9dpi_CTRL;9dpi_7dpi	DD
29.1664	29.0603	29.5626	28.9014	29.4653	28.6786	28.7944	29.8016	30.8798	30.4747	29.5002	30.9188	1.98928	0.023271	P06728	Apolipoprotein A-IV OS=Mus musculus OX=10090 GN=Ap	Apoa4	0.11	1.4	9dpi_CTRL;9dpi_7dpi	DTT
26.4735	26.5243	26.4761	26.6896	26.596	25.866	25.9582	26.3791	27.7755	27.6036	26.2891	27.5323	1.72924	0.0375766	P06728	Apolipoprotein A-IV OS=Mus musculus OX=10090 GN=Ap	Apoa4	-0.22	1.06	9dpi_7dpi	TCA
NaN	NaN	NaN	NaN	16.8521	17.0496	16.9959	17.612	16.2001	16.69	15.6752	15.0969	1.71954	0.0383149	P06745	Glucose-6-phosphate isomerase OS=Mus musculus OX=1	Gpi	6.64	6.64	CTRL_7dpi	DD
26.0717	26.4069	26.2873	26.4924	27.1753	27.2617	27.3531	27.2541	27.0937	27.1141	26.9531	26.6878	4.31349	0.000264567	P06909	Complement factor H OS=Mus musculus OX=10090 GN=(	Cfh	0.85	0.63	9dpi_CTRL;7dpi_CTRL	DD
25.9862	26.1324	26.1879	26.4077	26.6909	27.2009	27.0416	26.9101	26.6674	26.8655	26.7582	26.4473	3.09052	0.00274694	P06909	Complement factor H OS=Mus musculus OX=10090 GN=(	Cfh	0.76	0.54	9dpi_CTRL;7dpi_CTRL	TCA
28.0578	28.0096	28.2187	28.1374	26.8985	26.9148	26.9635	27.063	26.6231	26.7195	26.7531	26.9473	7.41138	1.80E-06	P07309	Transthyretin OS=Mus musculus OX=10090 GN=Ttr PE=1	Ttr	-1.23	-1.52	CTRL_9dpi;CTRL_7dpi	DD
28.0144	27.9177	28.2838	27.9021	27.4279	27.2022	27.2209	27.6692	26.9371	26.7817	27.2168	27.0258	3.95685	0.00053459	P07309	Transthyretin OS=Mus musculus OX=10090 GN=Ttr PE=1	Ttr	-0.46	-1.16	7dpi_9dpi;CTRL_9dpi;CTRL_7dpi	TCA
18.5955	NaN	14.9375	17.6209	20.5865	22.5998	20.9699	22.0078	20.586	20.6421	21.9217	20.8038	2.64964	0.00637427	P07310	Creatine kinase M-type OS=Mus musculus OX=10090 GN	Ckm	2.83	1.19	9dpi_CTRL;7dpi_CTRL	DD
15.8858	16.3446	15.2425	NaN	23.0873	23.7169	23.5625	24.2495	22.945	22.1588	22.7324	23.1034	7.31209	2.00E-06	P07361	Alpha-1-acid glycoprotein 2 OS=Mus musculus OX=10090	Orm2	6.64	6.64	9dpi_CTRL;7dpi_CTRL	DD
21.3605	22.5245	23.5658	21.9298	26.2517	26.5286	25.857	25.2817	25.0422	25.4684	25.1378	25.6502	4.28007	0.00028133	P07361	Alpha-1-acid glycoprotein 2 OS=Mus musculus OX=10090	Orm2	3.56	2.73	9dpi_CTRL;7dpi_CTRL	DTT
15.8678	16.6822	14.8849	15.7942	24.0866	24.3151	24.736	24.3252	23.7702	23.5827	23.9131	23.3788	8.82123	2.75E-07	P07361	Alpha-1-acid glycoprotein 2 OS=Mus musculus OX=10090	Orm2	6.64	6.64	9dpi_CTRL;7dpi_CTRL	TCA
33.1814	33.3194	33.3367	33.3666	33.1275	33.0052	32.9779	33.1404	33.0896	33.1093	33.1142	32.9773	2.56836	0.00753246	P07724	Serum albumin OS=Mus musculus OX=10090 GN=Alb PE=	Alb	-0.3	-0.25	CTRL_7dpi;CTRL_9dpi	DD
31.7212	31.845	31.8311	31.9819	31.3176	31.3596	31.3432	31.3686	31.2378	31.3894	31.3243	31.3419	5.35768	3.92E-05	P07724	Serum albumin OS=Mus musculus OX=10090 GN=Alb PE=	Alb	-0.73	-0.86	CTRL_9dpi;CTRL_7dpi	TCA
26.4262	26.5017	26.1588	26.2037	25.641	25.6337	25.7507	26.1404	24.7229	25.0587	24.8521	24.4554	4.87818	9.51E-05	P07758	Alpha-1-antitrypsin 1-1 OS=Mus musculus OX=10090 GN=	Serpina1a	-0.74	-1.45	7dpi_9dpi;CTRL_9dpi;CTRL_7dpi	DD
28.332	28.5972	28.5393	28.2638	27.9228	27.6274	27.3757	27.8939	27.2182	26.9343	27.2706	27.1249	4.67216	0.000134795	P07758	Alpha-1-antitrypsin 1-1 OS=Mus musculus OX=10090 GN=	Serpina1a	-0.89	-1.49	7dpi_9dpi;CTRL_9dpi;CTRL_7dpi	TCA
30.3873	30.1258	30.3558	30.4575	29.6092	29.7839	29.8081	29.8832	29.9397	29.7079	29.7634	29.9083	3.63775	0.00097868	P07759	Serine protease inhibitor A3K OS=Mus musculus OX=100	Serpina3k	-0.5	-0.6	CTRL_7dpi;CTRL_9dpi	DD
30.3198	30.3168	30.4968	30.7097	29.9631	30.1606	30.2243	30.1653	30.2806	29.9837	30.0185	29.9174	1.96825	0.0241892	P07759	Serine protease inhibitor A3K OS=Mus musculus OX=100	Serpina3k	-0.22	-0.41	CTRL_9dpi;CTRL_7dpi	TCA
16.4107	NaN	15.4557	15.1912	17.3528	16.2504	17.2251	17.9189	15.0429	15.2897	15.0414	NaN	2.28897	0.0129145	P08228	Superoxide dismutase [Cu-Zn] OS=Mus musculus OX=100	Sod1	1.64	-0.41	7dpi_9dpi;7dpi_CTRL	DD

22.0323	19.4521	21.1745	19.9925	22.0008	21.0196	19.8158	21.9645	24.4189	23.8043	23.7592	23.4213	2.71376	0.00568488	P08228	Superoxide dismutase [Cu-Zn] OS=Mus musculus OX=10(	Sod1		0.99	1.16	9dpi_CTRL;9dpi_7dpi	DTT
16.3111	14.7222	16.1439	15.4753	18.6109	16.7574	18.6264	20.0881	19.2834	19.3931	19.5135	20.8927	3.14621	0.00253034	P08730	Keratin, type I cytoskeletal 13 OS=Mus musculus OX=100	Krt13		2.81	3.98	7dpi_CTRL;9dpi_CTRL	DTT
18.4822	19.0911	18.8889	19.358	20.0838	20.0391	20.3702	19.9957	19.5891	19.5608	19.9534	19.3366	3.13222	0.00258323	P09581	Macrophage colony-stimulating factor 1 receptor OS=Mu	Csf1r		1.04	0.55	9dpi_CTRL;7dpi_CTRL	DD
27.0231	27.0415	27.0297	26.8831	25.8661	25.4452	25.6619	25.5979	25.3518	25.5141	24.9775	23.514	3.00246	0.00318259	P09813	Apolipoprotein A-II OS=Mus musculus OX=10090 GN=Ap(	Apoa2		-1.28	-1.47	CTRL_9dpi;CTRL_7dpi	DD
28.5223	28.4805	29.2672	28.2237	27.3011	27.5323	25.6388	27.6069	27.4042	27.6523	27.4677	28.0782	2.45627	0.00950219	P09813	Apolipoprotein A-II OS=Mus musculus OX=10090 GN=Ap(	Apoa2		-0.25	-0.21	CTRL_7dpi;CTRL_9dpi	DTT
23.6951	24.2278	24.5503	24.4619	23.1325	23.8099	24.2843	23.4548	22.0391	21.2534	22.5964	21.7327	3.38416	0.00160775	P09813	Apolipoprotein A-II OS=Mus musculus OX=10090 GN=Ap(	Apoa2		-2.17	-2.87	7dpi_9dpi;CTRL_9dpi;CTRL_7dpi	TCA
15.2356	NaN	NaN	14.7644	17.978	19.0521	18.6979	18.1073	17.0483	17.2405	17.6211	17.4042	4.22925	0.000306818	P10126	Elongation factor 1-alpha 1 OS=Mus musculus OX=10090	Eef1a1		3.4	2.33	9dpi_CTRL;7dpi_CTRL;7dpi_9dpi	TCA
19.305	18.0987	18.9707	18.9992	20.0708	20.6309	20.1443	20.6145	19.2438	19.2222	18.8287	19.5228	3.07755	0.00281652	P10404	MLV-related proviral Env polyprotein OS=Mus musculus (		1	2.08	0.33	7dpi_CTRL;7dpi_9dpi	DD
19.1269	19.0138	19.0056	19.082	19.8598	20.2901	19.5026	20.1354	19.5663	19.8251	19.7385	19.5367	3.10258	0.00271116	P10605	Cathepsin B OS=Mus musculus OX=10090 GN=Ctsb PE=1	Ctsb		1.1	0.61	9dpi_CTRL;7dpi_CTRL	DD
16.4216	NaN	15.8543	NaN	17.2712	17.9001	17.8256	17.5664	15.8479	16.2308	16.9374	17.0788	2.00929	0.0223687	P10639	Thioredoxin OS=Mus musculus OX=10090 GN=Txn PE=1 S	Txn		1.45	0.45	7dpi_CTRL;7dpi_9dpi	DD
16.9156	18.3288	18.8569	18.6802	NaN	NaN	NaN	NaN	NaN	14.7641	14.6205	NaN	2.28811	0.0129145	P11087	Collagen alpha-1(I) chain OS=Mus musculus OX=10090 GI	Col1a1		-6.64	-3.12	CTRL_7dpi	DTT
26.3121	25.9058	26.0167	25.9729	26.8981	26.6497	27.0234	26.5254	26.9873	26.6703	26.3235	26.2703	2.06913	0.0197486	P11276	Fibronectin OS=Mus musculus OX=10090 GN=Fn1 PE=1 S	Fn1		0.8	0.61	9dpi_CTRL;7dpi_CTRL	DD
22.7234	22.997	23.277	22.9336	22.5452	21.997	20.9894	22.331	22.9928	23.1118	23.0658	NaN	1.77011	0.0353244	P11404	Fatty acid-binding protein, heart OS=Mus musculus OX=1	Fabp3		-0.6	0.1	CTRL_7dpi;9dpi_7dpi	DD
25.2203	25.5413	24.8619	25.1787	21.4688	22.2898	21.8209	22.3032	20.6372	18.2416	19.0138	19.4771	5.80232	1.85E-05	P11589	Major urinary protein 2 OS=Mus musculus OX=10090 GN	Mup2		-2.95	-5.21	7dpi_9dpi;CTRL_9dpi;CTRL_7dpi	DD
23.0904	23.575	23.0754	22.1378	19.8379	16.7031	17.3504	20.1315	19.0425	16.7063	18.1793	18.0328	3.38678	0.00160775	P11589	Major urinary protein 2 OS=Mus musculus OX=10090 GN	Mup2		-2.95	-4.44	CTRL_9dpi;CTRL_7dpi	TCA
15.5585	16.0199	NaN	NaN	20.9507	20.8404	20.6732	22.0486	18.3973	19.07	16.9025	18.261	3.78226	0.000728314	P11672	Neutrophil gelatinase-associated lipocalin OS=Mus musc(	Lcn2		3.14	1.38	9dpi_CTRL;7dpi_CTRL;7dpi_9dpi	DD
NaN	NaN	NaN	NaN	21.4751	19.7164	22.0145	21.3683	18.7015	18.5169	17.5198	18.4997	2.62258	0.00672908	P11672	Neutrophil gelatinase-associated lipocalin OS=Mus musc(	Lcn2		6.64	6.64	CTRL_7dpi	TCA
22.1075	22.5982	22.3199	22.2513	21.774	21.7646	21.6686	21.4682	21.6303	21.5152	21.6005	21.3766	3.87487	0.000611678	P11680	Properdin OS=Mus musculus OX=10090 GN=Cfp PE=2 SV:	Cfp		-0.54	-0.67	CTRL_9dpi;CTRL_7dpi	DD
23.4574	24.404	24.1325	24.0022	22.7202	22.0423	21.9029	22.3399	21.9809	22.1477	22.1326	21.9473	4.72213	0.000126836	P11680	Properdin OS=Mus musculus OX=10090 GN=Cfp PE=2 SV:	Cfp		-1.69	-1.86	CTRL_9dpi;CTRL_7dpi	TCA
21.35	21.5298	21.414	21.549	21.9387	22.0855	21.8281	22.6145	22.2489	21.8981	21.783	21.6875	1.84414	0.0304239	P11859	Angiotensinogen OS=Mus musculus OX=10090 GN=Agt P	Agt		0.68	0.57	7dpi_CTRL	DD
21.7749	21.8143	21.895	21.679	22.186	21.4551	22.4417	22.2299	22.7281	22.534	22.5902	22.2989	1.92804	0.0263655	P11859	Angiotensinogen OS=Mus musculus OX=10090 GN=Agt P	Agt		0.41	0.61	9dpi_CTRL	TCA
24.0314	24.2381	23.5913	23.5365	26.2368	26.5641	26.391	26.5284	26.5978	26.4502	26.1452	26.2183	6.81364	4.12E-06	P12246	Serum amyloid P-component OS=Mus musculus OX=100!	Apcs		2.78	2.52	9dpi_CTRL;7dpi_CTRL	DD
24.5633	24.6786	24.055	23.9084	26.8197	27.0258	27.0491	26.8352	27.0632	26.8398	26.6863	26.4836	6.49261	6.18E-06	P12246	Serum amyloid P-component OS=Mus musculus OX=100!	Apcs		2.63	2.35	9dpi_CTRL;7dpi_CTRL	TCA
25.5177	25.4482	25.5504	25.5984	24.6879	24.9333	24.609	24.8682	24.2092	24.446	24.4882	24.5087	5.82981	1.78E-05	P13020	Gelsolin OS=Mus musculus OX=10090 GN=Gsn PE=1 SV=:	Gsn		-0.62	-1.39	7dpi_9dpi;CTRL_9dpi;CTRL_7dpi	DD
22.4048	22.2688	22.6777	22.0322	18.9387	19.1697	17.8287	17.8366	19.6859	20.789	21.4312	17.4804	2.58071	0.00735069	P13020	Gelsolin OS=Mus musculus OX=10090 GN=Gsn PE=1 SV=:	Gsn		-2.4	-1.66	CTRL_7dpi;CTRL_9dpi	DTT
25.9661	25.9784	26.2377	26.4472	25.1919	23.5918	24.5999	24.9959	24.4659	24.7276	24.7819	24.4451	3.002	0.00318259	P13020	Gelsolin OS=Mus musculus OX=10090 GN=Gsn PE=1 SV=:	Gsn		-1.11	-1.49	CTRL_7dpi;CTRL_9dpi	TCA
20.765	21.1988	20.8887	20.3397	19.3278	19.4519	19.1374	18.6212	19.2781	19.5317	20.3837	19.5016	3.09085	0.00274694	P14106	Complement C1q subcomponent subunit B OS=Mus mus	C1qb		-1.35	-1.09	CTRL_7dpi;CTRL_9dpi	DD
22.2754	22.5154	22.6765	22.7238	20.2424	21.0296	20.3834	20.2975	20.5753	20.7522	21.6555	20.101	3.67595	0.000912961	P14106	Complement C1q subcomponent subunit B OS=Mus mus	C1qb		-2.34	-1.93	CTRL_7dpi;CTRL_9dpi	TCA
16.8899	15.1045	15.4989	17.9294	20.1504	20.1666	19.8156	20.3904	17.8536	19.1734	19.2196	19.4831	3.27889	0.0019611	P14152	Malate dehydrogenase, cytoplasmic OS=Mus musculus O	Mdh1		2.52	1.5	9dpi_CTRL;7dpi_CTRL	DD
19.07	14.7651	18.3719	19.0492	21.1119	20.7428	20.9889	20.7239	19.9392	20.4421	20.4836	20.646	1.91213	0.0270034	P14152	Malate dehydrogenase, cytoplasmic OS=Mus musculus O	Mdh1		2.13	1.52	9dpi_CTRL;7dpi_CTRL	TCA
15.2113	14.8132	15.2681	15.0522	18.3373	18.6767	18.6977	18.4566	18.4165	18.225	17.8262	18.2278	8.38025	3.23E-07	P14426	H-2 class I histocompatibility antigen, D-K alpha chain OS	H2-D1		3.45	3.08	9dpi_CTRL;7dpi_CTRL	TCA
16.5321	16.9458	16.7475	17.065	20.6481	20.6046	20.6583	20.7372	20.58	20.8776	20.0689	20.5892	8.44119	3.15E-07	P14430	H-2 class I histocompatibility antigen, Q8 alpha chain OS	H2-Q8		3.82	3.73	9dpi_CTRL;7dpi_CTRL	DD
16.8609	16.1262	16.1813	15.9472	19.874	18.7422	19.2666	19.9631	20.0949	20.565	19.9176	20.4247	5.9984	1.30E-05	P14430	H-2 class I histocompatibility antigen, Q8 alpha chain OS	H2-Q8		3.12	3.97	7dpi_CTRL;9dpi_CTRL	TCA
19.735	19.393	19.5438	19.5691	21.0408	20.869	21.0974	21.1442	20.0443	20.6544	21.2612	20.1584	3.29317	0.00190789	P14847	C-reactive protein OS=Mus musculus OX=10090 GN=Crp	Crp		0.92	-0.12	9dpi_CTRL;7dpi_CTRL	DD
20.7414	20.6403	21.5543	19.8171	22.3749	22.2413	22.0129	21.7629	21.684	22.1089	21.9976	21.116	2.03787	0.0211524	P14847	C-reactive protein OS=Mus musculus OX=10090 GN=Crp	Crp		0.9	0.3	9dpi_CTRL;7dpi_CTRL	TCA
17.7852	17.1722	17.555	16.2306	18.2567	16.4036	17.1285	18.1366	18.9402	19.9257	19.1174	18.4557	1.99623	0.0229761	P15327	Bisphosphoglycerate mutase OS=Mus musculus OX=100!	Bpgm		0.41	1.75	9dpi_CTRL;9dpi_7dpi	TCA
17.7649	16.3052	16.7314	17.6379	20.5114	18.7357	18.133	18.6018	17.8788	18.0779	18.3485	18.0097	1.75713	0.0358614	P16125	L-lactate dehydrogenase B chain OS=Mus musculus OX=1	Ldhb		1.56	1.12	7dpi_CTRL	TCA
21.2421	21.407	21.2283	21.2019	22.9469	22.8696	23.2112	22.9771	22.5107	22.5617	22.1505	21.9238	5.59457	2.65E-05	P16301	Phosphatidylcholine-sterol acyltransferase OS=Mus musc	Lcat		1.82	1.09	9dpi_CTRL;7dpi_CTRL;7dpi_9dpi	DD
20.8303	17.7079	19.6796	19.976	22.0048	23.304	23.0577	22.0596	20.2141	20.5562	22.5165	21.6331	2.07965	0.0194049	P16858	Glyceraldehyde-3-phosphate dehydrogenase OS=Mus m	Gapdh		2.49	1.31	7dpi_CTRL	TCA
14.9218	NaN	15.1079	NaN	19.0339	19.28	19.267	18.4434	17.763	17.5928	17.8632	18.378	5.01103	7.31E-05	P17182	Alpha-enolase OS=Mus musculus OX=10090 GN=Eno1 PE	Eno1		3.81	2.75	9dpi_CTRL;7dpi_CTRL;7dpi_9dpi	TCA
16.4818	NaN	NaN	16.0695	21.7017	21.4264	17.6381	18.5483	15.6631	15.7745	17.5276	16.8333	1.61672	0.0477241	P17751	Triosephosphate isomerase OS=Mus musculus OX=1009(	Tpi1		1.29	0.03	7dpi_9dpi	DD
15.6404	NaN	15.1355	17.0338	19.8441	20.7307	20.1203	19.7793	16.164	17.4227	19.4566	17.8104	2.74365	0.00541248	P17751	Triosephosphate isomerase OS=Mus musculus OX=1009(	Tpi1		2.36	0.95	7dpi_CTRL;7dpi_9dpi	TCA
12.9329	NaN	NaN	13.5238	16.1878	14.5233	15.8565	16.5028	13.9073	14.4824	NaN	NaN	1.65515	0.0438068	P18337	L-selectin OS=Mus musculus OX=10090 GN=Sell PE=1 SV:	Sell		2.79	0.97	7dpi_CTRL	DD
NaN	14.1576	NaN	15.6483	17.7054	18.4058	18.3841	17.9034	17.0134	17.0052	NaN	15.6614	2.38361	0.010786	P18337	L-selectin OS=Mus musculus OX=10090 GN=Sell PE=1 SV:	Sell		3.15	1.43	7dpi_CTRL	TCA
26.1854	25.9418	25.7037	26.0368	25.4007	25.5315	25.5133	25.203	25.297	25.3986	25.5848	25.2171	2.85221	0.00425539	P19221	Prothrombin OS=Mus musculus OX=10090 GN=F2 PE=1 S	F2		-0.31	-0.47	CTRL_9dpi;CTRL_7dpi	DD
26.1175	26.4094	26.1711	26.0515	25.7059	25.3986	25.5917	25.7555	26.1933	25.5667	25.5684	25.4433	1.84688	0.0303255	P19221	Prothrombin OS=Mus musculus OX=10090 GN=F2 PE=1 S	F2		-0.63	-0.5	CTRL_7dpi;CTRL_9dpi	TCA
16.2388	16.388	16.1217	15.8045	17.4922	17.685	17.8758	17.9807	18.4651	18.1086	17.8743	18.7093	5.24658	4.59E-05	P20029	Endoplasmic reticulum chaperone BiP OS=Mus musculus	Hspa5		1.54	1.82	7dpi_CTRL;9dpi_CTRL	TCA
27.5114	27.713	27.5004	27.5755	27.247	27.1861	27.1725	27.2234	26.9337	27.1916	27.1526	27.0074	4.0951	0.000399949	P20918	Plasminogen OS=Mus musculus OX=10090 GN=Plg PE=1	Plg		-0.34	-0.49	CTRL_9dpi;CTRL_7dpi	DD
27.5141	27.6537	27.6065	27.8143	26.946	27.3831	27.4702	26.964	26.8019	27.0544	27.0947	26.8931	2.68716	0.00594374	P20918	Plasminogen OS=Mus musculus OX=10090 GN=Plg PE=1	Plg		-0.58	-0.6	CTRL_9dpi;CTRL_7dpi	TCA
21.397	21.2976	21.243	21.2191	22.3003	22.5689	22.4138	22.0961	22.2181	22.2913	21.9115	22.4146	4.61179	0.000152134	P21180	Complement C2 OS=Mus musculus OX=10090 GN=C2 PE:	C2		1.11	0.74	9dpi_CTRL;7dpi_CTRL	DD
22.334	22.4805	22.5615	22.6968	23.2265	23.5722	23.5149	23.3414	22.8498	22.7057	22.7595	23.0317	4.29442	0.000274296	P21180	Complement C2 OS=Mus musculus OX=10090 GN=C2 PE:	C2		1.09	0.66	9dpi_CTRL;7dpi_CTRL;7dpi_9dpi	TCA
27.4729	27.6342	27.6891	27.6023	27.4701	27.6846	27.628	27.9233	27.2488	27.3713	27.3409	27.2497	2.26069	0.0136581	P21614	Vitamin D-binding protein OS=Mus musculus OX=10090 (	Gc		0.18	-0.35	CTRL_9dpi;7dpi_9dpi	DD
29.2749	29.0923	29.0167	29.1925	28.6206	28.8316	28.9991	28.9418	28.4387	28.6089	28.378	28.4535	3.81779	0.00068409	P22599	Alpha-1-antitrypsin 1-2 OS=Mus musculus OX=10090 GN:	Serpina1b		-0.33	-0.67	7dpi_9dpi;CTRL_9dpi;CTRL_7dpi	DD
27.7109	27.8429	27.7521	27.8467	26.4606	26.3791	26.232	26.2406	25.6148	25.9067	26.0406	25.0382	5.39566	3.77E-05	P23953	Carboxylesterase 1C OS=Mus musculus OX=10090 GN=Ce	Ces1c		-1.54	-2.19	7dpi_9dpi;CTRL_9dpi;CTRL_7dpi	DD
26.8379	27.3298	26.2981	28.2058	26.0354	26.0221	26.2685	26.5706	25.2458	24.5121	25.0287	23.736	3.0406	0.00298023	P23953	Carboxylesterase 1C OS=Mus musculus OX=10090 GN=Ce	Ces1c		-1.06	-2.58	7dpi_9dpi;CTRL_9dpi	DTT
27.5602	27.6985	27.7432	27.9544	26.4097	26.3322	26.6469	26.548	26.0061	26.0717	26.4561	25.5348	4.713	0.000126836	P23953	Carboxylesterase 1C OS=Mus musculus OX=10090 GN=Ce	Ces1c		-1.49	-2.17	7dpi_9dpi;CTRL_9dpi;CTRL_7dpi	TCA
17.453	15.9217	16.9197	15.9295	18.6572	18.7319	18.2268	18.7205	18.922	19.9026	19.4428	19.8099	4.14667	0.000362946	P24270	Catalase OS=Mus musculus OX=10090 GN=Cat PE=1 SV=4	Cat		1.27	1.4	7dpi_CTRL;9dpi_CTRL	TCA
23.7774	24.0312	23.7956	24.1045	22.5667	22.8213	22.5984	22.3826	22.7098	22.7566	22.5911	22.6922	6.20347	9.69E-06	P26262	Plasma kallikrein OS=Mus musculus OX=10090 GN=Klkb1	Klkb1		-1.28	-1.33	CTRL_7dpi;CTRL_9dpi	DD
24.0862	24.3589	24.1951	24.4266	22.9971	22.5375	22.3941	22.9489	23.1388	23.0364	23.1028	22.9701	5.39355	3.77E-05	P26262	Plasma kallikrein OS=Mus musculus OX=10090 GN=Klkb1	Klkb1		-1.37	-1.12	CTRL_7dpi;CTRL_9dpi	TCA
NaN	NaN	NaN	NaN	16.3273	16.4205	16.0241	16.0621	15.2165	15.8756	15.5559	15.6957	1.97382	0.0239581	P26645	Myristoylated alanine-rich C-kinase substrate OS=Mus m	Marcks		6.64	6.64	CTRL_7dpi	DD
29.8725	29.8411	29.7167	29.6171	28.667	28.6914	28.8006	28.4732	29.2284	28.9445	29.2077	28.9597	5.38509	3.78E-05	P28665	Murinoglobulin-1 OS=Mus musculus OX=10090 GN=Mug	Mug1		-0.98	-0.75	9dpi_7dpi;CTRL_7dpi;CTRL_9dpi	DD
26.8939	27.5778	26.9047	27.2463	25.2486	25.8252	25.6108	24.8123	25.2644	25.7545	26.7416	23.486	1.67184	0.0423881	P28665	Murinoglobulin-1 OS=Mus musculus OX=10090 GN=Mug	Mug1		-1.83	-1.63	CTRL_9dpi;CTRL_7dpi	DTT
30.1333	30.1842	30.1315	30.1508	29.2334	29.279	29.1288	29.2236	29.5678	29.1807	29.6134	29.1767	4.81402	0.000105898	P28665	Murinoglobulin-1 OS=Mus musculus OX=10090 GN=Mug	Mug1		-1.01	-0.8	CTRL_7dpi;CTRL_9dpi	TCA
NaN	NaN	NaN	NaN	NaN	NaN	15.2805	15.9309	17.5553	18.5941	18.1399	NaN	1.91437	0.0269496	P28667	MARCKS-related protein OS=Mus musculus OX=10090 GI	Marcksl1		6.64	6.64	7dpi_CTRL	DTT
18.239	18.8232	18.6515	18.5779	21.3052	21.4984	21.151	21.4173	20.937	20.8579	19.5187	20.2094	4.714	0.000126836	P28798	Progranulin OS=Mus musculus OX=10090 GN=Grn PE=1 S	Grn		2.41	1.74	9dpi_CTRL;7dpi_CTRL;7dpi_9dpi	DD
20.8096	20.7369	19.7807	20.0649	22.7099	22.9511	22.7815	23.9713	23.2492	23.4639	22.4308	23.1059	4.36221	0.000242164	P28798	Progranulin OS=Mus musculus OX=10090 GN=Grn PE=1 S	Grn		2.53	2.58	9dpi_CTRL;7dpi_CTRL	DTT
17.0883	17.9138	17.0916	17.3446	19.1132	19.1736	18.5723	19.2196	17.9227	18.3892	17.7536	18.6484	3.31663	0.00182718	P28798	Progranulin OS=Mus musculus OX=10090 GN=Grn PE=1 S	Grn		1.43	0.35	9dpi_CTRL;7dpi_CTRL;7dpi_9dpi	TCA
24.4731	24.6982	23.9988	24.394	24.2516	23.2895	23.5522	24.365	23.3122	23.4765	23.0726	23.2611	2.22132	0.0145419	P29788	Vitronectin OS=Mus musculus OX=10090 GN=Vtn PE=1 S	Vtn		-0.25	-0.71	CTRL_9dpi	DTT
25.2936	25.524	25.4123	25.4617	25.6739	25.7791	25.542	25.4353	25.2052	25.2108	25.1854	24.9044	2.65976	0.00625285	P29788	Vitronectin OS=Mus musculus OX=10090 GN=Vtn PE=1 S	Vtn		0.25	-0.23	CTRL_9dpi;7dpi_9dpi	TCA
24.3127	24.4355	23.9375	23.7216	25.0808	25.3243	25.3653	24.8697	24.6132	24.8934	25.5184	24.274	2.84513	0.00431015	P31532	Serum amyloid A-4 protein OS=Mus musculus OX=10090	Saa4		1.11	0.5	9dpi_CTRL;7dpi_9dpi	DD
26.9488	27.1617	27.1505	27.2727	26.1894	26.2698	25.6642	26.2219	26.223	26.2828	26.4001	26.1603	4.84458	0.000101725	P32261	Antithrombin-III OS=Mus musculus OX=10090 GN=Serpin	Serpinc1		-0.82	-0.79	CTRL_9dpi;CTRL_7dpi	DD
26.0402	25.892	25.9223	25.8867	24.3981	24.7539	24.3284	24.0999	24.2578	24.3973	24.1647	24.4299	6.26997	9.13E-06	P33622	Apolipoprotein C-III OS=Mus musculus OX=10090 GN=Ap	Apoc3		-1.57	-1.65	CTRL_9dpi;CTRL_7dpi	DD
28.609	28.7516	29.3405	28.4331	27.1588	27.3002	26.8141	27.9437	28.11	27.8892	27.3315	28.0885	2.67178	0.00613254	P33622	Apolipoprotein C-III OS=Mus musculus OX=10090 GN=Ap	Apoc3		-1.65	-1.12	CTRL_7dpi;CTRL_9dpi	DTT
26.2407	26.2718	26.0267	26.4313	24.6938	25.4292	24.5584	24.4538	24.8647	24.844	24.5257	24.575	4.27083	0.00028511	P33622	Apolipoprotein C-III OS=Mus musculus OX=10090 GN=Ap	Apoc3		-1.65	-1.64	CTRL_9dpi;CTRL_7dpi	TCA
23.545	22.8796	22.6951	22.5487	22.0184	22.4727	22.1839	21.7019	21.4816	22.2294	21.8257	21.577	2.30671	0.0124748	P34928	Apolipoprotein C-I OS=Mus musculus OX=10090 GN=Apo	Apoc1		-0.37	-0.77	CTRL_9dpi;CTRL_7dpi	DD
23.7309	23.6264	23.9232	23.0612	21.9844	19.0036	21.0142	20.9287	21.89	22.0607	21.9164	20.8896	2.62539	0.00671283	P34928	Apolipoprotein C-I OS=Mus musculus OX=10090 GN=Apo	Apoc1		-2.54	-1.75	CTRL_7dpi;CTRL_9dpi	DTT
15.3069	15.2983	15.0788	14.899	13.8928	NaN	NaN	12.7951	NaN	NaN	NaN	NaN	2.09878	0.018631	P35917	Vascular endothelial growth factor receptor 3 OS=Mus m	Flt4		-1.76	-6.64	CTRL_7dpi	DD
20.7165	20.9338	20.8874	21.0144	20.0685	20.2183	20.0328	19.5002	19.5034	19.3125	19.6752	18.9833	4.23842	0.000302668	P39039	Mannose-binding protein A OS=Mus musculus OX=1009(	Mbl1		-0.97	-1.33	7dpi_9dpi;CTRL_9dpi;CTRL_7dpi	DD
21.9636	22.0673	22.1649	21.9767	20.9064	21.5199	21.2153	21.0406	20.7266	20.8325	20.7198	19.9643	3.85084	0.00064224	P39039	Mannose-binding protein A OS=Mus musculus OX=1009(	Mbl1		-0.73	-1.47	7dpi_9dpi;CTRL_9dpi;CTRL_7dpi	TCA
17.9206	NaN	18.0501	16.8508	14.778	NaN	NaN	14.9431	NaN	16.7705	16.5845	15.9817	2.31382	0.0123499	P39876	Metalloproteinase inhibitor 3 OS=Mus musculus OX=100	Timp3		-3.05	-1.28	9dpi_CTRL;CTRL_7dpi	TCA
13.2522	NaN	12.4894	NaN	15.8844	16.0659	16.2926	16.1577	14.5593	14.4604	13.7018	14.001	4.48404	0.00019378	P40142	Transketolase OS=Mus musculus OX=10090 GN=Tkt PE=1	Tkt		2.62	1.26	9dpi_CTRL;7dpi_CTRL;7dpi_9dpi	DD
NaN	NaN	13.8487	NaN	18.4377	19.2745	19.1287	18.2497	16.1519	17.6454	16.3355	17.8215	1.9227	0.0265216	P40142	Transketolase OS=Mus musculus OX=10090 GN=Tkt PE=1	Tkt		1.68	0.18	CTRL_7dpi	TCA
21.6958	21.5207	21.642	21.6247	20.6369	20.4814	19.9543	20.5635	20.1795	20.2626	19.5458	18.5216	3.6059	0.00104046	P41317	Mannose-binding protein C OS=Mus musculus OX=10090	Mbl2		-1.64	-2.12	7dpi_9dpi;CTRL_9dpi;CTRL_7dpi	DD
22.0515	22.1993	22.251	22.2377	19.6726	20.0344	18.19	20.1757	19.9681	20.0754	20.2562	19.4823	3.73995	0.000792791	P41317	Mannose-binding protein C OS=Mus musculus OX=10090	Mbl2		-1.77	-2.11	CTRL_7dpi;CTRL_9dpi	TCA
23.1427	23.2229	22.9492	23.2051	20.5661	21.0386	20.7291	20.3166	19.5095	19.6041	19.2603	19.2035	8.23428	4.06E-07	P42703	Leukemia inhibitory factor receptor OS=Mus musculus O:	Lifr		-2.05	-3.61	7dpi_9dpi;CTRL_9dpi;CTRL_7dpi	DD
19.5279	20.1145	20.201	19.3872	14.9718	14.5065	14.7148	14.4	NaN	15.5744	NaN	NaN	6.17308	9.96E-06	P42703	Leukemia inhibitory factor receptor OS=Mus musculus O:	Lifr		-2.72	-1.97	CTRL_7dpi	DTT
23.5159	24.0457	23.9913	24.2128	21.2484	20.6504	20.2538	21.3005	19.898	19.7588	19.4399	19.5413	6.93856	3.65E-06	P42703	Leukemia inhibitory factor receptor OS=Mus musculus O:	Lifr		-2.63	-4.01	7dpi_9dpi;CTRL_9dpi;CTRL_7dpi	TCA
22.5064	22.7066	22.2717	22.1813	22.5749	23.2362	22.9096	22.895	22.7459	22.9769	22.7278	23.1081	1.59137	0.0498857	P46412	Glutathione peroxidase 3 OS=Mus musculus OX=10090 G	Gpx3		0.45	0.4	9dpi_CTRL;7dpi_CTRL	DD
22.7339	23.2869	23.0239	23.2125	23.6436	23.3747	23.5716	23.7558	23.7097	24.0781	23.4539	23.4157	1.85293	0.0299974	P46412	Glutathione peroxidase 3 OS=Mus musculus OX=10090 G	Gpx3		0.41	0.7	7dpi_CTRL;9dpi_CTRL	TCA
16.8982	17.0586	16.8321	16.9651	NaN	NaN	NaN	16.1992	15.5827	NaN	15.1924	NaN	3.4251	0.00151388	P47878	Insulin-like growth factor-binding protein 3 OS=Mus mus	Igfbp3		-0.73	-1.56	CTRL_7dpi	DD
18.4732	19.3405	19.0353	18.9395	17.0915	16.8025	17.973	18.0235	16.4681	17.0124	15.6978	15.6078	3.49878	0.00131567	P49182	Heparin cofactor 2 OS=Mus musculus OX=10090 GN=Ser	Serpind1		-1.04	-2.02	CTRL_9dpi;CTRL_7dpi	DTT
21.2208	21.4379	21.1343	21.2966	20.8722	20.3539	20.789	20.7741	20.0406	19.7304	19.8046	20.502	3.64844	0.000960736	P51885	Lumican OS=Mus musculus OX=10090 GN=Lum PE=1 SV=	Lum		-0.76	-1.35	7dpi_9dpi;CTRL_9dpi;CTRL_7dpi	DD
19.6578	19.1075	21.5902	19.0721	18.2413	18.0439	15.1901	16.6911	14.9502	14.8589	15.1975	17.8515	2.26528	0.0135628	P51885	Lumican OS=Mus musculus OX=10090 GN=Lum PE=1 SV=	Lum		-0.79	-2.43	CTRL_9dpi;CTRL_7dpi	DTT
21.5879	22.0488	21.7631	22.1118	23.0162	22.9348	22.7478	22.7493	23.5923	23.6184	23.8024	23.6459	6.32088	8.54E-06	P51910	Apolipoprotein D OS=Mus musculus OX=10090 GN=Apod	Apod		1.01	1.67	7dpi_CTRL;9dpi_CTRL;9dpi_7dpi	DD
23.4598	23.5561	23.3614	23.5355	23.9686	24.2094	23.9942	23.9985	24.7939	24.8882	25.0482	24.6662	6.54018	5.91E-06	P51910	Apolipoprotein D OS=Mus musculus OX=10090 GN=Apod	Apod		0.65	1.37	7dpi_CTRL;9dpi_CTRL;9dpi_7dpi	TCA

23.5731	23.7391	23.546	23.742	22.3837	22.6001	22.34	22.0602	22.4325	22.8278	23.3584	22.3043	3.22209	0.00218738	P52430	Serum paraoxonase/arylesterase 1 OS=Mus musculus OX	Pon1	-1.2	-0.88	CTRL_7dpi;CTRL_9dpi	DD
24.6761	24.6896	24.7654	24.8061	23.2551	22.6001	22.5263	22.9283	23.4852	23.9334	24.4497	22.954	3.38524	0.00160775	P52430	Serum paraoxonase/arylesterase 1 OS=Mus musculus OX	Pon1	-1.79	-0.93	9dpi_7dpi;CTRL_7dpi;CTRL_9dpi	TCA
17.2755	17.0749	17.4434	17.7703	19.4657	19.9215	20.1125	19.8557	18.9268	18.876	18.8707	19.2146	6.1234	1.05E-05	P52480	Pyruvate kinase PKM OS=Mus musculus OX=10090 GN=P	Pkm	2.34	1.37	9dpi_CTRL;7dpi_CTRL;7dpi_9dpi	DD
18.0013	17.3075	18.1995	19.326	21.4222	21.9037	21.637	21.007	20.8525	20.9955	20.5314	20.9113	4.51244	0.000183067	P52480	Pyruvate kinase PKM OS=Mus musculus OX=10090 GN=P	Pkm	2.09	1.83	9dpi_CTRL;7dpi_CTRL	TCA
22.6176	21.7856	21.7684	22.37	25.8725	26.3049	25.7994	26.1567	25.5543	25.7585	25.048	26.0698	6.63913	5.16E-06	P60710	Actin, cytoplasmic 1 OS=Mus musculus OX=10090 GN=Ac	Actb	3.62	3.07	9dpi_CTRL;7dpi_CTRL	DD
21.5655	21.3719	21.7632	22.0223	24.8512	24.8687	25.5791	24.1953	23.591	23.5759	23.495	22.7853	5.07954	6.36E-05	P60710	Actin, cytoplasmic 1 OS=Mus musculus OX=10090 GN=Ac	Actb	3.45	2.01	9dpi_CTRL;7dpi_CTRL;7dpi_9dpi	DTT
23.6008	22.4767	22.7606	23.4098	26.5535	27.112	27.0688	26.4585	26.3414	26.5614	25.9497	26.5195	6.23729	9.39E-06	P60710	Actin, cytoplasmic 1 OS=Mus musculus OX=10090 GN=Ac	Actb	3.64	3.17	9dpi_CTRL;7dpi_CTRL	TCA
16.8245	18.3682	18.4634	18.1021	15.3825	NaN	NaN	16.2136	13.8103	13.8491	14.567	15.1151	3.34963	0.00171212	P61110	Kidney androgen-regulated protein OS=Mus musculus O)	Kap	-2.2	-3.66	CTRL_9dpi;CTRL_7dpi	TCA
16.5103	15.9169	15.0299	17.4563	19.3511	20.028	19.2133	19.8553	19.1301	19.5084	18.5016	18.8474	3.88419	0.00060265	P62897	Cytochrome c, somatic OS=Mus musculus OX=10090 GN=	Cycs	2.32	2.01	9dpi_CTRL;7dpi_CTRL	DD
16.4561	16.2644	16.7733	16.238	18.3491	17.1157	16.7412	18.3677	17.9929	18.4479	17.8692	18.4445	2.45548	0.00950219	P62897	Cytochrome c, somatic OS=Mus musculus OX=10090 GN=	Cycs	1.17	1.57	7dpi_CTRL;9dpi_CTRL	TCA
18.8968	16.1957	17.0499	17.9831	21.7213	22.3589	22.3036	21.8076	21.7832	22.027	20.8448	22.1939	4.54069	0.000174519	P63017	Heat shock cognate 71 kDa protein OS=Mus musculus O)	Hspa8	3.36	2.98	9dpi_CTRL;7dpi_CTRL	DD
19.8847	15.4283	18.7843	18.8478	22.5032	22.9666	22.9136	22.7	22.7342	22.7067	21.8291	22.8381	3.26348	0.00201046	P63017	Heat shock cognate 71 kDa protein OS=Mus musculus O)	Hspa8	3.87	3.37	9dpi_CTRL;7dpi_CTRL	TCA
16.4529	14.8329	15.8668	16.3981	19.4459	19.9098	19.4008	19.9708	19.1217	19.352	18.6324	19.5142	5.29921	4.27E-05	P63101	14-3-3 protein zeta/delta OS=Mus musculus OX=10090 G	Ywhaz	3.17	2.6	9dpi_CTRL;7dpi_CTRL	DD
15.8325	14.9384	15.635	16.5809	17.1423	16.671	17.5291	17.0819	17.4116	17.939	16.6116	17.6169	2.33114	0.0119105	P63101	14-3-3 protein zeta/delta OS=Mus musculus OX=10090 G	Ywhaz	1.38	1.73	7dpi_CTRL;9dpi_CTRL	TCA
14.8195	16.905	NaN	17.4264	22.3368	23.3172	22.9034	23.1153	23.3382	22.6108	21.9213	22.718	5.14302	5.70E-05	P68134	Actin, alpha skeletal muscle OS=Mus musculus OX=1009(	Acta1	6.1	5.57	9dpi_CTRL;7dpi_CTRL	DD
18.2359	17.6805	18.1503	18.7503	21.2612	20.8218	21.3582	21.3502	20.5458	20.8273	21.0214	20.4584	6.06359	1.16E-05	P68134	Actin, alpha skeletal muscle OS=Mus musculus OX=1009(	Acta1	2.83	2.49	9dpi_CTRL;7dpi_CTRL	DTT
20.3104	19.6311	19.6818	20.8324	23.3934	22.9775	23.4768	23.6698	23.0506	23.3943	22.5973	23.4882	5.46584	3.38E-05	P68134	Actin, alpha skeletal muscle OS=Mus musculus OX=1009(	Acta1	2.96	2.86	9dpi_CTRL;7dpi_CTRL	TCA
21.0327	20.9275	20.9372	21.3096	20.7894	21.2228	21.1871	21.3624	20.5041	20.7167	20.8455	20.18	1.76391	0.0355143	P70274	Selenoprotein P OS=Mus musculus OX=10090 GN=Selenc	Selenop	0.19	-0.57	CTRL_9dpi;7dpi_9dpi	DD
21.4205	21.0634	20.9709	21.0389	17.5007	18.2908	17.3966	17.0036	18.1845	17.3493	18.3755	16.9439	5.25135	4.59E-05	P70389	Insulin-like growth factor-binding protein complex acid la	Igfals	-2.77	-2.78	CTRL_7dpi;CTRL_9dpi	DD
21.6326	21.4438	21.3866	21.268	18.365	17.9547	16.3501	18.3158	18.8338	18.4625	19.3726	16.726	3.39108	0.00160775	P70389	Insulin-like growth factor-binding protein complex acid la	Igfals	-2.59	-2.35	CTRL_7dpi;CTRL_9dpi	TCA
17.8146	18.2089	18.0853	18.4602	19.1061	19.8388	18.9821	20.1812	19.8488	20.3832	19.3414	18.8583	2.25194	0.0138863	P70663	SPARC-like protein 1 OS=Mus musculus OX=10090 GN=S|	Sparcl1	1.42	1.45	7dpi_CTRL;9dpi_CTRL	DD
15.7283	16.0155	15.5074	15.295	17.0294	17.3047	16.4767	16.8695	17.4617	17.6541	18.3707	16.9823	3.47592	0.00137046	P82198	Transforming growth factor-beta-induced protein ig-h3 C	Tgfbi	1.3	1.81	7dpi_CTRL;9dpi_CTRL	TCA
24.2438	24.4199	24.0455	24.3043	25.6855	25.939	25.7469	25.7742	25.3356	25.5816	24.985	25.1084	5.45679	3.38E-05	P97290	Plasma protease C1 inhibitor OS=Mus musculus OX=1009	Serping1	1.59	1	9dpi_CTRL;7dpi_CTRL;7dpi_9dpi	DD
25.1027	25.2043	25.1597	24.9844	26.5699	25.6124	25.8316	26.4062	26.2073	26.3236	26.093	25.9572	3.05464	0.00291278	P97290	Plasma protease C1 inhibitor OS=Mus musculus OX=1009	Serping1	1.01	1.04	7dpi_CTRL;9dpi_CTRL	TCA
17.3624	17.4859	17.4632	17.5272	17.3156	16.9536	16.3872	17.6168	16.1469	16.4038	16.1342	NaN	2.38224	0.010786	P97298	Pigment epithelium-derived factor OS=Mus musculus OX	Serpinf1	-0.31	-1.27	7dpi_9dpi;CTRL_9dpi	DD
15.6802	16.0296	16.0937	15.5045	13.5392	14.7849	13.419	NaN	NaN	13.6824	14.2867	NaN	2.34499	0.0115791	P97298	Pigment epithelium-derived factor OS=Mus musculus OX	Serpinf1	-2.11	-1.82	CTRL_7dpi;CTRL_9dpi	DTT
21.2472	21.3898	20.9032	20.4598	18.7667	19.4395	18.8089	19.0536	19.3853	19.5788	20.3513	19.247	3.6213	0.0010103	P98086	Complement C1q subcomponent subunit A OS=Mus mus	C1qa	-1.51	-1.43	CTRL_7dpi;CTRL_9dpi	DD
20.2033	20.5951	20.7778	20.7855	19.2868	18.4153	18.3206	18.7954	18.9067	18.5546	19.8102	18.0932	3.0275	0.00304294	P98086	Complement C1q subcomponent subunit A OS=Mus mus	C1qa	-1.59	-1.5	CTRL_7dpi;CTRL_9dpi	TCA
15.2726	NaN	15.4575	NaN	17.8418	16.4775	16.3317	17.3722	18.4403	18.1322	17.6659	17.9506	2.74248	0.00541248	P99026	Proteasome subunit beta type-4 OS=Mus musculus OX=1	Psmb4	1.56	2.68	7dpi_CTRL;9dpi_CTRL	TCA
30.3916	30.4645	30.4648	30.3573	30.2196	29.9935	30.0578	29.9012	30.4358	30.4329	30.3131	30.1044	2.30629	0.0124748	Q00623	Apolipoprotein A-I OS=Mus musculus OX=10090 GN=Apo	Apoa1	-0.46	-0.01	9dpi_7dpi;CTRL_7dpi	DD
29.4952	29.6443	29.5097	29.755	29.2931	28.8789	28.7637	28.8234	29.6315	29.6174	28.9967	29.346	2.0768	0.0194678	Q00623	Apolipoprotein A-I OS=Mus musculus OX=10090 GN=Apo	Apoa1	-0.79	-0.61	CTRL_7dpi	TCA
23.6828	22.9569	23.157	22.9906	21.9947	21.4162	21.7169	22.2425	21.6872	21.5806	21.792	21.7649	4.13286	0.00037196	Q00724	Retinol-binding protein 4 OS=Mus musculus OX=10090 G	Rbp4	-1.38	-1.45	CTRL_9dpi;CTRL_7dpi	DD
19.9318	19.2326	19.6352	19.5586	16.7759	16.7502	17.3215	17.6854	17.3547	17.0097	15.8976	16.61	4.58733	0.000158125	Q00896	Alpha-1-antitrypsin 1-3 OS=Mus musculus OX=10090 GN:	Serpina1c	-2.47	-2.56	CTRL_9dpi;CTRL_7dpi	DD
24.6626	24.604	24.3151	25.033	24.9091	25.9633	25.3056	24.3144	23.7402	22.9727	23.9024	23.0965	2.51381	0.00837311	Q00896	Alpha-1-antitrypsin 1-3 OS=Mus musculus OX=10090 GN:	Serpina1c	0.39	-1.09	CTRL_9dpi;7dpi_9dpi	DTT
23.2831	23.1971	23.0862	23.1179	23.9907	24.3595	24.7706	24.0791	23.7039	23.7343	23.648	23.7585	3.90707	0.000577766	Q00897	Alpha-1-antitrypsin 1-4 OS=Mus musculus OX=10090 GN:	Serpina1d	1.41	0.83	9dpi_CTRL;7dpi_CTRL;7dpi_9dpi	DD
22.9161	21.8524	22.0724	21.6964	22.7953	23.5953	22.4303	23.8208	24.0944	23.5941	23.6088	24.3791	2.41885	0.0101799	Q00897	Alpha-1-antitrypsin 1-4 OS=Mus musculus OX=10090 GN:	Serpina1d	0.74	2.12	9dpi_CTRL	DTT
24.5804	24.6755	24.7527	24.7761	23.9474	24.3006	24.2964	24.0241	24.0794	23.7747	23.7083	23.6107	3.99326	0.000502057	Q01279	Epidermal growth factor receptor OS=Mus musculus OX=	Egfr	-0.51	-0.89	7dpi_9dpi;CTRL_9dpi;CTRL_7dpi	DD
24.4264	24.692	24.716	24.9328	24.0685	24.5148	24.3135	24.1695	24.1232	23.8636	23.8954	23.6372	3.00908	0.0031601	Q01279	Epidermal growth factor receptor OS=Mus musculus OX=	Egfr	-0.52	-0.82	CTRL_9dpi;CTRL_7dpi	TCA
26.4819	26.5593	26.5661	26.6487	27.0645	27.0196	27.2127	27.046	27.176	27.2514	27.0488	27.2014	5.37414	3.82E-05	Q01339	Beta-2-glycoprotein 1 OS=Mus musculus OX=10090 GN=/	Apoh	0.46	0.56	7dpi_CTRL;9dpi_CTRL	DD
16.6523	NaN	15.8402	NaN	19.667	20.0699	20.0097	19.5661	19.1604	19.2367	18.9185	19.7889	4.60123	0.000154497	Q01768	Nucleoside diphosphate kinase B OS=Mus musculus OX=	Nme2	2.73	2.44	9dpi_CTRL;7dpi_CTRL	DD
17.0276	NaN	16.0026	16.5039	20.1345	19.7728	19.6912	19.3623	19.5874	19.7771	19.8048	20.151	4.6183	0.000151219	Q01768	Nucleoside diphosphate kinase B OS=Mus musculus OX=	Nme2	3.23	2.76	9dpi_CTRL;7dpi_CTRL	TCA
19.7238	20.755	20.1131	19.5193	19.1645	19.1232	18.2366	19.0563	18.9572	18.7427	19.6379	18.7287	1.81591	0.0323689	Q02105	Complement C1q subcomponent subunit C OS=Mus mus	C1qc	-1.33	-0.78	CTRL_7dpi;CTRL_9dpi	DD
21.4897	21.6859	21.8003	21.0342	19.5623	18.0777	17.9472	19.4849	19.7717	19.9087	20.7351	19.2184	2.92659	0.00370117	Q02105	Complement C1q subcomponent subunit C OS=Mus mus	C1qc	-2.58	-1.45	CTRL_7dpi;CTRL_9dpi	TCA
19.2866	20.0135	19.6947	20.2274	21.4687	21.8891	21.3721	21.3856	21.4401	21.3649	21.2157	21.452	4.70657	0.000126836	Q03734	Serine protease inhibitor A3M OS=Mus musculus OX=10(	Serpina3m	1.65	1.53	9dpi_CTRL;7dpi_CTRL	DD
20.972	21.0743	21.0196	20.739	22.9791	22.2672	22.4696	22.8067	22.6184	22.4071	22.7403	22.542	5.62512	2.54E-05	Q03734	Serine protease inhibitor A3M OS=Mus musculus OX=10(	Serpina3m	1.71	1.48	9dpi_CTRL;7dpi_CTRL	TCA
22.561	22.0682	22.181	21.9034	18.8271	18.0846	19.5125	19.2461	20.4664	19.3747	20.523	19.3402	4.35343	0.00024515	Q05020	Apolipoprotein C-II OS=Mus musculus OX=10090 GN=Ap(	Apoc2	-3.21	-2.19	CTRL_7dpi;CTRL_9dpi	DD
25.6308	25.4121	26.047	24.832	23.6549	23.7181	22.3177	24.0505	24.2452	24.1418	23.6573	24.7986	2.54624	0.00786303	Q05020	Apolipoprotein C-II OS=Mus musculus OX=10090 GN=Ap(	Apoc2	-1.63	-1.11	CTRL_7dpi;CTRL_9dpi	DTT
22.0114	22.1071	22.3318	22.1437	19.4799	18.6368	18.8086	18.8304	20.1455	20.0153	19.3282	19.2246	5.95469	1.38E-05	Q05020	Apolipoprotein C-II OS=Mus musculus OX=10090 GN=Ap(	Apoc2	-3.34	-2.78	7dpi_9dpi;CTRL_7dpi;CTRL_9dpi	TCA
NaN	NaN	16.8842	NaN	18.2483	18.5484	19.0048	19.9727	16.3646	NaN	16.3624	17.2936	2.1545	0.0165544	Q06318	Uteroglobin OS=Mus musculus OX=10090 GN=Scgb1a1 P	Scgb1a1	1.89	-0.52	CTRL_7dpi	DD
23.6765	23.8176	23.5826	23.8858	22.5895	22.495	22.6367	22.4471	20.8265	22.4639	22.4162	21.2403	3.06324	0.00286931	Q06770	Corticosteroid-binding globulin OS=Mus musculus OX=10	Serpina6	-0.81	-2.4	CTRL_9dpi;CTRL_7dpi	DD
24.2063	24.5969	24.6666	24.3149	23.3447	17.7045	20.5661	23.1622	22.63	22.4336	23.0885	21.9741	1.75988	0.0357402	Q06770	Corticosteroid-binding globulin OS=Mus musculus OX=10	Serpina6	-1.47	-2.36	CTRL_7dpi	TCA
25.5506	25.6877	25.5071	25.5954	26.5374	26.6529	26.6071	26.5258	26.3283	26.1994	25.8374	26.1371	5.09324	6.32E-05	Q06890	Clusterin OS=Mus musculus OX=10090 GN=Clu PE=1 SV=	Clu	1.19	0.64	9dpi_CTRL;7dpi_CTRL;7dpi_9dpi	DD
25.416	25.1391	24.2252	25.0617	26.2252	26.5411	25.8604	26.746	26.2716	25.9694	25.1402	25.9844	2.15181	0.0166008	Q06890	Clusterin OS=Mus musculus OX=10090 GN=Clu PE=1 SV=	Clu	0.89	0.5	7dpi_CTRL	DTT
26.3269	26.7832	26.8279	26.9213	26.9867	27.5022	27.2361	27.1853	26.6914	26.5119	26.3859	26.7624	2.22122	0.0145419	Q06890	Clusterin OS=Mus musculus OX=10090 GN=Clu PE=1 SV=	Clu	0.73	0.17	7dpi_9dpi;7dpi_CTRL	TCA
24.0868	24.4099	24.2038	24.1231	25.5857	25.6999	25.6596	25.632	25.9622	25.9645	25.8593	25.8432	8.6256	2.75E-07	Q07456	Protein AMBP OS=Mus musculus OX=10090 GN=Ambp PI	Ambp	1.44	1.63	7dpi_CTRL;9dpi_CTRL;9dpi_7dpi	DD
24.2338	24.391	23.9905	24.1419	25.5339	25.9953	25.9897	25.3994	26.0441	26.1505	25.8948	25.8045	5.64614	2.50E-05	Q07456	Protein AMBP OS=Mus musculus OX=10090 GN=Ambp PI	Ambp	1.68	1.78	7dpi_CTRL;9dpi_CTRL	TCA
20.8778	21.0847	21.083	21.075	19.3603	19.464	19.5963	19.5105	19.5994	19.6712	19.8067	18.872	4.83097	0.000103904	Q07968	Coagulation factor XIII B chain OS=Mus musculus OX=10(	F13b	-1.45	-1.45	CTRL_7dpi;CTRL_9dpi	TCA
18.0848	18.9373	19.2825	19.0968	16.6062	17.3079	17.2878	17.2035	14.9734	15.9022	17.4164	15.5815	3.07361	0.00282853	Q08879	Fibulin-1 OS=Mus musculus OX=10090 GN=Fbln1 PE=1 S\	Fbln1	-1.16	-1.85	CTRL_9dpi;CTRL_7dpi	DD
20.1459	20.3162	20.3208	20.6555	18.8829	19.0906	18.8003	18.8998	17.9511	18.215	18.2635	17.8715	7.07475	2.87E-06	Q08879	Fibulin-1 OS=Mus musculus OX=10090 GN=Fbln1 PE=1 S\	Fbln1	-1.51	-2.24	7dpi_9dpi;CTRL_9dpi;CTRL_7dpi	TCA
14.8477	15.4975	15.3571	15.1821	16.8101	16.5589	15.7361	16.1638	16.0907	16.1115	15.8402	15.7595	2.45336	0.00951165	Q3SXB8	Collectin-11 OS=Mus musculus OX=10090 GN=Colec11 PI	Colec11	1.13	0.7	9dpi_CTRL;7dpi_CTRL	TCA
15.0034	14.9945	15.1583	14.5737	19.1846	15.9541	19.0031	20.3099	19.9758	19.7894	19.7554	20.7419	3.49394	0.00132254	Q3TTY5	Keratin, type II cytoskeletal 2 epidermal OS=Mus muscul	Krt2	3.38	4.19	7dpi_CTRL;9dpi_CTRL	DTT
16.9963	15.2559	16.7025	16.8869	19.0631	19.0973	NaN	NaN	20.0994	20.0704	19.1251	19.0704	3.20497	0.0022527	Q3UV17	Keratin, type II cytoskeletal 2 oral OS=Mus musculus OX=	Krt76	2.29	3.14	7dpi_CTRL;9dpi_CTRL	DTT
17.3395	16.9453	17.0968	17.1508	14.0627	14.4561	NaN	13.7417	13.9748	13.8937	14.6797	13.094	4.90196	9.10E-05	Q3V2T4	Keratinocyte differentiation-associated protein OS=Mus r	Krtdap	-3.06	-3.18	CTRL_9dpi;CTRL_7dpi	DD
21.0652	21.1267	22.2724	20.685	18.3114	18.4678	17.0478	17.6553	17.5498	16.4681	18.4353	16.5963	4.11711	0.00038292	Q3V2T4	Keratinocyte differentiation-associated protein OS=Mus r	Krtdap	-3.41	-4.11	CTRL_9dpi;CTRL_7dpi	DTT
24.62	24.3667	24.1337	24.4033	26.7872	26.9492	26.5873	27.076	26.8678	26.593	26.9362	26.951	7.61031	1.22E-06	Q60590	Alpha-1-acid glycoprotein 1 OS=Mus musculus OX=1009(	Orm1	2.6	2.38	9dpi_CTRL;7dpi_CTRL	DD
21.2342	21.0213	23.1849	21.2354	24.7127	24.3829	22.8443	22.861	23.0355	23.1283	23.8114	24.0644	1.77698	0.0348744	Q60590	Alpha-1-acid glycoprotein 1 OS=Mus musculus OX=1009(	Orm1	2.43	1.57	9dpi_CTRL;7dpi_CTRL	DTT
24.1526	24.4165	24.4837	24.5597	27.2604	26.6749	27.1421	27.3691	27.1223	26.4087	26.9774	26.502	6.23702	9.39E-06	Q60590	Alpha-1-acid glycoprotein 1 OS=Mus musculus OX=1009(	Orm1	2.57	2.47	9dpi_CTRL;7dpi_CTRL	TCA
16.1751	16.2336	15.7768	15.7649	17.3915	17.9179	17.1553	17.1157	17.8987	17.704	17.7486	18.0016	4.92101	8.80E-05	Q60692	Proteasome subunit beta type-6 OS=Mus musculus OX=1	Psmb6	1.36	1.88	7dpi_CTRL;9dpi_CTRL	TCA
19.2056	19.3938	19.4759	19.3959	21.8832	21.7928	21.8756	21.8682	22.5397	22.485	21.9991	22.5047	8.72174	2.75E-07	Q60963	Platelet-activating factor acetylhydrolase OS=Mus muscu	Pla2g7	2.34	2.56	7dpi_CTRL;9dpi_CTRL;9dpi_7dpi	DD
16.0227	12.8568	15.376	12.7751	19.2029	18.5614	20.5591	18.8529	19.8538	18.2655	19.5411	17.3974	3.13897	0.00255621	Q60963	Platelet-activating factor acetylhydrolase OS=Mus muscu	Pla2g7	3.12	2.41	9dpi_CTRL;7dpi_CTRL	DTT
15.9571	19.9349	19.7113	19.637	22.219	21.882	21.9558	22.6184	23.3217	23.125	22.8099	23.1895	6.69205	4.88E-06	Q60963	Platelet-activating factor acetylhydrolase OS=Mus muscu	Pla2g7	2.67	3.17	7dpi_CTRL;9dpi_CTRL;9dpi_7dpi	TCA
20.6077	21.0415	21.1954	21.2227	20.6822	18.3163	18.3729	19.8314	19.8424	20.5225	20.1795	19.6451	1.5916	0.0498857	Q60994	Adiponectin OS=Mus musculus OX=10090 GN=Adipoq PE	Adipoq	-1.55	-0.78	CTRL_7dpi	TCA
26.9352	27.0014	26.92	26.8589	28.7538	28.9245	28.8914	28.9932	29.1026	29.0047	29.0453	29.0609	10.7619	1.21E-08	Q61147	Ceruloplasmin OS=Mus musculus OX=10090 GN=Cp PE=1	Cp	2.11	2.17	7dpi_CTRL;9dpi_CTRL;9dpi_7dpi	DD
27.2876	27.4721	27.4187	27.498	29.1198	29.3551	29.5569	29.1863	29.2869	29.257	29.3137	29.2022	8.1255	4.75E-07	Q61147	Ceruloplasmin OS=Mus musculus OX=10090 GN=Cp PE=1	Cp	2.1	2.12	9dpi_CTRL;7dpi_CTRL	TCA
23.8154	23.1018	24.0897	22.6961	21.812	21.3407	20.8718	22.5969	21.8317	22.7707	22.0431	23.021	1.90388	0.0273581	Q61207	Prosaposin OS=Mus musculus OX=10090 GN=Psap PE=1	Psap	0.83	1.44	CTRL_7dpi	DTT
NaN	NaN	NaN	NaN	18.6606	18.8553	18.6374	18.7584	18.0904	18.3071	17.8813	18.1625	3.04276	0.00297942	Q61233	Plastin-2 OS=Mus musculus OX=10090 GN=Lcp1 PE=1 SV	Lcp1	6.64	6.64	CTRL_7dpi	DD
17.0359	17.6643	16.6786	17.1052	19.2038	19.6051	19.3495	19.6315	18.9331	18.009	18.4631	17.8989	4.36945	0.000240082	Q61233	Plastin-2 OS=Mus musculus OX=10090 GN=Lcp1 PE=1 SV	Lcp1	1.79	0.39	9dpi_CTRL;7dpi_CTRL;7dpi_9dpi	TCA
25.9329	26.1775	25.7771	25.6954	25.3745	24.9667	25.3452	25.1395	25.3363	25.4894	25.2706	24.9579	2.69447	0.00589338	Q61247	Alpha-2-antiplasmin OS=Mus musculus OX=10090 GN=Se	Serpinf2	-0.81	-0.68	CTRL_7dpi;CTRL_9dpi	TCA
22.744	22.592	22.5329	22.3907	21.1358	21.4612	21.1092	20.3081	21.4605	21.758	20.751	20.8827	3.11284	0.00267423	Q61268	Apolipoprotein C-IV OS=Mus musculus OX=10090 GN=Ap	Apoc4	-1.4	-1.4	CTRL_7dpi;CTRL_9dpi	DD
21.8727	21.6421	21.1384	21.4083	19.7289	20.4062	19.465	19.9891	19.0446	19.2054	18.2985	19.7954	3.90539	0.000577766	Q61268	Apolipoprotein C-IV OS=Mus musculus OX=10090 GN=Ap	Apoc4	-1.64	-1.27	CTRL_9dpi;CTRL_7dpi	DTT
21.1954	21.0736	20.6847	20.6878	19.2913	19.7959	18.3564	19.3338	20.0533	20.228	18.8099	19.2211	2.41071	0.0102936	Q61268	Apolipoprotein C-IV OS=Mus musculus OX=10090 GN=Ap	Apoc4	-1.54	-1.28	CTRL_7dpi;CTRL_9dpi	TCA
13.1496	14.0481	12.9574	13.9981	15.323	14.9561	15.8249	15.8392	14.1108	14.5877	15.001	12.4851	1.85565	0.029966	Q61508	Extracellular matrix protein 1 OS=Mus musculus OX=1009	Ecm1	1.83	0.77	7dpi_CTRL	DD
24.5455	24.3528	23.133	24.1636	20.796	21.6041	21.1903	21.3529	20.6447	20.9098	19.9808	20.6326	5.26098	4.55E-05	Q61646	Haptoglobin OS=Mus musculus OX=10090 GN=Hp PE=1 S	Hp	-2.73	-3.35	CTRL_9dpi;CTRL_7dpi	DD
24.5846	23.5685	22.9421	23.1702	19.1735	18.3014	20.7642	19.7921	18.8673	19.1385	18.1211	18.2095	4.70778	0.000126836	Q61646	Haptoglobin OS=Mus musculus OX=10090 GN=Hp PE=1 S	Hp	-2.82	-3.43	CTRL_9dpi;CTRL_7dpi	DTT
26.1637	26.3499	25.0961	25.7453	22.3385	22.4134	22.5439	22.3711	22.2708	22.4255	21.5026	22.0574	6.30228	8.69E-06	Q61646	Haptoglobin OS=Mus musculus OX=10090 GN=Hp PE=1 S	Hp	-3.4	-3.8	CTRL_9dpi;CTRL_7dpi	TCA
24.1399	24.1978	24.1309	24.2305	23.6207	24.0889	23.8435	23.8308	23.7945	24.0383	24.0286	23.7972	1.70154	0.0397066	Q61702	Inter-alpha-trypsin inhibitor heavy chain H1 OS=Mus mus	Itih1	-0.35	-0.66	CTRL_7dpi	DD
21.0016	21.771	21.85	21.9306	19.3211	19.6823	19.0341	19.38	18.869	20.064	20.686	17.4306	2.2028	0.0150672	Q61702	Inter-alpha-trypsin inhibitor heavy chain H1 OS=Mus mus	Itih1	-1.58	-1.24	CTRL_9dpi;CTRL_7dpi	DTT
23.9851	24.2746	23.5158	23.6696	26.2024	26.3524	26.4315	26.4063	26.6477	26.5732	26.362	26.2456	7.06313	2.87E-06	Q61704	Inter-alpha-trypsin inhibitor heavy chain H3 OS=Mus mus	Itih3	2.49	2.55	7dpi_CTRL;9dpi_CTRL	DD
21.3683	22.9996	21.3746	22.8652	24.1065	23.9992	24.7068	24.0498	23.9358	23.7228	24.4345	23.8619	2.87167	0.00412611	Q61704	Inter-alpha-trypsin inhibitor heavy chain H3 OS=Mus mus	Itih3	2.38	1.88	9dpi_CTRL;7dpi_CTRL	DTT
25.3114	25.6165	25.4232	25.4493	27.2067	27.1222	27.0603	27.128	27.4007	27.279	27.1951	27.1396	8.67181	2.75E-07	Q61704	Inter-alpha-trypsin inhibitor heavy chain H3 OS=Mus mus	Itih3	2.2	2.21	7dpi_CTRL;9dpi_CTRL	TCA
22.0451	22.4122	22.2828	22.5469	20.7053	21.0365	20.4458	20.2065	20.8944	20.61	21.3156	20.926	4.43451	0.00021291	Q61730	Interleukin-1 receptor accessory protein OS=Mus muscul	Il1rap	-0.98	-1.34	CTRL_7dpi;CTRL_9dpi	DD
18.7776	20.1197	19.2699	20.3152	15.3754	NaN	NaN	NaN	14.7381	17.6634	NaN	15.5445	2.11356	0.0180681	Q61730	Interleukin-1 receptor accessory protein OS=Mus muscul	Il1rap	-1.65	-1.64	CTRL_7dpi	DTT
21.7616	21.7783	22.1812	22.5372	20.7654	20.5281	20.895	20.7367	20.2456	20.8453	20.7936	20.1242	3.90868	0.000577766	Q61730	Interleukin-1 receptor accessory protein OS=Mus muscul	Il1rap	-1.64	-1.71	CTRL_9dpi;CTRL_7dpi	TCA
16.8162	17.5332	17.7338	17.5051	20.3219	20.8013	20.5671	20.5136	20.195	20.5871	19.8307	19.6711	6.04568	1.18E-05	Q61805	Lipopolysaccharide-binding protein OS=Mus musculus O)	Lbp	2.54	2.14	9dpi_CTRL;7dpi_CTRL	DD
17.4444	18.4937	17.7973	17.173	21.0429	20.2841	20.6351	21.0257	21.1305	21.3861	20.6192	20.5939	5.31321	4.21E-05	Q61805	Lipopolysaccharide-binding protein OS=Mus musculus O)	Lbp	2.43	2.63	7dpi_CTRL;9dpi_CTRL	TCA

28.1945	28.9809	28.6458	28.9023	27.7008	27.9361	27.6526	27.0614	27.1875	27.6677	28.2415	26.4216	1.80573	0.0328372	Q61838	Pregnancy zone protein OS=Mus musculus OX=10090 GN Pzp	-1.36	-1.39	CTRL_9dpi;CTRL_7dpi	DTT
31.3223	31.5626	31.5962	31.5002	31.2065	30.9733	30.6142	31.1442	31.3032	31.1976	31.1428	31.0467	2.03558	0.0211938	Q61838	Pregnancy zone protein OS=Mus musculus OX=10090 GN Pzp	-0.5	-0.46	CTRL_7dpi	TCA
NaN	17.0518	NaN	16.9421	NaN	15.1056	NaN	14.5303	14.709	NaN	NaN	NaN	1.75485	0.0358885	Q62351	Transferrin receptor protein 1 OS=Mus musculus OX=100 Tfrc	-2.18	-2.29	CTRL_7dpi	DD
20.0864	20.6258	20.3078	20.4846	18.6787	15.0155	15.0082	18.8127	18.5732	18.7218	18.7241	18.8021	1.96354	0.0243745	Q64288	Olfactory marker protein OS=Mus musculus OX=10090 G Omp	-3.51	-1.71	CTRL_7dpi	TCA
23.7301	23.8011	23.6574	23.5485	23.9656	24.2061	24.357	24.2012	24.4191	24.3614	24.4129	24.3285	4.43225	0.00021291	Q64726	Zinc-alpha-2-glycoprotein OS=Mus musculus OX=10090 G Azgp1	0.67	0.7	7dpi_CTRL;9dpi_CTRL	DD
23.9065	23.9884	24.0968	24.3069	24.3608	24.7419	24.9364	24.7244	24.7107	24.5881	24.6052	24.4858	2.73764	0.00544971	Q64726	Zinc-alpha-2-glycoprotein OS=Mus musculus OX=10090 G Azgp1	0.49	0.54	9dpi_CTRL;7dpi_CTRL	TCA
16.9092	17.7382	18.1669	17.3095	14.7844	NaN	13.9533	NaN	16.9318	16.2498	NaN	17.0248	2.93138	0.00367706	Q64739	Collagen alpha-2(XI) chain OS=Mus musculus OX=10090 (Col11a2	-2.44	-0.1	9dpi_7dpi;CTRL_7dpi	DTT
16.1801	19.8462	17.1681	17.2724	15.0041	14.6588	15.2777	15.5443	15.5883	15.3754	16.6343	15.6099	1.80843	0.0328317	Q6NXH9	Keratin, type II cytoskeletal 73 OS=Mus musculus OX=100 Krt73	-2.08	-1.62	CTRL_7dpi	DD
15.7147	15.3696	15.8718	15.7732	16.8735	17.2861	16.9767	16.7783	16.3039	16.4263	17.1323	16.3821	3.43178	0.00149942	Q7TMM9	Tubulin beta-2A chain OS=Mus musculus OX=10090 GN= Tubb2a	1.23	0.69	9dpi_CTRL;7dpi_CTRL	TCA
17.0068	17.2194	17.845	16.9685	17.7242	16.8393	NaN	15.4709	15.1442	15.6049	14.2447	14.6509	2.34871	0.0115227	Q80YC5	Coagulation factor XII OS=Mus musculus OX=10090 GN=F F12	0.05	-1.28	7dpi_9dpi;CTRL_9dpi	DTT
24.3168	24.5759	24.3294	24.6049	23.2509	23.6744	23.3755	23.1832	22.9738	23.1863	23.1318	22.8555	5.4952	3.23E-05	Q8BH35	Complement component C8 beta chain OS=Mus musculu C8b	-0.87	-1.32	CTRL_9dpi;CTRL_7dpi	DD
20.5468	21.2722	20.7988	21.0095	18.4724	17.0809	18.9158	18.0341	17.0341	17.9417	18.6038	17.067	3.91456	0.000577237	Q8BH35	Complement component C8 beta chain OS=Mus musculu C8b	-1.86	-2.15	CTRL_9dpi;CTRL_7dpi	DTT
24.4491	24.6415	24.7453	24.9898	23.4179	23.5701	23.2978	23.3949	23.1725	23.3096	23.1728	22.7933	5.63912	2.50E-05	Q8BH35	Complement component C8 beta chain OS=Mus musculu C8b	-1.04	-1.33	CTRL_9dpi;CTRL_7dpi	TCA
19.076	19.6835	21.315	20.4761	17.8906	NaN	17.88	17.1238	18.3497	17.2783	17.8357	18.0033	2.71534	0.00568488	Q8BH61	Coagulation factor XIII A chain OS=Mus musculus OX=100 F13a1	-2.57	-2.27	CTRL_7dpi;CTRL_9dpi	DTT
23.8635	23.8612	23.8711	24.0985	24.5174	24.1041	24.3658	24.3402	24.49	24.4967	24.3662	23.9937	1.80594	0.0328372	Q8BND5	Sulfhydryl oxidase 1 OS=Mus musculus OX=10090 GN=Qs Qsox1	1.29	1.06	7dpi_CTRL;9dpi_CTRL	TCA
19.1116	19.1313	19.5645	19.431	18.6925	19.1586	19.2037	18.0256	17.9617	18.2899	18.4467	17.8805	2.24144	0.0139773	Q8BPB5	EGF-containing fibulin-like extracellular matrix protein 1 (Efemp1	-0.38	-0.72	CTRL_9dpi	TCA
26.4133	26.3592	26.7673	26.226	14.7012	NaN	15.6502	14.2297	NaN	NaN	NaN	NaN	6.17353	9.96E-06	Q8CG14	Complement C1s-A subcomponent OS=Mus musculus OX C1sa	-6.64	-6.64	CTRL_7dpi	DTT
19.4268	19.8246	20.0459	19.9387	19.6283	19.6083	19.2431	19.6168	18.1416	18.4111	18.8151	17.1643	2.96648	0.0034069	Q8CG14	Complement C1s-A subcomponent OS=Mus musculus OX C1sa	-0.12	-1.44	7dpi_9dpi;CTRL_9dpi	TCA
22.0591	23.5081	22.2053	22.8501	22.6652	22.1192	21.6221	22.6142	21.3995	20.6204	21.7332	21.1919	1.85998	0.0297889	Q8CG16	Complement C1r-A subcomponent OS=Mus musculus OX C1ra	-0.13	-1.21	CTRL_9dpi	TCA
18.9082	18.9754	18.4122	18.5608	17.9829	18.1475	17.8206	18.3833	17.998	16.8746	18.1427	17.7261	1.85472	0.029966	Q8K0D2	Hyaluronan-binding protein 2 OS=Mus musculus OX=100 Habp2	-0.51	-0.72	CTRL_9dpi	DD
23.1833	23.4892	23.1978	23.4785	22.5843	22.4853	22.7071	22.7445	21.7784	21.9922	21.8312	21.4734	5.78704	1.87E-05	Q8K182	Complement component C8 alpha chain OS=Mus muscul C8a	-0.81	-1.49	7dpi_9dpi;CTRL_9dpi;CTRL_7dpi	DD
21.4408	21.7038	21.4554	21.6395	18.5815	18.3376	19.3009	19.1232	18.7256	19.7607	19.7286	18.2249	4.1813	0.000340094	Q8K182	Complement component C8 alpha chain OS=Mus muscul C8a	-2.13	-1.7	CTRL_7dpi;CTRL_9dpi	DTT
23.7877	23.8804	23.7954	24.2233	22.6693	23.5219	23.2519	22.8561	22.3839	22.6357	22.4901	22.1395	3.94538	0.000545112	Q8K182	Complement component C8 alpha chain OS=Mus muscul C8a	-0.96	-1.51	7dpi_9dpi;CTRL_9dpi;CTRL_7dpi	TCA
20.0748	20.1134	20.0772	19.9116	19.6365	19.5575	19.8071	19.1217	20.0621	20.016	19.5617	20.0042	1.60902	0.0483037	Q8K1I3	Secreted phosphoprotein 24 OS=Mus musculus OX=1009 Spp2	-0.48	-0.09	CTRL_7dpi	DD
21.03	21.0647	20.7533	21.3186	20.2597	20.3079	20.2044	20.4316	20.596	20.7737	20.2403	20.4069	2.87712	0.0040926	Q8K1I3	Secreted phosphoprotein 24 OS=Mus musculus OX=1009 Spp2	-0.7	-0.47	CTRL_7dpi;CTRL_9dpi	TCA
21.8296	22.1721	21.5101	21.9096	22.0882	22.5655	22.456	22.6055	21.6285	21.8854	21.8313	21.5502	2.39671	0.0105506	Q8R121	Protein Z-dependent protease inhibitor OS=Mus musculu Serpina10	0.58	-0.24	7dpi_9dpi;7dpi_CTRL	DD
22.6642	23.1496	23.0668	23.0773	23.4326	23.5878	23.6436	23.4427	23.029	22.9887	23.024	22.3992	2.24812	0.0138902	Q8R121	Protein Z-dependent protease inhibitor OS=Mus musculu Serpina10	0.5	-0.12	7dpi_9dpi;7dpi_CTRL	TCA
23.5233	23.6499	23.4914	23.6597	22.4667	22.8683	22.6646	22.3929	22.2009	22.284	22.2937	22.2501	6.21029	9.69E-06	Q8VCG4	Complement component C8 gamma chain OS=Mus musc C8g	-1	-1.31	7dpi_9dpi;CTRL_9dpi;CTRL_7dpi	DD
24.2065	24.3199	24.2885	24.24	23.0956	22.9266	22.801	23.2098	22.9	23.2251	22.9762	22.6585	5.59665	2.65E-05	Q8VCG4	Complement component C8 gamma chain OS=Mus musc C8g	-1.15	-1.26	CTRL_9dpi;CTRL_7dpi	TCA
23.6797	23.7645	23.4793	23.6288	22.8054	23.166	22.6612	23.1553	23.2092	23.2534	23.3913	23.5817	2.71864	0.00566923	Q8VCS0	N-acetylmuramoyl-L-alanine amidase OS=Mus musculus Pglyrp2	0.45	0.75	9dpi_7dpi;CTRL_7dpi	DD
22.1792	22.2903	21.3807	23.418	20.8028	20.3159	24.1049	22.6364	19.877	19.2169	20.3128	17.9374	1.76843	0.0353559	Q8VCS0	N-acetylmuramoyl-L-alanine amidase OS=Mus musculus Pglyrp2	0.84	-0.88	7dpi_9dpi;CTRL_9dpi	DTT
22.3792	22.2885	22.8104	22.7164	22.8468	23.0299	23.1064	22.7675	23.2207	23.2218	22.9306	22.8922	1.86513	0.0295286	Q8VCS0	N-acetylmuramoyl-L-alanine amidase OS=Mus musculus Pglyrp2	0.6	0.78	9dpi_CTRL	TCA
16.7365	16.9358	15.7233	15.923	16.4464	16.1013	15.2918	15.3544	14.5041	14.523	NaN	NaN	1.75425	0.0358885	Q91WP0	Mannan-binding lectin serine protease 2 OS=Mus muscul Masp2	-0.56	-1.52	CTRL_9dpi	DD
22.9445	22.9717	22.8404	23.0055	25.5834	25.286	25.6446	25.9707	25.2064	24.9831	24.5341	25.0629	6.7638	4.29E-06	Q91WP6	Serine protease inhibitor A3N OS=Mus musculus OX=100 Serpina3n	3.04	2.37	9dpi_CTRL;7dpi_CTRL;7dpi_9dpi	DD
20.3624	20.8239	19.672	21.049	23.0396	22.6755	24.1868	22.5597	22.0827	21.9786	22.3339	20.2147	2.44935	0.00956267	Q91WP6	Serine protease inhibitor A3N OS=Mus musculus OX=100 Serpina3n	2.52	1.34	7dpi_CTRL	DTT
23.6935	23.578	23.437	23.3074	26.4551	26.1965	26.8665	26.4697	26.2535	25.801	25.7181	26.1586	7.22485	2.31E-06	Q91WP6	Serine protease inhibitor A3N OS=Mus musculus OX=100 Serpina3n	3.28	2.69	9dpi_CTRL;7dpi_CTRL;7dpi_9dpi	TCA
28.8655	28.8769	28.4845	28.7632	30.6433	30.7521	30.6969	30.8127	30.4752	30.4797	30.5124	30.4307	8.5333	2.92E-07	Q91X72	Hemopexin OS=Mus musculus OX=10090 GN=Hpx PE=1 S Hpx	1.92	1.77	9dpi_CTRL;7dpi_CTRL;7dpi_9dpi	DD
29.1474	29.2973	28.9727	29.0335	30.782	31.0025	31.174	30.8951	30.8156	30.7716	30.7768	30.6164	7.63557	1.22E-06	Q91X72	Hemopexin OS=Mus musculus OX=10090 GN=Hpx PE=1 S Hpx	1.83	1.58	9dpi_CTRL;7dpi_CTRL	TCA
31.0491	30.9925	30.9681	30.985	31.1032	31.3248	31.1886	31.2062	31.0736	31.1777	31.1048	31.0914	2.42596	0.010053	Q921I1	Serotransferrin OS=Mus musculus OX=10090 GN=Tf PE=1 Tf	0.29	0.09	7dpi_CTRL	DD
17.7541	16.254	16.9879	16.7743	19.5903	19.5383	18.7106	19.7763	19.9192	19.8779	19.8089	21.3649	3.98566	0.000507327	Q922U2	Keratin, type II cytoskeletal 5 OS=Mus musculus OX=1009 Krt5	2.24	2.85	7dpi_CTRL;9dpi_CTRL	DTT
14.5134	15.4261	15.1364	NaN	18.5148	19.3943	19.2174	18.637	18.379	18.1676	18.0538	18.1484	5.79902	1.85E-05	Q9CPY7	Cytosol aminopeptidase OS=Mus musculus OX=10090 GN Lap3	2.75	1.46	9dpi_CTRL;7dpi_CTRL;7dpi_9dpi	TCA
20.1793	20.982	20.6454	20.4339	17.1229	17.1035	18.5082	15.9032	16.2407	13.5723	17.1727	17.4266	2.86001	0.00420134	Q9CQW3	Vitamin K-dependent protein Z OS=Mus musculus OX=10 Proz	-2.62	-3.06	CTRL_9dpi;CTRL_7dpi	DD
21.4509	22.2726	22.1229	22.0419	19.2715	18.4404	17.4049	19.5726	18.7232	17.0818	18.0077	17.6766	4.3306	0.000256345	Q9CQW3	Vitamin K-dependent protein Z OS=Mus musculus OX=10 Proz	-2.66	-3.9	CTRL_9dpi;CTRL_7dpi	TCA
24.4071	24.581	24.5811	24.5376	23.1556	23.4033	23.3076	23.075	23.4358	23.4861	23.3557	23.2107	6.7833	4.25E-06	Q9DBB9	Carboxypeptidase N subunit 2 OS=Mus musculus OX=100 Cpn2	-1.21	-1.21	CTRL_7dpi;CTRL_9dpi	DD
21.9352	23.0986	22.3222	23.6247	20.8283	21.21	21.5794	20.9406	20.2407	20.0742	20.7295	19.373	3.40174	0.00157928	Q9DBB9	Carboxypeptidase N subunit 2 OS=Mus musculus OX=100 Cpn2	-1.34	-2.43	CTRL_9dpi;CTRL_7dpi	DTT
24.4522	24.7314	24.7331	24.8261	23.4884	23.8747	23.7943	23.4867	23.7339	23.7647	23.6534	23.3173	4.47917	0.000194318	Q9DBB9	Carboxypeptidase N subunit 2 OS=Mus musculus OX=100 Cpn2	-1.02	-1.07	CTRL_9dpi;CTRL_7dpi	TCA
25.1395	25.0585	25.1151	25.1089	25.3094	25.6388	25.3997	25.5388	25.6188	25.6836	25.6835	25.8638	4.16235	0.000352651	Q9DBD0	Inhibitor of carbonic anhydrase OS=Mus musculus OX=10 Ica	0.4	0.56	7dpi_CTRL;9dpi_CTRL;9dpi_7dpi	DD
25.3984	25.3314	25.2996	25.6995	25.7032	26.1779	26.2364	25.6583	25.9425	25.9906	25.9869	25.798	1.92541	0.0264408	Q9DBD0	Inhibitor of carbonic anhydrase OS=Mus musculus OX=10 Ica	0.54	0.54	9dpi_CTRL;7dpi_CTRL	TCA
23.5641	23.6615	20.7849	23.4974	24.4216	24.2745	24.662	25.5018	24.6938	24.2878	25.1569	24.8529	1.61407	0.0478811	Q9ESB3	Histidine-rich glycoprotein OS=Mus musculus OX=10090 Hrg	1.41	1.3	7dpi_CTRL;9dpi_CTRL	DD
20.82	21.2606	19.5833	21.3197	21.6324	23.8517	22.7906	22.4041	22.4618	22.3774	23.1163	23.057	2.19836	0.0151235	Q9ESB3	Histidine-rich glycoprotein OS=Mus musculus OX=10090 Hrg	1.48	1.76	7dpi_CTRL;9dpi_CTRL	DTT
23.48	23.7498	20.5337	23.5599	24.5118	24.9765	25.8815	24.889	24.8101	24.4667	25.3859	25.0405	1.76573	0.0354711	Q9ESB3	Histidine-rich glycoprotein OS=Mus musculus OX=10090 Hrg	1.8	1.5	9dpi_CTRL;7dpi_CTRL	TCA
20.3382	20.6926	19.7586	20.6322	21.158	21.5824	21.5551	21.6205	21.7811	21.5349	21.5449	21.1743	3.12797	0.00259558	Q9JHH6	Carboxypeptidase B2 OS=Mus musculus OX=10090 GN=C Cpb2	0.97	1.09	7dpi_CTRL;9dpi_CTRL	DD
20.2943	21.0092	19.7994	20.1476	21.3853	19.7983	20.5129	21.22	22.1235	21.8354	21.8238	21.2484	1.86838	0.0294001	Q9JHH6	Carboxypeptidase B2 OS=Mus musculus OX=10090 GN=C Cpb2	0.85	1.39	9dpi_CTRL	TCA
22.179	22.4878	22.3608	22.4375	20.8375	21.3625	21.3243	21.2977	21.1015	20.92	21.1099	21.0576	5.5269	3.05E-05	Q9JJN5	Carboxypeptidase N catalytic chain OS=Mus musculus OX Cpn1	-0.88	-1.11	CTRL_9dpi;CTRL_7dpi	DD
19.6595	19.6841	20.2912	20.3667	16.0657	16.469	17.3264	16.5789	16.7083	18.3058	18.7427	NaN	3.51196	0.00128398	Q9JJN5	Carboxypeptidase N catalytic chain OS=Mus musculus OX Cpn1	-2.6	-1.3	CTRL_7dpi;CTRL_9dpi	DTT
23.3058	23.6176	23.8238	23.8038	22.3994	22.747	22.0909	22.3992	22.5072	22.4252	22.39	21.9922	4.26765	0.00028511	Q9JJN5	Carboxypeptidase N catalytic chain OS=Mus musculus OX Cpn1	-1.09	-1.17	CTRL_9dpi;CTRL_7dpi	TCA
15.5277	15.3033	NaN	15.6398	18.4113	18.9081	18.8253	18.2624	17.9479	17.9731	17.4262	18.0048	5.9626	1.38E-05	Q9JM99	Proteoglycan 4 OS=Mus musculus OX=10090 GN=Prg4 PE Prg4	2.6	2.02	9dpi_CTRL;7dpi_CTRL;7dpi_9dpi	DD
16.083	16.5641	16.1135	16.0268	18.5648	18.6132	18.2439	17.9799	18.2724	18.6214	18.1813	18.2874	6.12986	1.05E-05	Q9QUM9	Proteasome subunit alpha type-6 OS=Mus musculus OX= Psma6	2.15	2.17	9dpi_CTRL;7dpi_CTRL	DD
17.6478	17.4873	17.6631	17.1355	19.7377	19.8532	19.4389	19.8919	20.4614	20.5341	19.9757	20.099	6.89194	3.89E-06	Q9QUM9	Proteasome subunit alpha type-6 OS=Mus musculus OX= Psma6	2.35	2.84	7dpi_CTRL;9dpi_CTRL;9dpi_7dpi	TCA
15.9448	16.6936	17.2666	16.2917	18.1035	16.9786	18.6017	19.1044	18.3412	18.0419	17.8069	19.4569	1.90156	0.0274084	Q9QUN9	Dickkopf-related protein 3 OS=Mus musculus OX=10090 Dkk3	1.22	1.19	7dpi_CTRL;9dpi_CTRL	DTT
24.2176	24.3608	23.9896	24.1484	24.7479	24.8032	24.6631	24.8854	25.0157	24.8945	25.2199	24.5978	3.22151	0.00218738	Q9QXC1	Fetuin-B OS=Mus musculus OX=10090 GN=Fetub PE=1 SV Fetub	0.6	0.8	7dpi_CTRL;9dpi_CTRL	DD
24.3261	24.7444	24.4185	24.544	25.0925	25.4111	25.3253	25.2891	25.1357	25.0207	25.7617	25.2059	2.86087	0.00420134	Q9QXC1	Fetuin-B OS=Mus musculus OX=10090 GN=Fetub PE=1 SV Fetub	0.65	0.67	7dpi_CTRL;9dpi_CTRL	TCA
20.7799	20.8963	20.7803	20.5807	20.7429	20.892	20.646	20.4781	20.203	20.5124	20.5091	20.3571	1.74464	0.0363722	Q9R098	Hepatocyte growth factor activator OS=Mus musculus OX Hgfac	-0.33	-0.45	CTRL_9dpi	DD
21.0381	21.534	21.4016	21.3479	20.7377	20.7682	20.4595	20.557	20.2795	20.6439	20.2816	20.2179	3.93811	0.000550521	Q9R098	Hepatocyte growth factor activator OS=Mus musculus OX Hgfac	-0.82	-0.99	CTRL_9dpi;CTRL_7dpi	TCA
NaN	14.4893	13.9141	NaN	16.9405	NaN	15.332	16.9372	17.7188	17.5133	NaN	16.911	2.03045	0.021375	Q9R1P0	Proteasome subunit alpha type-4 OS=Mus musculus OX= Psma4	2.45	3.13	7dpi_CTRL;9dpi_CTRL	TCA
14.8531	16.5217	15.6868	17.2063	17.0143	18.3525	17.938	17.6491	19.0278	19.1643	17.8765	18.4752	2.53699	0.00800064	Q9R1P4	Proteasome subunit alpha type-1 OS=Mus musculus OX= Psma1	1.09	1.95	7dpi_CTRL;9dpi_CTRL	TCA
16.4425	16.6294	16.8614	16.8244	18.3845	18.3717	18.4496	18.441	18.5624	18.3632	17.9679	18.0696	6.06159	1.16E-05	Q9WUU7	Cathepsin Z OS=Mus musculus OX=10090 GN=Ctsz PE=1 S Ctsz	1.42	1.51	9dpi_CTRL;7dpi_CTRL	DD
NaN	NaN	NaN	NaN	15.6746	14.8408	15.264	14.7714	13.8579	14.0982	13.7759	14.7006	1.88091	0.0286535	Q9Z0E6	Guanylate-binding protein 2 OS=Mus musculus OX=1009 Gbp2	6.64	6.64	CTRL_7dpi	TCA
15.8746	16.7393	16.178	15.3847	21.3217	21.3925	21.1058	22.3812	22.0713	22.1719	21.3037	22.5782	6.82422	4.12E-06	Q9Z0M9	Interleukin-18-binding protein OS=Mus musculus OX=100 Il18bp	5.23	5.06	7dpi_CTRL;9dpi_CTRL	DTT
22.113	22.0663	21.9592	21.9546	21.5007	21.7026	21.924	20.6728	20.8724	21.267	21.3192	20.3299	1.79474	0.0335773	Q9Z1R3	Apolipoprotein M OS=Mus musculus OX=10090 GN=Apoi Apom	-0.65	-0.92	CTRL_9dpi	DD
19.6669	20.3295	19.9387	20.6509	19.5965	18.834	19.3893	18.3355	17.9649	17.833	18.9312	16.7253	2.51471	0.00837311	Q9Z1R3	Apolipoprotein M OS=Mus musculus OX=10090 GN=Apoi Apom	-0.69	-1.94	CTRL_9dpi	DTT
17.2919	17.3989	17.6156	17.7443	16.335	17.1545	16.42	16.7237	NaN	15.9415	NaN	15.8283	3.14559	0.00253034	Q9Z1T2	Thrombospondin-4 OS=Mus musculus OX=10090 GN=Thl Thbs4	-0.37	-1.02	7dpi_9dpi;CTRL_9dpi;CTRL_7dpi	DD
17.2246	17.1868	17.8371	18.6578	16.6623	NaN	NaN	16.5859	16.2164	16.2169	15.9207	15.7582	2.35687	0.0113923	Q9Z1T2	Thrombospondin-4 OS=Mus musculus OX=10090 GN=Thl Thbs4	-0.91	-1.54	CTRL_9dpi	TCA
14.6187	14.6167	NaN	15.617	16.9425	NaN	16.986	18.7029	20.4924	21.352	21.2895	19.4841	3.82867	0.000671487	Q9Z2U1	Proteasome subunit alpha type-5 OS=Mus musculus OX= Psma5	2	3.07	7dpi_CTRL;9dpi_CTRL;9dpi_7dpi	DTT
16.5942	16.603	16.7636	16.4406	18.3095	19.0846	18.7345	18.4393	19.0334	19.1242	18.6381	19.2385	6.12735	1.05E-05	Q9Z2U1	Proteasome subunit alpha type-5 OS=Mus musculus OX= Psma5	2.3	2.52	7dpi_CTRL;9dpi_CTRL	TCA

Supplementary Table 6: analysis of variance of differentially regulated serum proteins in control (CTRL), 7DPI

Gene name	Method	-Log ANOVA p value	ANOVA q-value	Significant pairs
Apcs	DD	6.81364	0.00000412	9dpi_CTRL;7dpi_CTRL
Apcs	TCA	6.49261	6.18E-06	9dpi_CTRL;7dpi_CTRL
Crp	DD	3.29317	0.00190789	9dpi_CTRL;7dpi_CTRL
Crp	TCA	2.03787	0.0211524	9dpi_CTRL;7dpi_CTRL
F2	DD	2.85257	0.00425539	CTRL_9dpi;CTRL_7dpi
F2	TCA	1.84688	0.0303255	CTRL_7dpi;CTRL_9dpi
Fn1	DD	2.06913	0.0197486	9dpi_CTRL;7dpi_CTRL
Hp	DD	5.26098	0.0000455	CTRL_9dpi;CTRL_7dpi
Hp	DTT	4.70778	0.0001268	CTRL_9dpi;CTRL_7dpi
Hp	TCA	6.30228	8.69E-06	CTRL_9dpi;CTRL_7dpi
Itih4	DD	5.07825	0.00006356	9dpi_CTRL;7dpi_CTRL
Itih4	TCA	4.81935	0.0001057	9dpi_CTRL;7dpi_CTRL
Lbp	DD	6.04582	0.00001183	9dpi_CTRL;7dpi_CTRL
Lbp	TCA	5.31321	4.21E-05	7dpi_CTRL;9dpi_CTRL
Orm1	DD	7.61031	0.00000122	9dpi_CTRL;7dpi_CTRL
Orm1	DTT	1.77698	0.0348744	9dpi_CTRL;7dpi_CTRL
Orm1	TCA	6.23702	9.39E-06	9dpi_CTRL;7dpi_CTRL
Orm2	DD	7.31209	0.000002	9dpi_CTRL;7dpi_CTRL
Orm2	DTT	4.28007	0.0002813	9dpi_CTRL;7dpi_CTRL
Orm2	TCA	8.82123	2.75E-07	9dpi_CTRL;7dpi_CTRL
Saa1	DD	3.26532	0.00201046	9dpi_CTRL;7dpi_CTRL
Saa1	TCA	1.71152	0.0389163	CTRL_7dpi
Saa1	DD	7.32137	0.000002	9dpi_CTRL;7dpi_CTRL
Saa2	DTT	6.83887	4.12E-06	9dpi_CTRL;7dpi_CTRL
Saa2	TCA	6.55352	5.91E-06	9dpi_CTRL;7dpi_CTRL
Saa2	DD	6.50746	0.00000618	9dpi_CTRL;7dpi_CTRL
Saa3	DTT	7.07511	2.87E-06	9dpi_CTRL;7dpi_CTRL
Saa3	TCA	7.7498	1.03E-06	9dpi_CTRL;7dpi_CTRL;7dpi_9dpi
Saa4	DD	2.84513	0.00431015	7dpi_CTRL;7dpi_9dpi
Serpina1a	DD	4.87818	0.00009512	7dpi_9dpi;CTRL_9dpi;CTRL_7dpi
Serpina1a	TCA	4.67216	0.0001348	7dpi_9dpi;CTRL_9dpi;CTRL_7dpi

Serpina1b	DD	3.81779	0.00068409	7dpi_9dpi;CTRL_9dpi;CTRL_7dpi
Serpina3n	DD	6.7638	0.00000429	9dpi_CTRL;7dpi_CTRL;7dpi_9dpi
Serpina3n	DTT	2.44935	0.0095627	7dpi_CTRL
Serpina3n	TCA	7.22485	2.31E-06	9dpi_CTRL;7dpi_CTRL;7dpi_9dpi
Serpinf2	TCA	2.69447	0.0058934	CTRL_7dpi;CTRL_9dpi
Tfrc	DD	1.75485	0.0358885	CTRL_7dpi

Supplementary Table 7: analysis of variance per method of differentially regulated serum proteins in control (CTRL), 7DPI and 9DPI conditio

Gene name	Anova q-value		
	DD	TCA	DTT
Apcs	<0.0001	<0.0001	ns
Crp	<0.005	<0.05	ns
F2	<0.005	<0.05	ns
Fn1	<0.05	ns	ns
Hp	<0.0001	<0.0001	0.0001
Itih4	<0.0001	<0.0001	ns
Lbp	<0.0001	<0.0001	ns
Orm1	<0.0001	<0.0001	<0.05
Orm2	<0.0001	<0.0001	>0.0005
Saa1	<0.005	<0.0001	<0.05
Saa2	<0.0001	<0.0001	<0.0001
Saa3	<0.0001	<0.0001	ns
Saa4	<0.005	ns	ns
Serpina1a	<0.0001	0.0001	ns
Serpina1b	<0.0001	ns	ns
Serpina3n	<0.0001	ns	<0.05
Serpinf2	ns	0.005	ns
Tfrc	<0.05	ns	ns

Significance <0.05 by ANOVA and 9DPI conditions

ns.
